# Aurka-Bhlhe41 axis prevents premature aging-like microglial dysfunction and promotes remyelination

**DOI:** 10.1038/s41467-026-71014-w

**Published:** 2026-03-27

**Authors:** Weixing Yan, Yelin Zhao, Hui Li, Li Hong, Qi Jia, Di Zhu, Dong Xiang, Li Du, Lang Hu, Ruixue Bai, Meizhen Xu, Yangyang Tang, Xinzhu Chen, Yiwei Cao, Wenyu Jia, Siyu Wang, Yuting Liu, Jinfeng Ren, Shuai Pan, Yanbiao Shi, Sijia Gao, Fuxing Dong, Jianhong Shi, Jinghua Li, Kuiyang Zheng, Jing Yang, Shuli Zhao, Hui Wang

**Affiliations:** 1 Jiangsu Key Laboratory of Immunity and Metabolism, Department of Pathogen Biology and Immunology, School of Basic Medical Science, Xuzhou Medical University, Xuzhou, Jiangsu China; 2 National Experimental Demonstration Center for Basic Medicine Education, Xuzhou Medical University, Xuzhou, Jiangsu China; 3 Department of Clinical Sciences, Intervention and Technology, Medical Digital Twin Research Group, Division of ENT Diseases, Karolinska Institutet, Stockholm, Sweden; 4 Jiangsu Key Laboratory of Brain Disease Bioinformation, Xuzhou Medical University, Xuzhou, Jiangsu China; 5 Jinling Hospital, Affiliated Hospital of Medical School, Nanjing University, Nanjing, Jiangsu China; 6 Department of Dermatology, the Affiliated Hospital of Xuzhou Medical University, Xuzhou, Jiangsu China; 7 Public Experimental Research Center, Xuzhou Medical University, Xuzhou, Jiangsu China; 8 Hebei International Joint Research Center for Digital Twin Diagnosis and Treatment of Digestive Tract Tumors, Affiliated Hospital of Hebei University, Baoding, Hebei China; 9 General Clinical Research Center, Nanjing First Hospital, Nanjing Medical University, Nanjing, Jiangsu China

**Keywords:** Microglial cells, Neuroimmunology, Neurodegeneration

## Abstract

Aging accelerates central nervous system remyelination failure and neurodegeneration. Microglia promote remyelination by phagocytosing myelin debris, but this function is impaired by aging-related CD22 upregulation. However, the molecular mechanisms counteracting premature aging-related microglial dysfunction and remyelination impairment remain unclear. Here, we report that Aurka-Bhlhe41 axis prevents premature aging-like microglial dysfunction and promotes remyelination by restraining progressive CD22 upregulation. We identified that microglia-enriched Bhlhe41 was negatively autoregulated and inhibited by *Aurka* loss. *Bhlhe41-* or *Aurka*-deficient young mice exhibited aging-like microglial morphology, phagocytic deficits, progressive CD22 upregulation, and remyelination impairment in cuprizone-induced demyelination model. Conversely, ectopic Bhlhe41 expression induced hypertrophic microglia, and counteracted phagocytic deficits and CD22 upregulation in *Aurka*-deficient microglia. CD22 blockade restored phagocytic function and remyelination in *Bhlhe41*-deficient mice. Notably, a conserved pattern of *CD22* upregulation was observed in human *PCDH9*^high^ microglia subsets with *BHLHE41* downregulation. These findings offer insights into potential therapeutic strategies to combat aging-related neurodegeneration and central nervous system functional decline.

## Abstract (web summary)

In this study, the authors identify an Aurka–Bhlhe41 axis, which prevents microglia from aging-like dysfunction by limiting CD22 upregulation, preserving myelin debris clearance and remyelination, and highlighting a potential therapy target for neurodegeneration.

## Introduction

Aging is a primary risk factor for central nervous system (CNS) degeneration and neurodegenerative diseases, including Alzheimer’s disease (AD), Parkinson’s disease (PD), and multiple sclerosis (MS)^1^. A key pathological feature of these age-related CNS disorders is the accumulation of neurotoxic material, which contributes to neuronal damage and CNS dysfunction^1,2^. In the context of normal aging and MS, inhibitory myelin debris accumulated from demyelination inhibits the maturation of myelinating oligodendrocytes, leading to remyelination failure and neurodegeneration^2^. Microglia, the CNS resident macrophages, play a crucial role in promoting CNS remyelination by phagocytic clearance of myelin debris, thereby restoring neuronal health^2,3^. However, this function deteriorates with aging, which is increasingly recognized as essential to age-related CNS remyelination failure and neurodegeneration^2^.

Microglia, which originate from yolk sac myeloid progenitors before embryonic day 8 (E8) and reside in the brain between E9.5 and E14.5, are self-maintained in adulthood under physiological conditions^4–6^. With aging, microglia exhibit progressive dystrophy along with functional decline^7^. It is well documented that microglial phagocytic function is crucial for maintaining CNS homeostasis and is positively regulated by multiple phagocytic receptors, including triggering receptor expressed on myeloid cells 2 (TREM2), MER proto-oncogene, tyrosine kinase (MERTK) and C-X3-C motif chemokine receptor 1 (CX3CR1), along with Toll-like receptors (TLRs) and scavenger receptors^8–13^. Remarkably, mutations in *TREM2* have been associated with multiple neurodegenerative diseases^14,15^. In contrast, CD22, which is barely detected in young microglia, is upregulated during aging and impairs microglial phagocytic clearance of myelin debris^16^. Targeting CD22 with neutralizing antibodies restores aging microglial function and promotes CNS homeostasis^16^. Nevertheless, the intrinsic molecular mechanisms that counteract premature aging-related microglial dysfunction and CNS remyelination impairment remain poorly understood.

Transcription repressor basic helix-loop-helix family member e41(Bhlhe41), also known as Dec2, has been reported to regulate the molecular clock and sleep length in mammals^17,18^. In the immune system, Bhlhe41 is highly expressed in B-1a cells, alveolar macrophages (AM) and microglia^19–21^. Bhlhe41 regulates the self-renewal of B-1a cells and AM^19,20^, but its function in microglia remains unknown.

In this work, we dissect the intrinsic role of Bhlhe41 in microglial biology by integrating genetic mouse models and a cuprizone (CPZ)-induced demyelination model. We show that loss of *Bhlhe41* leads to premature aging-like microglial dysfunction, including dystrophic-like morphology, phagocytic deficits, progressive CD22 upregulation and remyelination impairment. Unexpectedly, we identify the mitotic kinase aurora kinase A (Aurka) as an upstream regulator of Bhlhe41, establishing an Aurka–Bhlhe41 axis that counteracts premature aging-like microglial dysfunction and promotes remyelination by restraining progressive CD22 upregulation.

## Results

### Bhlhe41 is microglia-enriched and negatively autoregulated

**Fig. 1:**
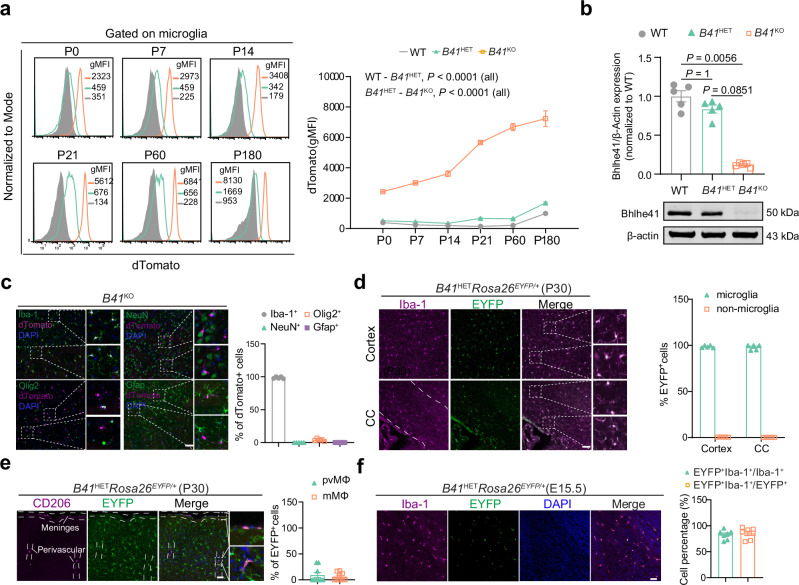
Bhlhe41 is microglia-enriched and negatively autoregulated in the mouse CNS. **a** Flow cytometric analysis of Bhlhe41 expression (dTomato^+^) in microglia from wildtype (WT), *Bhlhe41*^*dTomato-Cre/+*^ (referred to as *B41*^HET^) and *Bhlhe41*^*dTomato-Cre/dTomato-Cre*^ (referred to as *B41*^KO^) mice (*n* = 4 mice per genotype per timepoint) at indicated postnatal (P) days. Fluorescence intensity histograms are normalized to their respective modes. gMFI, geometric mean fluorescence intensity. WT vs *B41*^HET^, *P* < 0.0001 at all timepoints; *B41*^HET^ vs *B41*^KO^, *P* < 0.0001 at all timepoints. **b** Western blot (WB) analysis of Bhlhe41 expression in WT, *B41*^HET^ and *B41*^KO^ microglia (*n* = 5 mice per genotype, 3-months-old). Band intensities were quantified using ImageJ. **c** Immunofluorescence (IF) analysis of Bhlhe41 expression (dTomato^+^) in astrocytes (Gfap^+^), pan-oligodendrocytes (Olig2^+^), neurons (NeuN^+^) and microglia (Iba-1^+^) in the cerebral cortex of *B41*^KO^ mice (*n* = 5 mice) at 8 weeks of age. **d** IF analysis of EYFP^+^ cells in the cerebral cortex and corpus callosum (CC) of *B41*^HET^*Rosa26*^*EYFP/+*^ mice (*n* = 5 mice) at P30. **e** IF analysis of EYFP^+^ perivascular macrophages (pvMΦ, CD206^+^) and leptomeningeal macrophages (mMΦ, CD206^+^) from the cerebral cortex of *B41*^HET^*Rosa26*^*EYFP/+*^ mice (*n* = 3 mice) at P30. Each brain region represents a single data point, and three different regions per mouse were included in the quantification analysis. **f** IF analysis of EYFP^+^ cells in the CNS of *B41*^HET^*Rosa26*^*EYFP/+*^ mouse embryos (*n* = 7 embryos) at E15.5. Data are presented as mean ± SEM. Two-way ANOVA on log2-transformed gMFI values with Bonferroni-corrected post hoc comparisons (**a**); Kruskal–Wallis test with Bonferroni-corrected post hoc comparisons (**b**). dTomato (AF555, **c**), Iba-1 (AF555, **d**, **f**) and CD206 (AF555, **e**) shown in magenta (pseudocolor). Scale bar for IF images: 50 μm. Data are shown for descriptive purposes only with no inferential statistics applied (**c**–**f**). Source data are provided as a Source Data file.

We and others have reported that Bhlhe41 is preferentially expressed in mouse microglia, AM and B-1 lymphocytes in the immune system^19–21^. To further elucidate Bhlhe41 expression in microglia during development, we first analyzed public RNA sequencing data from yolk sac myeloid progenitors and microglia across embryonic, postnatal, and adult stages^22^. *Bhlhe41* is barely detected in yolk sac myeloid progenitors, but notably, it is observed in microglia from embryonic days 11.5 (E11.5) and increases progressively during development (Supplementary Fig. 1a). To validate this finding, we used Bhlhe41 reporter mice, *Bhlhe41*^*dTomato-Cre*^, in which a *dTomato-2A-Cre-polyA* cassette was inserted into the upstream region of the start codon of *Bhlhe41* locus. This resulted in *Bhlhe41* promoter-driven co-expression of dTomato and Cre, with Bhlhe41 translation terminated. In agreement with the RNA sequencing data, we failed to detect dTomato expression in yolk sac myeloid progenitors during E10.5 and E14.5 (Supplementary Fig. 1b). However, dTomato in microglia (Supplementary Fig. 1c) from *Bhlhe41*^*dTomato-Cre/+*^ mice (hereafter referred to as *B41*^HET^ mice) increased in an age-dependent manner (Fig. 1a). Of note, *Bhlhe41* deficiency in *Bhlhe41*^*dTomato-Cre/dTomato-Cre*^ mice (hereafter referred to as *B41*^KO^ mice) increased dTomato levels by 5–10 times in microglia, compared to a twofold increase in AM and peritoneal B-1 cells (Fig. 1a, Supplementary Fig. 1d, e), indicating potential microglia-specific self-repression of *Bhlhe41* promoter activity. Further Western blot (WB) analysis was consistent with potential microglial self-repression of Bhlhe41, with *B41*^HET^ microglia showing only a slight reduction rather than the expected 50%, and complete loss in *B41*^KO^ microglia (Fig. 1b).

To assess whether Bhlhe41 was specific to microglia in the mouse CNS, we first performed immunofluorescence (IF) analysis of Bhlhe41^+^ cells (dTomato^+^) in the CNS of *B41*^KO^ mice, as dTomato levels in *B41*^HET^ microglia were too low to be detected by IF. Notably, Bhlhe41 expression was enriched in microglia (Iba-1^+^) rather than astrocytes (Gfap^+^) or neurons (NeuN^+^), and was observed to a much lesser extent in pan-oligodendrocyte lineage cells (Olig2^+^) (Fig. 1c). *Bhlhe41* deficiency may affect the analysis of its expression profile in the CNS. To further corroborate this finding, we analyzed *B41*^HET^*Rosa26*^*EYFP/+*^ mice (P7 and P30), in which Bhlhe41-expressing cells were irreversibly and persistently labeled as EYFP^+^ cells. More than 99% of microglia, but only a few other CNS-resident cells and ~10% of CNS-associated macrophages (CAM), including perivascular and leptomeningeal macrophages (CD206^+^) were EYFP^+^ (Fig. 1d, e, Supplementary Fig. 2a). Further analysis revealed >80% of EYFP^+^ fetal microglia in *B41*^HET^*Rosa26*^*EYFP/+*^ mice at E15.5 (Fig. 1f, Supplementary Fig. 2b), corroborating Bhlhe41 expression early in fetal microglia. Altogether, these data demonstrate that Bhlhe41 is microglia-enriched and negatively autoregulated in the mouse CNS, and *B41*^HET^ mice may be a useful genetic tool for targeting and tracing microglia in vivo.

### *Bhlhe41*-deficient young microglia exhibit aging-like *deficits*

**Fig. 2:**
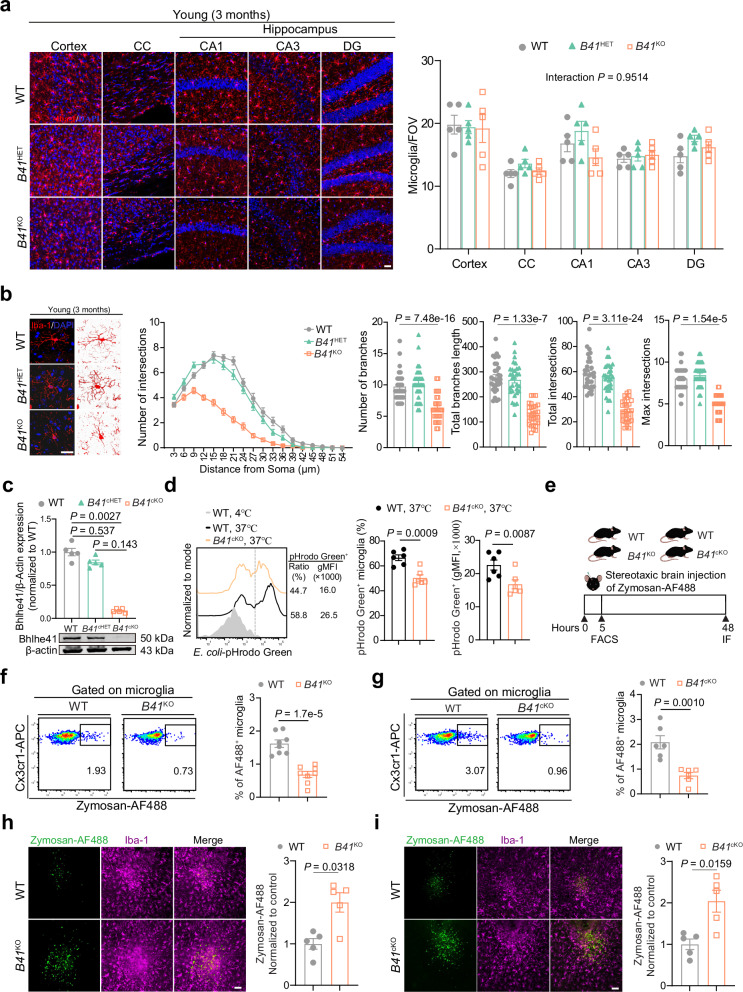
*Bhlhe41*-deficient microglia exhibit aging-like morphology and phagocytic deficits. **a** IF analysis of microglia in cerebral cortex, corpus callosum (CC) and hippocampus of WT, *B41*^HET^ and *B41*^KO^ mice (3-months-old; *n* = 5 mice per genotype). CA, cornu ammonis; DG, dentate gyrus. **b** Sholl analysis of microglial processes and branch intersections of WT, *B41*^HET^ and *B41*^KO^ mice (3 months old; *n* = 6 mice per genotype). Five microglia per mouse were quantified. **c** WB analysis of Bhlhe41 expression in *Cx3cr1*^*Cre/+*^(WT), *Cx3cr1*^*Cre/+*^*Bhlhe41*^*fl/+*^ (*B41*^cHET^) and *Cx3cr1*^*Cre/+*^*Bhlhe41*^*fl/fl*^ (*B41*^cKO^) microglia (3-months-old; *n* = 5 mice per genotype). Band intensities were quantified using ImageJ. **d** Flow cytometric analysis of pHrodo Green uptake in enriched microglia from WT and *B41*^cKO^ mice (3-months-old; n = 6 mice per genotype). **e** Schematic representation of in vivo phagocytosis assay. 2 μL of Zymosan conjugated with Zymosan-AF488 (0.5 mg/mL) were stereotaxically injected into the cortex of male *B41*^KO^, *B41*^cKO^ and WT mice (3-months-old), which were sacrificed after 5 hours and 48 hours, respectively, for subsequent analyses. Created in BioRender. Zhao, Y. (2026) https://BioRender.com/osy77p2. **f**, **g** Flow cytometric analysis of microglial phagocytosing Zymosan (AF488^+^) from the entire brain of **f** WT and *B41*^KO^ mice (*n* = 8 mice per genotype) and **g** WT and *B41*^cKO^ (*n* = 6 mice per genotype). **h**, **i** IF analysis of Zymosan clearance (AF488^+^) in the cortex of **h** WT and *B41*^cKO^ mice (*n* = 5 mice per genotype) and **i** WT and *B41*^cKO^ mice (*n* = 5 mice per genotype). Data are presented as mean ± SEM. Poisson generalized linear model (GLM, two-sided) (**a**); linear mixed-effects models (LMM) for continuous data and negative binomial generalized linear mixed-effects models (GLMM) for count data (two-sided) were used, with repeated measures from the same mouse accounted for as a random effect, and Bonferroni-adjusted post hoc pairwise comparisons applied (**b**); Kruskal–Wallis test with Bonferroni-corrected post hoc comparisons (**c**); two-sided unpaired Student’s *t* tests (**f**, **g**). Two-sided Wilcoxon rank-sum test (**d**, **h**, **i**). Iba-1(AF555, **h**, **i**) shown in magenta (pseudocolor). Scale bar for IF images in (**b**) is 10 μm, while the others are 50 μm. Source data are provided as a Source Data file.

Bhlhe41 regulates the self-renewal of B-1a cells and AM^19,20^. To examine whether Bhlhe41 was required for maintaining microglia, we assessed microglial populations in young and middle-aged *B41*^KO^ mice. *Bhlhe41* deficiency did not affect microglial numbers at either age (Fig. 2a, Supplementary Fig. 3a). However, while wildtype (WT) and *B41*^HET^ microglia displayed a typical ramified morphology, young *Bhlhe41*-deficient microglia exhibited aging-like morphology characterized by shorter processes and fewer branch intersections, which became more evident in middle-aged microglia (Fig. 2b, Supplementary Fig. 3b)^7^. To confirm whether this abnormality was due to intrinsic *Bhlhe41* deficiency in microglia, we generated a *Bhlhe41* conditional knockout (KO) mouse, *Bhlhe41*^*fl/fl*^, in which a 2.4 kb genomic region containing the entire *Bhlhe41* coding sequence was *loxP* flanked. Using microglia-specific Cre mice *Cx3cr1*^*Cre*^, we obtained microglia-specific *Bhlhe41* knockout mice, *Cx3cr1*^*Cre/+*^*Bhlhe41*^*fl/fl*^ (hereafter referred to as *B41*^cKO^), as well as heterozygous controls *Cx3cr1*^*Cre/+*^*Bhlhe41*^*fl/+*^ (hereafter referred to as *B41*^cHET^), and Cre controls, *Cx3cr1*^*Cre/+*^ (hereafter referred to as WT). WB analysis showed efficient Bhlhe41 ablation in *B41*^cKO^ microglia, while *B41*^cHET^ microglia exhibited only a slight, less-than-expected 50% reduction, consistent with potential microglial self-repression of Bhlhe41 (Fig. 2c). Microglial numbers were comparable across middle-aged WT, *B41*^cHET^ and *B41*^cKO^ mice (Supplementary Fig. 3c). While WT and *B41*^cHET^ microglia retained a ramified morphology, middle-aged *B41*^cKO^ microglia exhibited dystrophic-like features, including spheroidal swelling, de-ramified and fragmented processes^23^ (Supplementary Fig. 3d).

Microglial dystrophy suggests potential functional impairment^24^. We therefore assessed whether Bhlhe41 regulates microglial phagocytic function using pHrodo Green–labeled *E. coli* and Alexa fluor 488 (AF488)-labeled Zymosan A (*S. cerevisiae*) as sensitive, generalizable screening targets. pHrodo Green fluoresces only in acidic compartments such as phagosomes, enabling assessment of particle internalization, whereas AF488 is constitutively fluorescent and was used to evaluate overall particle clearance in vivo.

In vitro phagocytosis assay revealed that *B41*^cKO^ microglia exhibit impaired uptake of pHrodo Green–labeled *E. coli*, as evidenced by both a reduced fraction of pHrodo Green^+^ microglia and lower geometric mean fluorescence intensity (gMFI) per cell compared to WT microglia (Fig. 2d). To confirm this finding in vivo, we performed stereotactic injection of AF488-Zymosan into the cerebral cortex of *B41*^KO^ and *B41*^cKO^ mice (Fig. 2e). Unlike the pHrodo-based assay, AF488 is not restricted to acidic compartments and therefore reflects overall particle clearance rather than specific internalization. The injected Zymosan recruits and activates microglia, which phagocytose the Zymosan bioparticles^25^. However, 5 hours after injection, ablation of *Bhlhe41* in both *B41*^KO^ and *B41*^cKO^ mice resulted in fewer microglial phagocytosing Zymosan (Fig. 2f, g). Consistently, accumulated Zymosan was detected at the injection site of both *Bhlhe41*-deficient models (Fig. 2h, i), indicating impaired Zymosan clearance due to loss of *Bhlhe41*.

Taken together, these results demonstrate that Bhlhe41 is essential for maintaining microglial morphology and phagocytic function.

### *Bhlhe41* loss disrupts microglial responses to remyelination

**Fig. 3:**
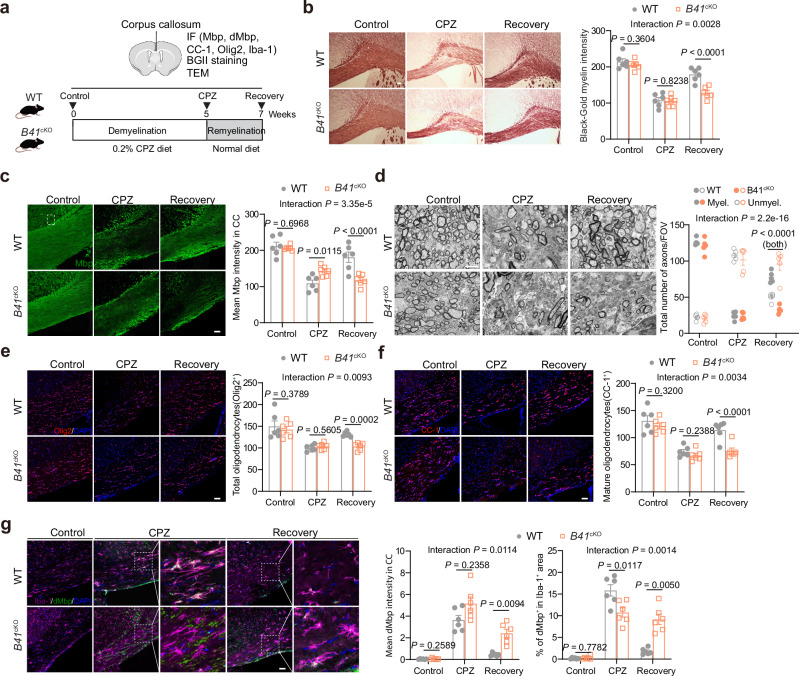
*Bhlhe41* deficiency disrupts microglial responses to remyelination in CPZ-induced demyelination model. **a** Schematic representation of the CPZ-induced demyelination model. Male WT and *B41*^cKO^ mice (3-months-old) were fed a 0.2% CPZ-enriched diet for 5 weeks to induce demyelination (denoted as the CPZ group) or followed by a CPZ-free normal diet for an additional 2 weeks (denoted as the Recovery group). WT and *B41*^cKO^ mice fed a CPZ-free normal diet were sacrificed as the control group. The CC of WT and *B41*^cKO^ mice from the control, CPZ and Recovery groups were collected for the subsequent analyses. Created in BioRender. Zhao, Y. (2026) https://BioRender.com/osy77p2. **b** Representative Black Gold II (BGII) staining images and quantification of myelinated area. **c** Representative IF images of Mbp staining and quantification of Mbp^+^ area. **d** Representative transmission electron microscopy (TEM) images of myelinated and unmyelinated axon ultrastructure. Myelinated and unmyelinated axons were counted in five fields of view (FOV) per mouse and were averaged per mouse. Stacked bar graphs show the numbers of myelinated and unmyelinated axons per FOV. Myelinated and unmyelinated axons: *P* < 0.0001, WT vs *B41*^cKO^. **e**, **f** Representative IF images and quantification of **e** pan-oligodendrocyte lineage cells (Olig2^+^) and **f** mature oligodendrocytes (CC-1^+^). **g** Representative IF images and quantification of the colocalization of degraded Mbp (dMbp) with microglia (Iba-1^+^). Data are presented as mean ± SEM (*n* = 6 mice per genotype per group for all panels except (**d**), where *n* = 5 mice per genotype per group). Iba-1(AF555) shown in magenta (pseudocolor). Two-way ANOVA with Bonferroni-corrected post hoc comparisons (**b**, **c**); LMM with Bonferroni-corrected post hoc comparisons (two-sided, **d**); ART-ANOVA with Bonferroni-corrected post hoc comparisons (**g**); Negative binomial GLM with Bonferroni-corrected post hoc comparisons (two-sided, **e**, **f**). Scale bar for IF images: 50 μm. Source data are provided as a Source Data file.

During aging and MS, microglia promote CNS remyelination by facilitating the phagocytic clearance of myelin debris^12^. To examine whether *Bhlhe41* deficiency affects CNS myelination, we utilized the CPZ-induced demyelination model, a toxin-induced, non-immune-mediated system in which demyelination occurs without peripheral immune cell infiltration^26^. Five weeks of CPZ diet exposure induces the loss of myelinating oligodendrocytes, microglial activation and demyelination in the corpus callosum (CC). Subsequent exposure to a CPZ-free diet for two weeks leads to remyelination (Fig. 3a). We analyzed myelination in the CC of WT and *B41*^cKO^ mice using Black Gold II (BGII) staining to assess intact myelin, IF analysis of major basic protein (Mbp) to detect total myelin, and transmission electron microscopy (TEM) analysis to examine unmyelinated and myelinated axons. Despite dystrophic-like morphology and phagocytic deficits in *B41*^cKO^ microglia, myelination in the CC of WT and *B41*^cKO^ mice was comparable under steady-state conditions (Fig. 3b–d). After 5 weeks of CPZ treatment, both WT and *B41*^cKO^ mice exhibited significant but similar demyelination, evidenced by reduced BGII staining and Mbp levels, and increased unmyelinated axons (Fig. 3b–d). However, while WT mice displayed remyelination in the CC after being fed a CPZ-free diet for an additional two weeks, *B41*^cKO^ mice exhibited delayed remyelination in the CC, characterized by a higher proportion of unmyelinated axons (Fig. 3d).

Microglial clearance of myelin debris is critical for remyelination by promoting the maturation of myelinating oligodendrocytes^11^. We therefore analyzed pan-oligodendrocyte lineage cells (Olig2^+^) and mature oligodendrocytes (CC-1^+^) in the CC of WT and *B41*^cKO^ mice in the CPZ-induced demyelination model. While loss of oligodendrocytes was observed in both WT and *B41*^cKO^ mice during the demyelination phase, mature oligodendrocytes in the CC of *B41*^KO^ mice failed to repopulate during the recovery phase (Fig. 3e, f). We next co-stained degraded Mbp (dMbp), a marker of myelin debris, with Iba-1 to assess microglial phagocytosis. Although overall demyelination was comparable between WT and *B41*^cKO^ mice (Fig. 3b–d), *B41*^cKO^ mice accumulated more myelin debris (dMbp^+^) during the demyelination phase, accompanied by markedly reduced colocalization with microglia (Iba-1^+^), indicating impaired debris uptake (Fig. 3g). During recovery, WT mice exhibited low levels of residual myelin debris, whereas *B41*^cKO^ mice retained elevated debris (Fig. 3g). Notably, increased colocalization of Iba-1 with dMbp in *B41*^cKO^ mice indicates delayed microglial clearance and impaired remyelination (Fig. 3g).

Altogether, these data demonstrate that Bhlhe41 promotes remyelination by facilitating phagocytic clearance of myelin debris.

### *Aurka* loss impairs Bhlhe41, microglial function and remyelination

**Fig. 4:**
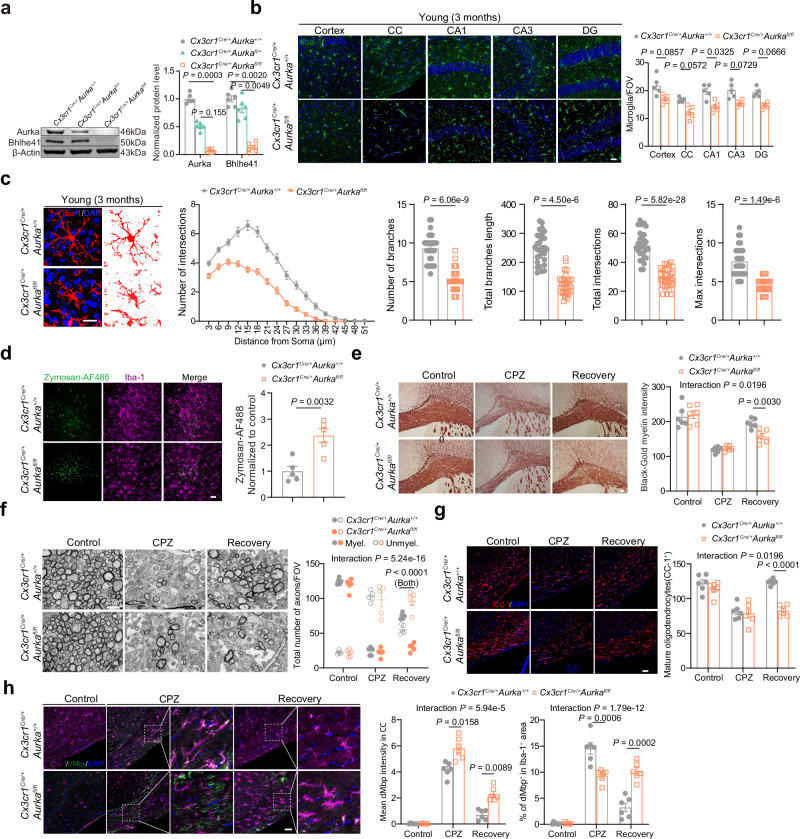
Loss of *Aurka* inhibits Bhlhe41 expression, and disrupts microglial morphology, phagocytic function and responses to remyelination. **a** WB analysis of Aurka and Bhlhe41 expression in microglia from WT, *Cx3cr1*^Cre/+^*Aurka*^*fl/+*^ and *Cx3cr1*^Cre/+^*Aurka*^*fl/fl*^ mice (3-months-old, *n* = 5 mice per genotype). Band intensities were quantified using ImageJ. **b** IF analysis of microglial number in the cerebral cortex, CC and hippocampus of *Cx3cr1*^*Cre/+*^*Aurka*^*+/+*^ and *Cx3cr1*^*Cre/+*^*Aurka*^*fl/fl*^ mice (3-months-old, *n* = 5 mice per genotype). **c** Sholl analysis of microglial processes and branch intersections in young WT and *Cx3cr1*^*Cre/+*^*Aurka*^*fl/fl*^ mice (3-months-old, *n* = 6 mice per genotype). Five microglia per mouse were quantified. **d** IF analysis of Zymosan clearance (AF488^+^) 48 hours after stereotaxic injection of 2 μL of Zymosan-AF488 (0.5 mg/mL) into the cortex of WT and *Cx3cr1*^*Cre/+*^*Aurka*^*fl/fl*^ mice (3-months-old, n = 5 mice per genotype). **e**–**h** The CPZ-induced demyelination model was induced with male WT and *Cx3cr1*^*Cre/+*^*Aurka*^*fl/fl*^ mice (3-months-old, *n* = 6 mice per genotype per group) following the experimental design in Fig. 3a. **e** Representative BGII staining images and quantification of myelinated area. **f** Representative TEM images of myelinated and unmyelinated axon ultrastructure. Myelinated and unmyelinated axons were counted in five fields of view (FOV) per mouse, averaged per mouse and shown in stacked bar graphs. **g**, **h** Representative IF images of **g** mature oligodendrocytes (CC-1^+^) and **h** colocalization of dMbp with microglia (Iba-1^+^). Data are presented as mean ± SEM. Kruskal-Wallis test with Bonferroni-corrected post hoc comparisons (**a**); Poisson GLM with Bonferroni-corrected post hoc comparisons (two-sided, **b**, **g**); LMM for continuous data and negative binomial GLMM for count data, with repeated measures from the same mouse accounted for as a random effect and post hoc pairwise comparisons adjusted using the Bonferroni correction (two-sided, **c**); two-sided unpaired Student’s *t* tests (**d**); two-way ANOVA with Bonferroni-corrected post hoc comparisons (**e**); Weighted LMM with Bonferroni-corrected post hoc comparisons (two-sided, **f**); ART-ANOVA with Bonferroni-corrected post hoc comparisons (two-sided, **h**). Iba-1 (AF647) shown in magenta, CD22 (AF555) shown in green (pseudocolor). Scale bar for IF images in (**c**) is 10 μm, while the others are 50 μm. Source data are provided as a Source Data file.

In a B cell study, we unexpectedly identified that Bhlhe41 expression in B-1 cells was inhibited by loss of *Aurka* (Supplementary Fig. 4a), a mitotic kinase that regulates mitosis and multiple other cellular events in mitotic kinase-dependent and independent manners^27,28^. To examine whether Aurka regulated Bhlhe41 expression in microglia, we analyzed the Bhlhe41 expression (dTomato^+^) in *B41*^HET^*Aurka*^*fl/fl*^ microglia, in which *loxP* flanked the exon 3 of *Aurka* was deleted by *Bhlhe41*-promoter-driven Cre recombinase^29^. Compared to *B41*^HET^*Aurka*^*fl/+*^ microglia, the gMFI of dTomato was markedly reduced in *Aurka*-deficient microglia, reaching the background level observed in WT microglia (Supplementary Fig. 4b). Consistently, WB analysis confirmed that *Aurka* ablation inhibited Bhlhe41 expression in microglia (Supplementary Fig. 4c). To further validate this finding, we generated independent microglia-specific *Aurka* knockout strain, *Cx3cr1*^*Cre/+*^*Aurka*^*fl/fl*^, in which Bhlhe41 expression was similarly diminished (Fig. 4a).

To explore whether inhibited Bhlhe41 expression in *Aurka*-deficient microglia was associated with disrupted mitosis, we analyzed Bhlhe41 expression in microglia deficient in *Aurkb*, another key mitotic enzyme that regulates chromosome segregation and cytokinesis^30^. Loss of *Aurkb* had no effect on Bhlhe41 expression in microglia, suggesting that Bhlhe41 expression is independent of mitosis (Supplementary Fig. 4d). These data demonstrate that Aurka controls Bhlhe41 expression in microglia.

This finding led us to examine the role of Aurka in regulating microglial number, morphology and phagocytic function. We first investigated microglial numbers and morphology in both *Cx3cr1*^*Cre/+*^*Aurka*^*fl/fl*^ and *B41*^HET^*Aurka*^*fl/fl*^ mice. Compared to WT mice, both young and middle-aged *Aurka*-deficient mice exhibited decreased microglial numbers and a rod-like morphology with diminished processes and branch intersections (Fig. 4b, c, Supplementary Fig. 5). *Aurka* deficiency leads to stalled mitosis with a consequence of arrested proliferation and increased apoptosis^31^. Therefore, we sought to examine if decreased microglia by *Aurka* deficiency was due to impaired mitosis and cell apoptosis. Considering that microglia proliferate explosively during the first two weeks^32^, we performed IF analysis of microglia in *B41*^HET^*Aurka*^*fl/fl*^ mice at P13. *Aurka* deficiency at P13 had no effect on microglial numbers yet (Supplementary Fig. 6a). However, this resulted in disturbed microglial proliferation in the cortex and increased apoptotic microglia in the cortex, subventricular zone (SVZ) and CC (Supplementary Fig. 6a, b). Loss of *Aurka* impaired microglial mitosis, identified by increased microglia positive for phospho-Histone H3, a marker for mitosis (Supplementary Fig. 6c). Despite unchanged microglia population, *B41*^HET^*Aurka*^*fl/fl*^ microglia at P13 displayed a rod-like morphology with reduced processes and branch intersections (Supplementary Fig. 6d). Notably, compared to infant *B41*^HET^*Aurka*^*fl/fl*^ mice and adult *Cx3cr1*^*Cre/+*^*Aurka*^*fl/fl*^ mice, less rod-like microglia were observed in young and middle-aged *B41*^HET^*Aurka*^*fl/fl*^ mice (Fig. 4b, Supplementary Fig. 5 and 6). Altogether, Aurka regulates microglia development in a mitotic kinase-dependent manner.

We next examined whether Aurka regulated microglial phagocytic function and responses to remyelination. To test this, we performed in vivo phagocytosis analysis by stereotactic injection of Zymosan-AF488 into the cortex of WT and *Cx3cr1*^*Cre/+*^*Aurka*^*fl/fl*^ mice, followed by IF analysis 48 hours post injection. *Aurka* deficiency resulted in increased extracellular accumulation of Zymosan (AF488^+^), suggesting that Aurka promotes microglial phagocytosis (Fig. 4d).

Using *Cx3cr1*^*Cre/+*^*Aurka*^*fl/fl*^ mice, we further examined whether *Aurka* deficiency impaired remyelination in CPZ-indued demyelination model, following the experimental design in Fig. 3a. At steady-state condition, the myelination was unaffected by *Aurka* deficiency (Fig. 4e, f). After CPZ treatment, both WT and *Aurka-*deficient mice exhibited significant but comparable demyelination in the CC (Fig. 4e, f). During recovery, *Aurka*-deficient mice remained exacerbated demyelination (Fig. 4e, f). Consistently, mature oligodendrocytes (CC-1^+^) failed to repopulate in the CC of *Aurka-*deficient mice during recovery (Fig. 4g). Further co-staining of dMbp with Iba-1 showed that, similar to *B41*^cKO^ mice, *Aurka*-deficient mice accumulated myelin debris (dMbp^+^) during both demyelination and recovery (Fig. 4h). Colocalization of dMbp with Iba-1 was initially reduced during demyelination but increased during recovery (Fig. 4h), indicating delayed microglial clearance of myelin debris and impaired remyelination. These data collectively suggest that Aurka is required for remyelination by sustaining microglial clearance of myelin debris.

### Premature CD22 upregulation in *Bhlhe41*- or *Aurka*-KO microglia

To understand the molecular mechanisms by which Bhlhe41 maintained microglial phagocytosis, we performed gene expression microarray (GEM) analysis of young *B41*^HET^ and *B41*^KO^ microglia. Next, we identified reliable differentially expressed (DE) genes by comparing in-house GEM data with public bulk RNA sequencing data from *Bhlhe40/Bhlhe41* double knockout microglia^33^. This led to the identification of 122 shared DE genes (Supplementary Data 1, Fig. 5a). *Cacng1*, which regulates calcium ion transmembrane transport, and *Upk1b*, which encodes a microglia-specific membrane receptor^34,35^, ranked as the top upregulated and downregulated genes, respectively. Several DE genes including *Plaur*, *Ptprg*, *Ccr5*, *Fcgr2b* and *Ciita* have been associated with microglial biology (Supplementary Data 1, Fig. 5a)^36–40^.

**Fig. 5:**
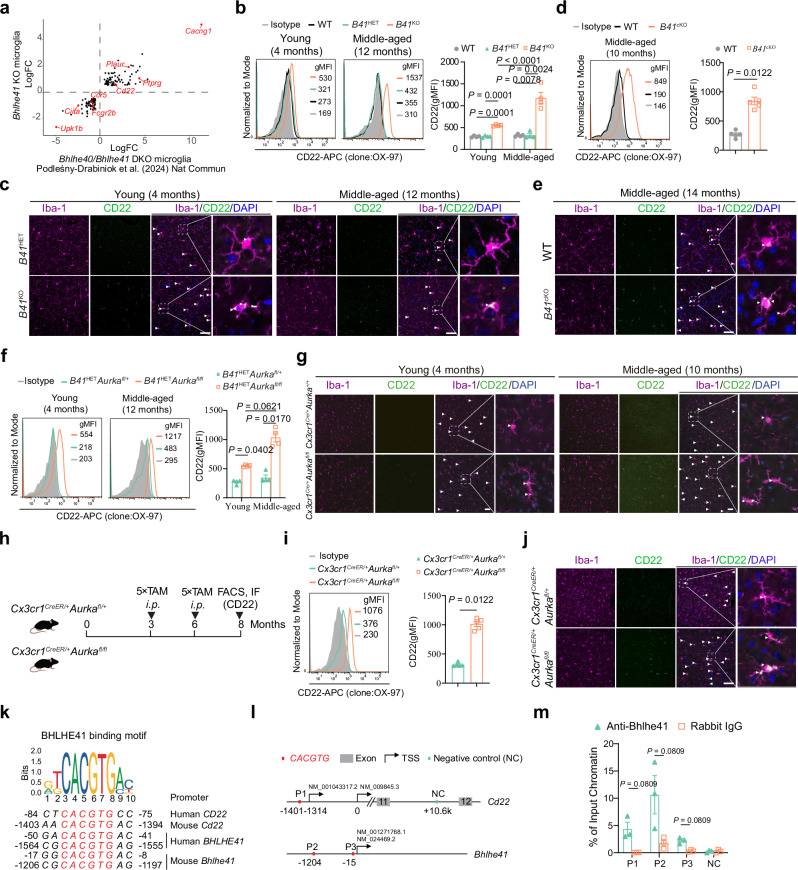
*Bhlhe41*- or *Aurka*-deficiency induces premature and progressive CD22 upregulation in microglia with age. **a** Gene expression microarray analysis (GEM) of microglia from *B41*^HET^ and *B41*^KO^ mice (3-months-old). Shared differentially expressed genes (DEGs) were identified by comparing DEGs from the in-house GEM data and public bulk RNA sequencing data from *Bhlhe40*/*Bhlhe41* double knockout (DKO) microglia. **b**–**g** Flow cytometric analysis (**b**, **d**, **f**) or representative IF images (**c**, **e**, **g**) of CD22 expression on (**b**, **c**) young and middle-aged WT, *B41*^HET^ and *B41*^KO^ microglia (*n* = 4 mice per genotype per age), **d**, **e** middle-aged WT and *B41*^cKO^ microglia (*n* = 5 mice per genotype), **f** young and middle-aged *B41*^HET^*Aurka*^*fl/+*^ and *B41*^HET^*Aurka*^*fl/fl*^ microglia (*n* = 4 mice per genotype per age), **g** middle-aged *Cx3cr1*^*Cre/+*^*Aurka*^*fl/+*^ and *Cx3cr1*^*Cre/+*^*Aurka*^*fl/fl*^ microglia (*n* = 3 mice per genotype). **h**–**j** *Cx3cr1*^*CreER/+*^*Aurka*^*fl/+*^ and *Cx3cr1*^*CreER/+*^*Aurka*^*fl/fl*^ mice injected with tamoxifen (TAM, 75 mg/kg) for 5 consecutive days at 3 and 6 months of age, were sacrificed at 8 months for flow cytometry (**i**, **j**) IF analysis of CD22 expression on microglia (*n* = 4 mice per genotype). Created in BioRender. Zhao, Y. (2026) https://BioRender.com/osy77p2. White arrows indicate areas of colocalization between CD22 and Iba-1, appearing as yellow. **k** Identification of the BHLHE41-binding motif *CACGTG* in the promoters of human and mouse *CD22* and *BHLHE41* using JASPAR database. **l** Schematic representation of mouse *Bhlhe41* and *Cd22* promoters showing P1-P3 containing the BHLHE41-binding motif and a negative control region (NC) used for Chromatin immunoprecipitation (ChIP)-qPCR. TSS, transcription start site. **m** ChIP was performed on fragmented chromatin of microglia pooled from 10 to 15 WT mice (3-months-old) per experiment (*n *= 3 biological replicates). Microglia were enriched by 40% Percoll and immunoprecipitated with an anti-Bhlhe41 antibody or rabbit IgG control. Enrichment of P1, P2, P3 and NC regions was analyzed by qPCR. Data are presented as mean ± SEM. Non-parametric ART-ANOVA with Bonferroni-corrected post hoc comparisons (two-sided, **b**, **f**); Two-sided Wilcoxon rank-sum test (**d**, **i**, **m**). Iba-1 (AF647) shown in magenta, CD22 (AF555) shown in green (pseudocolor). Scale bar for IF images: 50 μm. Source data are provided as a Source Data file.

Notably, *Cd22*, an aging-related phagocytic repressor^16^, was upregulated in young *B41*^KO^ microglia (Supplementary Data 1, Fig. 5a). In line with prior reports, we confirmed that CD22 expression in the mouse CNS was microglia-specific, being barely detectable in young microglia but prominent in aged populations (Supplementary Fig. 7a). This observation prompted us to hypothesize that Bhlhe41 represses CD22 expression in young microglia. Flow cytometric analysis validated this hypothesis, showing that CD22 was barely detected on both young and middle-aged (10–14 months) WT and *B41*^HET^ microglia (Fig. 5b). In contrast, CD22 was prematurely upregulated on young *B41*^KO^ microglia, and this upregulation was accelerated on middle-aged *B41*^KO^ microglia (Fig. 5b, Supplementary Fig. 7b). IF analysis confirmed that *B41*^KO^ microglia with dystrophic-like morphology exhibited CD22 upregulation (Fig. 5c). Further studies using *B41*^cKO^ mice corroborated an intrinsic effect of Bhlhe41 on CD22 suppression in microglia (Fig. 5d, e, Supplementary Fig. 7c). Nevertheless, CD22 expression was unaffected on *Bhlhe41*-deficient AM and B-1 cells (Supplementary Fig. 8), indicating microglia-specific CD22 suppression by Bhlhe41.

Considering inhibited Bhlhe41 expression in *Aurka*-deficient microglia, we further analyzed CD22 expression on *B41*^HET^*Aurka*^*fl/fl*^ microglia. Indeed, *Aurka* deficiency induced premature and progressive CD22 upregulation on young and middle-aged microglia (Fig. 5f). Given that CD22 upregulation on *B41*^HET^*Aurka*^*fl/fl*^ microglia might be affected by heterozygous deletion of *Bhlhe41*, we analyzed CD22 expression on *Cx3cr1*^*Cre/+*^*Aurka*^*fl/fl*^ microglia. Consistently, CD22 upregulation was observed on rod-like *Cx3cr1*^*Cre/+*^*Aurka*^*fl/fl*^ microglia (Fig. 5g). To further examine whether CD22 was upregulated on *Aurka-*deficient microglia that bypassed neonatal developmental deficits, we generated tamoxifen-inducible microglia-specific *Aurka* knockout model, *Cx3cr1*^*CreER/+*^*Aurka*^*fl/fl*^ (Fig. 5h). CD22 was upregulated upon tamoxifen-induced ablation of *Aurka* in adult microglia, despite a modest reduction in microglial numbers (Fig. 5i-j, Supplementary Fig. 9).

Bhlhe41 represses transcription through binding to the E-box motif *CACGTG*^19,20^. Notably, the promoters of human and murine *CD22* and *BHLHE41* contain the *CACGTG* motif (Fig. 5k). Therefore, we performed chromatin immunoprecipitation (ChIP)-qPCR analysis to determine whether Bhlhe41 is bound to the three sites, P1 in the *Cd22* promoter, and P2 and P3 in the *Bhlhe41* promoter (Fig. 5l). While a negative control DNA fragment located in intron 11 of *Cd22* was not enriched, ChIP-qPCR analysis revealed significant enrichment of P1, P2, and P3 in fragmented chromatin from purified microglia immunoprecipitated with anti-Bhlhe41 polyclonal antibody (Fig. 5m), indicating that Bhlhe41 directly binds to its own promoter and the *Cd22* promoter.

Taken together, Aurka and Bhlhe41 prevents premature aging-related CD22 upregulation in microglia.

### Bhlhe41 reverses Aurka-loss-induced CD22 and microglial deficits

To address whether *Aurka*-deficient microglial deficits was dependent on Bhlhe41, we generated *Bhlhe41* transgenic mice, *Rosa26*^*Bhlhe41-EYFP*^ (hereafter referred to as *R26*^*B41-EYFP*^). In *R26*^*B41-EYFP*^ mice, a *CAG* promoter-*loxP*-*PGK*-*Neo*-*6xSV40 pA*-*loxP*-Kozak-*Bhlhe41* CDS-*P2A*-*EYFP*-*WPRE*-*BGH pA* cassette was integrated into the *Rosa26* locus (Supplementary Fig. 10a). Upon Cre recombinase-mediated removal of the *loxP*-flanked DNA cassette, which prevents the expression of the downstream bicistronic sequences, Bhlhe41 and EYFP were independently co-expressed. WB analysis confirmed ectopic Bhlhe41 expression in microglia from microglia-specific *Bhlhe41* transgenic mice, *B41*^HET^*R26*^*B41-EYFP/+*^ (Supplementary Fig. 10b).

**Fig. 6:**
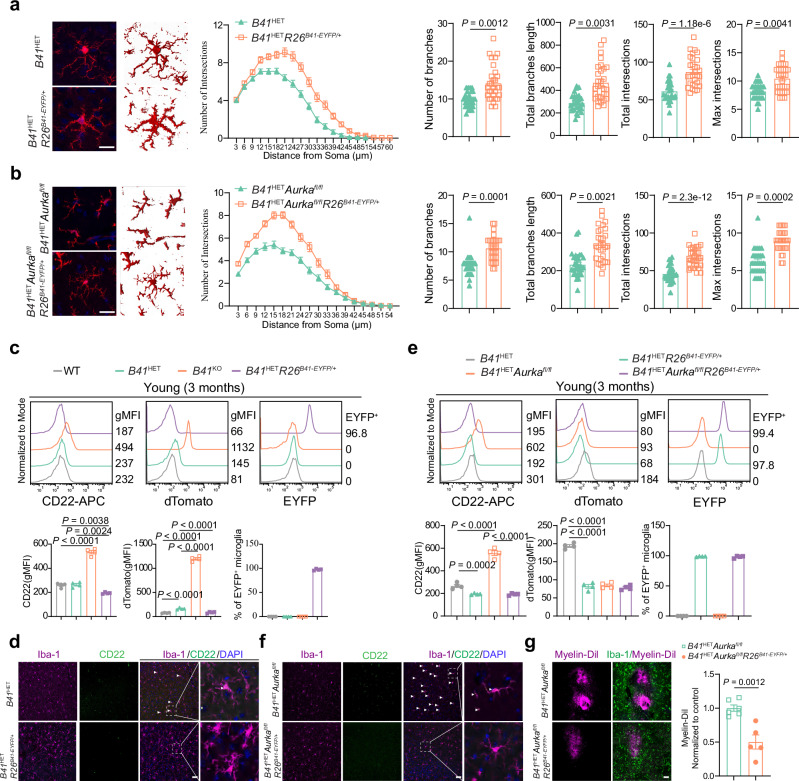
Ectopic Bhlhe41 expression induces hypertrophic microglia and reverses CD22 upregulation and phagocytic deficits in *Aurka*-deficient microglia. **a**, **b** Sholl analysis of microglial processes and branch intersections in **a** young *B41*^HET^ and *B41*^HET^*R26*^*B41-EYFP/+*^ mice (3-months-old) and **b** young *B41*^HET^*Aurka*^*fl/fl*^ and *B41*^HET^*Aurka*^*fl/fl*^ *R26*^*B41-EYFP/+*^ mice (3-months-old). *n* = 6 mice per genotype. Five microglia per mouse were quantified. **c**–**f** Flow cytometric analysis of CD22 and Bhlhe41 (dTomato^+^) expression in **c** young WT, *B41*^HET^, *B41*^KO^ and *B41*^HET^*R26*^*B41-EYFP/+*^ mice (3-months-old), **e** young *B41*^HET^, *B41*^HET^*R26*^*B41-EYFP/+*^, *B41*^HET^*Aurka*^*fl/fl*^ and *B41*^HET^*Aurka*^*fl/fl*^*R26*^*B41-EYFP/+*^ mice (3-months-old). Representative IF images of CD22 expression in microglia from **d** young *B41*^HET^ and *B41*^HET^*R26*^*B41-EYFP/+*^ mice (3-months-old), **f** young *B41*^HET^*Aurka*^*fl/fl*^ and *B41*^HET^*Aurka*^*fl/fl*^*R26*^*B41-EYFP/+*^ mice (3-months-old). *n* = 4 mice per genotype. Fluorescence intensity histograms are normalized to their respective modes. White arrows indicate areas of colocalization between CD22 and Iba-1, appearing as yellow. **g** Stereotaxic injection of 1 μL of myelin conjugated with Dil (Myelin-Dil, 20 mg/mL) into the cortex of male *B41*^HET^*Aurka*^*fl/fl*^ (3-months-old, n = 6 mice) and *B41*^HET^*Aurka*^*fl/fl*^*R26*^*B41-EYFP/+*^ mice (3-months-old, *n* = 5 mice), followed by sacrifice 48 hours after injection for IF analysis of myelin clearance (Dil^+^). Data are presented as mean ± SEM. LMM for continuous data and negative binomial GLMM for count data (two-sided) were used, with repeated measures from the same mouse accounted for as a random effect and Bonferroni-corrected post hoc comparisons (**a**, **b**); one-way ANOVA with Bonferroni-corrected post hoc comparisons (**c**, **e**); dTomato data were log2-transformed prior to analysis (**c**, **e**); two-sided unpaired Student’s *t* test (**g**). Iba-1 (AF647, **d**, **f**), Myelin-Dil (**g**) shown in magenta, CD22 (AF555) shown in Green (pseudocolor). Scale bar for IF images in (**a**, **b**) is 10 μm, while the others are 50 μm. Source data are provided as a Source Data file.

Ectopic Bhlhe41 expression mildly increased microglial numbers in the CA1 and CC of *B41*^HET^*R26*^*B41-EYFP/+*^ mice (Supplementary Fig. 11a). Of note, *B41*^HET^*R26*^*B41-EYFP/+*^ microglia displayed enlarged somas with thicker, shorter and more ramified processes, a typical hypertrophic morphology^24,41,42^ (Fig. 6a). Additionally, CD68, an activation marker of microglia, was induced in *Bhlhe41* transgenic microglia (Supplementary Fig. 11b). Using *R26*^*B41-EYFP*^ mice, we next rescued Bhlhe41 expression in *Aurka*-deficient microglia by generating *B41*^HET^*Aurka*^*fl/fl*^*R26*^*B41-EYFP/+*^ mice. In *Aurka*-deficient microglia, ectopic Bhlhe41 expression resulted in a morphology change from rod-like microglia to ramified microglia with long and branched processes (Fig. 6b). Collectively, these data reinforce the role of Bhlhe41 in maintaining microglial morphology and homeostasis and demonstrate that Bhlhe41 is sufficient to restore morphology deficits in *Aurka*-deficient microglia.

*Bhlhe41* deficiency enhanced *Bhlhe41* promoter activity and CD22 expression (Figs. 1a and  5b–e). Hence, we investigated whether ectopic Bhlhe41 expression inhibited *Bhlhe41* promoter activity and CD22 expression. The gMFI of dTomato in *B41*^HET^*R26*^*B41-EYFP/+*^ microglia was decreased to background levels observed in WT microglia, supporting potential Bhlhe41 self-repression in microglia (Fig. 6c). In contrast to CD22 upregulation on *B41*^KO^ microglia, ectopic Bhlhe41 expression suppressed CD22 expression (Fig. 6c, d), corroborating that Bhlhe41 represses its own expression as well as CD22 expression in microglia.

To assess whether Aurka-mediated CD22 suppression was dependent on Bhlhe41, we analyzed *B41*^HET^*Aurka*^*fl/fl*^*R26*^*B41-EYFP/+*^ microglia. More than 97% of EYFP^+^ microglia from *B41*^HET^*Aurka*^*fl/fl*^*R26*^*B41-EYFP/+*^ mice corroborated the fact that *B41*^HET^*Aurka*^*fl/fl*^ microglia have undergone efficient DNA recombination (Fig. 6e). As expected, ectopic Bhlhe41 expression reversed CD22 upregulation on *Aurka*-deficient microglia (Fig. 6e, f), suggesting that Aurka suppresses CD22 expression on microglia through Bhlhe41.

To further investigate whether ectopic Bhlhe41 expression restored microglial phagocytic function in *Aurka*-deficient mice, we performed in vivo phagocytosis analysis by stereotactic injection of purified myelin conjugated with Dil (Myelin-Dil) into the cortex of *B41*^HET^*Aurka*^*fl/fl*^ and *B41*^HET^*Aurka*^*fl/fl*^*R26*^*B41-EYFP/+*^ mice. After 48 hours post injection, the injection site of *B41*^HET^*Aurka*^*fl/fl*^*R26*^*B41-EYFP/+*^ mice displayed reduced accumulation of injected myelin (Dil^+^), suggesting that Aurka-mediated microglial phagocytosis is dependent on Bhlhe41 (Fig. 6g).

Altogether, these data demonstrate that Aurka maintains microglial morphology, phagocytic function and CD22 suppression through Bhlhe41.

### CD22 blockade restores CNS repair in *Bhlhe41*-deficient mice

**Fig. 7:**
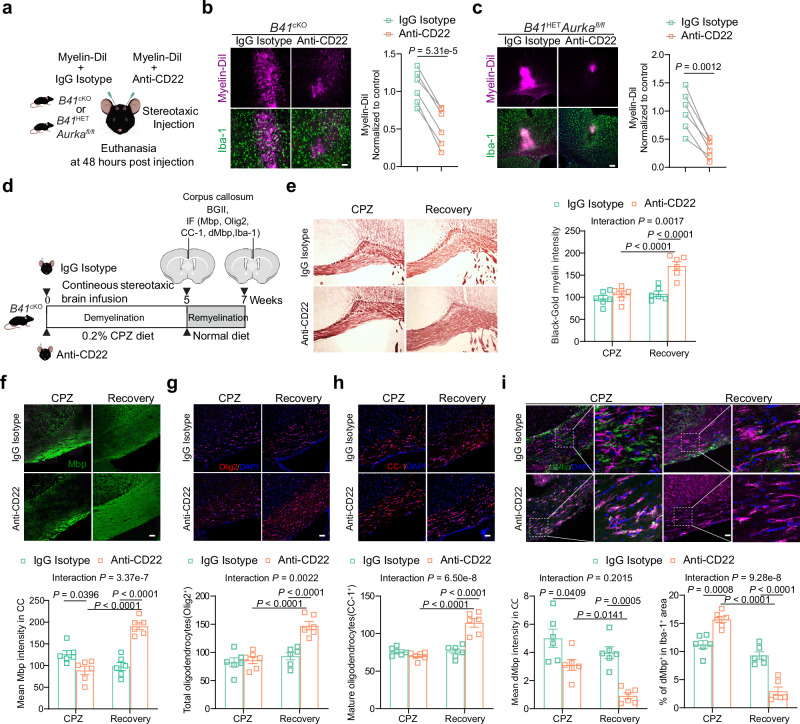
CD22 blockade rescues phagocytic function and remyelination in *Bhlhe41*-deficient mice. **a** Schematic representation of stereotaxic co-injection of 1 µL of Myelin-Dil (20 mg/mL) equally mixed with 1 µL of either an IgG isotype control (1 mg/mL) or anti-CD22 antibody (1 mg/mL) into opposite brain hemispheres of male *B41*^cKO^ or *B41*^HET^*Aurka*^*fl/fl*^ mice (3-months-old), respectively. **b**, **c** IF analysis of myelin clearance (Dil^+^) in the injection sites of **b** *B41*^cKO^ or **c** *B41*^HET^*Aurka*^*fl/fl*^ mice treated with either an IgG isotype control or anti-CD22 blocking antibody for 48 hours. *n* = 6 mice per group. **d** Schematic representation of long-term CD22 blockade in male *B41*^cKO^ mice in the CPZ-induced demyelination model. *B41*^cKO^ mice (3-months-old) underwent 7 weeks of continuous stereotaxic brain infusion of 2 µL of either an IgG isotype control (100 µg/mL) or anti-CD22 blocking antibody (100 µg/mL) every three days while being fed a 0.2% CPZ-enriched diet for 5 weeks, or followed by a CPZ-free normal diet for an additional 2 weeks. The CC of IgG isotype control- and anti-CD22-treated *B41*^cKO^ mice (*n* = 6 mice per group) from CPZ and Recovery groups were collected for the analyses for **e** BGII staining, and IF analysis of **f** Mbp, **g** pan-oligodendrocyte lineage cells (Olig2^+^) and **h** mature oligodendrocytes (CC-1^+^), and **i**) colocalization of dMbp with Iba-1. Data are presented as mean ± SEM. Two-sided paired Student’s *t* test (**b**, **c**); two-way ANOVA with Bonferroni-corrected post hoc comparisons (**e**, **f**, **i**). Negative binomial GLM with Bonferroni-corrected post hoc comparisons (two-sided, **g**); Poisson GLM with Bonferroni-corrected post hoc comparisons (two-sided, **h**). Created in BioRender. Zhao, Y. (2026) https://BioRender.com/osy77p2. Myelin-Dil (**b**, **c**) shown in magenta, Iba-1 (AF594, **i**) shown in magenta (pseudocolor). Scale bar for IF images:50 μm. Source data are provided as a Source Data file.

To investigate whether phagocytic deficits in *Bhlhe41*- or *Aurka*- deficient microglia were ascribed to CD22 upregulation, we conducted in vivo phagocytosis by stereotaxic co-injection of Myelin-Dil with either an IgG isotype control or anti-CD22 blocking antibody into opposite brain hemispheres of the same *B41*^cKO^ or *B41*^HET^*Aurka*^*fl/fl*^ mice, respectively (Fig. 7a). After 48 hours post injection, CD22 blockade robustly reduced the accumulation of injected myelin (Dil^+^) in *B41*^cKO^ or *B41*^HET^*Aurka*^*fl/fl*^ mice (Fig. 7b, c), suggesting that phagocytic deficits in *Bhlhe41*- or *Aurka*-deficient microglia are dependent on CD22 upregulation.

To further assess whether long-term CD22 inhibition restored remyelination in *Bhlhe41*-deficient mice, we stereotaxically infused either an IgG isotype control or anti-CD22 blocking antibody every three days into the brain of *B41*^cKO^ mice, which were meanwhile fed CPZ-enriched diet for 5 weeks, or followed by CPZ-free normal diet for an additional 2 weeks (Fig. 7d). During demyelination, BGII staining showed comparable overall demyelination between IgG- and anti-CD22-treated mice, although Mbp signal was slightly reduced, indicating that anti-CD22 did not significantly affect myelin loss (Fig. 7e, f). In contrast, during the recovery phase, anti-CD22 treatment markedly enhanced remyelination, as evidenced by increased BGII and Mbp staining and elevated numbers of mature oligodendrocytes (CC-1^+^), compared to IgG-treated controls (Fig. 7e–h). Colocalization analyses further revealed that anti-CD22 treatment enhanced microglia-mediated clearance of myelin debris (Fig.7i). Collectively, these results indicate that long-term CD22 blockade facilitates microglia-mediated clearance of myelin debris and promotes remyelination in Bhlhe41-deficient mice.

Taken together, CD22 blockade restores phagocytic function in *Bhlhe41*- and *Aurka*-deficient microglia and remyelination in *Bhlhe41*-deficient mice.

### Elevated *CD22* in human *PCDH9*^high^ microglia with low *BHLHE41*

**Fig. 8:**
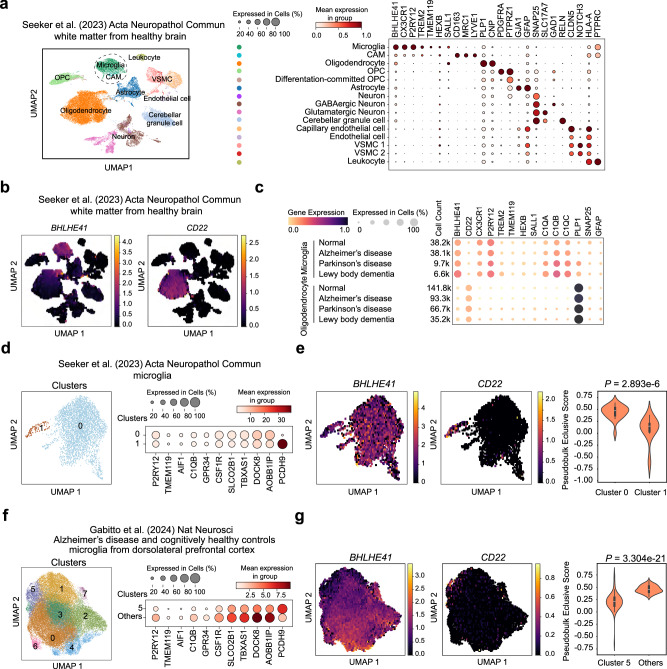
*CD22* upregulation in human *PCDH9*^high^ microglia subsets with *BHLHE41* downregulation. **a**, **b** Analysis of the expression pattern of *BHLHE41* and *CD22* in human CNS cells using single-cell RNA sequencing (scRNA-seq) data from healthy white matter. **a** The UMAP plot shows clusters of various cell types. The dot plot depicts the expression of *BHLHE41* and marker genes across various cell types. CAM, CNS-associated macrophages; OPC, oligodendrocytes progenitor cells; VSMC, Vascular smooth muscle cells. **b** The UMAP plot shows the expression pattern of *BHLHE41* and *CD22* in human CNS cells. **c** The dot plot showing the expression of *BHLHE41* and *CD22* in microglia and oligodendrocytes using scRNA-seq data from patients with neurodegenerative diseases and cognitively healthy controls. **d**–**g** human microglia subclusters in scRNA-seq data from **d**, **e** healthy white matter (*n* = 20 donors), and **f**, **g** the prefrontal cortex of patients with Alzheimer’s disease and cognitively healthy controls (*n* = 83 donors). **d**, **f** UMAP plots depict microglial subclusters, and dot plots illustrate the expression of microglial marker genes and *PCDH9* across microglial subsets. **e**, **g** UMAP plots visualize the expression pattern of *BHLHE41* and *CD22* in human microglial subclusters (left panel). Violin plots illustrate donor-level *BHLHE41-CD22* Tendency Scores computed using a pseudobulk approach (mean score per donor × cluster) (right panel). Positive values indicate bias toward *BHLHE41*, negative toward *CD22*, and values near zero indicate balanced expression. Two-sided Wilcoxon rank-sum test was used to compare the Tendency Score between two clusters (**e**, **g**).

To extend our studies from mouse to human microglia, we analyzed whether *BHLHE41* is specific to microglia in human CNS using public single-cell RNA sequencing (scRNA-seq) data from healthy white matter^43^. *BHLHE41* is highly expressed in human microglia, with lower expression levels observed in certain oligodendrocytes, neurons, and astrocytes (Fig. 8a, b). Additionally, co-expression of *BHLHE41* with microglia-specific genes (*P2RY12* and *TMEM119*) was observed in a small fraction of CAM (Fig. 8a). Furthermore, analysis of two independent scRNA-seq datasets from human fetal brains revealed high *BHLHE41* expression in fetal microglia (Supplementary Fig. 12)^44,45^. These findings suggest that *BHLHE41* is highly expressed in human CNS microglia starting early in development.

Nevertheless, analysis of public scRNA-seq data from either healthy white matter^43^ or brains of patients with AD^46^, PD, and LBD^47^ showed that *CD22* is barely expressed in human microglia (Fig. 7b, c). Intriguingly, oligodendrocytes from both cognitively healthy subjects and patients with neurodegenerative diseases, which weakly express *BHLHE41*, exhibit high *CD22* expression (Fig. 7b, c). These data suggest an inverse expression pattern of *CD22* and *BHLHE41* in human microglia and oligodendrocytes.

To further refine the analysis of *BHLHE41* and *CD22* expression in microglia, we performed sub-cluster analysis of 3690 microglia from healthy white matter^43^. A *PCDH9*^high^ microglia sub-population, which express multiple microglia marker genes *APBB1IP*^48^, *TBXAS1*^49^, *SLCO2B1*^50^, *DOCK8*^51^, *CSF1R* and *P2RY12*^52^, exhibits decreased *BHLHE41* expression but upregulated *CD22* expression (Fig. 8d, e, Supplementary Fig. 13a). Given that oligodendrocytes also highly express *PCDH9* and *CD22* (Supplementary Fig. 13a), we further validated this finding using independent scRNA-seq data from the dorsolateral prefrontal cortex of patients with AD and cognitively healthy subjects, which covers 40754 microglia (Supplementary Fig. 13b, c). Consistently, the sub-cluster analysis identified *PCDH9*^high^ microglia sub-population, in which *BHLHE41* is downregulated while *CD22* is upregulated, independent of health condition (Fig. 8f, g, Supplementary Fig. 13d).

Taken together, *BHLHE41* is downregulated in human *PCDH9*^high^ microglia subsets with *CD22* upregulation.

## Discussion

Here, we identified Bhlhe41, as a microglia-enriched transcription repressor in the mouse CNS, subject to negative autoregulation and was inhibited by *Aurka* deficiency. Aurka-Bhlhe41 axis in young mice maintained microglial function and responses to remyelination through inhibiting premature and progressive CD22 upregulation. Furthermore, we showed that a conserved expression pattern of *CD22* upregulation with *BHLHE41* downregulation in human *PCDH9*^high^ microglia subsets.

With aging, microglial dysfunction is ascribed to CNS remyelination failure and neurodegeneration^2,7^. We identified that *Aurka* or *Bhlhe41*-deficient young microglia displayed aging-like phenotypes, including microglial dystrophy and phagocytic deficits, and progressive CD22 upregulation, the latter of which is restricted to aging microglia^16^. Thus, the significance of this study lies in the identification of Aurka-Bhlhe41 as a safeguard that counteracts premature aging-like microglial dysfunction and remyelination impairment. Furthermore, we demonstrated that ectopic Bhlhe41 expression led to hypertrophic morphology and the expression of the activation marker CD68. These findings suggest that Bhlhe41 functions as a negative feedback mechanism, limiting excessive microglial phagocytosis while maintaining the phagocytic function.

The conserved high expression pattern of BHLHE41 in human and mouse microglia across different developmental stages suggests its potential role in regulating human microglial phagocytosis through suppressing CD22. We and others identified that *CD22*, inhibited by Bhlhe41 in mouse microglia, is almost specifically expressed by oligodendrocytes but not microglia in human CNS^53^. This discrepancy supports the notion of divergent microglial phenotypes across species. Nevertheless, this discrepancy may be explained by a more pronounced CD22 suppression by BHLHE41 in human microglia. Despite this, we observed *CD22* upregulation in human *PCDH9*^high^ microglial sub-population with decreased expression of *BHLHE41*. A previous study reports that human *PCDH9*^high^ microglial sub-population might be associated with myelination, but its function remains unknown^54^. Further studies are warranted to expand this analysis to more neurological diseases and elucidate its function. It is noteworthy that distinguishing between microglia-derived surface CD22 and oligodendrocyte-derived soluble CD22 bound to human microglia is necessary^53^.

Mutations in BHLHE41, e.g P384R and Y362H are associated with shorter sleep length and decrease sleep length of transgenic mice expressing P384R-BHLHE41 or Y362H-BHLHE41^17,55,56^. Unlike microglia-enriched Bhlhe41 expression in the mouse CNS, BHLHE41 is also expressed in human oligodendrocytes and neurons, supporting that mutated BHLHE41 in non-microglia cells from CNS may contribute to shorter sleep duration^55,57–59^. Of note, transgenic mice expressing P384R-BHLHE41 exhibit longer wakefulness upon knockout of *Bhlhe41*, suggesting that *Bhlhe41*-deficient microglia promote longer wakefulness caused by P384R-BHLHE41. Recent findings identify an association of microglial phagocytosis with sleep and prolonged sleep duration by microglia depletion^60–65^. Hence, it is worth exploring the function of P384R-BHLHE41 in regulating microglial identity and its association with sleep duration. Additionally, whether mutations in BHLHE41 are associated with neurodegeneration and neurodegenerative diseases need to be further elucidated.

An intriguing and unexpected finding in this study is that the mitotic kinase Aurka maintains Bhlhe41expression in microglia. In addition to regulate mitosis during the phase of G2 to M transition, Aurka also acts as a non-mitotic regulator in controlling multiple cellular events such as neurite extension in post-mitotic neurons and primary cilium disassembly at G0/G1 phase^27,28,66,67^. Here, we have shown impaired mitosis in *Aurka*-deficient infant microglia, suggesting that Aurka regulates microglia development in a mitotic kinase-dependent manner. Nevertheless, the molecular mechanism by which Bhlhe41 is controlled by Aurka is still unknown. Given that Bhlhe41 expression remains unchanged in *Aurkb*-deficient microglia, we speculate that Aurka might regulate Bhlhe41 expression in a mitosis-independent manner.

Our findings define an Aurka–Bhlhe41 axis that is crucial for safeguarding young microglial function and promoting myelin repair, yet several limitations highlight important directions for future work. First, while our loss- and gain-of-function studies together with ChIP–qPCR strongly support that Bhlhe41 represses the activity of self- and CD22 promoters, direct mechanistic confirmation, such as promoter–luciferase reporter assays demonstrating transcriptional repression, remains to be established. Second, although CD22 expression in the mouse CNS is largely restricted to microglia, we cannot fully exclude potential contributions from other CD22-expressing cells, such as peripheral B cells, during CD22 blockade in the cuprizone-induced demyelination model. Our intracerebral CD22 blockade targets the local CNS compartment, but a microglia-specific CD22 deletion model will be required to definitively rule out systemic or off-target effects. Third, our work focused on the CPZ-induced demyelination model in young mice. However, whether Bhlhe41 regulates CNS remyelination under physiological aging conditions remains unknown and represents an important future direction. Finally, although we identified a subset of *PCDH9*⁺ human microglia expressing *CD22*, validating the presence and functional relevance of this population in human brain tissue or in neurodegenerative disease contexts will be essential for translating these findings to human biology.

In summary, these findings reveal that the Aurka-Bhlhe41 axis prevents premature aging-like microglial dysfunction and remyelination impairment by inhibiting progressive CD22 upregulation. These findings offer insights into potential therapeutic strategies to combat age-related neurodegeneration and CNS functional decline.

## Methods

### Mice

*Cx3cr1*^*Cre*^ mice (Stock ID: 025524, JAX) and *Cx3cr1*^*CreER*^ mice (Stock ID: 021160, JAX) were obtained from the Jackson Laboratory. *Bhlhe41*^*dTomato-Cre/+*^ (referred to as *B41*^HET^) and *Aurka*^*fl/fl*^ mice were generated as previously reported^21,29^. *Bhlhe41*^*fl/fl*^, *Aurkb*^*fl/fl*^, and *Rosa26*^*Bhlhe41-EYFP/+*^ (referred to as *R26*^*B41-EYFP/+*^) mice were generated by Cyagen Biosciences Inc. using CRISPR-Cas9 technology. To generate *Bhlhe41*^*fl/+*^ mice, a targeting vector with homologous arms and a 2.4 kb genomic region containing the entire *Bhlhe41* coding sequence flanked by *loxP* sites was constructed by PCR using BAC clone RP23-133A9 as the template. To generate *Aurkb*^*fl/+*^mice, a targeting vector with homologous arms and exons 2-6 flanked by *loxP* sites was constructed by PCR using BAC clone RP24-281K2 as the template. To generate *Bhlhe41* transgenic mice, *R26*^*B41-EYFP/+*^, a targeting vector with homologous arms and a *CAG* promoter-*loxP*-*PGK*-*Neo*-*6xSV40 pA*-*loxP*-Kozak-*Bhlhe41* CDS-*P2A*-*EYFP*-*WPRE*-*BGH pA* cassette was integrated into the intron 1 of *Rosa26* locus. Mice production was performed by co-injection of ribonucleoprotein complexes and the targeting vector into fertilized eggs. Primers for in-house generated mouse genotyping were designed in-house, synthesized by Sangon Biotech and listed in Supplementary Table 1. In-house generated mice in this study are available upon request.

*Bhlhe41*^*fl/fl*^ mice were crossed with *Cx3cr1*^*Cre*^ mice for generating microglia-specific *Bhlhe41* knockout mice *Cx3cr1*^*Cre/+*^*Bhlhe41*^*fl/fl*^ mice (referred to as *B41*^cKO^). *Aurka*^*fl/fl*^ mice were crossed with *B41*^HET^, *Cx3cr1*^*Cre*^, or *Cx3cr1*^*CreER*^ to generate microglia-specific *Aurka* knockout mice, *B41*^HET^*Aurka*^*fl/fl*^, *Cx3cr1*^*Cre/+*^*Aurka*^*fl/fl*^ and *Cx3cr1*^*CreER/+*^*Aurka*^*fl/fl*^. *R26*^*B41-EYFP/+*^ mice were crossed with *B41*^HET^, or *B41*^HET^*Aurka*^*fl/+*^ mice to generate *B41*^HET^*R26*^*B41-EYFP/+*^ and *B41*^HET^*Aurka*^*fl/fl*^*R26*^*B41-EYFP/+*^mice. *Aurkb*^*fl/fl*^ mice were crossed with *B41*^HET^ mice to generate microglia-specific *Aurkb* knockout mice *B41*^HET^*Aurkb*^*fl/fl*^. To avoid germline recombination, only female *Bhlhe41*^*dTomato-Cre*^ mice or female offspring carrying the Cre allele were used for breeding with floxed mice. Wildtype (WT), *Cx3cr1*^*Cre/+*^, *B41*^HET^, *Cx3cr1*^*Cre/+*^*Bhlhe41*^*fl/+*^ (referred to as *B41*^cHET^), *B41*^HET^*Aurka*^*fl/+*^, *Cx3cr1*^*Cre/+*^*Aurka*^*fl/+*^, *Cx3cr1*^*CreER/+*^*Aurka*^*fl/+*^, *or B41*^HET^*Aurkb*^*fl/+*^ littermates were used as control mice. *Cx3cr1*^*CreER/+*^*Aurka*^*fl/fl*^ mice were injected with tamoxifen (TAM, 75 mg/kg) for five consecutive days at 3 months and 6 months of age, respectively.

All mice were maintained on the C57BL/6 genetic background and maintained in a specific pathogen-free (SPF) conditions in plastic cages (22 ± 2 °C; 50 ± 10% humidity; 12-h light/dark cycle) with ad libitum access to food and water. Sex-matched mice of different genotypes were used in all experiments, except for cuprizone-induced demyelination and in vivo phagocytosis assays, which were performed exclusively in male mice to minimize biological variability. Age of mice used in each experiment is specified in figure legends. All mouse experiments were performed in accordance with the guidelines and regulations of Xuzhou Medical University and were approved by the Animal Experimental Ethics Committee of Xuzhou Medical University (Approval no: 202211S011).

### Cuprizone-induced demyelination model

For the cuprizone (CPZ)-induced demyelination model, male WT and *B41*^cKO^ littermates, or *Cx3cr1*^*Cre/+*^*Aurka*^*+/+*^ and *Cx3cr1*^*Cre/+*^*Aurka*^*fl/fl*^ littermates, were fed regular chow mixed with 0.2% CPZ (Sigma, Cat: 14690) for 5 weeks to induce demyelination (denoted as the CPZ group), or followed by a CPZ-free normal diet for an additional 2 weeks to induce remyelination (denoted as the Recovery group). Mice fed a CPZ-free normal diet were sacrificed as the control group and sacrificed at the end of the experiment. The corpus callosum (CC), identified as the white matter tract between the lateral ventricles in coronal brain sections, was analyzed for myelination by immunofluorescence analysis of intact (Mbp^+^) and degraded (dMbp^+^) myelin, Black Gold II (BGII) staining of intact myelin, and electron microscopy assessment of myelinated and unmyelinated axons. For Mbp and BGII staining, myelin density was quantified as the total staining intensity within the CC. Myelin debris was defined as the dMbp^+^ immunostained area above background threshold and expressed as a percentage of the total CC area. Microglial engulfment of myelin debris was assessed by quantifying the area of dMbp^+^ signal colocalized within Iba-1^+^ microglia. Three sections containing the CC per mouse were analyzed. For immunofluorescence quantifications, two non-overlapping fields of view (FOV) were acquired per section at identical settings. Values from multiple FOV were averaged to generate a single value per mouse, which was used for statistical analysis. All image acquisition and quantifications were performed in a blinded manner.

### In vitro phagocytosis assay

For in vitro phagocytosis assays, microglia were enriched from the brain of WT and *B41*^cKO^ mice by 40% Percoll. Enriched microglia were seeded in duplicated 96-well plates (1 × 10^5^ cells per well) and in vitro cultured in RPMI 1640 complete medium containing 10% FBS and 200 μg/mL *E.coli* Bioparticles conjugated with pHrodo Green (ThermoFisher Scientific, Cat: P35366). One plate was cultured at 37 °C and the other plate at 4 °C as a negative control. After one hour, enriched microglia were washed with pre-cold phosphate buffer solution (PBS) and stained with antibodies for microglial surface markers. Microglial phagocytosis was assessed by flow cytometric analysis of pHrodo Green^+^ microglia by pre-gating live microglia (CD45^lo^CD11b^+^Cx3cr1^+^).

### In vivo phagocytosis assays

In vivo phagocytosis assays were performed by stereotaxic injection of phagocytic target material, Zymosan A conjugated with Alexa fluor 488 (Zymosan-AF488, ThermoFisher Scientific, Cat: Z23373) or myelin conjugated with Dil (Myelin-Dil) into the indicated mice using a stereotaxic apparatus (RWD Life Science, Cat: 68507).

For the preparation of Myelin-Dil, CNS myelin was isolated as previously described^68,69^. Briefly, crude brain homogenates from WT mice underwent sucrose density gradient ultracentrifugation and hypoosmotic shocks in hypotonic buffers and low-speed centrifugation to remove myelin sheaths from axons, followed by repeated sucrose density gradient ultracentrifugation. Myelin was quantified by the BCA Protein Assay Kit (Beyotime Biotechnology, Cat: P0012). Next, purified myelin pellets were conjugated with Dil by incubation of 10 mg of myelin with 5 µg/mL Dil (Yuanye, Cat: S25170) in PBS for 10 min at room temperature, followed by washing with PBS three times and centrifugation to remove free dye.

For the in vivo analysis of microglial phagocytosis in *Bhlhe41-* or *Aurka*-deficient mice, 2 µL of Zymosan-AF488 (2 mg/mL) was stereotactically injected into male WT and *B41*^cKO^ littermates, or male *Cx3cr1*^*Cre/+*^*Aurka*^*+/+*^ and *Cx3cr1*^*Cre/+*^*Aurka*^*fl/fl*^ littermates. For the in vivo phagocytosis analysis of *Aurka*-deficient microglia with Bhlhe41 overexpression, 1 µL of Myelin-Dil was stereotactically injected into male *B41*^HET^*Aurka*^*fl/fl*^ and *B41*^HET^*Aurka*^*fl/fl*^*R26*^*B41-EYFP/+*^mice. For in vivo phagocytosis assays by CD22 blockade, 1 µL of Myelin-Dil (20 mg/mL) pre-mixed with 1 µL either an IgG isotype control (1 mg/mL) or anti-CD22 antibody (1 mg/mL) were injected into opposite brain hemispheres of male *B41*^cKO^ or *B41*^HET^*Aurka*^*fl/fl*^ mice, respectively.

Briefly, mice pre-anesthetized with pentobarbital sodium (90 mg/kg body weight) were positioned on a stereotactic apparatus. Subsequently, the skulls were exposed with a hole drilled at the injection site using aseptic technique. The phagocytic target solution, or the antibody–phagocytic target solution was injected into the cerebral cortex of the mice through a micro-syringe pump (Quintessential Injector, Stoelting, Cat: 53311) at ±2 mm lateral, 0 mm anterior–posterior, and −1.5 mm deep relative to the bregma at a rate of 200 nL/min. The needle was slowly withdrawn after remaining in place for an additional 10 min to allow for diffusion. For flow cytometric analysis of phagocytic microglia, mice were sacrificed and transcardially perfused with PBS after 5 hours, followed by flow cytometric analysis of 40% Percoll-enriched microglia from the entire brain. For IF analysis of the clearance of phagocytic target material, mice were euthanized after 48 hours and were transcardially perfused with 4% paraformaldehyde (PFA). The entire injection site was sectioned at 40 μm in thickness using vibrating microtome (Leica, Cat: VT100S). The entire injection site was sectioned using vibrating microtome for IF analysis. Six to eight consecutive sections, separated by 40 µm, were then quantified to determine the overall target clearance over the injection site. All quantitative analyses were performed in a double-blind analysis, during which sections without significant Iba-1^+^ microgliosis at the injection site margin were excluded. Each dot represents an average fluorescence intensity of at least six consecutive slides from one mouse.

### In vivo BrdU assay analysis

Infant WT, *B41*^HET^*Aurka*^*fl/+*^ and *B41*^HET^*Aurka*^*fl/fl*^ mice (P10) were intraperitoneally injected with 100 µL of BrdU solution (10 mg/mL, Bestbio, Cat: BB-4261) once every 12 h for 3 consecutive days. Eight hours post last injection, mice were sacrificed for the analysis of microglial proliferation.

### Immunofluorescence analysis

Mice pre-anesthetized with pentobarbital sodium were perfused with 4% PFA. Brains were dissected out and post-fixed in 4% PFA overnight, followed by treatment with 30% sucrose for 48 hours. Next, brains were sectioned at 20 μm in thickness using freezing microtome (Leica, Cat: CM1950). Sections were blocked with 10% goat serum, 1% bovine serum albumin (BSA), 0.3% Triton-X in PBS for 1 hour at room temperature before applying the primary antibody mix diluted in blocking buffer overnight at 4 °C. To assay Bhlhe41 expression in CNS, sections were labeled with mouse Anti-NeuN (1:100, Millipore, Cat: MAB377), mouse anti-Gfap (1:200, Biolegend, Cat: 644702) and rabbit anti-Olig2 (1:500, Proteintech, Cat: 13999-1-AP). To examine microglial morphology and numbers, sections were stained with rabbit anti-Iba-1 (1:500, Wako, Cat: 019-19741). To further characterize microglia, sections were labeled with goat anti-dTomato (1:100, Biorbyt, Cat: orb182397) or rabbit anti-RFP (1:200, Rockland, Cat: 600-401-379S), rat anti-CD206 (1:200, Bio-Rad, Cat: MCA2235), rat anti-BrdU (1:400, Abcam, Cat: ab6326), mouse anti-phospho-Histone H3(Ser10) (1:200, Cell Signaling Technology, Cat: 9706S), rat anti-CD68 (1:500, Abcam, Cat: ab53444,) and mouse anti-CD22 (1:500, Abcam, Cat: ab181771). For the analysis of brains from mice following cuprizone-induced demyelination, sections were stained with rat anti-Mbp (1:1000, Abcam, Cat: ab7349), mouse anti-APC (1:100, CC-1, Merck, Cat: OP80), rabbit anti-degraded myelin basic protein antibody (1:2000, Merck, Cat: AB5864) and rabbit anti-Olig2 (1:500, Proteintech, Cat: 13999-1-AP). Sections were stained overnight and then washed five times at room temperature. Next, sections were stained with matched AF488 (1:500, ThermoFisher Scientific, Cat: A-21206/A-11006), AF555 (1:500, ThermoFisher Scientific, Cat: A-31572/A-21422), AF647 (1:500, ThermoFisher Scientific, Cat: A-32733)-conjugated secondary antibody for 2 hours at room temperature. TUNEL staining was performed according to manufacturer’s instructions (Roche, Cat: 12156792910) after incubation with secondary antibodies. Following washing five times, sections were mounted with DAPI mounting medium (Abcam, Cat: ab104139) and fluorescent images were acquired using a confocal laser-scanning microscope (Zeiss LSM710 and Leica STELLARIS 5) and processed with ZEISS ZEN (v3.9) and Leica Application Suite X (v4.6.1), respectively.

### Long-term CD22 blockade assay

Mice pre-anesthetized with pentobarbital sodium were positioned in a stereotaxic device. To continuously inject antibodies into the lateral ventricle, a guide cannula (RWD Life Science, Cat: 62003) was implanted at +1 mm lateral, −0.3 mm anterior–posterior, and −3 mm deep relative to bregma. The guide cannula and anchoring screws were fixed with dental cement and capped by a threaded plastic cap (RWD Life Science, Cat: 62102) to prevent clogging. After surgery, mice received antibiotic treatment (Penicillin V potassium, 200 U/g, Solarbio, Cat: p7480) and were allocated a recovery period of one week. Then the mice were given 2 µL of anti-CD22 antibody (100 µg/mL, BioXCell, Cat: BE0011) or IgG isotype control antibody (100 µg/mL, BioXCell, Cat: BE0083) every 3 days for 7 weeks, using an injector cannula (RWD Life Science, Cat: 62203) connected to the guide cannula, while being fed a 0.2% CPZ-enriched diet for 5 weeks followed by a CPZ-free normal diet for an additional 2 weeks.

### Transmission electron microscopy

Mice from CPZ-induced demyelination model were deeply anesthetized using sodium pentobarbital and subsequently transcardially perfused with PBS to remove blood, followed by perfusion with EM fixative (Cat: G1102-100 mL, Servicebio). The central region of the CC was dissected (1 mm in thickness), promptly fixed in EM fixative and washed with PBS. Post-fixation was conducted using 1% osmium tetroxide for two hours. The tissue was subsequently rinsed with double-distilled water and dehydrated through a graded series of ethanol and acetone. The samples were then infiltrated with varying concentrations of epoxy resin and polymerized sequentially at 37 °C for 24 hours, 45 °C for 24 hours, and finally at 60°C for 24 hours. Ultrathin sections with a thickness of 80 nm were prepared and treated with uranyl acetate and lead citrate before being examined using a transmission electron microscope (TEM, Hitachi HT7800) and processed with the Hitachi TEM system. TEM images of the CC were used to identify myelinated axons, defined by the presence of intact multilamellar myelin sheaths, and unmyelinated axons, including axons with unstructured myelin exhibiting lamellar splitting, disintegration or vacuolar formation^70^. For each mouse, three non-overlapping FOV per section were analyzed at identical magnification. The numbers of myelinated and unmyelinated axons were manually counted, averaged per mouse and used to assess demyelination and remyelination.

### Flow cytometric analysis

Prior to flow cytometric analysis, cell suspensions were made from yolk sac, brain, spinal cord, bronchoalveolar lavage fluid (BALF) and peritoneal cavity. Briefly, whole brains from embryos, newborn and adult mice were minced and mechanically dissociated by gentle homogenization, followed by suspension in 40% Percoll and centrifugation at 800 × *g* for 30 minutes at room temperature. Yolk sac from embryos were minced and incubated with 0.5 mg/mL Collagenase IV (ThermoFisher Scientific, Cat: 17104019) in RPMI 1640 medium with 1% FBS for one hour. BALF were collected by inserting a canula into the trachea following opening the chest cavity. 1 mL of pre-chilled PBS with 2 mM EDTA was used to flush the lung three times. Cell pellets were collected and washed using PBS with 1% FBS and 2 mM EDTA and filtered through 70 μm mesh prior to labeling for flow cytometric analysis. Data were acquired on BD FACSAria III using BD FACS DIVA software (v9).

For the surface staining, Anti-CD16/CD32 antibody was used as Fc blocker, while Zombie Aqua™ Fixable Viability Dye (FVD, Biolegend, Cat: 423102) was used to label dead cells. The following markers were used for cell identification: yolk sac myeloid cells (FVD^-^CD45^+^CD11b^+^Cx3cr1^+^), microglia (FVD^−^^-^CD45^lo^Cx3cr1^+^CD11b^+^), alveolar macrophage (FVD^-^SSC^hi^CD45^+^CD11b^lo^CD11c^+^CD24^−^) and peritoneal B-1 cells (FVD^-^CD45^+^CD19^+^B220^lo^). For the analysis of CD22 expression, microglia from the brains of young mice (2–4 months) and middle-aged mice (10–14 months) of different genotypes were enriched by 40% Percoll and labeled with antibodies for microglia markers and APC anti-mouse CD22 Antibody (clone OX-97, BioLegend, Cat: 126110), or matching APC Rat IgG1, κ Isotype Ctrl Antibody (BioLegend, Cat: 400411). For the validation of CD22 expression, InVivoMab anti-mouse CD22 Antibody (clone Cy34.1, BioXcell, Cat: BE0011) were in-house labeled with PacBlue succinimidyl ester (AAT, Cat: 570) according to the instructions by the manufacturers. Excess PacBlue dye was removed using Bio-Spin P-6 gel desalting column (Bio-Rad, Cat: 7326227). Flow cytometric data were analyzed using Flowjo (v10.8.1). For visualization in histogram overlays, fluorescence intensity data were normalized to the mode of each distribution to standardize peak heights, thereby enabling direct visual comparison of expression distribution shapes across samples independent of cell count.

### Western blot

For the analysis of *Bhlhe41* and *Aurka* expression, microglia were purified by flow cytometric sorting of FVD^-^CD45^lo^Cx3cr1^+^CD11b^+^ cells on FACSAria III (BD Biosciences). For the validation of *R26*^*B41-EYFP*^ mice, primary microglia from adult WT, *B41*^KO^ and *B41*^HET^*R26*^*B41-EYFP/+*^ mice were purified by flow cytometric sorting. Purified cells were lysed with RIPA lysis buffer with Protease and phosphatase inhibitor cocktail (Beyotime Biotechnology, Cat: P1045). The supernatant was collected after centrifugation at 12,000 × *g* for 5 minutes. The protein concentration of the supernatant was assayed using BCA protein assay kit (Beyotime Biotechnology, Cat: P0012S). Equal amounts of protein from each group were mixed with 5× loading buffer and heated at 95 °C for 5 minutes prior to loading onto the SDS-PAGE gel. The separated proteins were then transferred to a nitrocellulose membrane, which was blocked with 5% BSA at room temperature for 2 hours and stained with rabbit anti-Bhlhe41 polyclonal Antibody (1:1000, Proteintech, Cat: 12688-1-AP), anti-β-actin (1:50000, Proteintech, Cat: 66009-1), rabbit anti-Aurka polyclonal Antibody (1:1000, Novus, Cat: NBP-1-51843) and Anti-GAPDH (1:5000, Sangon Biotech, Cat: D190090) at 4 °C for overnight. The membranes were washed three times (5 min each) by Tris-buffered saline with Tween 20 (TBST) and stained with Goat Anti-Mouse/Rabbit lgG Dylight 800 (1:10000, Abbkin, Cat: A23910/A23920) at room temperature for 2 hours. The membranes were scanned using a Western blot imaging system (Odyssey CLXL1-CDR). Image acquisition was performed using Image Studio (V5.5). Band intensities were quantified using ImageJ (v1.54).

### Histological analysis

BGII myelin staining was used to assess myelination in the corpus callosum of mice subjected to the CPZ-induced demyelination model. Briefly, coronal brain sections (20 μm) were cut on a Leica CM1950 cryostat, mounted on gelatin-coated slides and oven-dried at 60 °C for 30 minutes. Sections were rehydrated in deionized water (2 minutes), stained in pre-heated BGII solution (Biosensis, Cat. BSS-TR-100-BG) at 60 °C for 12 minutes, rinsed in deionized water (3 × 5 minutes) and immersed in stop solution (2 minutes). Samples were dehydrated in graded ethanol (70%, 95% and 100%, each for 3 minutes), cleared in xylene (3 minutes) and coverslipped with neutral balsam mounting medium. Myelinated fibers were imaged by brightfield microscopy. Quantification of myelinated fibers was performed by measuring optical density in the CC using ImageJ.

### GEM analysis

Microglia were purified by flow cytometric sorting of FVD^-^CD45^lo^Cx3cr1^+^CD11b^+^ cells from 40% percoll-enriched cells from the brains of young *B41*^HET^ and *B41*^KO^ mice (10−15 mice per sample). Total RNA was extracted using a miRneasy Mini Kit (Qiagen, Cat: 217084) and quantified with NanoDrop 2000 Spectrophotometer (ThermoFisher Scientific). The cRNA was prepared using a LowInput QuickAmp Labeling Kit (Agilent, Cat: 5190-2305). GEM analysis was performed using SurePrint G3 Mouse Gene Expression v2 8x60K Microarray (Agilent, Cat: G4852B). The GEM data was quantile normalized, and the differential expression analysis was performed using R package *Limma* (v3.54.2)^71^. In-house generated GEM data was compared with public bulk RNA sequencing data from *Bhlhe40*/*Bhlhe41* double knockout microglia. Shared differentially expressed genes were defined as genes with an absolute fold-change (logFC) ≥ 0.5 and an adjusted *P* value ≤ 0.05.

### ChIP coupled with qPCR (ChIP-qPCR)

Putative BHLHE41-binding sites in the promoters of human and mouse *BHLHE41* and *CD22*, which were retrieved from UCSC Genome Browser, were identified using JASPAR database. For the validation of putative Bhlhe41-binding sites, ChIP coupled with qPCR (ChIP-qPCR) analysis was performed with microglia enriched by 40% Percoll from WT mice using a ChIP Assay Kit (Beyotime, Cat: P2078). Briefly, enriched microglia were fixed with 1% PFA for 10 min before quenching with 1× glycine solution for 5 min. The nuclei were isolated using 1× SDS lysis buffer with 1× PMSF protease inhibitor and sonicated on ultrasonic homogenizer to result in chromatin fragments ranging from 200 bp to 800 bp. 1% of total chromatin fragments were preserved as Input DNA for qPCR analysis. chromatin fragments were blocked with salmon sperm DNA and immunoprecipitated using 2 μg of rabbit anti-Bhlhe41 polyclonal antibody (Proteintech, Cat: 12688-1-AP) or rabbit IgG (Sangon Biotech, Cat: D110502) overnight at 4 °C. Protein-DNA complexes bound to Protein A/G agarose were washed, and the crude DNA was used for qPCR analysis. Enrichment of the putative Bhlhe41-binding sites in the promoters of *Bhlhe41* and *CD22*, as well as negative control regions was analyzed by qPCR analysis using Taq Pro Universal SYBR qPCR Master Mix (Vazyme, Cat: Q712) on a LightCycler 480 (Roche). The specific qPCR primers were designed in-house, synthesized by Sangon Biotech and are shown in Supplementary Table 2. All IP signals were normalized relative to the signal from the input chromatin.

### scRNA-seq data analysis

For the analysis of *BHLHE41* expression in microglia and oligodendrocytes from human post-mortem white matter^43^, the dorsolateral prefrontal cortex from patients with AD^46^, the substantia nigra pars compacta from patients with PD and LBD^47^, and fetal cerebellum^44,45^, dot plots were generated using Cellxgene platform (https://cellxgene.cziscience.com/gene-expression)^72^. For the analysis of *BHLHE41* and marker genes of microglia and oligodendrocytes from patients with AD, UMAP plots were generated by Cellxgene (https://cellxgene.cziscience.com/e/100c6145-7b0e-4ba6-81c1-ffebed0d1ac4.cxg/)^46^.

The scRNA-seq data from human post-mortem white matter and the dorsolateral prefrontal cortex from patients with AD were retrieved from Cellxgene^43,46^.The public scRNA-seq data were preprocessed and normalized using the ln(CPTT + 1) transformation of raw counts, where CPTT is Counts Per Ten Thousand^72^. Cells with more than 10% mitochondrial genes and fewer than 200 detected genes per cell were removed. Genes expressed in fewer than three cells were excluded before identifying the top 2000 variable genes in the dataset.

For the analysis of scRNA-seq data from human post-mortem white matter from healthy brains (*n* = 20 donors)^43^, two rounds of clustering were performed. In the first round, all cell types from the white matter were included to study the distribution of genes of interest across different cell types. In the second round, microglia were subclustered with a resolution of 0.05. For the analysis of scRNA-seq data from microglia from patients with AD (*n* = 83 donors)^46^, non-microglia contaminants characterized by high expression of *LYVE1*, *MRC1*, and *CD163* (cluster 13), *SKAP1* and *ETS1* (cluster 15), and *FCN1*, *VCAN*, and *FGR* (cluster 16) were removed prior to sub-cluster analysis. Microglial sub-cluster analysis was performed with a resolution of 0.4. The mean expression of genes of interest was visualized using dot plots and UMAP plots. A *BHLHE41*-*CD22* Tendency Score was calculated for each cell as the log-transformed expression of *BHLHE41* minus that of *CD22*. Positive values indicated a bias toward *BHLHE41* expression, negative values toward *CD22*, and values near zero reflected balanced expression. Donor-level pseudobulk scores were obtained by averaging the tendency score across all cells within each donor × cluster combination. For comparisons between two clusters, donor-level scores were tested using a two-sided Wilcoxon rank-sum test. The scRNA-seq data analysis was performed using the Scanpy v1.11.5 package in Python (v3.12)^73^.

### Statistical analysis

Statistical analyses were performed using R (v4.2.2) with the following packages: *lme4* (v1.1-33), *lmerTest* (v3.1-3), *emmeans* (v1.11.1), *DHARMa* (v0.4.7), MASS (v7.3-60), *nlme* (v3.1-162), *rstatix* (v0.7.2) and *ARTool* (v0.11.2). Data are presented as mean ± SEM unless stated otherwise. Genotype, treatment/model phase, CNS region and axon status were treated as categorical variables. Prior to analysis, data distributions were assessed using Q-Q plots and/or Shapiro-Wilk test, and homogeneity of variance was evaluated using Levene’s test. When appropriate, outcome variables were log2-transformed to meet normality assumptions.

For bounded or non-normal continuous outcomes, non-parametric methods were applied. For two-group comparisons of continuous outcomes meeting parametric assumptions, Student’s *t* test or paired *t* test was used when variances were equal; Welch’s *t* test was applied if variances were unequal. When normality assumptions were violated or sample sizes were very small, Wilcoxon rank-sum tests were used. For comparisons involving more than two groups, one-way or two-way ANOVA was applied, including interaction terms when multiple factors were modeled. When assumptions of normality or homogeneity were violated, or for bounded continuous data, Kruskal-Wallis tests (non-factorial designs) or Aligned Rank Transform (ART) ANOVA (factorial designs) were applied.

Group differences in count outcomes were analyzed using generalized linear models (GLMs) with either a Poisson or negative binomial distribution, selected based on the presence of overdispersion. Model fit was assessed using Pearson residuals, and overdispersion was evaluated. For experiments with repeated measurements from individual animals, linear mixed-effects models (LMMs) were applied. Mouse identity was included as a random intercept to account for within-animal correlation, while genotype, treatment/model phase, axon status, and their interactions were treated as fixed effects. Model assumptions were evaluated using DHARMa residual diagnostics, including tests for goodness-of-fit (testUniformity) and dispersion (testDispersion). In the presence of heteroscedasticity, weighted mixed-effects models were fitted using the *nlme* package.

All post hoc pairwise comparisons were performed using estimated marginal means (EMMeans) with Bonferroni correction to adjust for multiple testing. All statistical tests were two-sided, and a *P* value or adjusted *P* value < 0.05 was considered statistically significant. Detailed statistical results, including exact *P* values, are provided in the figure legends and the Source Data file.

### Reporting summary

Further information on research design is available in the Nature Portfolio Reporting Summary linked to this article.

## Supplementary information

Supplementary Information

Description of Additional Supplementary Files

Supplementary Data 1

Reporting Summary

Transparent Peer Review file

## Source data

Source Data

## Data Availability

In-house-generated GEM data have been deposited in Gene Expression Omnibus (GEO) under accession code GSE276929.The public bulk RNA-seq data from Bhlhe40/Bhlhe41 knockout microglia, and from yolk sac myeloid progenitors and microglia across multiple developmental stages used in this study are available in GEO under accession code GSE254233^33^ and GSE79818^22^. The single-cell RNA sequencing (scRNA-seq) datasets used in this study are available in the Cellxgene platform, including scRNA-seq data from human post-mortem white matter from healthy brains [https://cellxgene.cziscience.com/collections/9d63fcf1-5ca0-4006-8d8f-872f3327dbe9]^43^, the substantia nigra pars compacta of patients with Parkinson’s disease (PD) [https://cellxgene.cziscience.com/collections/b0f0b447-ac37-45b0-b1bf-5c0b7d871120]^47^, the dorsolateral prefrontal cortex of patients with AD [https://cellxgene.cziscience.com/collections/1ca90a2d-2943-483d-b678-b809bf464c30]^46^, fetal cerebellum between gestation week 10-18 [https://cellxgene.cziscience.com/collections/c114c20f-1ef4-49a5-9c2e-d965787fb90c]^45^, and neocortex between gestation week 14-25 [https://cellxgene.cziscience.com/collections/c8565c6a-01a1-435b-a549-f11b452a83a8]^44^. Source Data are provided with this paper and have been deposited in Figshare [10.6084/m9.figshare.30896234]. Source data are provided with this paper.

## Code availability

The codes for the scRNA-seq data analysis and statistical analysis are available at https://github.com/YelinZhao-A/Microglia_BHLHE41. Additional information needed to reanalyze the data reported in this paper can be obtained from the lead contact upon request.
