# Supplementary material for: Aurka-Bhlhe41 axis prevents premature aging-like microglial dysfunction and promotes remyelination
Source: Nat Commun. 2026 Mar 27;17:5238. doi: 10.1038/s41467-026-71014-w (PMC13260908; doi:10.1038/s41467-026-71014-w)
Supplement: Supplementary file 1 — Supplementary Information [file 41467_2026_71014_MOESM1_ESM.pdf]

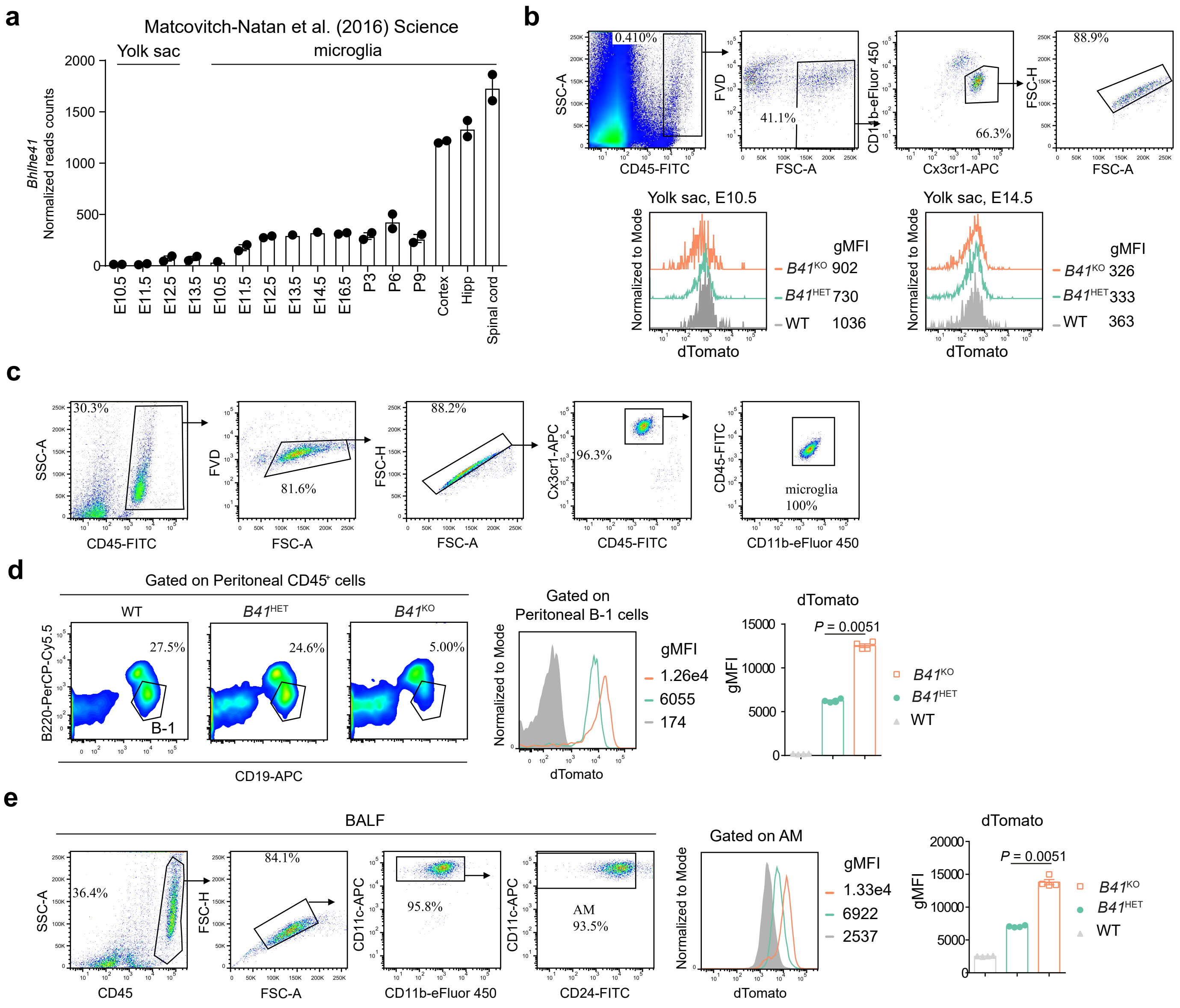

a
Matcovitch-Natan et al. (2016) Science
Yolk sac
microglia
Bhlhe41
Normalized reads counts
E10.5 E11.5 E12.5 E13.5 E10.5 E11.5 E12.5 E13.5 E14.5 E16.5 P3 P6 P9 Cortex Hipp Spinal cord
b
0.410%
41.1%
66.3%
88.9%
SSC-A
FVD
CD11b-eFluor 450
FSC-H
CD45-FITC
FSC-A
Cx3cr1-APC
Yolk sac, E10.5
Yolk sac, E14.5
Normalized to Mode
dTomato
gMFI
B41KO 902
B41HET 730
WT 1036
B41KO 326
B41HET 333
WT 363
c
30.3%
81.6%
88.2%
96.3%
microglia
100%
CD45-FITC
CD11b-eFluor 450
Cx3cr1-APC
d
Gated on Peritoneal CD45+ cells
WT
B41HET
B41KO
27.5%
24.6%
5.00%
B-1
B220-PerCP-Cy5.5
CD19-APC
Gated on Peritoneal B-1 cells
gMFI
1.26e4
6055
174
dTomato
P = 0.0051
gMFI
WT
e
BALF
36.4%
84.1%
95.8%
AM
93.5%
CD45
CD11c-APC
CD11b-eFluor 450
CD24-FITC
Gated on AM
1.33e4
6922
2537

Supplementary Figure 1. Bhlhe41 expression in mouse primitive myeloid progenitors, peritoneal B-1 cells and alveolar macrophages.

a) Analysis of *Bhlhe41* expression in yolk sac myeloid progenitors and microglia across embryonic, postnatal, and adult stages using public RNA sequencing data. Hipp, Hippocampus. E, embryonic. P, postnatal.

b) Flow cytometric analysis of Bhlhe41 expression (dTomato^{+}) in yolk sac myeloid progenitors from wildtype (WT), *Bhlhe41*$^{dTomato\text{-}Cre/+}$ (referred to as *B41*^{HET}) and *Bhlhe41*$^{dTomato\text{-}Cre/dTomato\text{-}Cre}$ (referred to as *B41*^{KO}) embryonic mice at E10.5 and E14.5. The upper panel shows the gating strategy for yolk sac myeloid progenitors.

c) The gating strategy for microglia in the mouse CNS, corresponding to **Fig. 1a**.

d-e) Flow cytometric analysis of Bhlhe41 expression (dTomato^{+}) in peritoneal B-1 cells **d**) and **e**) alveolar macrophages (AM) from WT, *B41*^{HET} and *B41*^{KO} mice (n = 4 mice per genotype) at 8 weeks of age. BALF, bronchoalveolar lavage fluid. Fluorescence intensity histograms are normalized to their respective modes. Kruskal-Wallis test with Bonferroni-corrected post hoc comparisons (**d**, **e**). Data are presented as mean ± SEM. Source data are provided as a Source Data file.

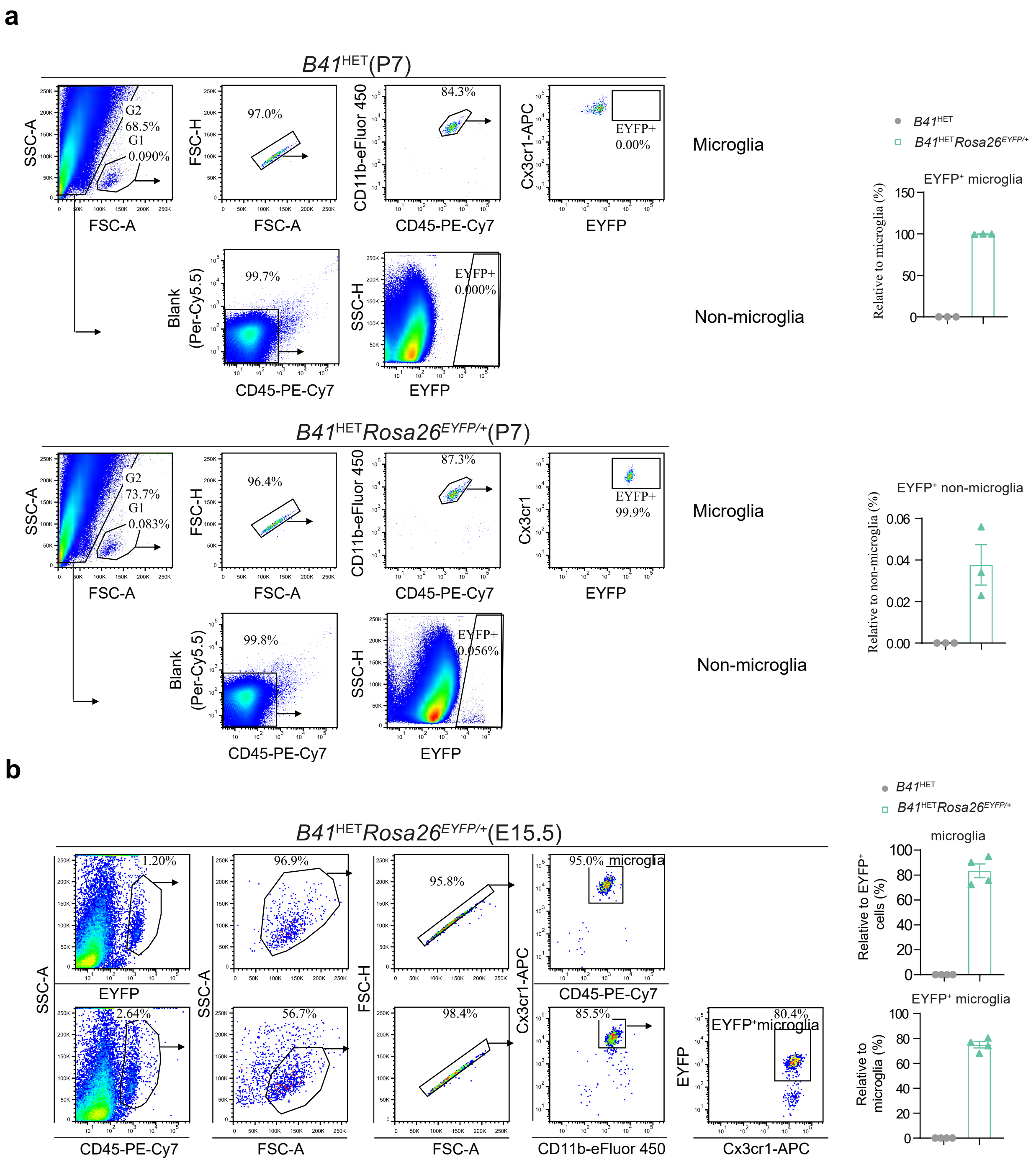
a
B41HET(P7)
G2
68.5%
G1
0.090%
SSC-A
FSC-A
97.0%
FSC-H
84.3%
CD11b-eFluor 450
CD45-PE-Cy7
Cx3cr1-APC
EYFP+
0.00%
EYFP
Microglia
99.7%
Blank (Per-Cy5.5)
SSC-H
EYFP+
0.000%
Non-microglia
B41HET
B41HETRosa26EYFP/+
EYFP+ microglia
Relative to microglia (%)
B41HETRosa26EYFP/+(P7)
G2
73.7%
G1
0.083%
96.4%
87.3%
Cx3cr1
EYFP+
99.9%
99.8%
EYFP+
0.056%
EYFP+ non-microglia
Relative to non-microglia (%)
b
B41HETRosa26EYFP/+(E15.5)
1.20%
96.9%
95.8%
95.0% microglia
2.64%
56.7%
98.4%
85.5%
EYFP+microglia
80.4%
CD11b-eFluor 450
Cx3cr1-APC
microglia
Relative to EYFP+ cells (%)
EYFP+ microglia
Relative to microglia (%)

Supplementary Figure 2. DNA recombination in the CNS of *B41*^{HET}*Rosa26*$^{EYFP/+}$ mice.

a) Flow cytometric analysis of EYFP^{+} cells in microglia and other CNS-resident cells (non-microglia) from *B41*^{HET} and *B41*^{HET}*Rosa26*$^{EYFP/+}$ mice (n = 3 mice per genotype) at P7.

b) Flow cytometric analysis of EYFP^{+} cells in 40% Percoll-enriched CNS cells from *B41*^{HET} (n = 3 embryo) and *B41*^{HET}*Rosa26*$^{EYFP/+}$ embryonic mice (n = 4 embryo) at E15.5. Data are presented as mean ± SEM. Data are shown for descriptive purposes only with no inferential statistics applied. Source data are provided as a Source Data file.

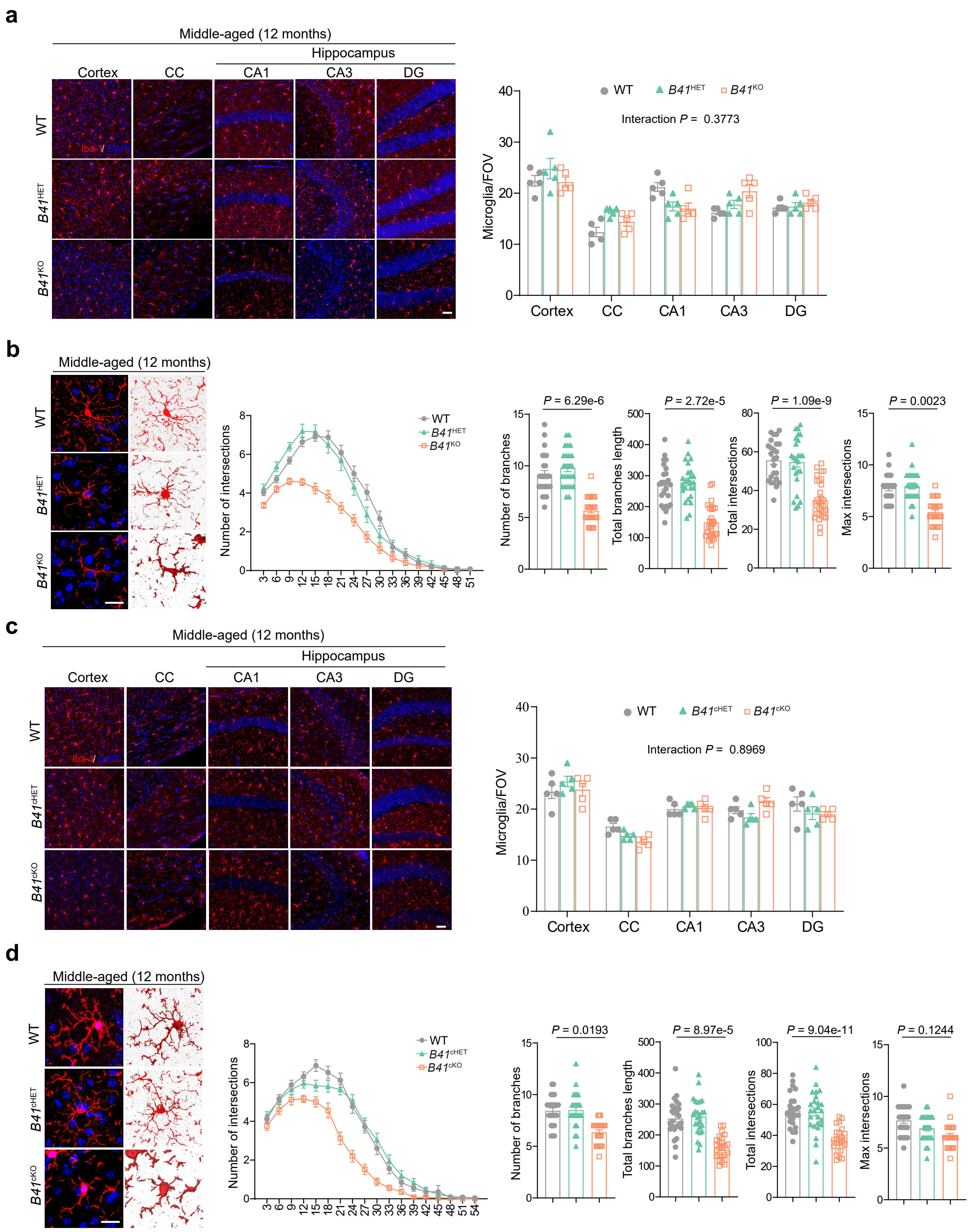

a
Middle-aged (12 months)
Hippocampus
Cortex
CC
CA1
CA3
DG
WT
B41HET
B41KO
Iba-1/ DAPI
WT
B41HET
B41KO
Interaction P = 0.3773
Microglia/FOV
b
Middle-aged (12 months)
Number of intersections
Number of branches
Total branches length
Total intersections
Max intersections
P = 6.29e-6
P = 2.72e-5
P = 1.09e-9
P = 0.0023
c
Middle-aged (12 months)
B41cHET
B41cKO
Interaction P = 0.8969
d
Middle-aged (12 months)
P = 0.0193
P = 8.97e-5
P = 9.04e-11
P = 0.1244

Supplementary Figure 3. Middle-aged *Bhlhe41*-deficient microglia exhibiting dystrophic-like morphology.

a) IF analysis of microglia (Iba-1^{+}) in the cerebral cortex, CC and hippocampus of middle-aged WT, *B41*^{HET} and *B41*^{KO} mice (n = 5 mice per genotype).

b) Sholl analysis of microglial processes and branch intersections in a total of 16 microglia from middle-aged WT, *B41*^{HET} and *B41*^{KO} mice (n = 5 mice per genotype). Five microglia per mouse were quantified.

c) IF analysis of microglia (Iba-1^{+}) in the cerebral cortex, CC and hippocampus of middle-aged WT, *B41*^{cHET} and *B41*^{cKO} mice (n = 5 mice per genotype).

d) Sholl analysis of microglial processes and branch intersections in middle-aged WT, *B41*^{cHET} and *B41*^{cKO} mice (n = 5 mice per genotype). Five microglia per mouse were quantified. Data are presented as mean ± SEM. Poisson generalized linear model (GLM) (two-sided, **a**, **c**); LMM for continuous data and negative binomial GLMM for count data (two-sided), with repeated measures from the same mouse accounted for as a random effect (**b**, **d**). All post hoc pairwise comparisons were adjusted using the Bonferroni correction (**b**, **d**). Scale bar for IF images in (**b**, **d**) is 10 μm, while the others are 50 μm. Source data are provided as a Source Data file.

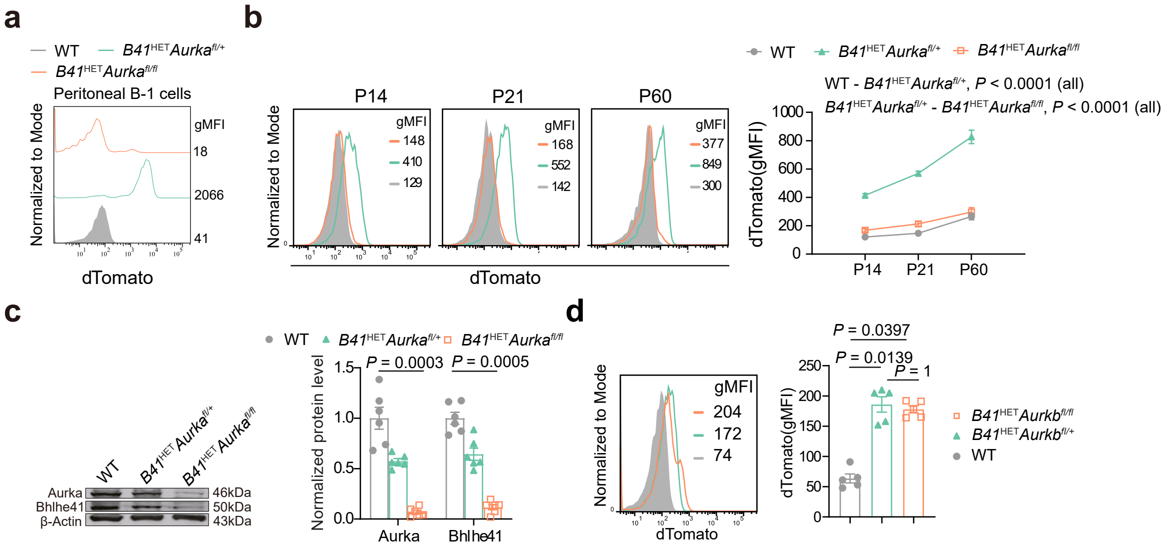

a
WT
B41HETAurkafl/+
B41HETAurkafl/fl
Peritoneal B-1 cells
Normalized to Mode
gMFI
18
2066
41
dTomato
b
P14
P21
P60
Normalized to Mode
gMFI
148
410
129
gMFI
168
552
142
gMFI
377
849
300
dTomato
WT
B41HETAurkafl/+
B41HETAurkafl/fl
WT - B41HETAurkafl/+, P < 0.0001 (all)
B41HETAurkafl/+ - B41HETAurkafl/fl, P < 0.0001 (all)
dTomato(gMFI)
1000
800
600
400
200
0
P14
P21
P60
c
WT
B41HETAurkafl/+
B41HETAurkafl/fl
Aurka
Bhlhe41
β-Actin
46kDa
50kDa
43kDa
WT
B41HETAurkafl/+
B41HETAurkafl/fl
Normalized protein level
1.5
1.0
0.5
0.0
P = 0.0003
P = 0.0005
Aurka
Bhlhe41
d
Normalized to Mode
gMFI
204
172
74
dTomato
P = 0.0397
P = 0.0139
P = 1
dTomato(gMFI)
250
200
150
100
50
0
B41HETAurkbfl/fl
B41HETAurkbfl/+
WT

Supplementary Figure 4. Loss of *Aurka* but not *Aurkb* reduces Bhlhe41 expression in microglia.

a) Representative flow cytometric analysis of Bhlhe41 expression (dTomato^{+}) in peritoneal B-1 cells (CD19^{+}B220^{low}CD43^{+}) from WT, *B41*^{HET}*Aurka*$^{fl/+}$ and *B41*^{HET}*Aurka*$^{fl/fl}$ mice (n = 3 mice per genotype).

b) Flow cytometric analysis of Bhlhe41 expression (dTomato^{+}) in microglia from WT, *B41*^{HET}*Aurka*$^{fl/+}$ and *B41*^{HET}*Aurka*$^{fl/fl}$ mice (n = 3 mice per genotype per timepoint) at P14, P21 and P60. Fluorescence intensity histograms are normalized to their respective modes.

c) WB analysis of Aurka and Bhlhe41 expression in microglia from WT, *B41*^{HET}*Aurka*$^{fl/+}$ and *B41*^{HET}*Aurka*$^{fl/fl}$ mice (n = 6 mice per genotype. Band intensities were quantified using ImageJ.

d) Flow cytometric analysis of Bhlhe41 expression (dTomato^{+}) in microglia from WT, *B41*^{HET}*Aurkb*$^{fl/+}$ and *B41*^{HET}*Aurkb*$^{fl/fl}$ mice (n = 5 mice per genotype). Fluorescence intensity histograms are normalized to their respective modes. Data are presented as mean ± SEM. Two-way ANOVA on log2 transformed gMFI with Bonferroni-corrected post hoc comparisons (**b**); Kruskal-Wallis test with Bonferroni-corrected post hoc comparisons (**c, d**). Source data are provided as a Source Data file.

a

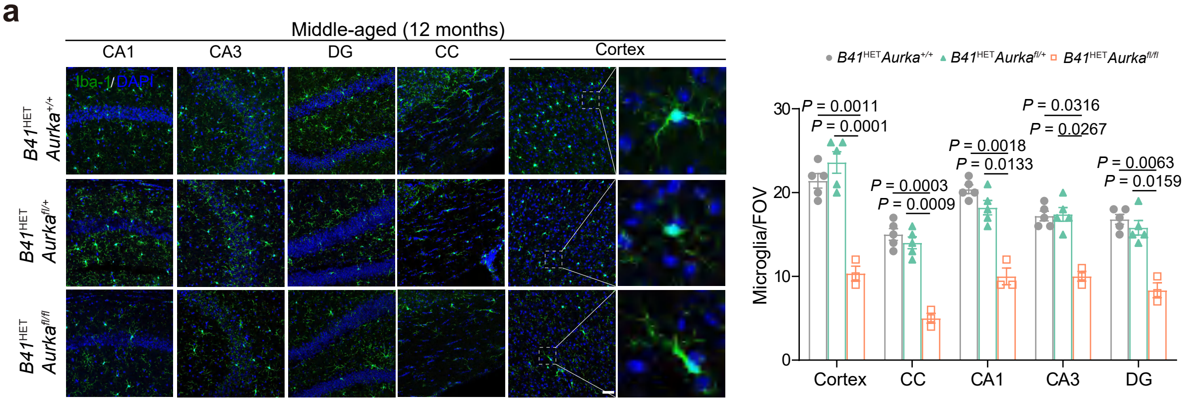

Middle-aged (12 months)
CA1
CA3
DG
CC
Cortex
Iba-1/DAPI
B41HET Aurka+/+
B41HET Aurkafl/+
B41HET Aurkafl/fl
B41HETAurka+/+
B41HETAurkafl/+
B41HETAurkafl/fl
Microglia/FOV
0
10
20
30
Cortex
CC
CA1
CA3
DG
P = 0.0011
P = 0.0001
P = 0.0003
P = 0.0009
P = 0.0018
P = 0.0133
P = 0.0316
P = 0.0267
P = 0.0063
P = 0.0159

b

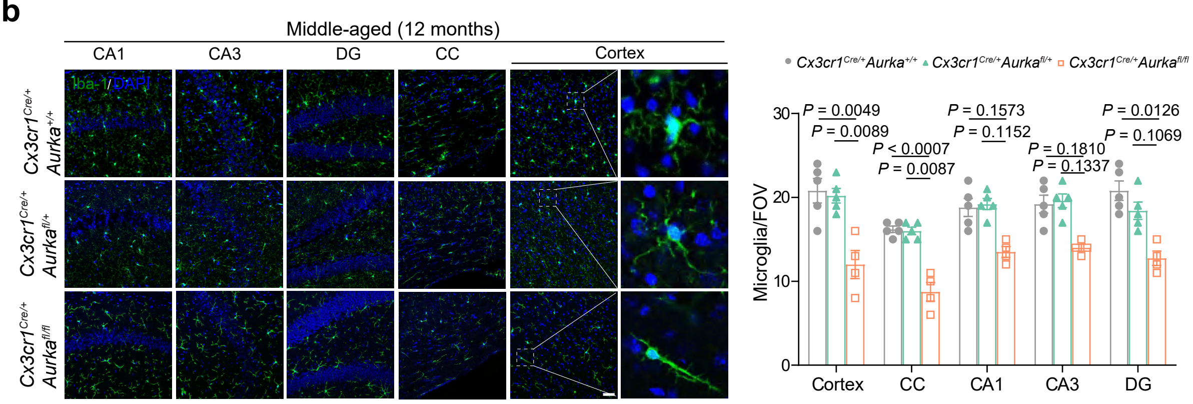

Middle-aged (12 months)
CA1
CA3
DG
CC
Cortex
Iba-1/DAPI
Cx3cr1Cre/+ Aurka+/+
Cx3cr1Cre/+ Aurkafl/+
Cx3cr1Cre/+ Aurkafl/fl
Cx3cr1Cre/+Aurka+/+
Cx3cr1Cre/+Aurkafl/+
Cx3cr1Cre/+Aurkafl/fl
Microglia/FOV
0
10
20
30
Cortex
CC
CA1
CA3
DG
P = 0.0049
P = 0.0089
P < 0.0007
P = 0.0087
P = 0.1573
P = 0.1152
P = 0.1810
P = 0.1337
P = 0.0126
P = 0.1069

Supplementary Figure 5. ***Aurka*** **deficiency led to reduced microglia numbers and rod-shaped morphology in middle-aged mice.**

a-b) IF analysis of microglia (Iba-1^{+}) in the cerebral cortex, CC and hippocampus of middle-aged **a)** WT (n = 5 mice)，*B41*^{HET}*Aurka*$^{fl/+}$ (n = 5 mice) and *B41*^{HET}*Aurka*$^{fl/fl}$ mice (n = 3 mice), and **b)** WT (n = 5 mice), *Cx3cr1*$^{Cre/+}$*Aurka*$^{fl/+}$ (n = 5 mice) and *Cx3cr1*$^{Cre/+}$*Aurka*$^{fl/fl}$ mice (n = 4 mice). Data are presented as mean ± SEM. Poisson GLM (two-sided, **a**, **b**) with Bonferroni-corrected post hoc comparisons. Scale bar for IF images:50 μm. Source data are provided as a Source Data file.

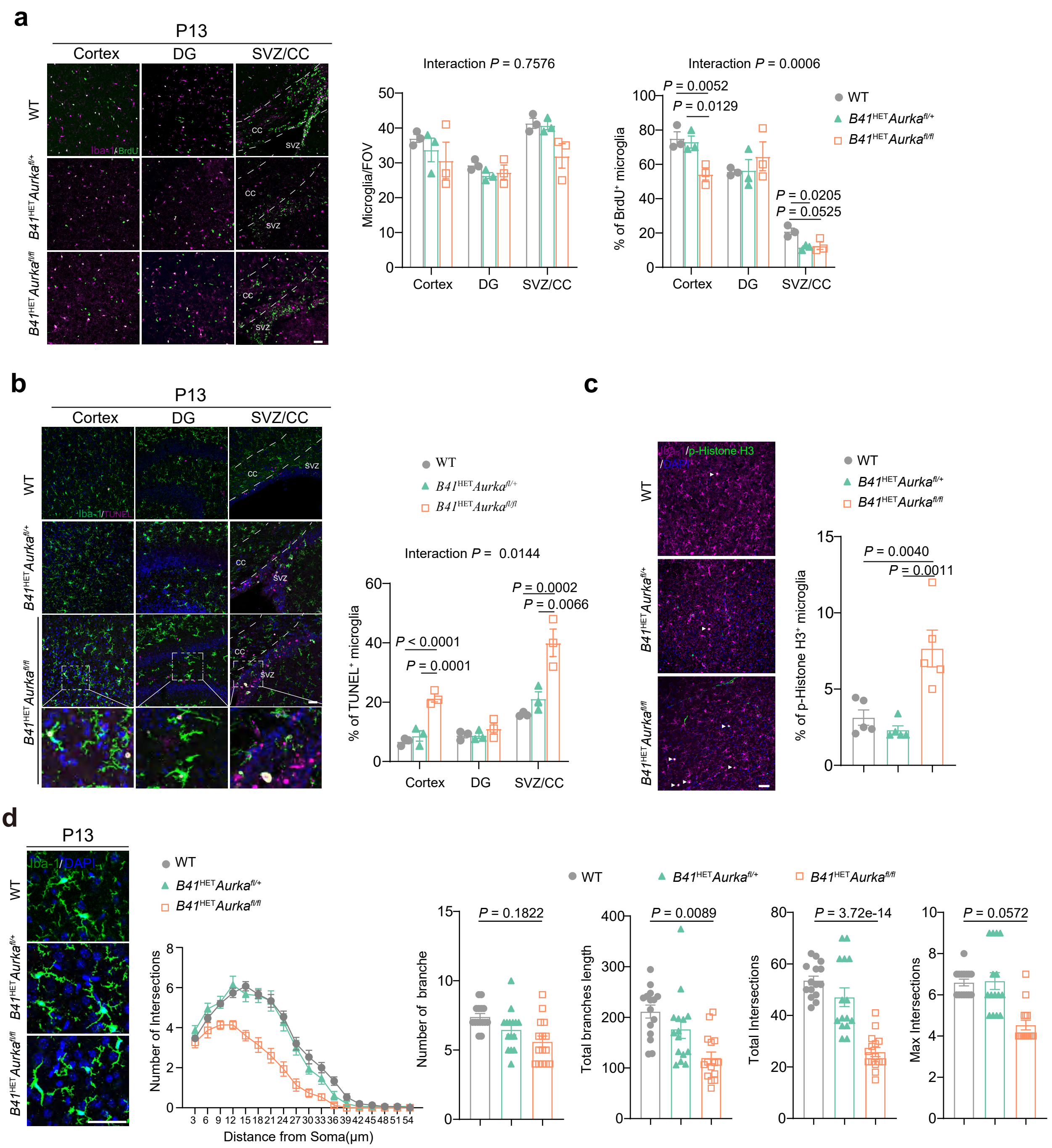
a
P13
Cortex
DG
SVZ/CC
WT
B41HETAurkafl/+
B41HETAurkafl/fl
Interaction P = 0.7576
Microglia/FOV
Interaction P = 0.0006
P = 0.0052
P = 0.0129
P = 0.0205
P = 0.0525
% of BrdU+ microglia
WT
B41HETAurkafl/+
B41HETAurkafl/fl
b
P13
Cortex
DG
SVZ/CC
Interaction P = 0.0144
P < 0.0001
P = 0.0001
P = 0.0002
P = 0.0066
% of TUNEL+ microglia
c
Iba-1/p-Histone H3/DAPI
P = 0.0040
P = 0.0011
% of p-Histone H3+ microglia
d
P13
Iba-1/DAPI
Number of Intersections
Distance from Soma(μm)
P = 0.1822
Number of branche
P = 0.0089
Total branches length
P = 3.72e-14
Total Intersections
P = 0.0572
Max Intersections

Supplementary Figure 6. *Aurka* deficiency impairs infant microglial survival, mitosis and morphology.

a-c) IF analysis of **a**) the proliferation (BrdU$^+$), **b**) apoptosis (TUNEL$^+$), and **c**) mitosis (p-Histone H3$^+$) of microglia (Iba-1$^+$) from infant WT, $B41^{\mathrm{HET}}Aurka^{fl/+}$ and $B41^{\mathrm{HET}}Aurka^{fl/fl}$ mice (P13, n = 3 mice per genotype). For the analysis of microglial proliferation, 100 µL of BrdU solution (10 mg/mL) were *i.p* injected once every 12 hours for 3 days before euthanasia.

d) Sholl analysis of microglial processes and branch intersections in infant WT, $B41^{\mathrm{HET}}Aurka^{fl/+}$ and $B41^{\mathrm{HET}}Aurka^{fl/fl}$ mice (P13, n = 3 mice per genotype). Five microglia per mouse were quantified. DG, dentate gyrus. SVZ, subventricular zone; CC, corpus callosum. Data are presented as mean ± SEM. Poisson GLM (two-sided, **a**); Two-way ANOVA on log2 transformed data (**b**); One-way ANOVA (**c**); LMM for continuous data and negative binomial GLMM for count data (two-sided), with repeated measures from the same mouse accounted for as a random effect (**d**). All post hoc pairwise comparisons were adjusted using the Bonferroni correction (**a-d**). Iba-1 (AF555, **a**, **c**) and TUNEL (AF594, **b**) shown in magenta (pseudocolor applied). Scale bar for IF images:50 µm. Source data are provided as a Source Data file.

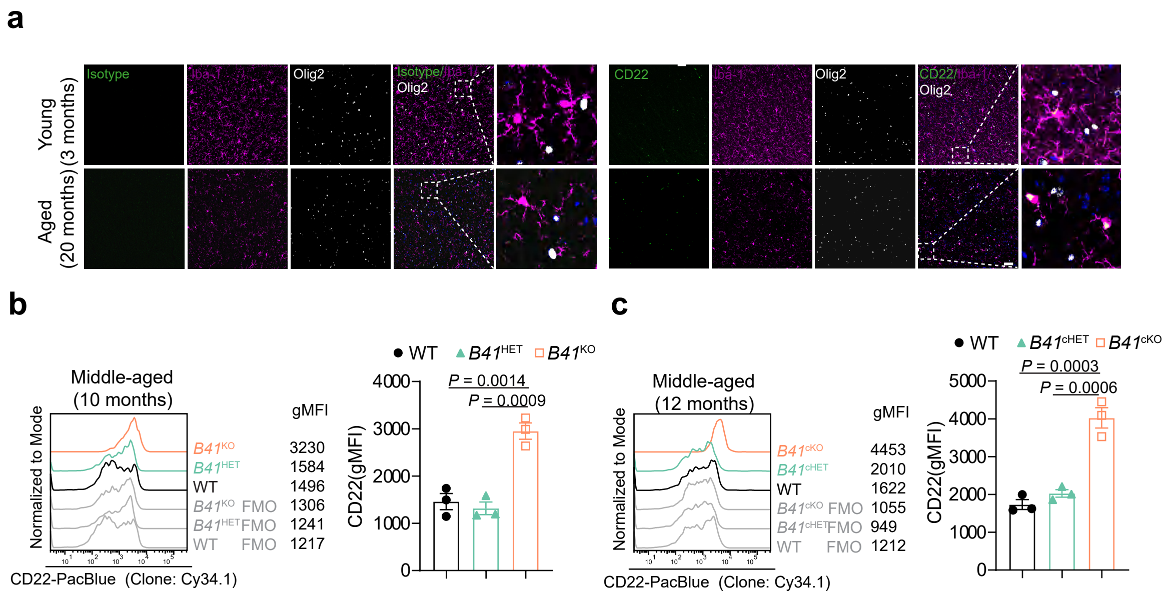
a
Young (3 months)
Aged (20 months)
Isotype
Iba-1
Olig2
Isotype/Iba-1/Olig2
CD22
CD22/Iba-1/Olig2
b
Middle-aged (10 months)
gMFI
B41KO 3230
B41HET 1584
WT 1496
B41KO FMO 1306
B41HET FMO 1241
WT FMO 1217
Normalized to Mode
CD22-PacBlue (Clone: Cy34.1)
WT B41HET B41KO
P = 0.0014
P = 0.0009
CD22(gMFI)
c
Middle-aged (12 months)
gMFI
B41cKO 4453
B41cHET 2010
WT 1622
B41cKO FMO 1055
B41cHET FMO 949
WT FMO 1212
Normalized to Mode
CD22-PacBlue (Clone: Cy34.1)
WT B41cHET B41cKO
P = 0.0003
P = 0.0006
CD22(gMFI)

Supplementary Figure 7. CD22 upregulation on aged- and *Bhlhe41*-deficient young microglia.

a) Representative immunofluorescence images of CD22 expression in microglia (Iba-1^+) and pan-oligodendrocyte lineage cells (Olig2^+) from young (3 months, n = 3) and aged (20 months, n = 3) WT mice. Iba-1 (AF555) shown in magenta, Olig2 (AF647) shown in gray (pseudocolors applied). Scale bar for IF images:50 µm

b-c) Flow cytometric analysis of CD22 expression on microglia from middle-aged **a**) WT, *B41*^{HET} and *B41*^{KO} mice and **b**) WT, *B41*^{cHET} and *B41*^{cKO} mice using anti-CD22 (clone Cy34.1). n = 3 mice per genotype. Fluorescence intensity histograms are normalized to their respective modes. FMO, fluorescence minus one. Data are presented as mean ± SEM. One-way ANOVA with Bonferroni-corrected post hoc comparisons (**b**, **c**). Source data are provided as a Source Data file.

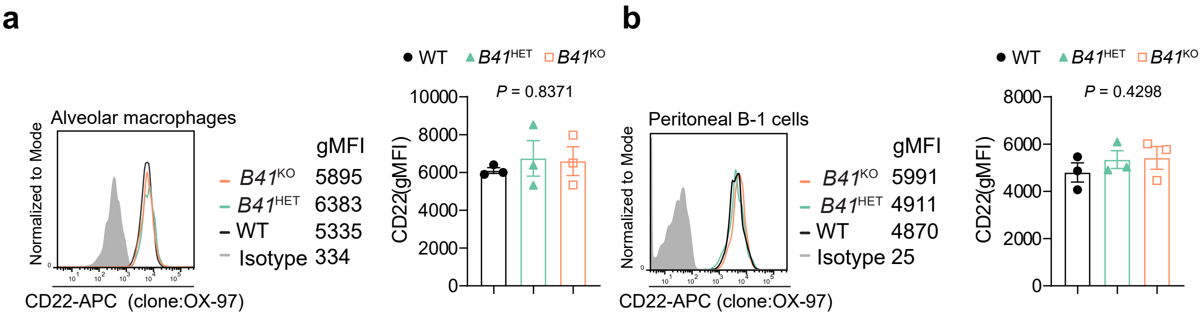

a
Alveolar macrophages
Normalized to Mode
CD22-APC (clone:OX-97)
gMFI
B41KO 5895
B41HET 6383
WT 5335
Isotype 334
WT B41HET B41KO
P = 0.8371
CD22(gMFI)
10000
8000
6000
4000
2000
0
b
Peritoneal B-1 cells
Normalized to Mode
CD22-APC (clone:OX-97)
gMFI
B41KO 5991
B41HET 4911
WT 4870
Isotype 25
WT B41HET B41KO
P = 0.4298
CD22(gMFI)
8000
6000
4000
2000
0

Supplementary Figure 8. *Bhlhe41* deficiency does not affect CD22 expression on alveolar macrophages or peritoneal B-1 cells.

a-b) Flow cytometric analysis of CD22 expression on **a**) alveolar macrophages and **b**) peritoneal B-1 cells from young WT, $B41^{HET}$ and $B41^{KO}$ mice (n = 3 mice per genotype). Fluorescence intensity histograms are normalized to their respective modes. Data are presented as mean ± SEM. Kruskal-Wallis test (**a**, **b**). Source data are provided as a Source Data file.

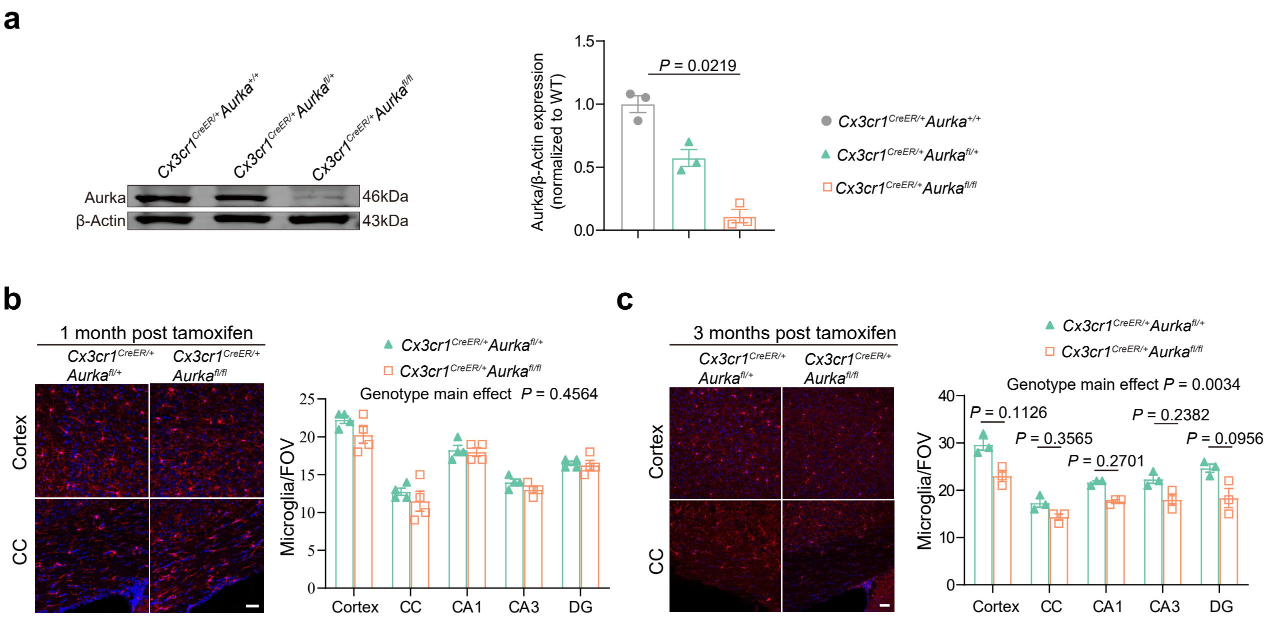
a
$Cx3cr1^{CreER/+}$ $Aurka^{+/+}$
$Cx3cr1^{CreER/+}$ $Aurka^{fl/+}$
$Cx3cr1^{CreER/+}$ $Aurka^{fl/fl}$
Aurka
β-Actin
46kDa
43kDa
Aurka/β-Actin expression (normalized to WT)
1.5
1.0
0.5
0.0
P = 0.0219
b
1 month post tamoxifen
$Cx3cr1^{CreER/+}$ $Aurka^{fl/+}$
$Cx3cr1^{CreER/+}$ $Aurka^{fl/fl}$
Cortex
CC
Genotype main effect P = 0.4564
Microglia/FOV
25
20
15
10
5
0
Cortex
CC
CA1
CA3
DG
c
3 months post tamoxifen
Genotype main effect P = 0.0034
P = 0.1126
P = 0.3565
P = 0.2701
P = 0.2382
P = 0.0956
40
30
20
10
0

Supplementary Figure 9. IF analysis of microglia in tamoxifen-induced microglia-specific *Aurka*-deficient mice.

a) WB analysis of Aurka expression in $Cx3cr1^{CreER/+}Aurka^{+/+}$, $Cx3cr1^{CreER/+}Aurka^{fl/+}$ and $Cx3cr1^{CreER/+}Aurka^{fl/fl}$ mice (n = 3 mice per genotype) 1 month after administration of tamoxifen (75 mg/kg) for 5 consecutive days. Band intensities were quantified using ImageJ.

b-c) IF analysis of microglia in young $Cx3cr1^{CreER/+}Aurka^{fl/+}$ and $Cx3cr1^{CreER/+}Aurka^{fl/fl}$ mice **a**) 1 month (n = 4 mice per genotype) and **b**) 3 months (n = 3 mice per genotype) after administration of tamoxifen (75 mg/kg) for 5 consecutive days. CC, corpus callosum. Data are presented as mean ± SEM. Kruskal-Wallis test (**a**); Poisson GLM (two-sided, **b**, **c**); All post hoc pairwise comparisons were adjusted using the Bonferroni correction (**a**, **c**). Scale bar for IF images:50 µm. Source data are provided as a Source Data file.

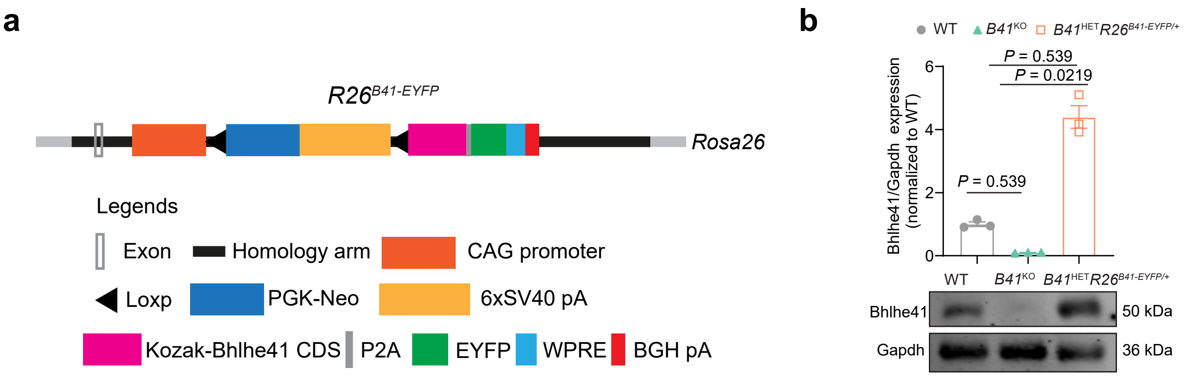
a
R26B41-EYFP
Rosa26
Legends
Exon
Homology arm
CAG promoter
Loxp
PGK-Neo
6xSV40 pA
Kozak-Bhlhe41 CDS
P2A
EYFP
WPRE
BGH pA
b
WT
B41KO
B41HETR26B41-EYFP/+
P = 0.539
P = 0.0219
P = 0.539
Bhlhe41/Gapdh expression (normalized to WT)
Bhlhe41
Gapdh
50 kDa
36 kDa

Supplementary Figure 10. Generation and validation of *Bhlhe41* transgenic mice.

a) Schematic representation of a *CAG* promoter-*loxP*-*PGK*-*Neo*-*6xSV40 pA*-*loxP*-*Kozak*-*Bhlhe41* CDS-*P2A*-*EYFP*-*WPRE*-*BGH pA* cassette that was integrated into the *Rosa26* locus in $Rosa26^{Bhlhe41\text{-}EYFP}$ mice (referred to as $R26^{B41\text{-}EYFP}$).

b) Validation of $Rosa26^{Bhlhe41\text{-}EYFP}$ mice by WB analysis of Bhlhe41 expression in isolated microglia from $B41^{\mathrm{HET}}R26^{B41\text{-}EYFP/+}$ mice, alongside microglia from WT and $B41^{\mathrm{KO}}$ mice (n = 3 mice per genotype). Band intensities were quantified using ImageJ. Data are presented as mean ± SEM. Kruskal-Wallis test with Bonferroni-corrected post hoc comparisons (**b**). Source data are provided as a Source Data file.

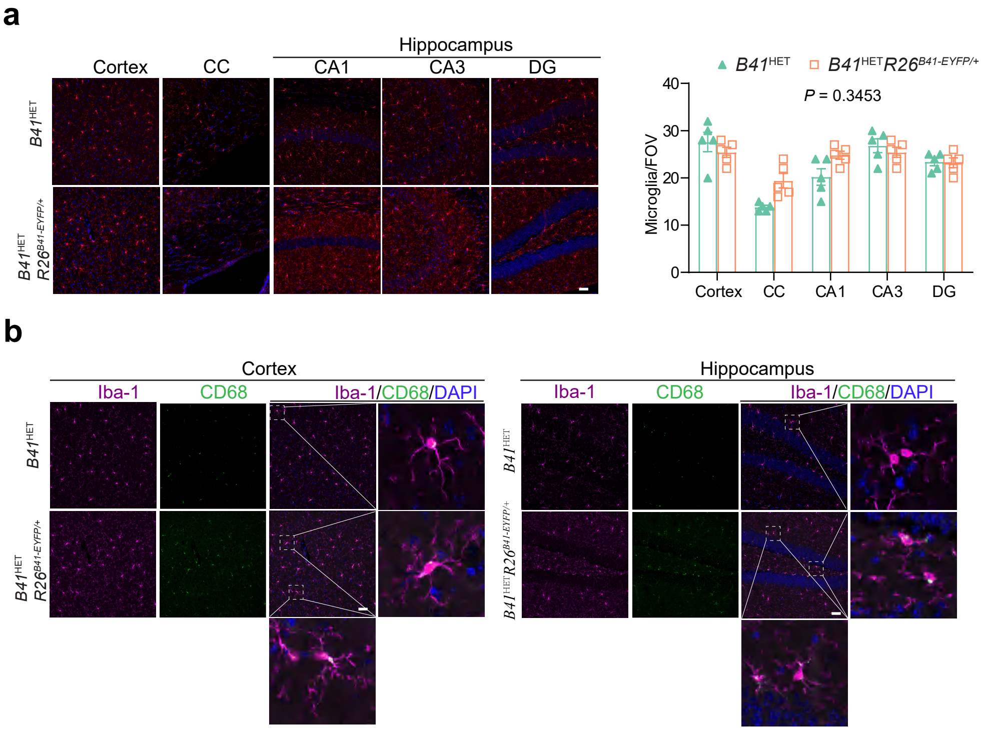
a
Hippocampus
Cortex
CC
CA1
CA3
DG
$B41^{HET}$
$B41^{HET}$ $R26^{B41-EYFP/+}$
$B41^{HET}$
$B41^{HET}R26^{B41-EYFP/+}$
P = 0.3453
Microglia/FOV
40
30
20
10
0
Cortex
CC
CA1
CA3
DG
b
Cortex
Iba-1
CD68
Iba-1/CD68/DAPI
Hippocampus
Iba-1
CD68
Iba-1/CD68/DAPI
$B41^{HET}$
$B41^{HET}$ $R26^{B41-EYFP/+}$
$B41^{HET}$
$B41^{HET}R26^{B41-EYFP/+}$

Supplementary Figure 11. Microglia numbers and CD68 expression in microglial *Bhlhe41* transgenic mice.

a) IF analysis of microglia (Iba-1^{+}) in the cerebral cortex, CC and hippocampus of young *B41*^{HET} and *B41*^{HET}*R26*$^{B41\text{-}EYFP/+}$ mice (n = 5 mice per genotype) at 3 months of age.

b) Representative IF images of CD68 in the cerebral cortex and hippocampus of young *B41*^{HET} and *B41*^{HET}*R26*$^{B41\text{-}EYFP/+}$ mice (n = 5 mice per genotype). Data are presented as mean ± SEM. Poisson GLM (two-sided, **a**). Iba-1 (AF647) shown in magenta, CD68 (AF555) shown in green (pseudocolors applied). Scale bar for IF images:50 µm. Source data are provided as a Source Data file.

a

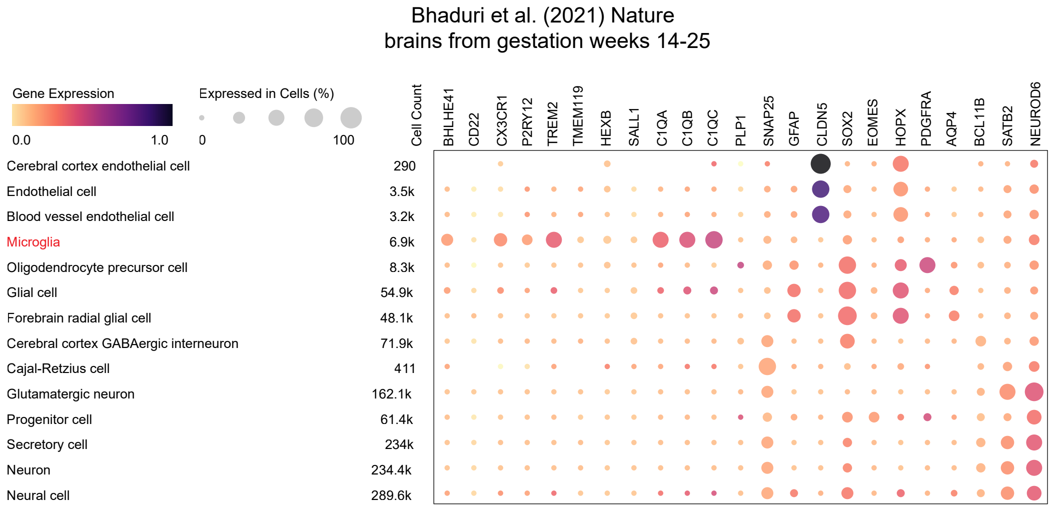

Bhaduri et al. (2021) Nature
brains from gestation weeks 14-25
Gene Expression
0.0
1.0
Expressed in Cells (%)
0
100
Cell Count
BHLHE41
CD22
CX3CR1
P2RY12
TREM2
TMEM119
HEXB
SALL1
C1QA
C1QB
C1QC
PLP1
SNAP25
GFAP
CLDN5
SOX2
EOMES
HOPX
PDGFRA
AQP4
BCL11B
SATB2
NEUROD6
Cerebral cortex endothelial cell 290
Endothelial cell 3.5k
Blood vessel endothelial cell 3.2k
Microglia 6.9k
Oligodendrocyte precursor cell 8.3k
Glial cell 54.9k
Forebrain radial glial cell 48.1k
Cerebral cortex GABAergic interneuron 71.9k
Cajal-Retzius cell 411
Glutamatergic neuron 162.1k
Progenitor cell 61.4k
Secretory cell 234k
Neuron 234.4k
Neural cell 289.6k

b

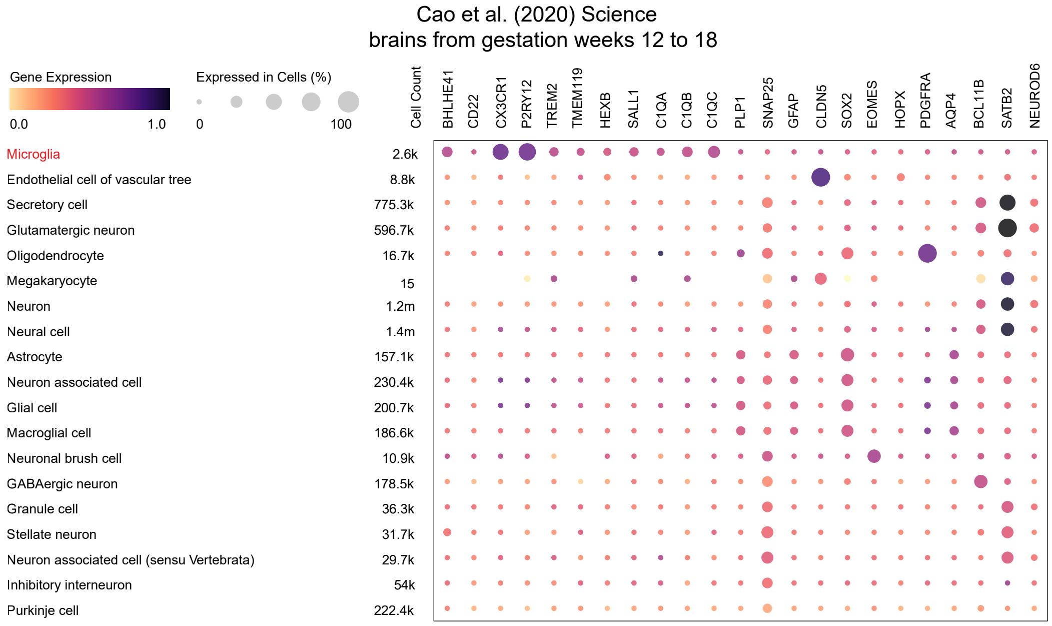

Cao et al. (2020) Science
brains from gestation weeks 12 to 18
Gene Expression
0.0
1.0
Expressed in Cells (%)
0
100
Cell Count
BHLHE41
CD22
CX3CR1
P2RY12
TREM2
TMEM119
HEXB
SALL1
C1QA
C1QB
C1QC
PLP1
SNAP25
GFAP
CLDN5
SOX2
EOMES
HOPX
PDGFRA
AQP4
BCL11B
SATB2
NEUROD6
Microglia 2.6k
Endothelial cell of vascular tree 8.8k
Secretory cell 775.3k
Glutamatergic neuron 596.7k
Oligodendrocyte 16.7k
Megakaryocyte 15
Neuron 1.2m
Neural cell 1.4m
Astrocyte 157.1k
Neuron associated cell 230.4k
Glial cell 200.7k
Macroglial cell 186.6k
Neuronal brush cell 10.9k
GABAergic neuron 178.5k
Granule cell 36.3k
Stellate neuron 31.7k
Neuron associated cell (sensu Vertebrata) 29.7k
Inhibitory interneuron 54k
Purkinje cell 222.4k

Supplementary Figure 12. *BHLHE41* is highly expressed in human fetal microglia. a-b) UMAP plots illustrate the expression patterns of *BHLHE41*, CD22 and marker genes across various cell types in two independent scRNA-seq datasets from human fetal cerebellum. Dot plots were generated using the Cellxgene platform (https://cellxgene.cziscience.com/gene-expression).

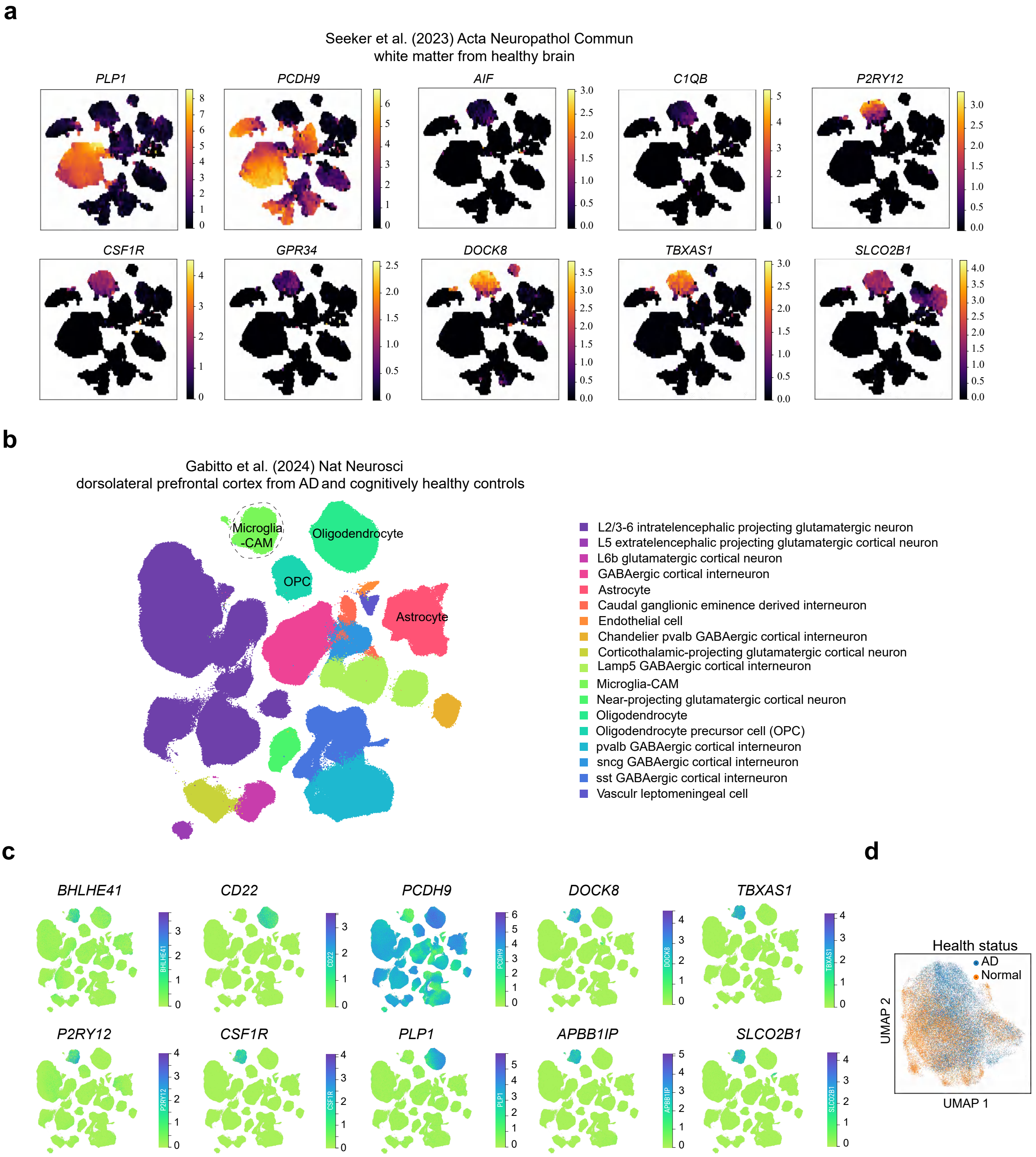
a
Seeker et al. (2023) Acta Neuropathol Commun
white matter from healthy brain
PLP1
PCDH9
AIF
C1QB
P2RY12
CSF1R
GPR34
DOCK8
TBXAS1
SLCO2B1
b
Gabitto et al. (2024) Nat Neurosci
dorsolateral prefrontal cortex from AD and cognitively healthy controls
Microglia
-CAM
Oligodendrocyte
OPC
Astrocyte
L2/3-6 intratelencephalic projecting glutamatergic neuron
L5 extratelencephalic projecting glutamatergic cortical neuron
L6b glutamatergic cortical neuron
GABAergic cortical interneuron
Astrocyte
Caudal ganglionic eminence derived interneuron
Endothelial cell
Chandelier pvalb GABAergic cortical interneuron
Corticothalamic-projecting glutamatergic cortical neuron
Lamp5 GABAergic cortical interneuron
Microglia-CAM
Near-projecting glutamatergic cortical neuron
Oligodendrocyte
Oligodendrocyte precursor cell (OPC)
pvalb GABAergic cortical interneuron
sncg GABAergic cortical interneuron
sst GABAergic cortical interneuron
Vasculr leptomeningeal cell
c
BHLHE41
CD22
PCDH9
DOCK8
TBXAS1
P2RY12
CSF1R
PLP1
APBB1IP
SLCO2B1
d
Health status
AD
Normal
UMAP 2
UMAP 1

Supplementary Figure 13. Subclusters and expression pattern of marker genes in human CNS cells.

a) The UMAP plot illustrates the expression of *PCDH9* and the marker genes for microglia and oligodendrocytes in scRNA-seq data from human healthy white matter. **b-d**) The UMAP plot visualizes **b**) cell clusters of human CNS cells, **c**) the expression of *BHLHE41, CD22* and marker genes of microglia and oligodendrocytes. Dot plots were generated using the Cellxgene platform (https://cellxgene.cziscience.com/gene-expression). **d**) microglia subsets from the prefrontal cortex of patients with Alzheimer's disease (AD) and cognitively healthy controls (Normal).

Supplementary Table 1. Primers for in-house generated mouse genotyping

Mouse strains	Forward primer (5' to 3')	Reverse Primer (5' to 3')	PCR products
Bhlhe41$^{dTomato\text{-}Cre/+}$	*ATACTGCACTGAAGAGGGAGAGC*	*TCGCTTCAAGCTCCTTTTGG*	366 bp for wildtype allele
	ATACTGCACTGAAGAGGGAGAGC	*CTTGGAGCCGTACATGAACTG*	339 bp for transgenic allele
Bhlhe41$^{fl/+}$	*GCTTTCCCTTGCTTGTCGTC*	*CGCTTTAGAGG ACGTTTGAACTTG*	283 bp for wildtype allele and 351 bp for transgenic allele
Aurkb$^{fl/+}$	*TGGTCTCCCTATGTTGCCTTAAA*	*GCCTGTAGATGAGTCAGAGAGCTA*	192 bp for wildtype allele and 259 bp for transgenic allele
Rosa26$^{Bhlhe41\text{-}EYFP/+}$	*GCAGGAGATGTTGAAGAAAACCC*	*GGACTTGAAGAAGTCGTGCTGC*	291 bp for transgenic allele
	CACTTGCTCTCCCAAAGTCGCTC	*ATACTCCGAGGCGGATCACAA*	453 bp for wildytpe allele

Supplementary Table 2. Primers used for the ChIP-qPCR analysis

Targeted genomic region	ID in Figure 5l	Forward primer (5' to 3')	Reverse Primer (5' to 3')	PCR product size	PCR product (5' to 3')
Cd22 intron 11	NC	*GTTTGTGCTGG CCTCTTTCA*	*GTCCACAGCA AGGGTTTCAG*	93 bp	*GTTTGTGCTGGCCTC TTTCAAATGACTGTA GCCCATCCCATCTC CCTAGCACCCTCTTC AAGGCAGAGACGAA CTGAAACCCTTGCTG TGGAC*
Cd22 promoter-1403/-1394	P1	*AGCCTGGACA AAGCAGCAT*	*TTCTCTGCAG TCACGTGTTG*	71 bp	*AGCCTGGACAAAGC AGCATGGAAATTCAT CAGACATGGAAGAC ACCCAAGCCAA CAC GTG ACTGCAGAGAA*
Bhlhe41 promoter -1206/-1197	P2	*CACGTGACCC GCTCCAG*	*CGAGAACCGA GAAGGAACCT G*	128 bp	*CACGTG ACCCGCTC CAGCCGCCCTGGCC CGCGACCCAGATCC GTCCCCGGAACCCA AGCGCCGCTGCCCT GGCCCTGCTGCGCT CCAGTGCGCGTGCA TTTGGGGCCCAGGT TCCTTCTCGGTTCTC G*
Bhlhe41 promoter -17/-8	P3	*CCGCGGATGG TACGTTCC*	*CGCTGGTAGT TTGCTCTCAC*	102 bp	*CCGCGGATGGTACG TTCCG CACGTG AGC TGGGTGCTGGTCTG GCCGGCGACGCGC GTGCCCTGTGGCCA AACACTGCCCGGAG TGAGAGCAAACTACC AGCG*
