# Supplementary material for: Aurka-Bhlhe41 axis prevents premature aging-like microglial dysfunction and promotes remyelination
Source: Nat Commun. 2026 Mar 27;17:5238. doi: 10.1038/s41467-026-71014-w (PMC13260908; doi:10.1038/s41467-026-71014-w)
Supplement: Supplementary file 2 — Description of Additional Supplementary Files [file 41467_2026_71014_MOESM2_ESM.pdf]

Description of Additional Supplementary Files

Supplementary Data 1. Shared differentially expressed genes in Bhlhe41-dificient microglia
