# Supplementary material for: Aurka-Bhlhe41 axis prevents premature aging-like microglial dysfunction and promotes remyelination
Source: Nat Commun. 2026 Mar 27;17:5238. doi: 10.1038/s41467-026-71014-w (PMC13260908; doi:10.1038/s41467-026-71014-w)
Supplement: Supplementary file 4 — Reporting Summary [file 41467_2026_71014_MOESM4_ESM.pdf]

nature portfolio

Corresponding author(s): Hui Wang

Last updated by author(s): Feb 23, 2026

Reporting Summary

Nature Portfolio wishes to improve the reproducibility of the work that we publish. This form provides structure for consistency and transparency in reporting. For further information on Nature Portfolio policies, see our Editorial Policies and the Editorial Policy Checklist.

Statistics

For all statistical analyses, confirm that the following items are present in the figure legend, table legend, main text, or Methods section.

n/a	Confirmed	
☐	☒	The exact sample size (*n*) for each experimental group/condition, given as a discrete number and unit of measurement
☐	☒	A statement on whether measurements were taken from distinct samples or whether the same sample was measured repeatedly
☐	☒	The statistical test(s) used AND whether they are one- or two-sided<br>*Only common tests should be described solely by name; describe more complex techniques in the Methods section.*
☐	☒	A description of all covariates tested
☐	☒	A description of any assumptions or corrections, such as tests of normality and adjustment for multiple comparisons
☐	☒	A full description of the statistical parameters including central tendency (e.g. means) or other basic estimates (e.g. regression coefficient) AND variation (e.g. standard deviation) or associated estimates of uncertainty (e.g. confidence intervals)
☐	☒	For null hypothesis testing, the test statistic (e.g. *F*, *t*, *r*) with confidence intervals, effect sizes, degrees of freedom and *P* value noted<br>*Give P values as exact values whenever suitable.*
☒	☐	For Bayesian analysis, information on the choice of priors and Markov chain Monte Carlo settings
☒	☐	For hierarchical and complex designs, identification of the appropriate level for tests and full reporting of outcomes
☒	☐	Estimates of effect sizes (e.g. Cohen's *d*, Pearson's *r*), indicating how they were calculated

Our web collection on statistics for biologists contains articles on many of the points above.

Software and code

Policy information about availability of computer code

Data collection	ZEN software, v3.9; Leica Application Suite X, v3.9; BD FACS DIVA v9; Feature Extraction software, v11.5.1.1; LightCycler 480 v1.5.1.62; Image Studio v5.5.
Data analysis	R (v4.2.2) with the following packages: lme4 (v1.1-33), lmerTest (v3.1-3), emmeans (v1.11.1), DHARMa (v0.4.7), MASS (v7.3-60), nlme (v3.1-162), rstatix (v0.7.2), ARTool (v0.11.2), limma v3.54.2. Scanpy v1.11.5 package in Python (v3.12); Flowjo (v10.8.1); Image J (v1.54);GraphPad Prism v10; The codes for the scRNA-seq data analysis are available at https://github.com/YelinZhao-A/Microglia_BHLHE41.

For manuscripts utilizing custom algorithms or software that are central to the research but not yet described in published literature, software must be made available to editors and reviewers. We strongly encourage code deposition in a community repository (e.g. GitHub). See the Nature Portfolio guidelines for submitting code & software for further information.

Data

Policy information about availability of data

All manuscripts must include a data availability statement. This statement should provide the following information, where applicable:

- Accession codes, unique identifiers, or web links for publicly available datasets
- A description of any restrictions on data availability
- For clinical datasets or third party data, please ensure that the statement adheres to our policy

In-house generated gene expression microarray data has been deposited in Gene Expression Omnibus (GEO) under accession code GSE276929 [https://

www.ncbi.nlm.nih.gov/geo/query/acc.cgi?acc=GSE276929].The public bulk RNA-seq data from Bhlhe40/Bhlhe41 knockout microglia, and from yolk sac myeloid progenitors and microglia across multiple developmental stages used in this study are available in GEO under accession code GSE254233[https://www.ncbi.nlm.nih.gov/geo/query/acc.cgi?acc=GSE254233]33 and GSE79818[https://www.ncbi.nlm.nih.gov/geo/query/acc.cgi?acc=GSE79818]22. The single-cell RNA sequencing (scRNA-seq) datasets used in this study are available in the Cellxgene platform, including scRNA-seq data from human post-mortem white matter from healthy brains [https://cellxgene.cziscience.com/collections/9d63fcf1-5ca0-4006-8d8f-872f3327dbe9] 43, the substantia nigra pars compacta of patients with Parkinson's disease (PD) [https://cellxgene.cziscience.com/collections/b0f0b447-ac37-45b0-b1bf-5c0b7d871120]47, the dorsolateral prefrontal cortex of patients with Alzheimer's disease (AD) [https://cellxgene.cziscience.com/collections/1ca90a2d-2943-483d-b678-b809bf464c30]46, fetal cerebellum between gestation week 10-18 [https://cellxgene.cziscience.com/collections/c114c20f-1ef4-49a5-9c2e-d965787fb90c] 45, and neocortex between gestation week 14-25 [https://cellxgene.cziscience.com/collections/c8565c6a-01a1-435b-a549-f11b452a83a8] 44. Source Data are provided with this paper and has been deposited in Figshare [https://doi.org/10.6084/m9.figshare.30896234].

Research involving human participants, their data, or biological material

Policy information about studies with human participants or human data. See also policy information about sex, gender (identity/presentation), and sexual orientation and race, ethnicity and racism.

Reporting on sex and gender	N/A
Reporting on race, ethnicity, or other socially relevant groupings	N/A
Population characteristics	N/A
Recruitment	N/A
Ethics oversight	N/A

Note that full information on the approval of the study protocol must also be provided in the manuscript.

Field-specific reporting

Please select the one below that is the best fit for your research. If you are not sure, read the appropriate sections before making your selection.

☒ Life sciences ☐ Behavioural & social sciences ☐ Ecological, evolutionary & environmental sciences

For a reference copy of the document with all sections, see nature.com/documents/nr-reporting-summary-flat.pdf

Life sciences study design

All studies must disclose on these points even when the disclosure is negative.

Sample size	For in vitro experiments, at least three independent biological replicates per group were used, consistent with standard practice in the field to detect reproducible cellular phenotypes. For in vivo experiments, a minimum of four mice per group were included. These sample sizes were chosen based on prior experience and preliminary data, which indicated that they were sufficient to detect expected biological differences while minimizing the number of animals used. Formal power calculations were not performed.
Data exclusions	No data were excluded from the analyses.
Replication	All key in vivo experiments were conducted in at least two independent experiments to ensure reproducibility. Flow cytometry and immunofluorescence analyses were repeated in at least two independent experiments with consistent results. Single-cell RNA sequencing (scRNA-seq) analysis of CD22 in human microglia was validated using two independent datasets. All attempts at replication were successful for the key findings.
Randomization	Mice were first stratified by genotype and sex and then randomly allocated to experimental groups. Sex-matched mice of different genotypes were used in all experiments, except for cuprizone-induced demyelination and in vivo phagocytosis assays, which were performed exclusively in male mice to minimize biological variability.
Blinding	For experiments involving the CPZ-induced demyelination model, including Black-Gold II myelin staining, immunofluorescence analysis, and TEM of brain sections, investigators were blinded to group allocation during data collection and analysis. For other experiments (e.g. flow cytometric analysis of microglial phagocytosis and CD22), blinding was not applied as the outcomes were quantitative and objective measurements, minimizing potential bias.

Reporting for specific materials, systems and methods

We require information from authors about some types of materials, experimental systems and methods used in many studies. Here, indicate whether each material, system or method listed is relevant to your study. If you are not sure if a list item applies to your research, read the appropriate section before selecting a response.

Materials & experimental systems

n/a	Involved in the study
☐	☒ Antibodies
☒	☐ Eukaryotic cell lines
☒	☐ Palaeontology and archaeology
☐	☒ Animals and other organisms
☒	☐ Clinical data
☒	☐ Dual use research of concern
☒	☐ Plants

Methods

n/a	Involved in the study
☒	☐ ChIP-seq
☐	☒ Flow cytometry
☒	☐ MRI-based neuroimaging

Antibodies

Antibodies used

CD4 Monoclonal Antibody (GK1.5), eFluor™ 450, eBioscience Thermo Fisher Scientific Cat# 48-0041-82,
CD11b Monoclonal Antibody (M1/70), eFluor™ 450, eBioscience Thermo Fisher Scientific Cat# 48-0112-82,
CD16/CD32 Monoclonal Antibody (93), eBioscience ThermoFisher Scientific Cat# 14-0161-86,
PerCP/Cyanine5.5 anti-mouse/human CD11b Antibody, clone M1/70 BioLegend Cat# 101228,
APC anti-mouse CD11c Antibody, clone N418 BioLegend Cat# 117310,
APC anti-mouse CD19 Antibody, clone 6D5 BioLegend Cat# 115512,
APC anti-mouse CD22 Antibody, clone OX-97 BioLegend Cat# 126110,
APC Rat IgG1, κ Isotype Ctrl Antibody BioLegend Cat# 400411,
FITC anti-mouse CD24 Antibody, clone M1/69 BioLegend Cat# 101806,
FITC anti-mouse CD45 Antibody, clone 30-F11 BioLegend Cat# 103108,
PE/Cyanine7 anti-mouse CD45 Antibody, clone 30-F11 BioLegend Cat# 103114,
PerCP/Cy5.5 anti-mouse/human CD45R/B220 Antibody, clone RA3-6B2 BioLegend Cat# 103236,
PerCP/Cyanine5.5 anti-mouse CX3CR1 Antibody, clone SA011F11 BioLegend Cat# 149010,
APC anti-mouse CX3CR1 Antibody, clone SA011F11 BioLegend Cat# 149008,
APC anti-mouse IFN-γ Antibody, clone XMG1.2 BioLegend Cat# 505810,
PE anti-mouse IL-17A Antibody,clone TC11-18H10.1 BioLegend Cat# 506904,
Mouse anti-GFAP Antibody, clone 2E1.E9 BioLegend Cat# 644702,
Mouse anti-NeuN Antibody, clone A60 Millipore Cat# MAB377,
Rabbit anti-Iba-1 Antibody Wako Cat# 019-17409,
Anti-Olig2 Antibody, clone 211F1.1 Sigma-Aldrich Cat# MABN50,
Rabbit anti-Olig2 Proteintech Cat# 13999-1-AP,
Rat anti-Mouse CD206 Monoclonal antibody,clone MR5D3 Bio-Rad Cat# MCA2235,
Rabbit anti-RFP Antibody Rockland Cat# 600-401-379S,
Goat anti-dTomato Biorbyt Cat# orb182397,
InVivoMab anti-mouse CD22 Antibody, clone Cy34.1 BioXcell Cat# BE0011,
IgG1 Isotype , clone MOPC-21 BioXcell Cat# BE0083,
Mouse anti-phospho-Histone H3(Ser10) Cell Signaling Technology Cat# 9706S,
Rat anti-BrdU Antibody, clone BU1/75 (ICR1) Abcam Cat# ab6326,
Rat anti-CD68 Antibody, clone FA-11 Abcam Cat# ab53444,
Rat Anti-Myelin Basic Protein Monoclonal Antibody, Clone 12 Abcam Cat# ab7349,
Mouse anti-CD22, clone 2H1C4 Abcam Cat# ab181771,
Donkey anti-Rabbit IgG (H + L) Antibody, Alexa Fluor 488 Conjugated Thermo Fisher Scientific Cat# A-21206,
Donkey Anti-Rabbit IgG (H+L) Polyclonal Antibody, Alexa Fluor 555 Conjugated Thermo Fisher Scientific Cat# A-31572,
Goat anti-Rabbit IgG (H+L) Highly Cross-Adsorbed Secondary Antibody, Alexa Fluor™ Plus 647 Thermo Fisher Scientific Cat# A-32733,
Goat Anti-Rat IgG (H+L) Antibody, Alexa Fluor 488 Conjugated Thermo Fisher Scientific Cat# A-11006,
Goat anti-Mouse IgG (H+L) Cross-Adsorbed Secondary Antibody, Alexa Fluor™ 555 Thermo Fisher Scientific Cat# A-21422,
Rabbit anti-Bhlhe41 polyclonal Antibody Proteintech Cat# 12688-1-AP,
Rabbit IgG Sangon Biotech Cat# D110502,
Anti-Beta Actin Monoclonal antibody Proteintech Cat# 66009-1,
Dylight 800, Goat Anti-Mouse IgG Abbkine Cat# A23910,
Dylight 800, Goat Anti-Rabbit IgG Abbkine Cat# A23920,
mouse anti-APC (1:100, CC-1, Merck, Cat: OP80),
rabbit anti-degraded myelin basic protein antibody , Merck, Cat: AB5864
rabbit anti-Aurka polyclonal Antibody, Novus, Cat: NBP-1-51843
Anti-GAPDH,Sangon Biotech, Cat: D190090

Flow cytometric antibodies were 200x diluted for surface staining. For IF staining, antibodies dilutions are as below: mouse Anti-NeuN (1:100, Millipore, Cat: MAB377), mouse anti-Gfap (1:200, Biolegend, Cat: 644702), rabbit anti-Olig2 (1:500, Proteintech, Cat: 13999-1-AP), rabbit anti-Iba-1 (1:500, Wako, Cat: 019-19741), goat anti-dTomato (1:100, Biorbyt, Cat: orb182397), rabbit anti-RFP (1:200, Rockland, Cat: 600-401-379S), rat anti-CD206 (1:200, Bio-Rad, Cat: MCA2235), rat anti-BrdU (1:400, Abcam, Cat: ab6326), mouse anti-phospho-Histone H3(Ser10) (1:200, Cell Signaling Technology, Cat: 9706S), rat anti-CD68 (1:500, Abcam, Cat: ab53444,), mouse anti-CD22 (1:500, Abcam, Cat: ab181771),rat anti-Mbp (1:1000, Abcam, Cat: ab7349), mouse anti-APC (1:100, CC-1, Merck, Cat: OP80), rabbit anti-degraded myelin basic protein antibody (1:2000, Merck, Cat: AB5864) and rabbit anti-Olig2 (1:500, Proteintech, Cat: 13999-1-AP), as well as matched secondary antibodies: matched AF488 (1:500, ThermoFisher Scientific, Cat: A-21206/A-11006), AF555 (1:500, ThermoFisher Scientific, Cat: A-31572/A-21422), AF647 (1:500, ThermoFisher Scientific, Cat: A-32733)-conjugated secondary antibody. For WB analysis, antibodies were diluted as below: rabbit anti-Bhlhe41 polyclonal Antibody (1:1000, Proteintech, Cat: 12688-1-AP), anti-β-actin (1:50000, Proteintech, Cat: 66009-1), rabbit anti-Aurka polyclonal Antibody

(1:1000, Novus, Cat: NBP-1-51843) and Anti-GAPDH (1:5000, Sangon Biotech, Cat: D190090), Goat Anti-Mouse/Rabbit IgG Dylight 800 (1:10000, Abbkin, Cat: A23910/A23920).

Validation	All antibodies used in this study were commercially sourced and validated by the manufacturers. Detailed validation data are available on the manufacturer's official websites using the catalog numbers provided.

Animals and other research organisms

Policy information about studies involving animals; ARRIVE guidelines recommended for reporting animal research, and Sex and Gender in Research

Laboratory animals	Cx3cr1Cre mice (Stock ID: 025524, JAX) and Cx3cr1CreER mice (Stock ID: 021160, JAX) were obtained from the Jackson Laboratory. Bhlhe41dTomato-Cre/+ (referred to as B41HET), Bhlhe41fl/fl, Aurkafl/fl, Aurkbfl/fl, and Rosa26Bhlhe41-EYFP/+ (referred to as R26B41-EYFP/+) mice were generated by Cyagen Biosciences Inc. All mice were maintained on the C57BL/6 genetic background. Age of mice used in each experiment is specified in the figure legends.
Wild animals	The study did not involve wild animals.
Reporting on sex	Sex-matched mice of different genotypes were generally used across experiments, except that cuprizone-induced demyelination experiments and in vivo phagocytosis assays were performed exclusively in male mice. Sex-disaggregated analyses were not performed, and sex-disaggregated sample numbers are not available for all experiments, as the study was not designed or powered to assess sex-specific effects.
Field-collected samples	The study did not involve samples collected from the field.
Ethics oversight	All mouse experiments were performed in accordance with the guidelines and regulations of Xuzhou Medical University and were approved by the Animal Experimental Ethics Committee of Xuzhou Medical University (Approval no: 202211S011).

Note that full information on the approval of the study protocol must also be provided in the manuscript.

Plants

Seed stocks	N/A
Novel plant genotypes	N/A
Authentication	N/A

Flow Cytometry

Plots

Confirm that:

☒ The axis labels state the marker and fluorochrome used (e.g. CD4-FITC).

☒ The axis scales are clearly visible. Include numbers along axes only for bottom left plot of group (a 'group' is an analysis of identical markers).

☒ All plots are contour plots with outliers or pseudocolor plots.

☒ A numerical value for number of cells or percentage (with statistics) is provided.

Methodology

Sample preparation	Prior to flow cytometric analysis, cell suspensions were made from yolk sac, brain, spinal cord, bronchoalveolar lavage fluid (BALF) and peritoneal cavity. Briefly, brains from embryos, newborn and adult mice, and spinal cord were minced and homogenized by gentle Dounce homogenization, followed by suspension in 40% Percoll and centrifugation at 800 g for 30 minutes at room temperature. Yolk sac from embryos were minced and incubated with 0.5 mg/mL Collagenase IV (ThermoFisher Scientific, Cat: 17104019) in RPMI 1640 medium with 1% FBS for one hour. BALF were collected by inserting a canula into the trachea following opening the chest cavity. 1 mL of pre-chilled PBS with 2 mM EDTA was used to flush the lung three times. Cell pellets were collected and washed using PBS with 1% FBS and 2 mM EDTA, and filtered through 70 µm mesh prior to labeling for flow cytometric analysis.
Instrument	BD FACS Aria III

Software	BD FACSDiva v9 and Flowjo v 10.8.1 were used to collect and analyze the flow cytometry data, respectively.
Cell population abundance	Microglia were purified by flow cytometric sorting of FVD-CD45loCx3cr1+CD11b+ cells from 40% percoll-enriched cells from the brains of young B41HET and B41KO mice (10 - 15 mice per sample). About 1-2 millions microglia per sample with a purity > 90% were used for RNA extraction.
Gating strategy	Cells were gated by FSC-A/SSC-A and/or CD45/SSC-A followed by gating on singlets (FSC-A/FSC-H). Live cells (FVD-) were gated prior to further analysis.

☒ Tick this box to confirm that a figure exemplifying the gating strategy is provided in the Supplementary Information.
