# Supplementary material for: Aurka-Bhlhe41 axis prevents premature aging-like microglial dysfunction and promotes remyelination
Source: Nat Commun. 2026 Mar 27;17:5238. doi: 10.1038/s41467-026-71014-w (PMC13260908; doi:10.1038/s41467-026-71014-w)
Supplement: Supplementary file 6 — Source Data [file 41467_2026_71014_MOESM6_ESM.zip › Source Data/Statistical Report/Diagnosis/Fig4f_LMM_Residuals_QQPlot.pdf]

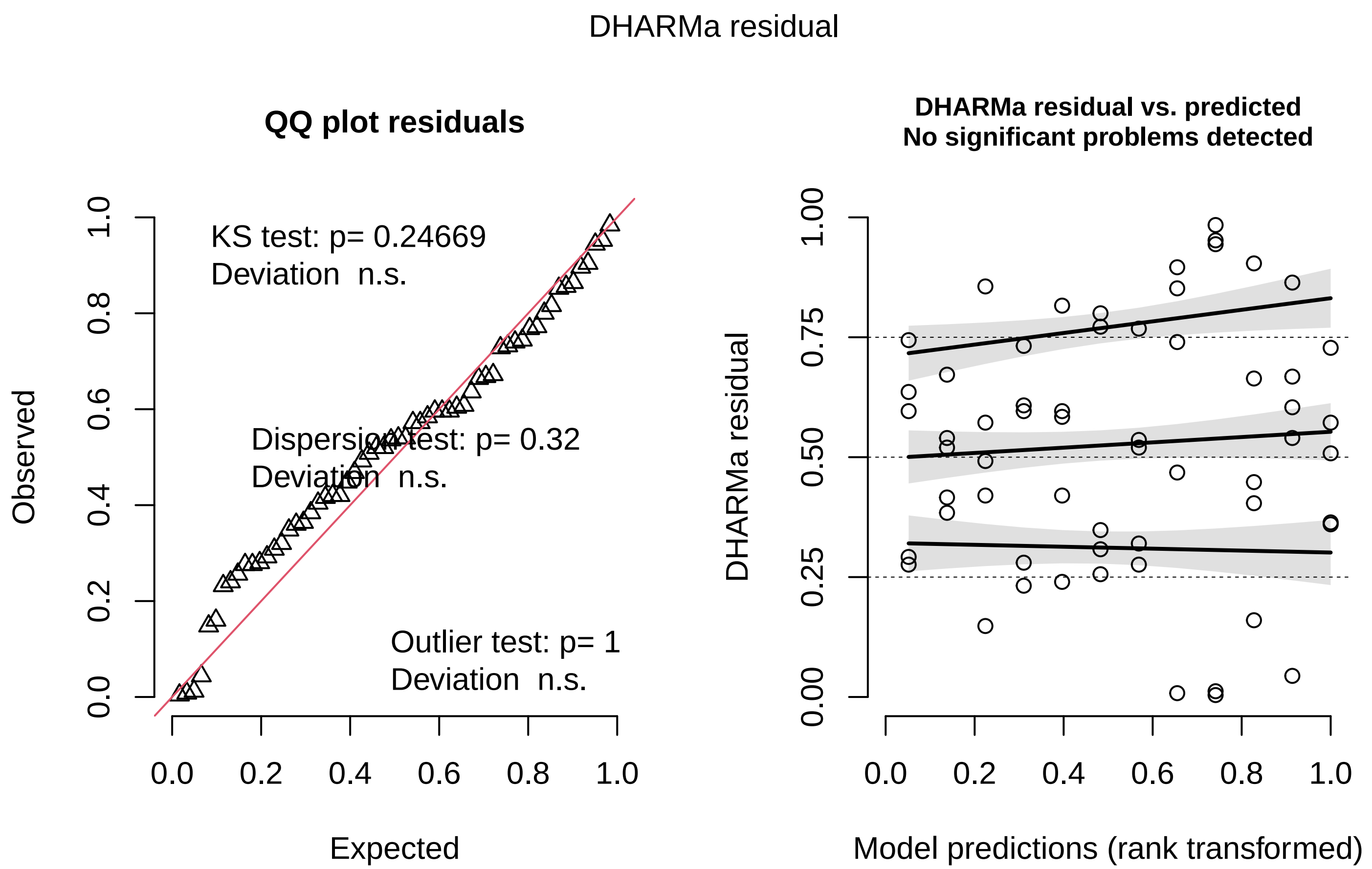
DHARMa residual
QQ plot residuals
KS test: p= 0.24669
Deviation n.s.
Dispersion test: p= 0.32
Deviation n.s.
Outlier test: p= 1
Deviation n.s.
Observed
Expected
0.0
0.2
0.4
0.6
0.8
1.0
DHARMa residual vs. predicted
No significant problems detected
DHARMa residual
Model predictions (rank transformed)
0.00
0.25
0.50
0.75
1.00
