# Supplementary figures and images for: Aurka-Bhlhe41 axis prevents premature aging-like microglial dysfunction and promotes remyelination
Source: Nat Commun. 2026 Mar 27;17:5238. doi: 10.1038/s41467-026-71014-w (PMC13260908; doi:10.1038/s41467-026-71014-w)

Normal Q-Q

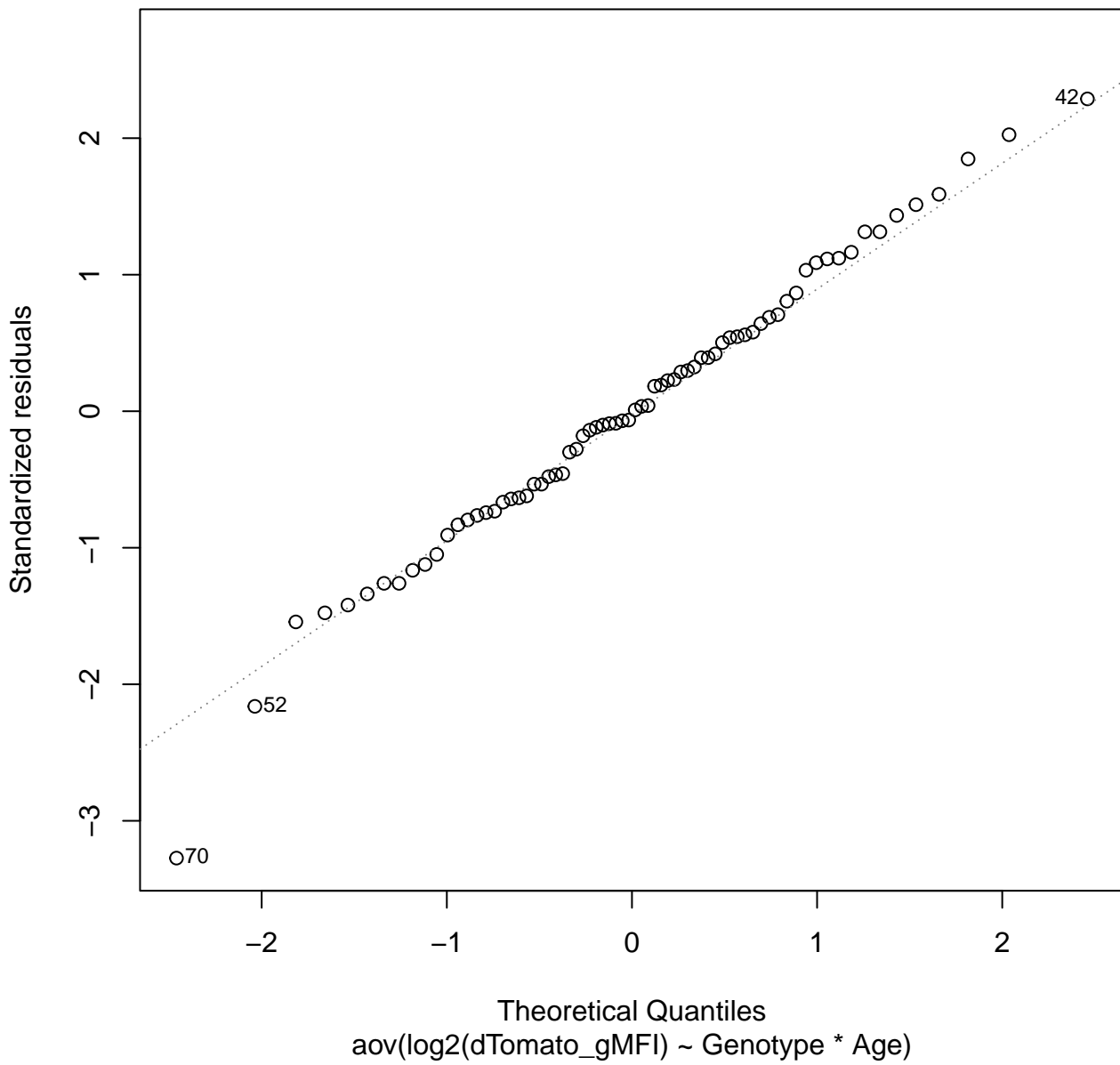

Supplement: Supplementary file 6 — Source Data [file 41467_2026_71014_MOESM6_ESM.zip › Source Data/Statistical Report/Diagnosis/Fig1a_Residuals_QQPlot.pdf]

Q-Q Plot

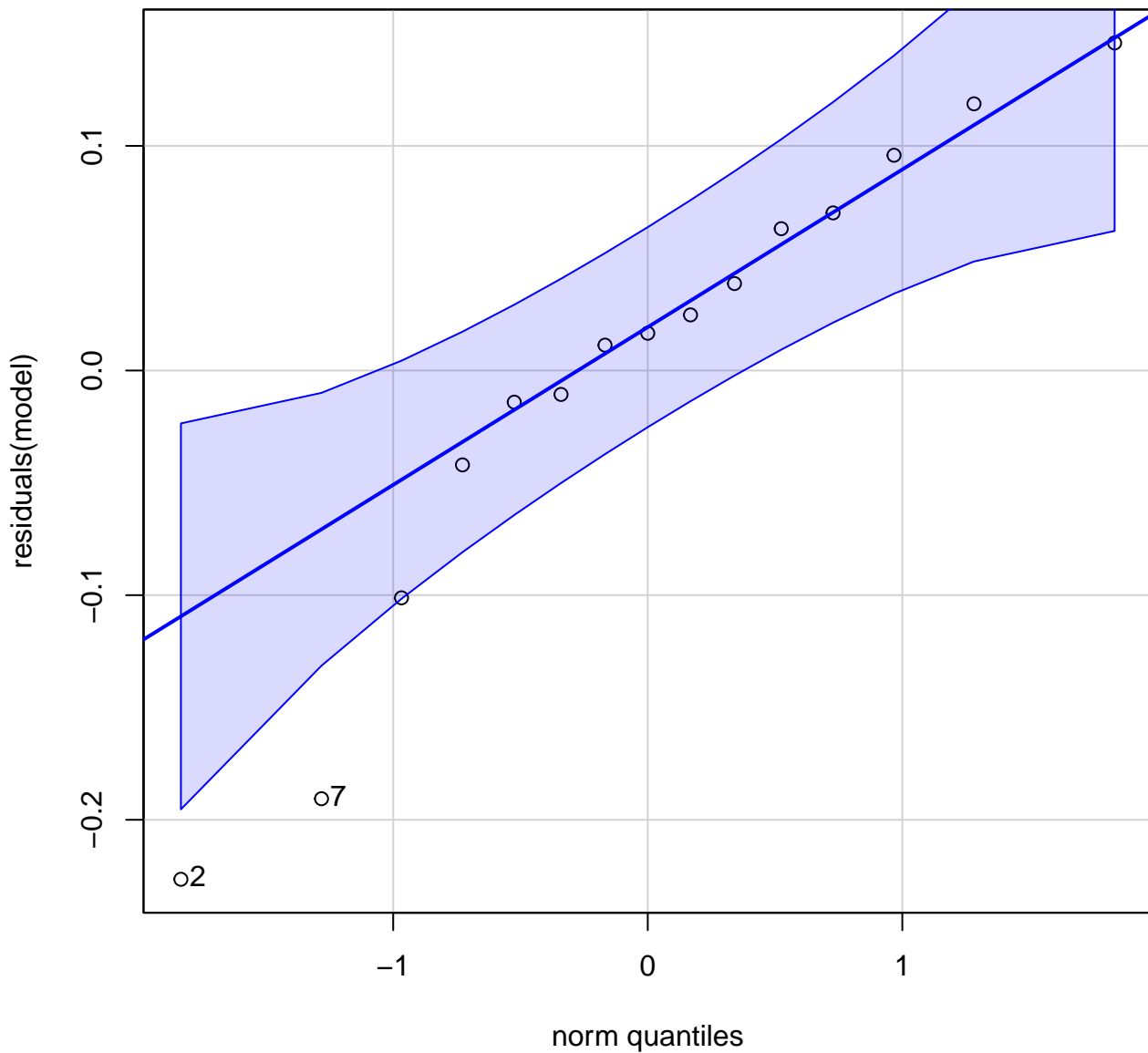

Supplement: Supplementary file 6 — Source Data [file 41467_2026_71014_MOESM6_ESM.zip › Source Data/Statistical Report/Diagnosis/Fig1b_Residuals_QQPlot.pdf]

Q-Q Plot

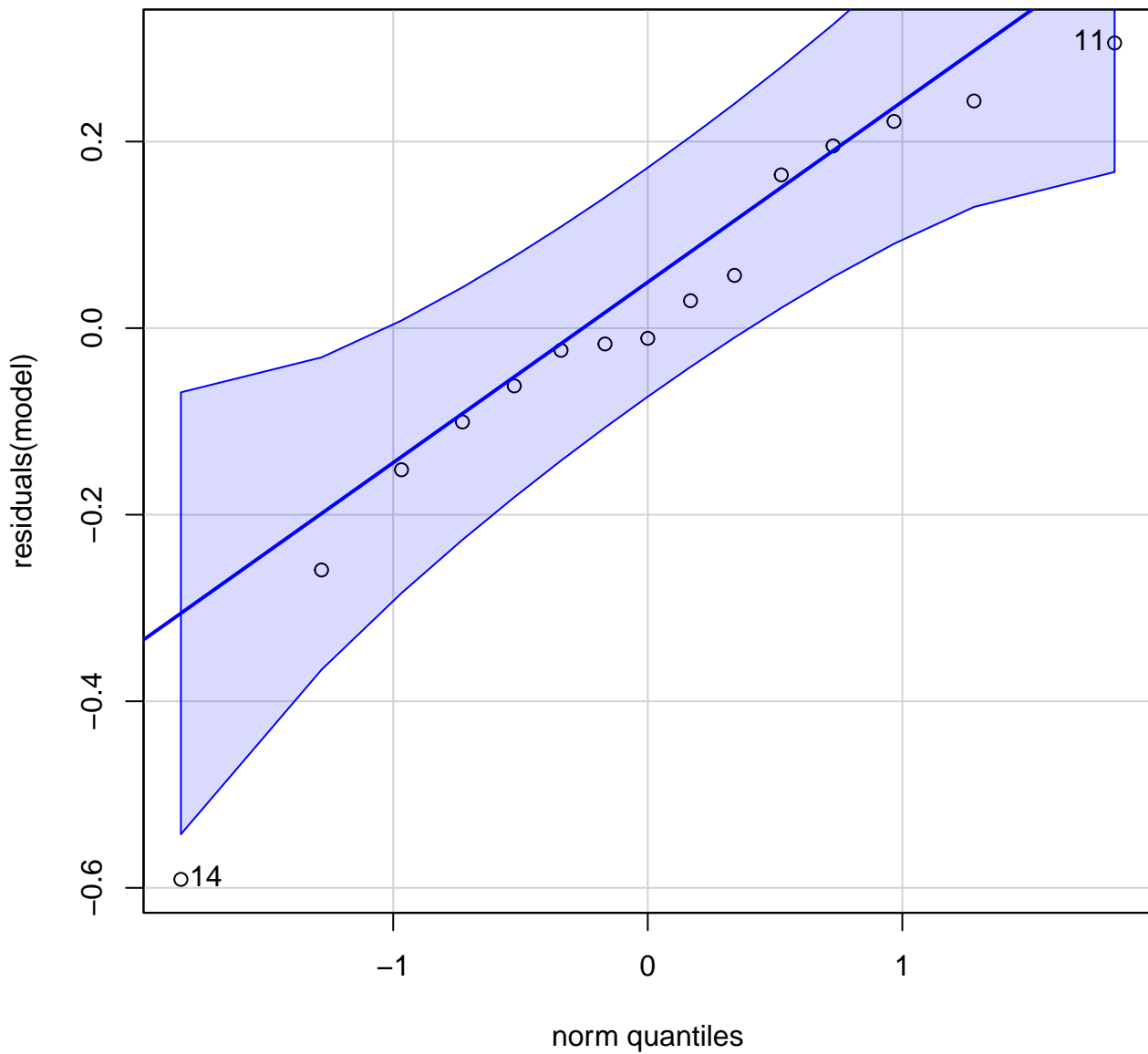

Supplement: Supplementary file 6 — Source Data [file 41467_2026_71014_MOESM6_ESM.zip › Source Data/Statistical Report/Diagnosis/Fig2c_Residuals_QQPlot.pdf]

**Q-Q Plot of gMFI**

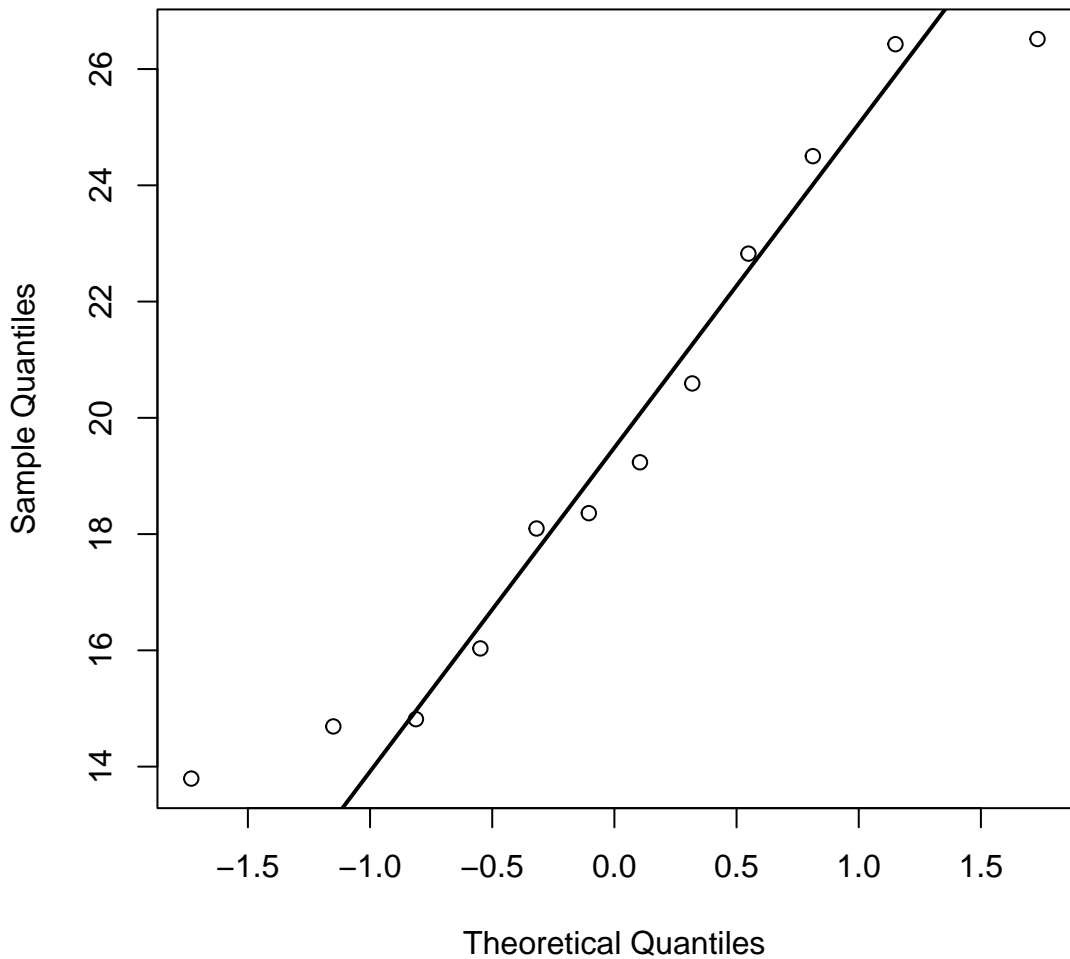

Supplement: Supplementary file 6 — Source Data [file 41467_2026_71014_MOESM6_ESM.zip › Source Data/Statistical Report/Diagnosis/Fig2d_QQ_Plot_gMFI.pdf]

**Q-Q Plot of gMFI**

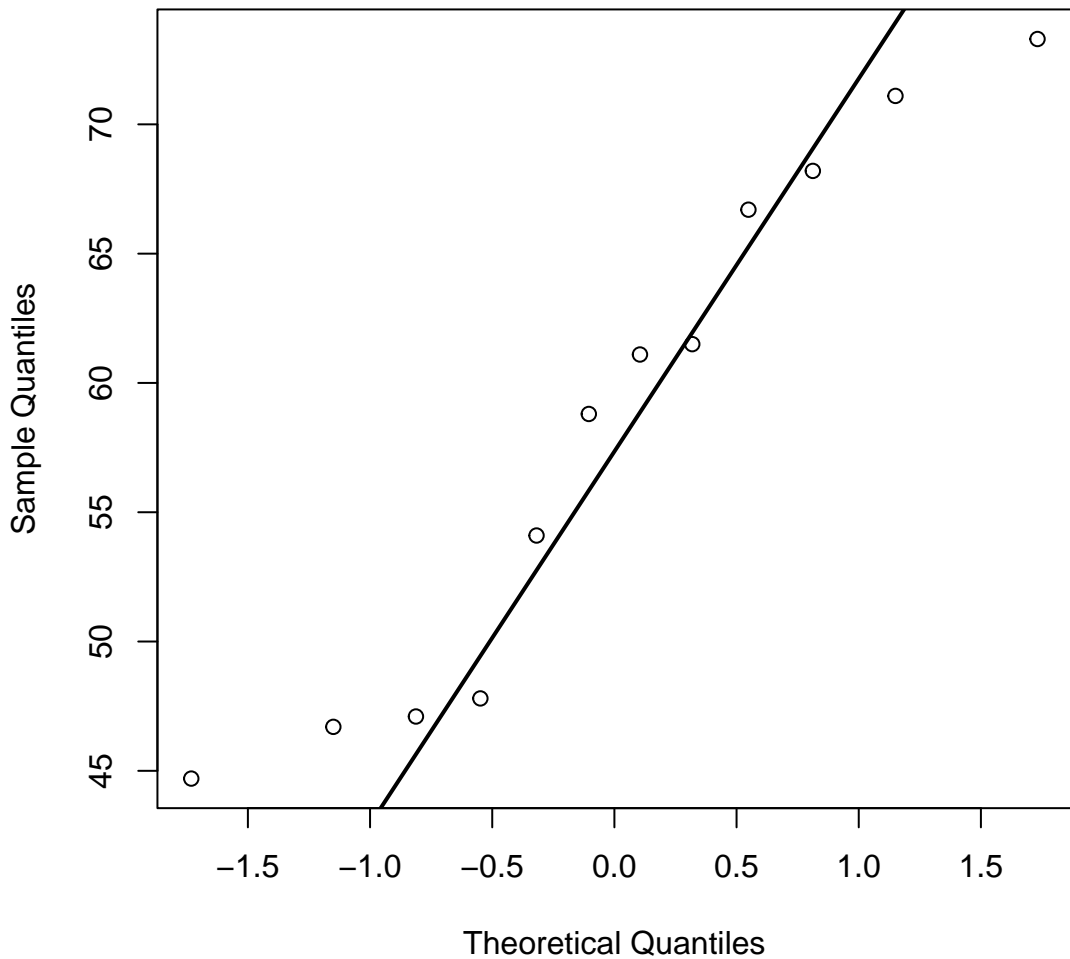

Supplement: Supplementary file 6 — Source Data [file 41467_2026_71014_MOESM6_ESM.zip › Source Data/Statistical Report/Diagnosis/Fig2d_QQ_Plot_Ratio.pdf]

**Q-Q Plot of AF488\_Pos**

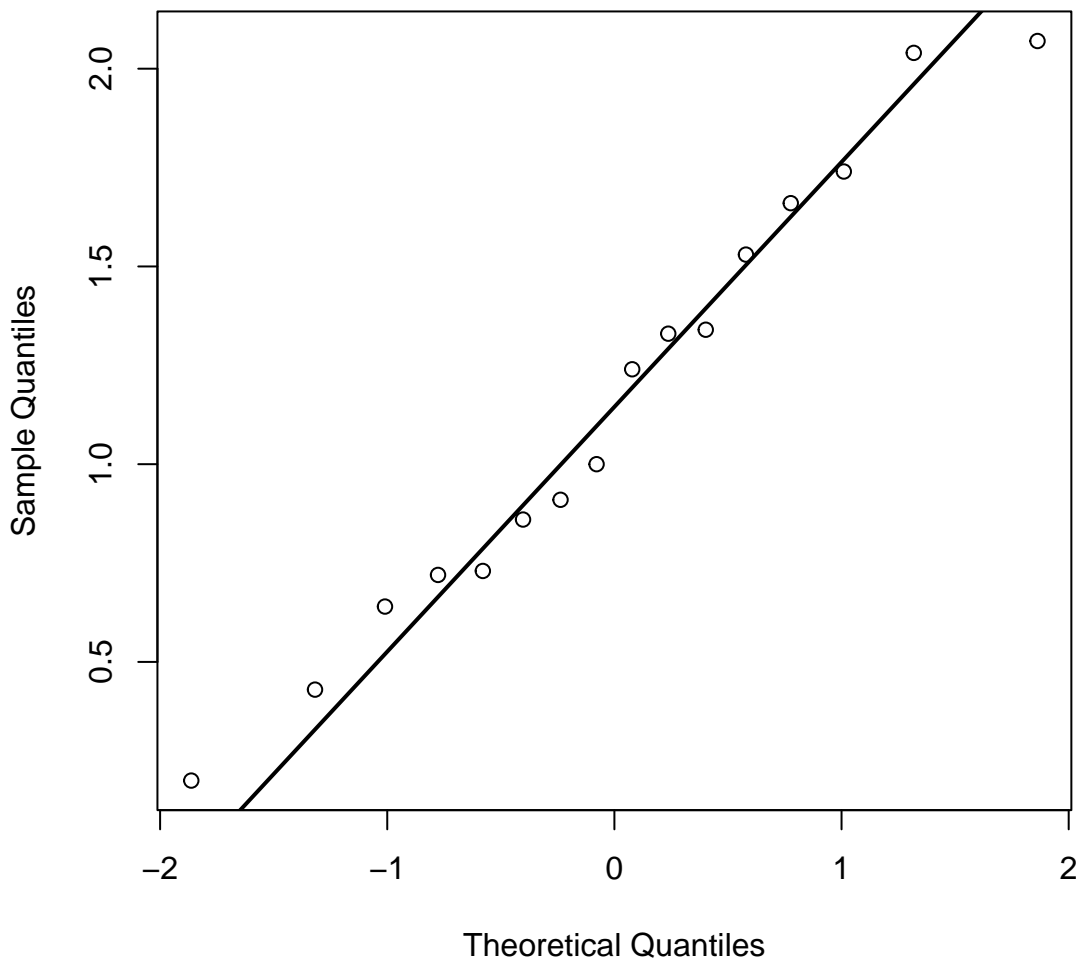

Supplement: Supplementary file 6 — Source Data [file 41467_2026_71014_MOESM6_ESM.zip › Source Data/Statistical Report/Diagnosis/Fig2f_QQ_Plot.pdf]

**Q-Q Plot of AF488\_MFI**

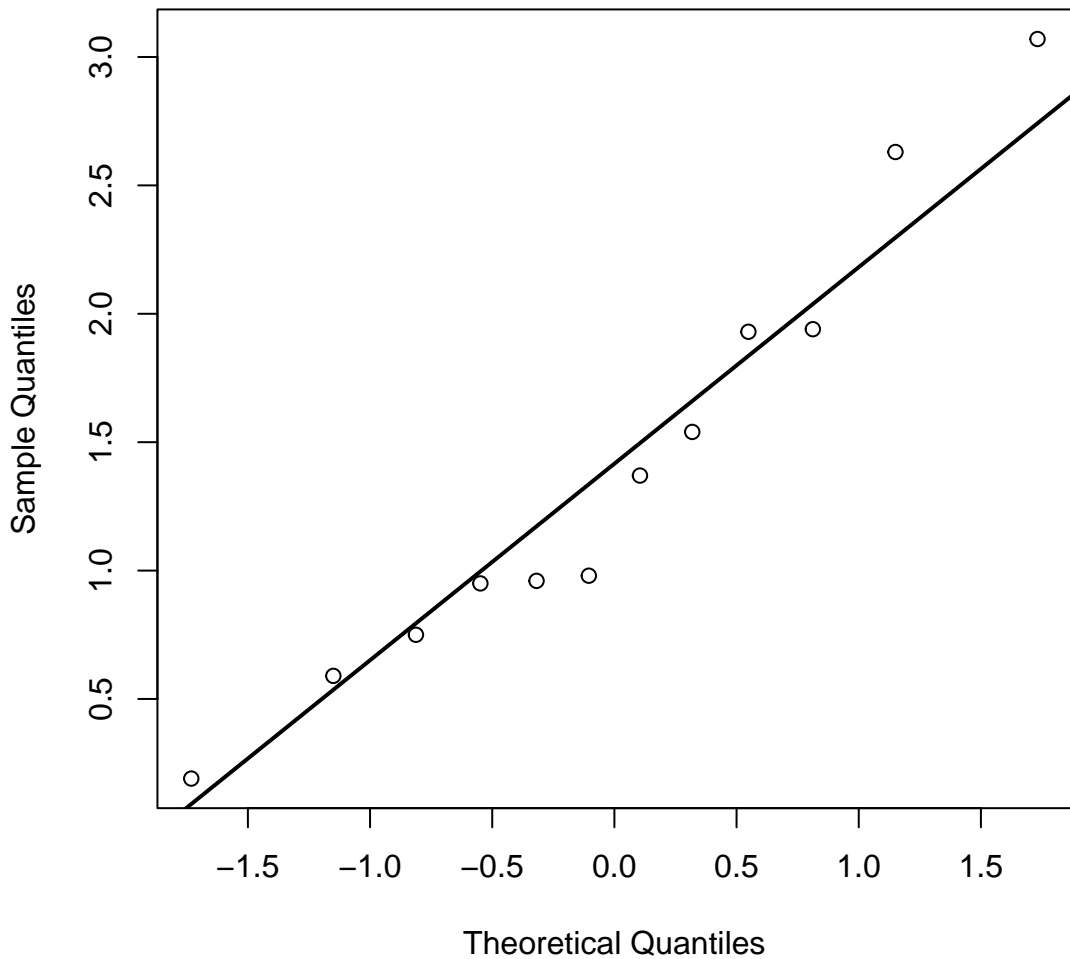

Supplement: Supplementary file 6 — Source Data [file 41467_2026_71014_MOESM6_ESM.zip › Source Data/Statistical Report/Diagnosis/Fig2g_QQ_Plot.pdf]

**Q-Q Plot of AF488\_Pos**

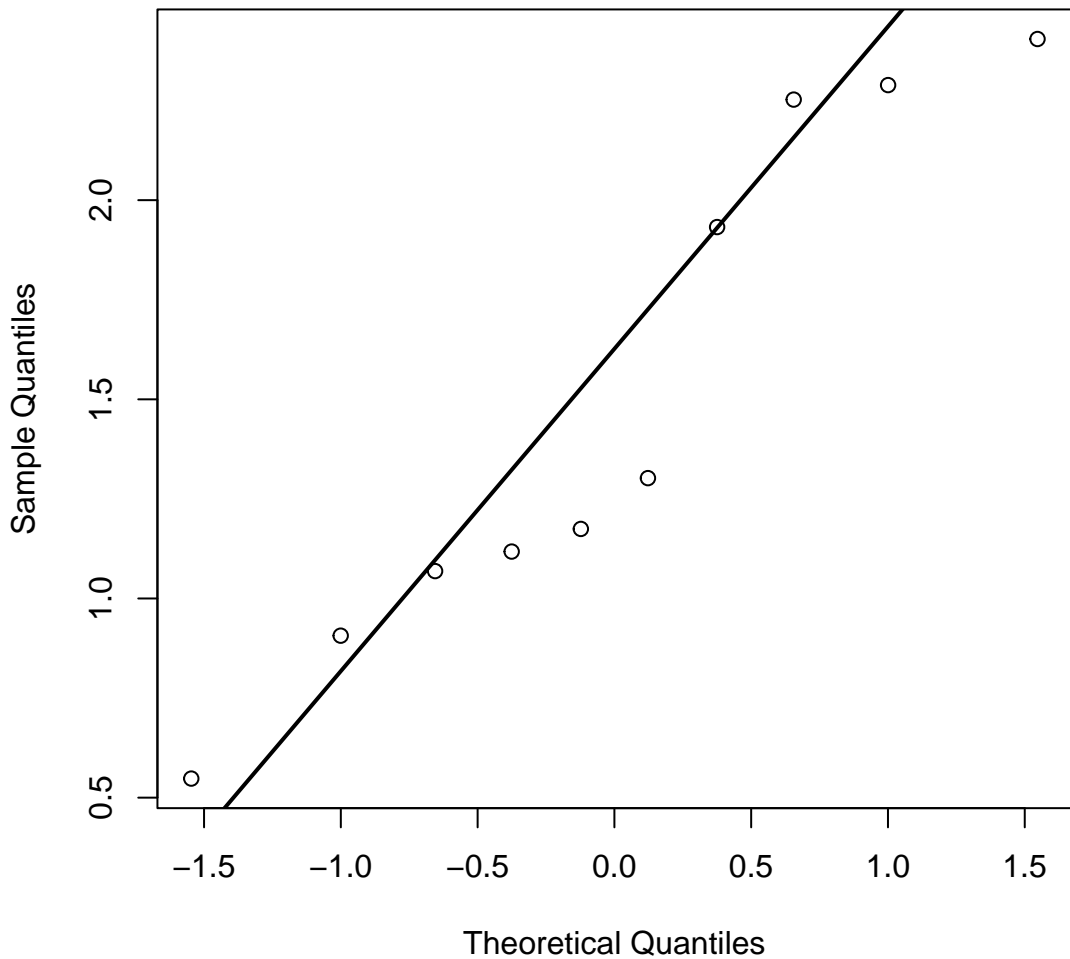

Supplement: Supplementary file 6 — Source Data [file 41467_2026_71014_MOESM6_ESM.zip › Source Data/Statistical Report/Diagnosis/Fig2h_QQ_Plot.pdf]

**Q-Q Plot of AF488\_Pos**

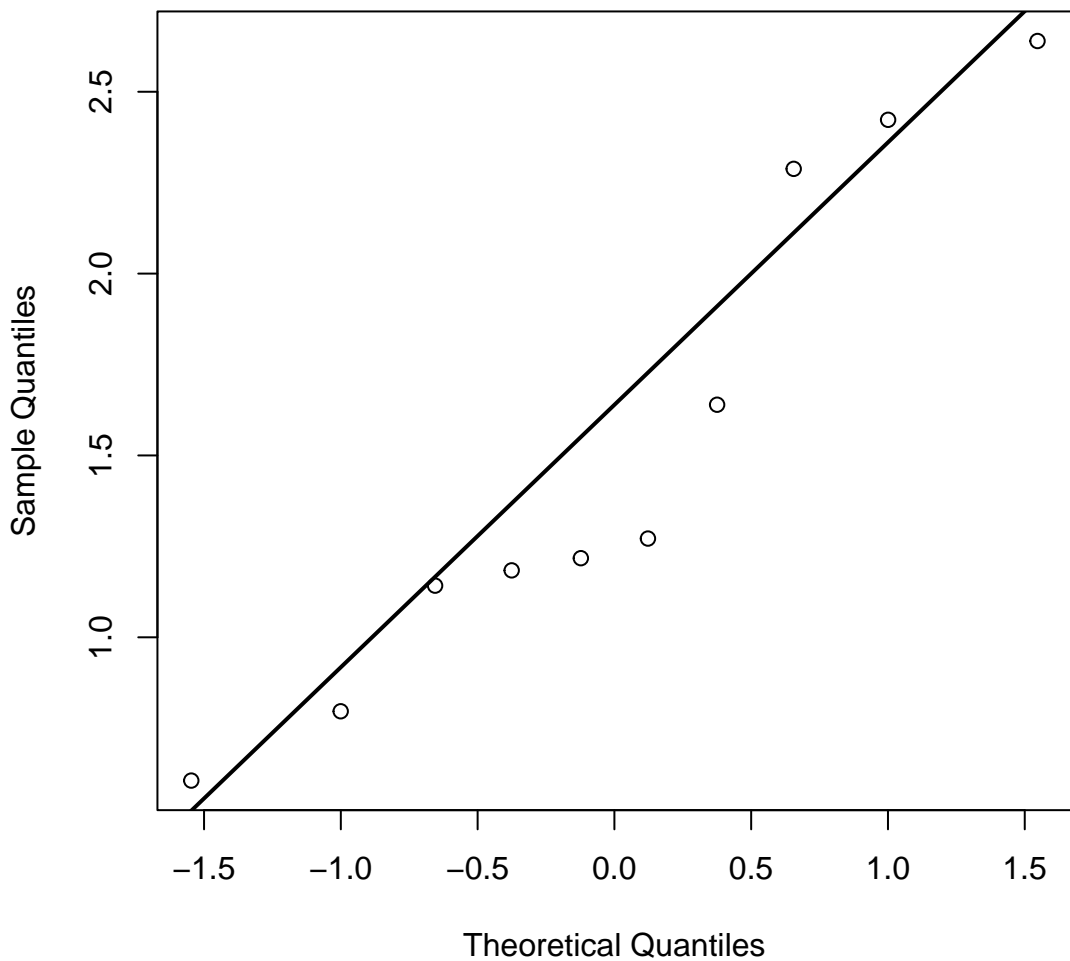

Supplement: Supplementary file 6 — Source Data [file 41467_2026_71014_MOESM6_ESM.zip › Source Data/Statistical Report/Diagnosis/Fig2i_QQ_Plot.pdf]

**Q-Q Plot of Residuals for Fig3b**

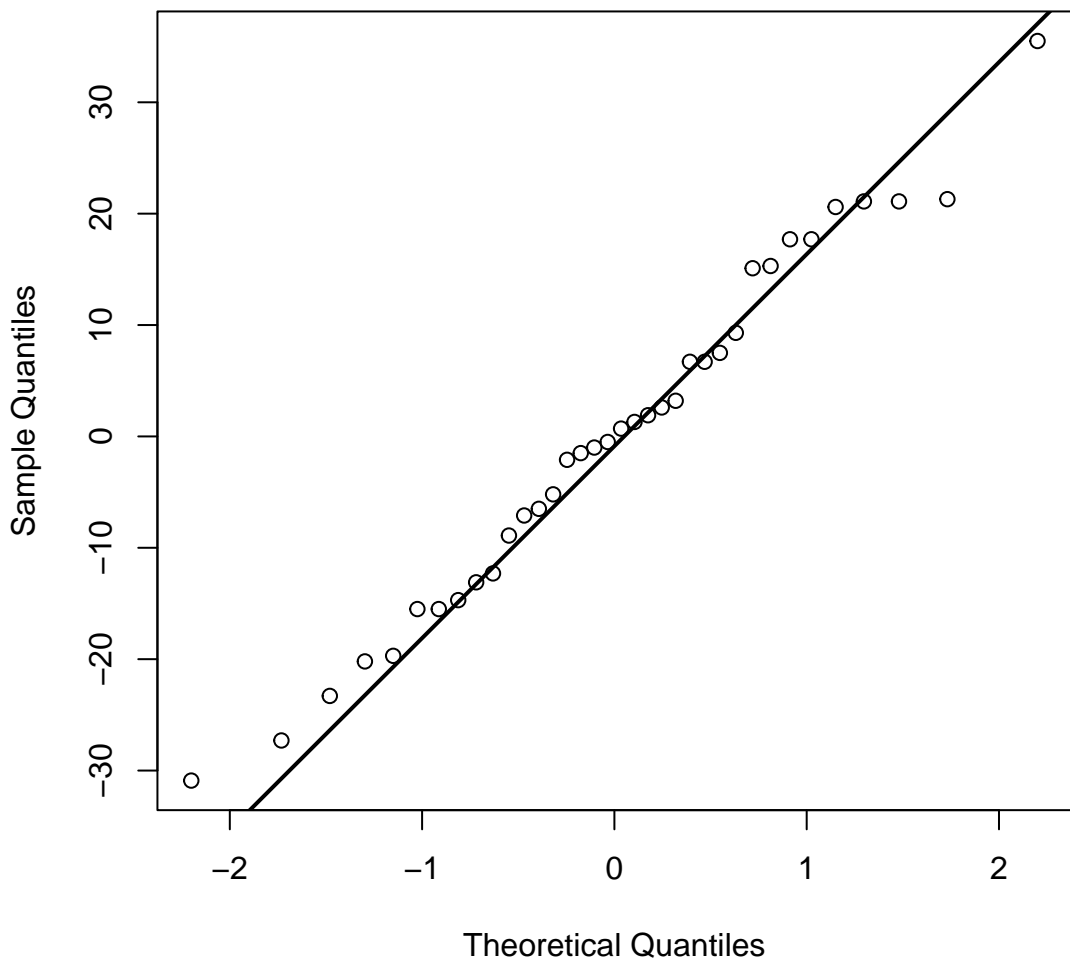

Supplement: Supplementary file 6 — Source Data [file 41467_2026_71014_MOESM6_ESM.zip › Source Data/Statistical Report/Diagnosis/Fig3b_Residuals_QQ_Plot.pdf]

**Q-Q Plot of Residuals for Fig3c**

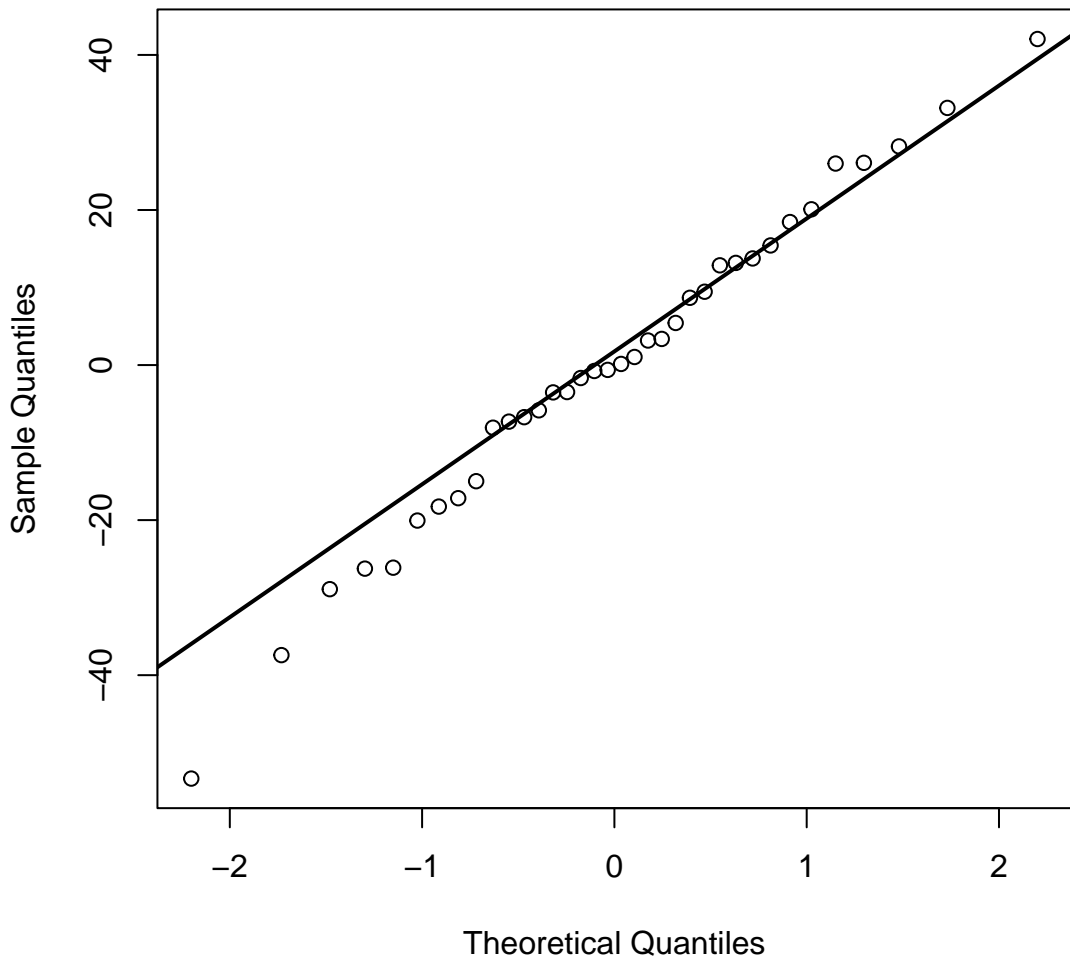

Supplement: Supplementary file 6 — Source Data [file 41467_2026_71014_MOESM6_ESM.zip › Source Data/Statistical Report/Diagnosis/Fig3c_Residuals_QQ_Plot.pdf]

**Q-Q Plot of Residuals for Fig3g\_dMbp\_lba1**

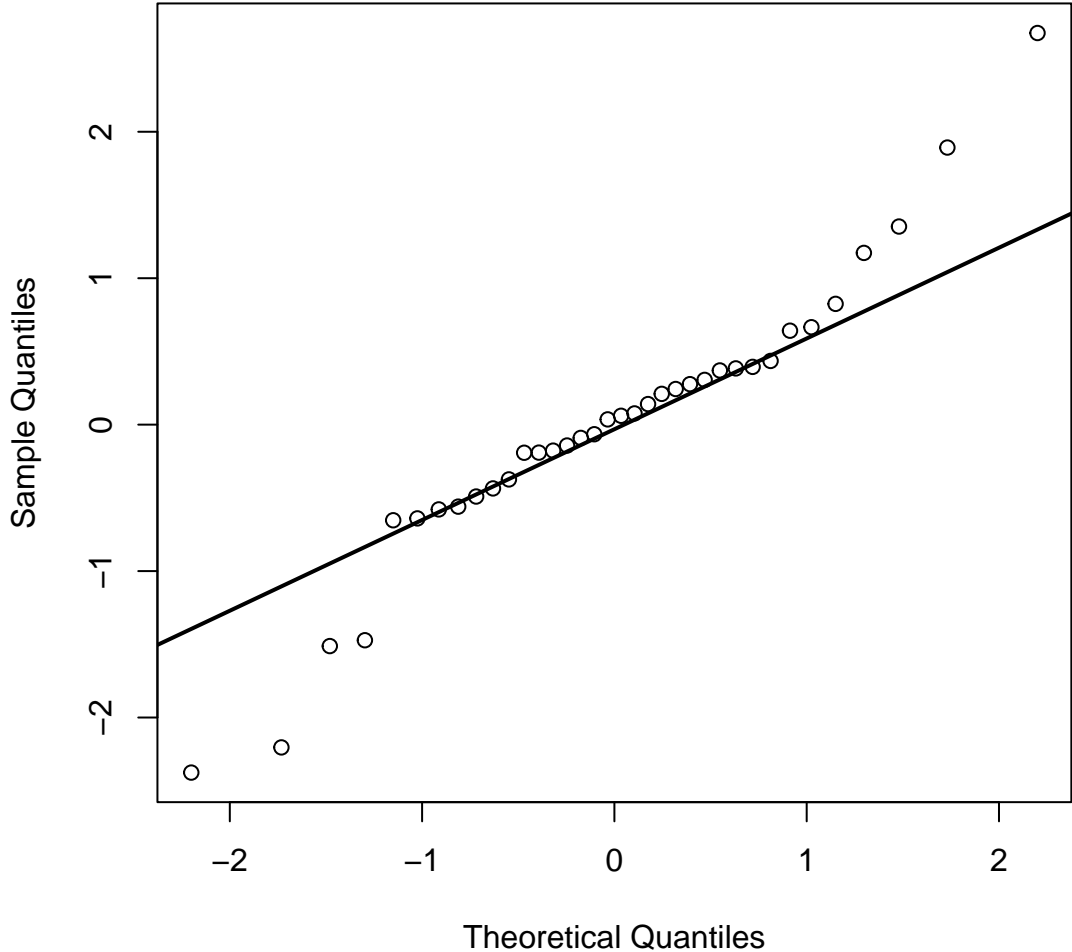

Supplement: Supplementary file 6 — Source Data [file 41467_2026_71014_MOESM6_ESM.zip › Source Data/Statistical Report/Diagnosis/Fig3g_dMbp_Iba1_Residuals_QQ_Plot.pdf]

Q-Q Plot

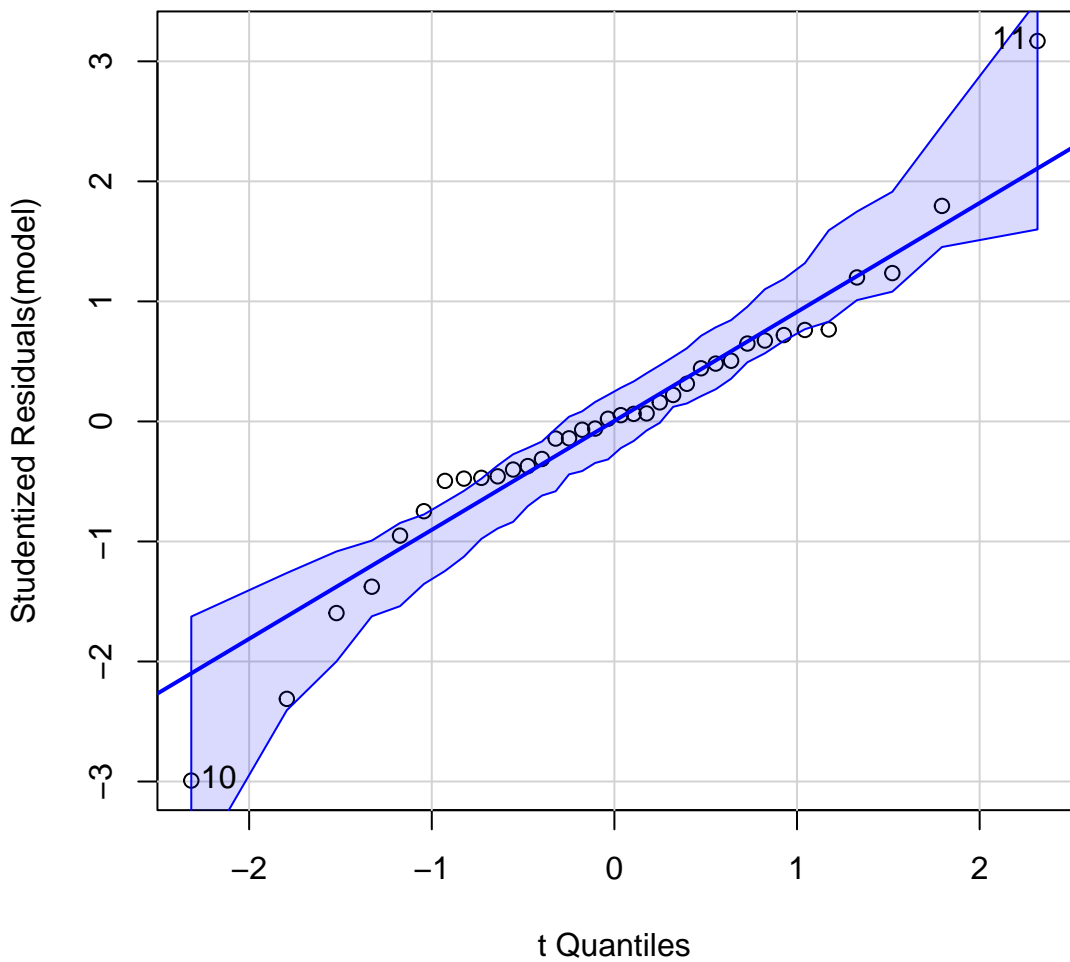

Supplement: Supplementary file 6 — Source Data [file 41467_2026_71014_MOESM6_ESM.zip › Source Data/Statistical Report/Diagnosis/Fig3g_dMbp_Residuals_QQ_Plot.pdf]

**Q-Q Plot of AF488\_Pos**

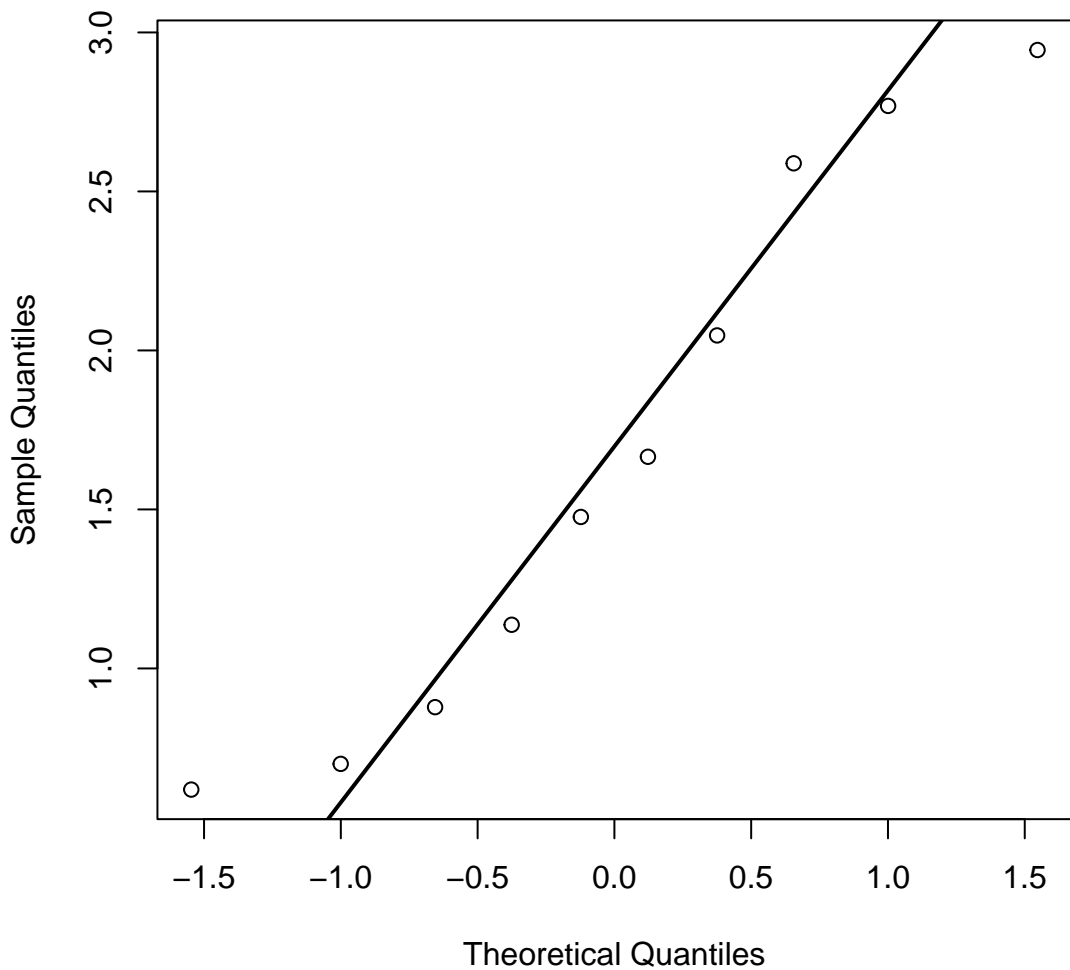

Supplement: Supplementary file 6 — Source Data [file 41467_2026_71014_MOESM6_ESM.zip › Source Data/Statistical Report/Diagnosis/Fig4d_QQ_Plot.pdf]

Normal Q-Q

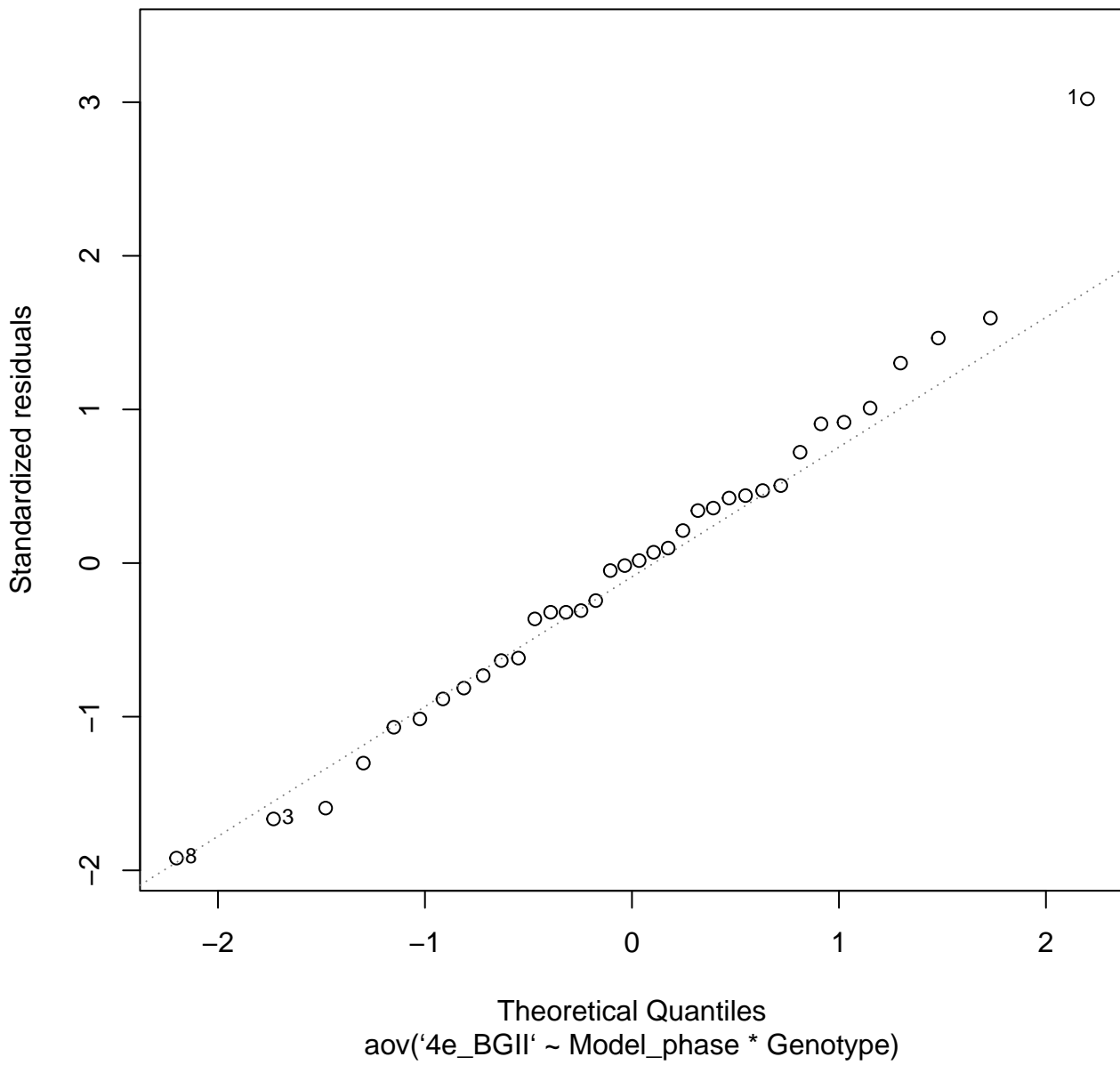

Supplement: Supplementary file 6 — Source Data [file 41467_2026_71014_MOESM6_ESM.zip › Source Data/Statistical Report/Diagnosis/Fig4e_Residuals_QQPlot.pdf]

Normal Q-Q

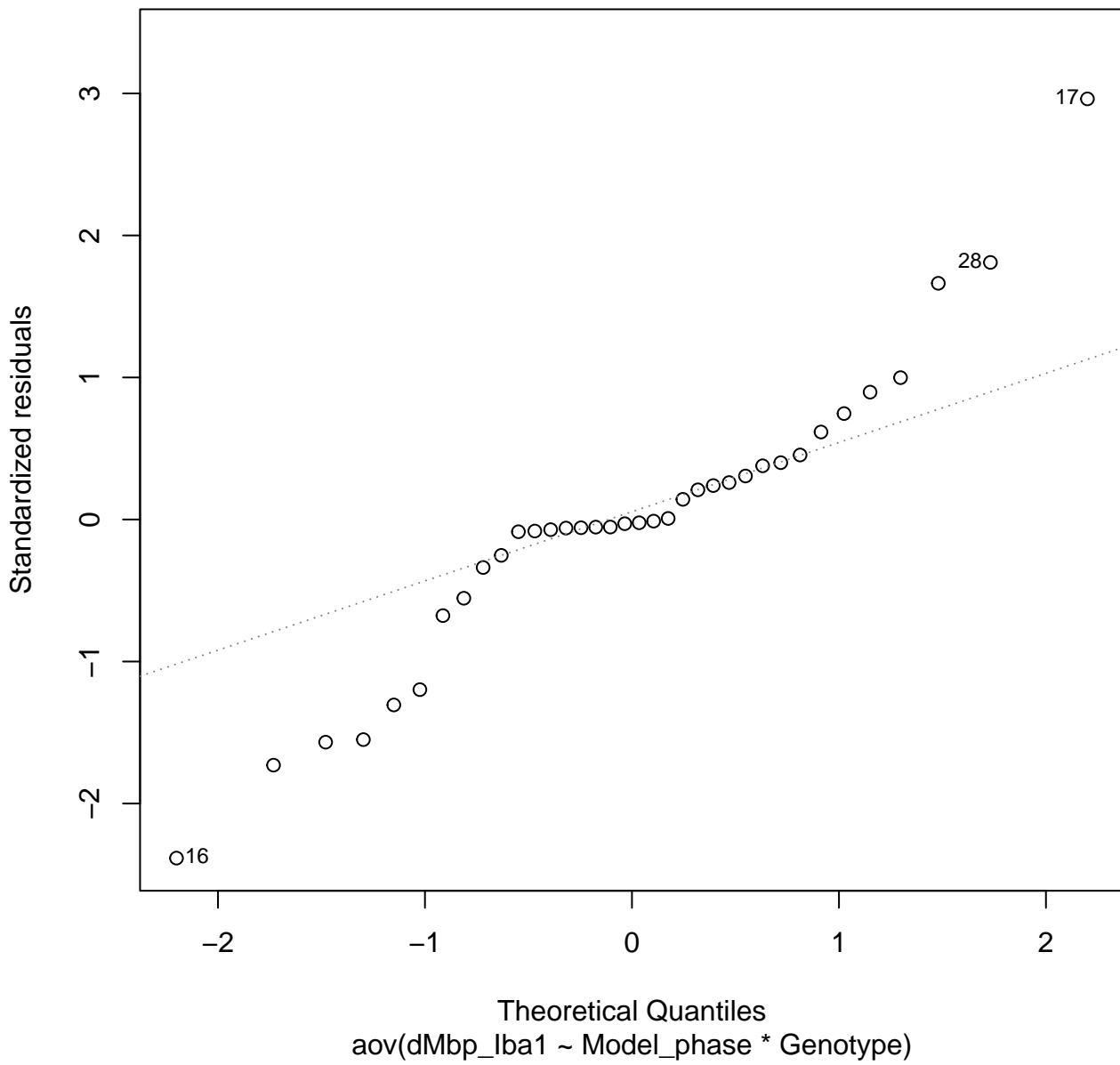

Supplement: Supplementary file 6 — Source Data [file 41467_2026_71014_MOESM6_ESM.zip › Source Data/Statistical Report/Diagnosis/Fig4h_dMbp_Iba1_Residuals_QQPlot.pdf]

Normal Q-Q

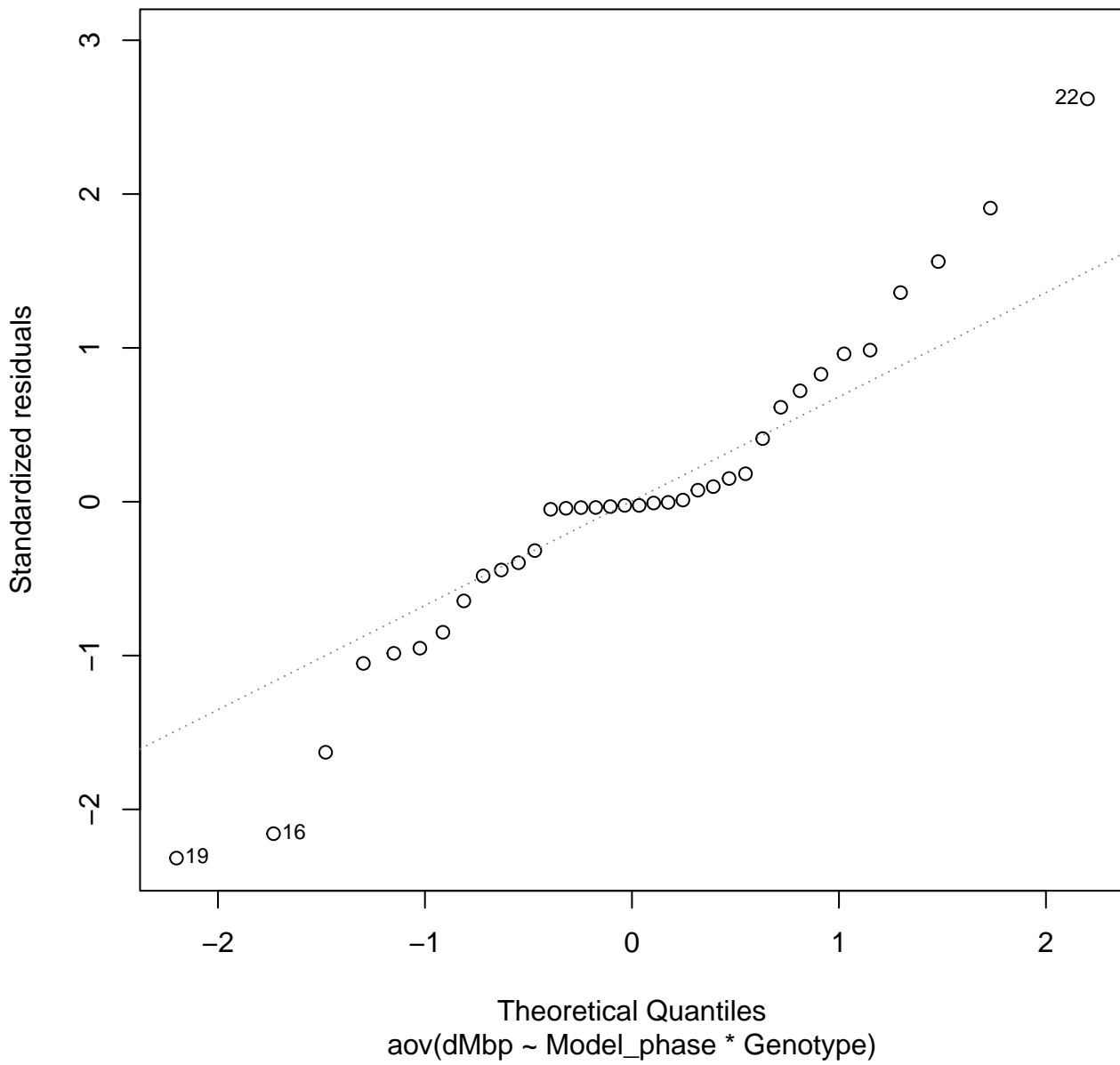

Supplement: Supplementary file 6 — Source Data [file 41467_2026_71014_MOESM6_ESM.zip › Source Data/Statistical Report/Diagnosis/Fig4h_Residuals_QQPlot.pdf]

Q-Q Plot

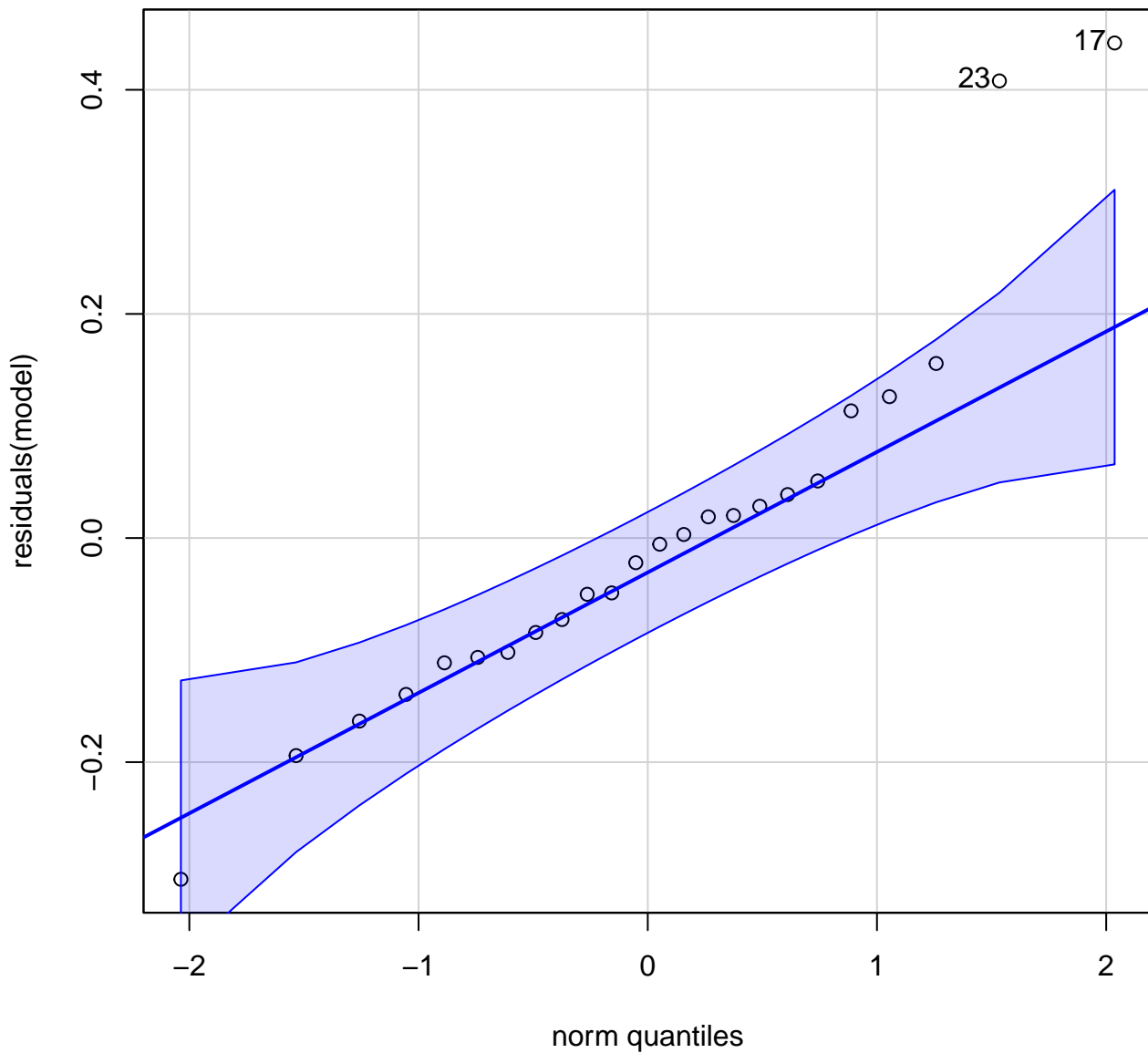

Supplement: Supplementary file 6 — Source Data [file 41467_2026_71014_MOESM6_ESM.zip › Source Data/Statistical Report/Diagnosis/Fig5b_Residuals_QQPlot.pdf]

**Q-Q Plot of AF488\_Pos**

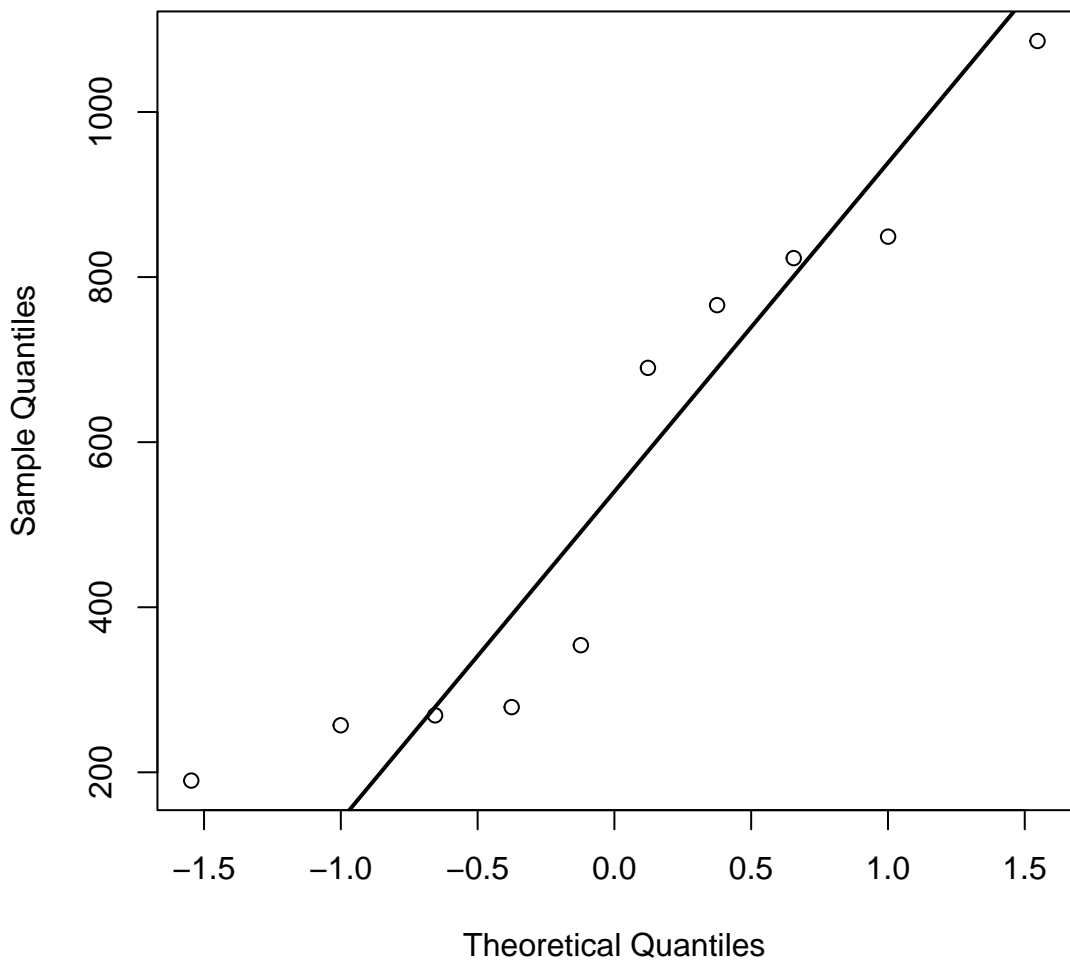

Supplement: Supplementary file 6 — Source Data [file 41467_2026_71014_MOESM6_ESM.zip › Source Data/Statistical Report/Diagnosis/Fig5d_QQ_Plot.pdf]

Q-Q Plot

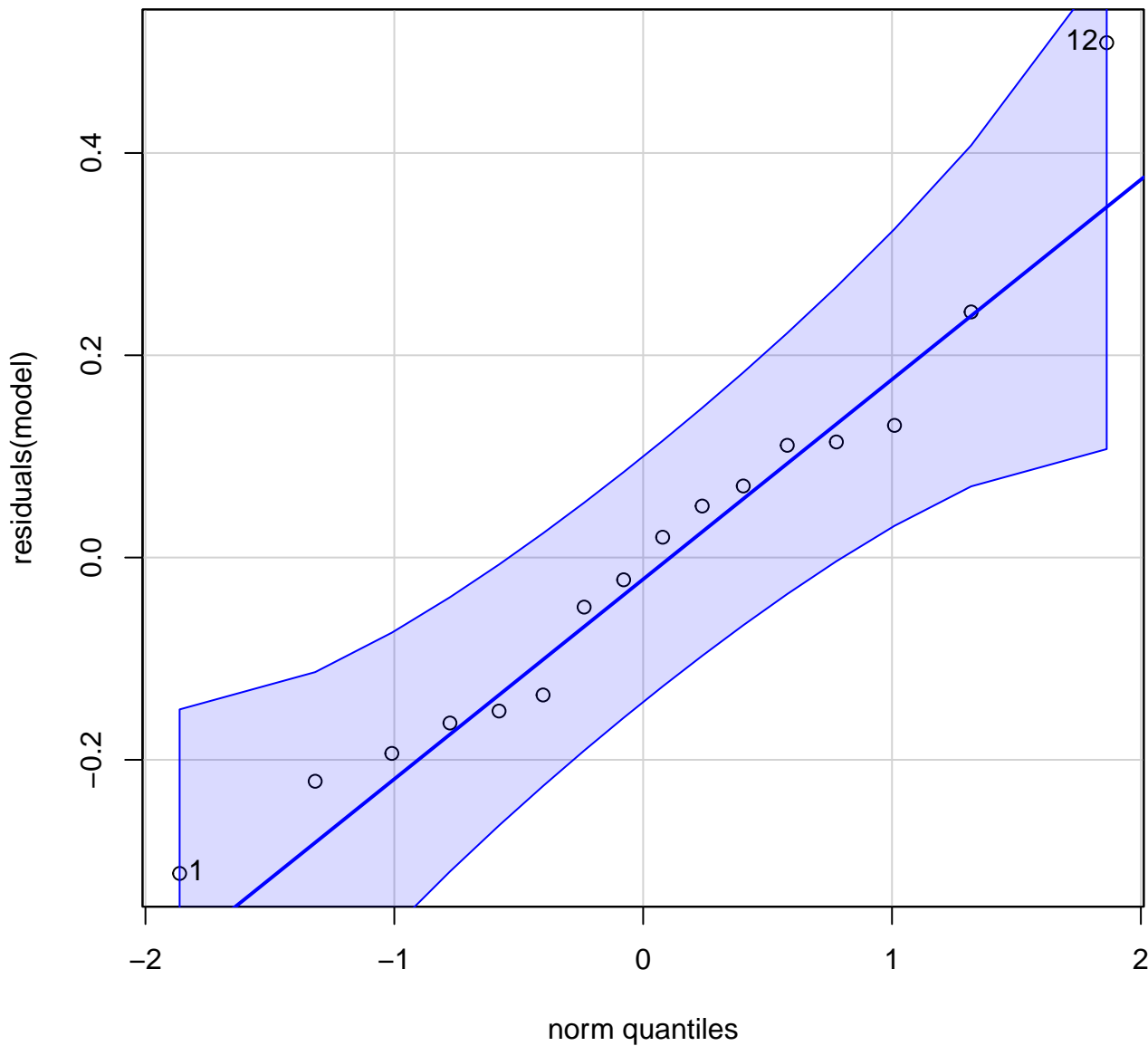

Supplement: Supplementary file 6 — Source Data [file 41467_2026_71014_MOESM6_ESM.zip › Source Data/Statistical Report/Diagnosis/Fig5f_Residuals_QQPlot.pdf]

**Normal Q-Q Plot**

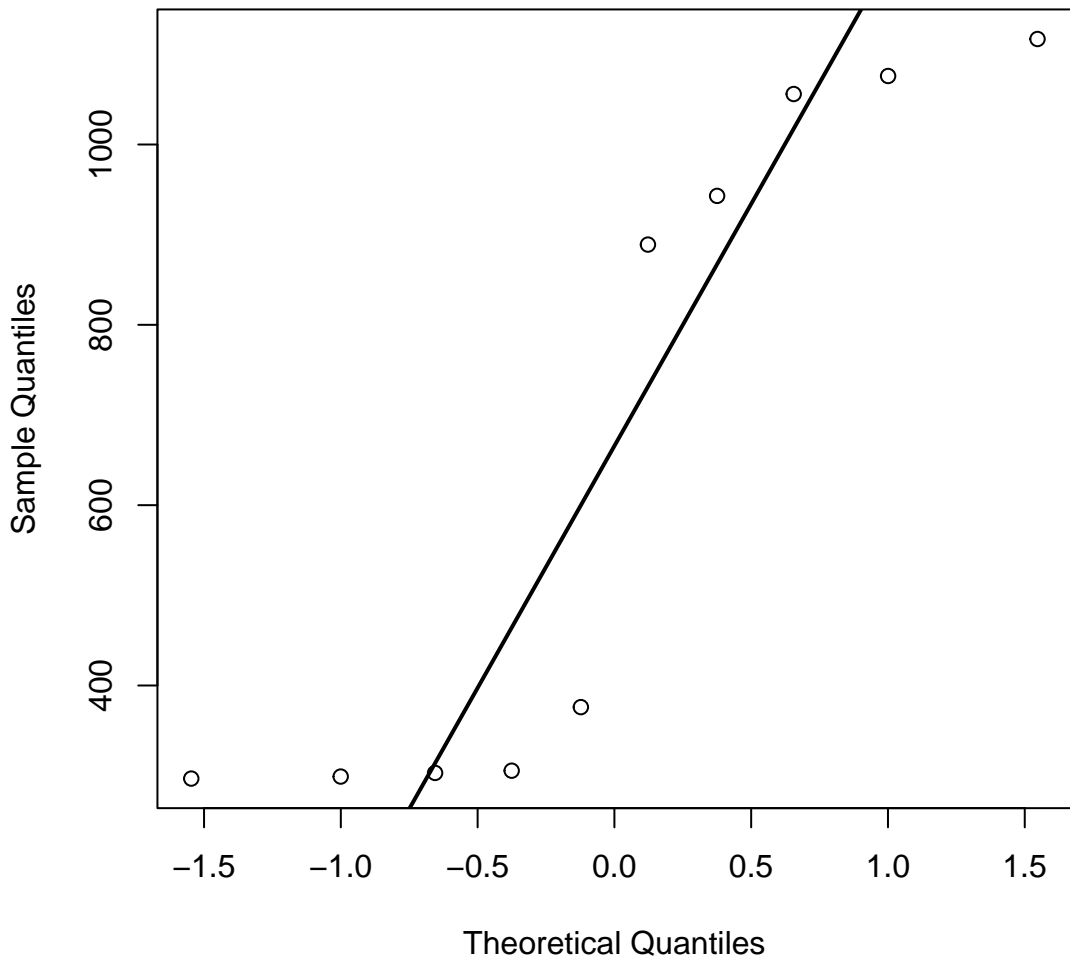

Supplement: Supplementary file 6 — Source Data [file 41467_2026_71014_MOESM6_ESM.zip › Source Data/Statistical Report/Diagnosis/Fig5i_QQ_Plot.pdf]

**Q-Q Plot**

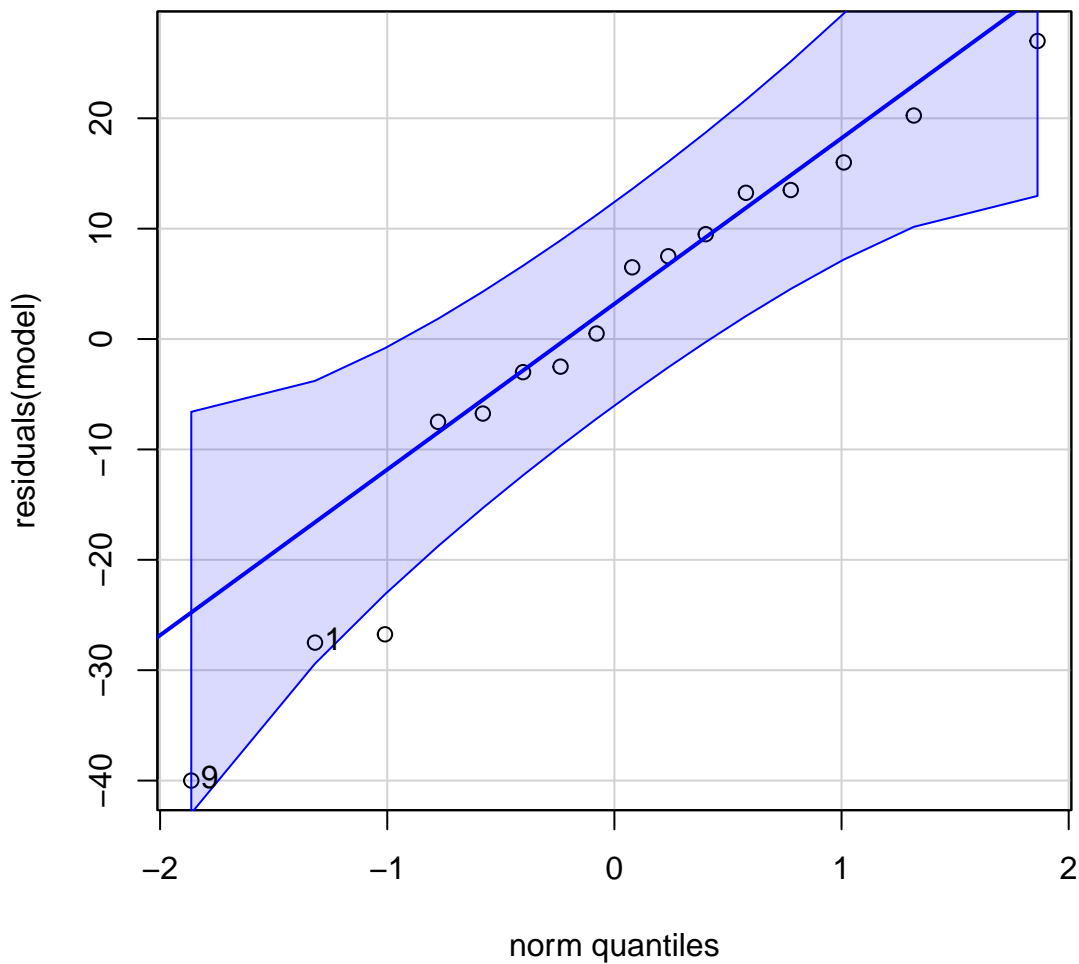

Supplement: Supplementary file 6 — Source Data [file 41467_2026_71014_MOESM6_ESM.zip › Source Data/Statistical Report/Diagnosis/Fig6c_CD22_QQ_Plot.pdf]

Q-Q Plot

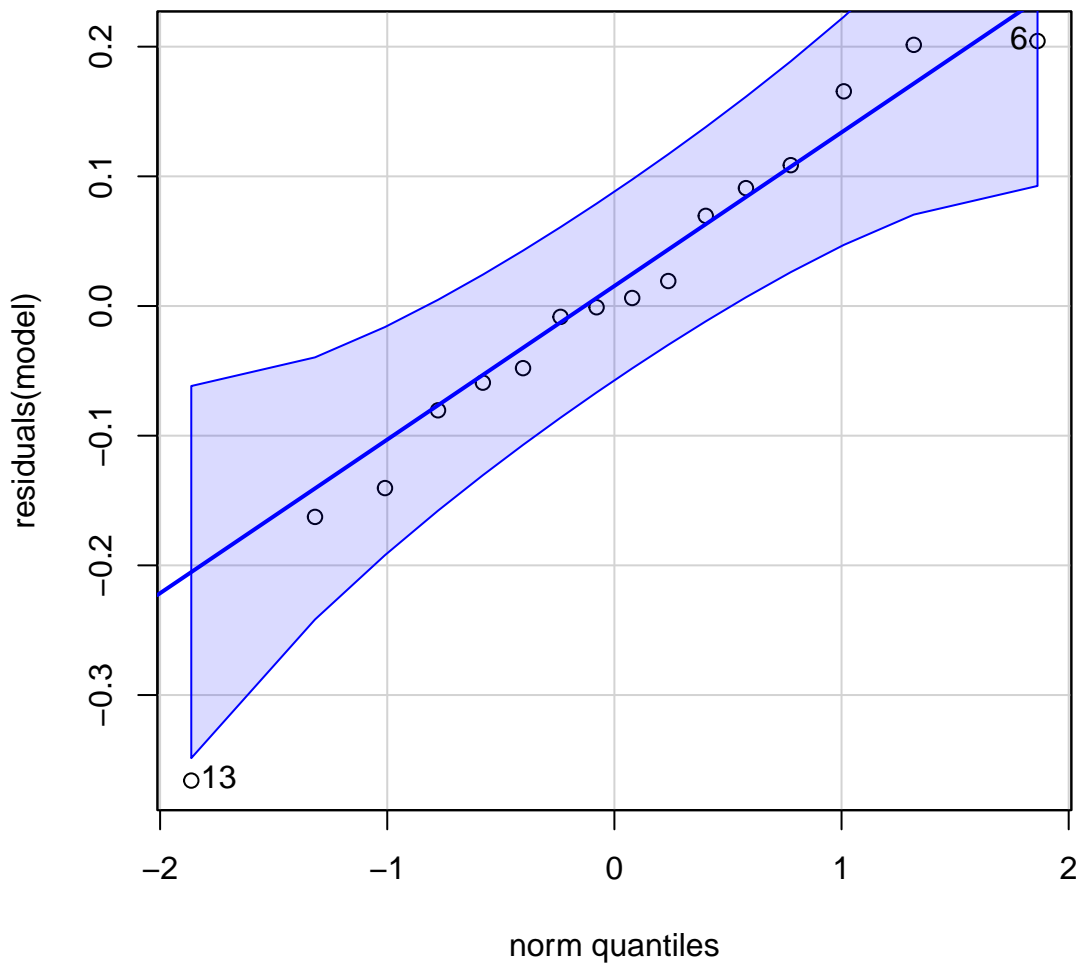

Supplement: Supplementary file 6 — Source Data [file 41467_2026_71014_MOESM6_ESM.zip › Source Data/Statistical Report/Diagnosis/Fig6c_dTomato_QQ_Plot.pdf]

Q-Q Plot

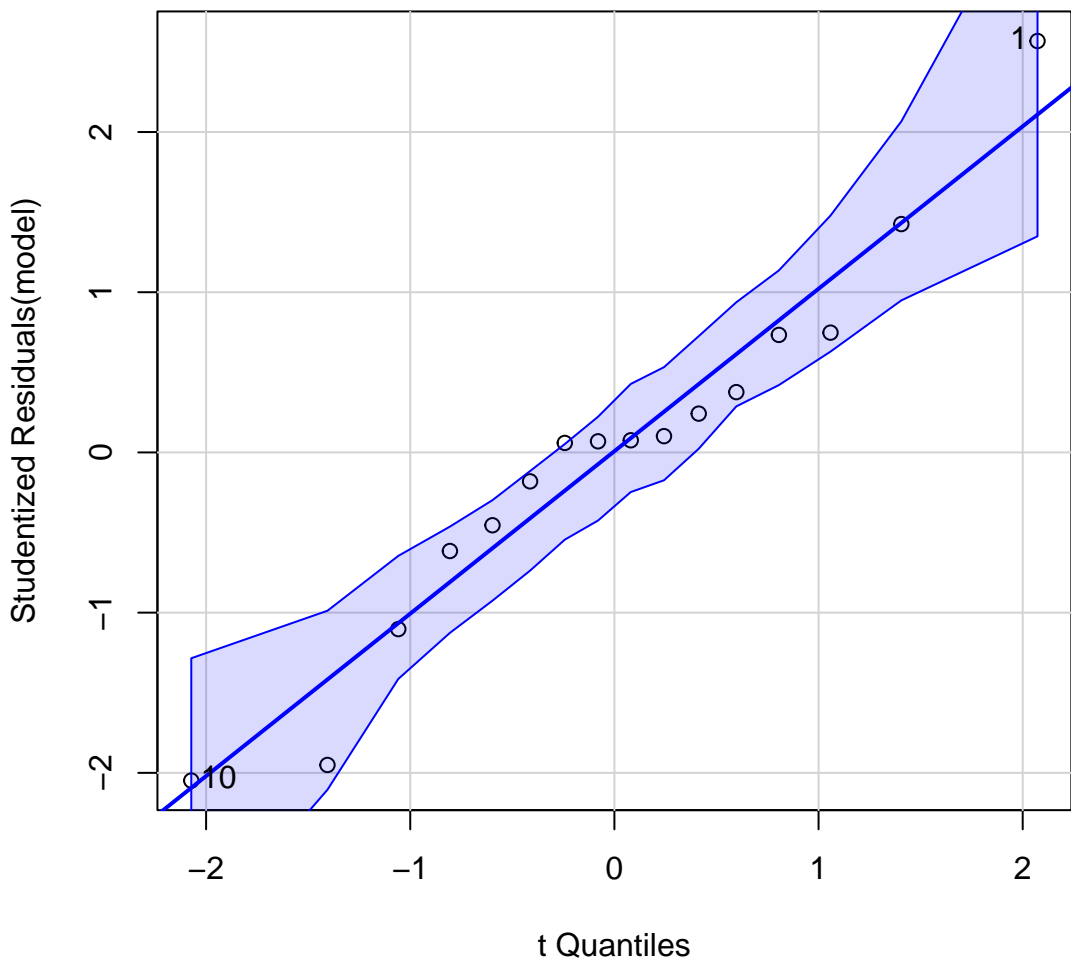

Supplement: Supplementary file 6 — Source Data [file 41467_2026_71014_MOESM6_ESM.zip › Source Data/Statistical Report/Diagnosis/Fig6e_CD22_QQ_Plot.pdf]

Q-Q Plot

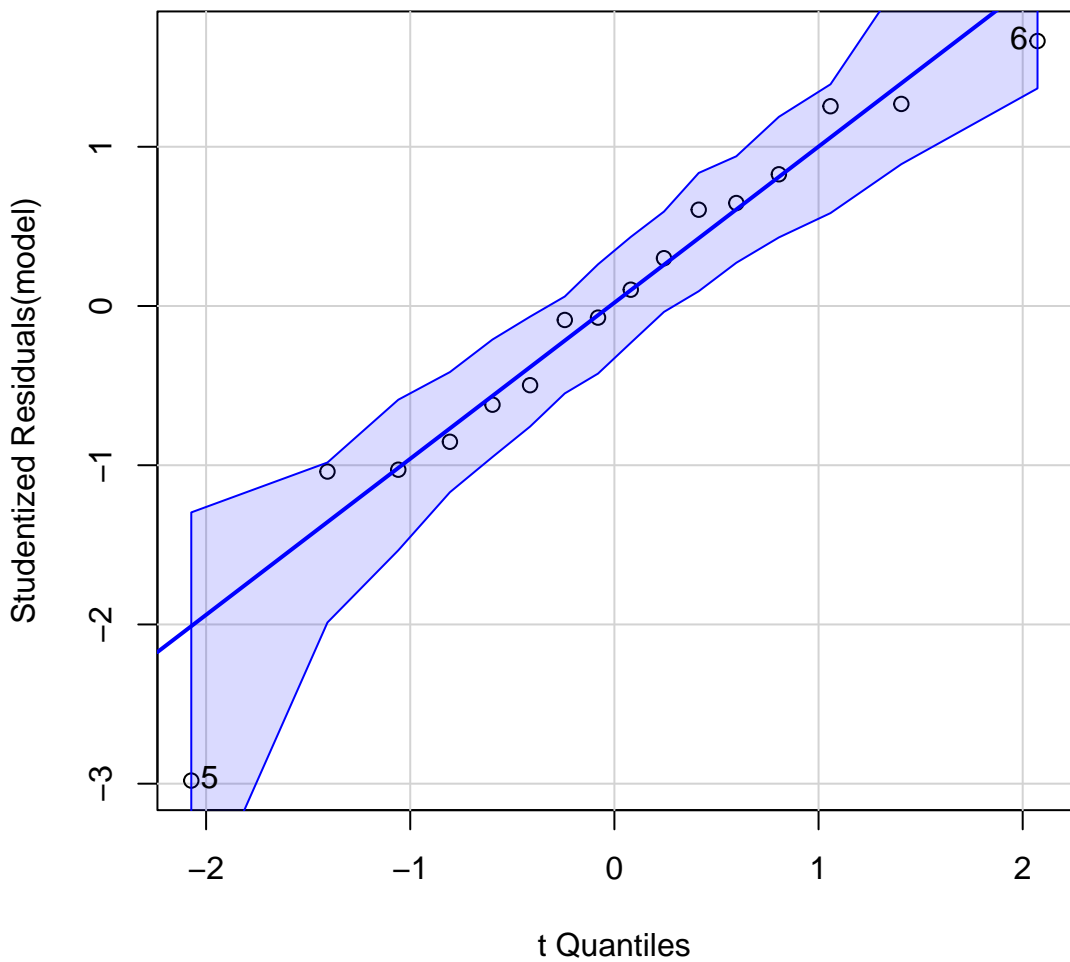

Supplement: Supplementary file 6 — Source Data [file 41467_2026_71014_MOESM6_ESM.zip › Source Data/Statistical Report/Diagnosis/Fig6e_dTomato_QQ_Plot.pdf]

**Q-Q Plot of AF488\_Pos**

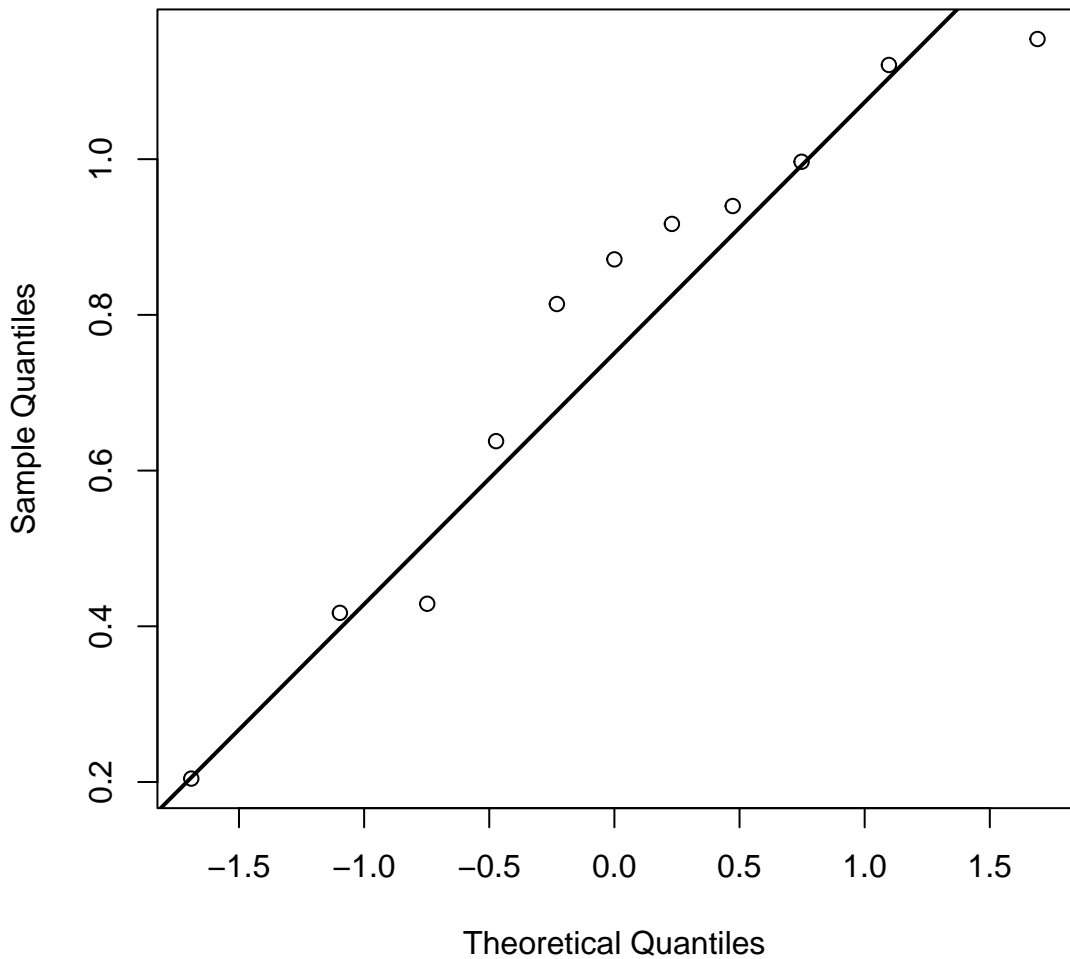

Supplement: Supplementary file 6 — Source Data [file 41467_2026_71014_MOESM6_ESM.zip › Source Data/Statistical Report/Diagnosis/Fig6g_QQ_Plot.pdf]

**Q-Q Plot of Mye\_Dil**

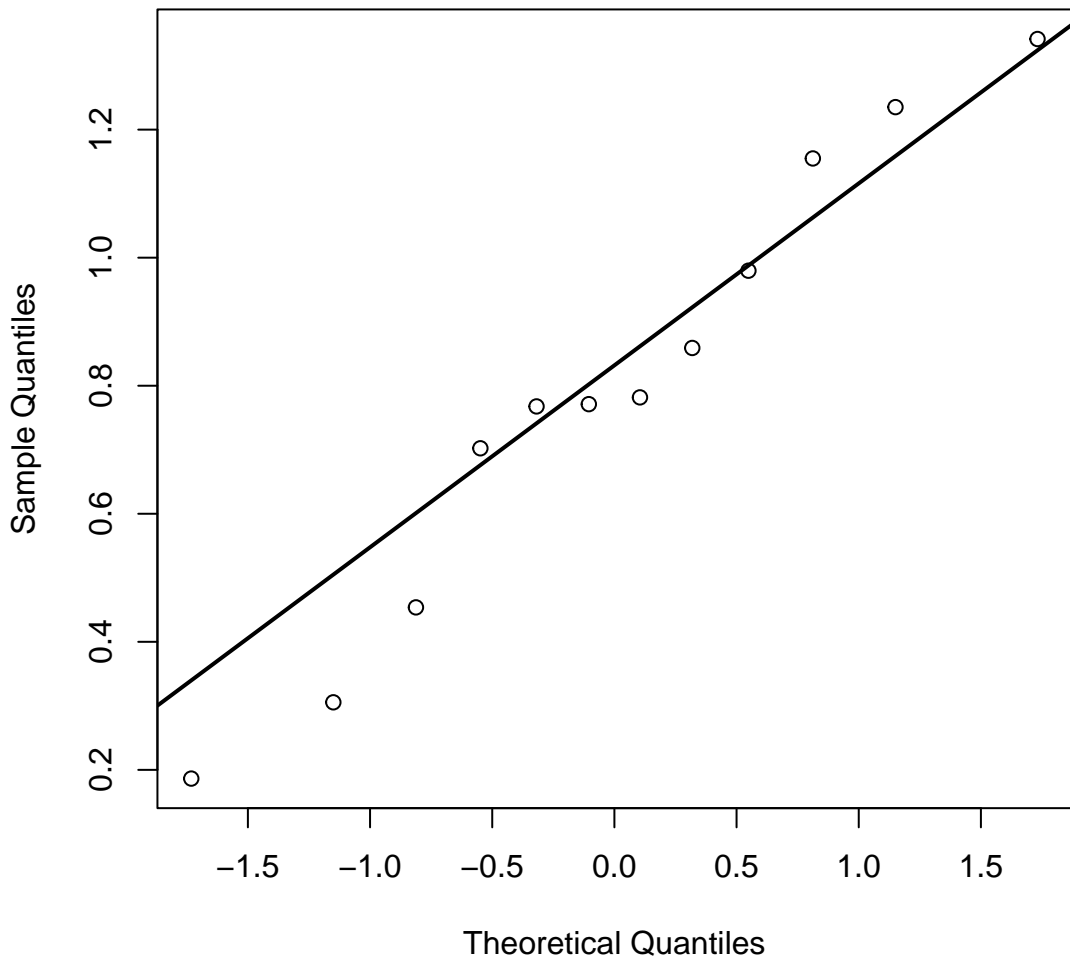

Supplement: Supplementary file 6 — Source Data [file 41467_2026_71014_MOESM6_ESM.zip › Source Data/Statistical Report/Diagnosis/Fig7b_QQ_Plot.pdf]

**Q-Q Plot of Mye\_Dil**

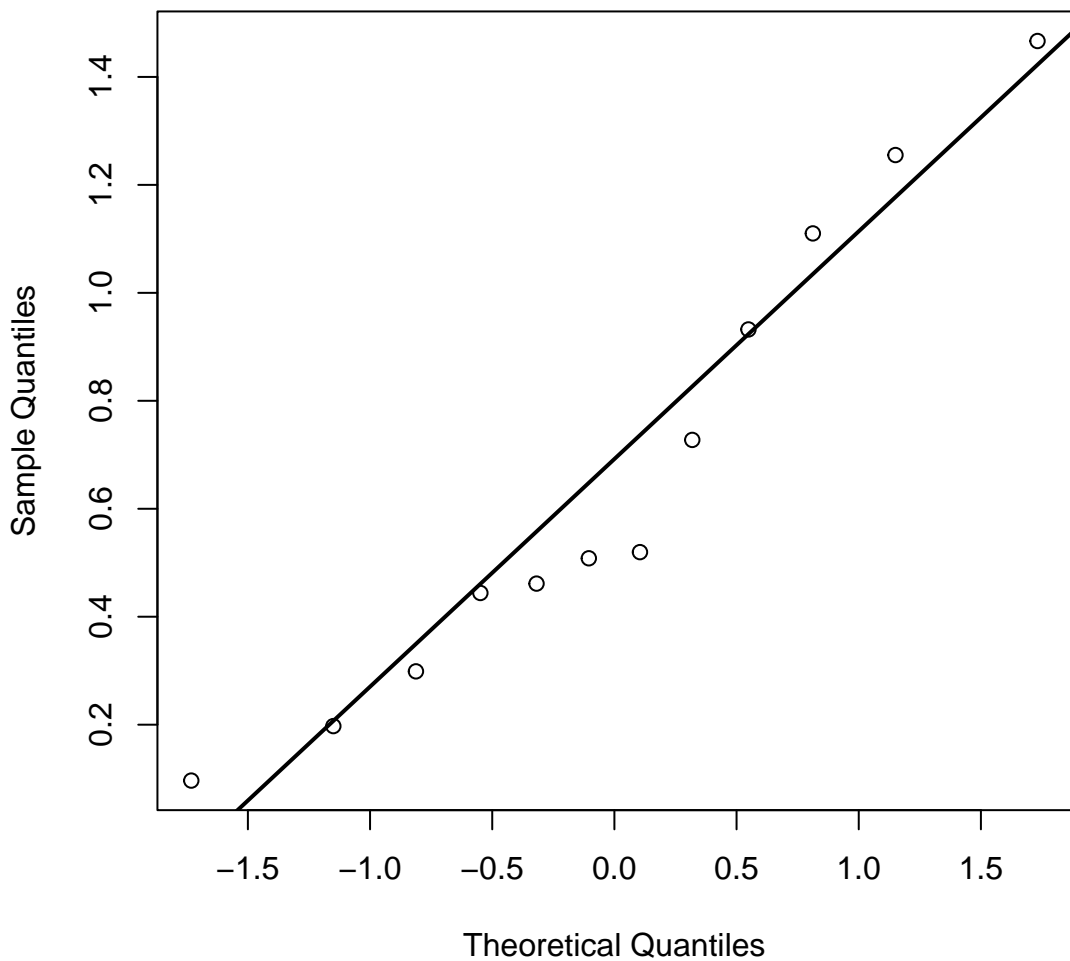

Supplement: Supplementary file 6 — Source Data [file 41467_2026_71014_MOESM6_ESM.zip › Source Data/Statistical Report/Diagnosis/Fig7c_QQ_Plot.pdf]

Q-Q Plot

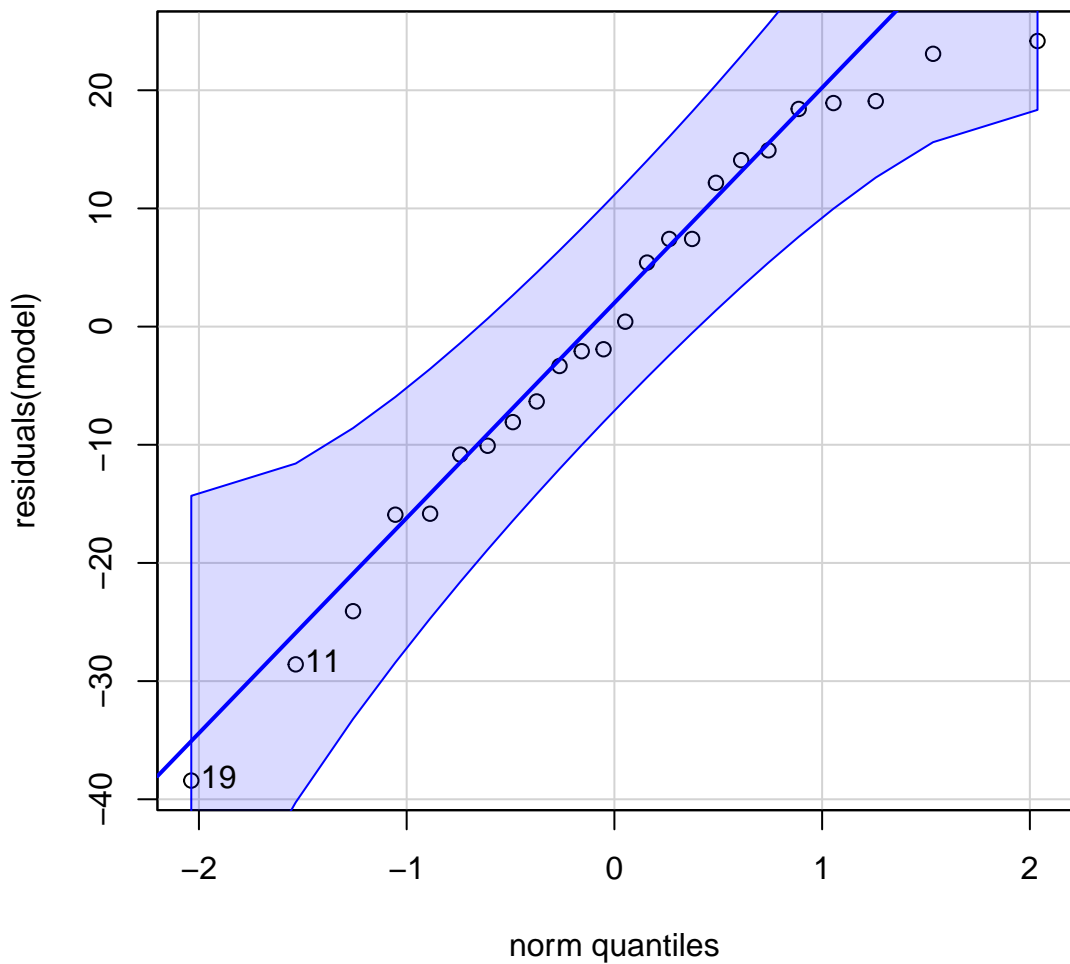

Supplement: Supplementary file 6 — Source Data [file 41467_2026_71014_MOESM6_ESM.zip › Source Data/Statistical Report/Diagnosis/Fig7e_Residuals_QQ_Plot.pdf]

Q-Q Plot

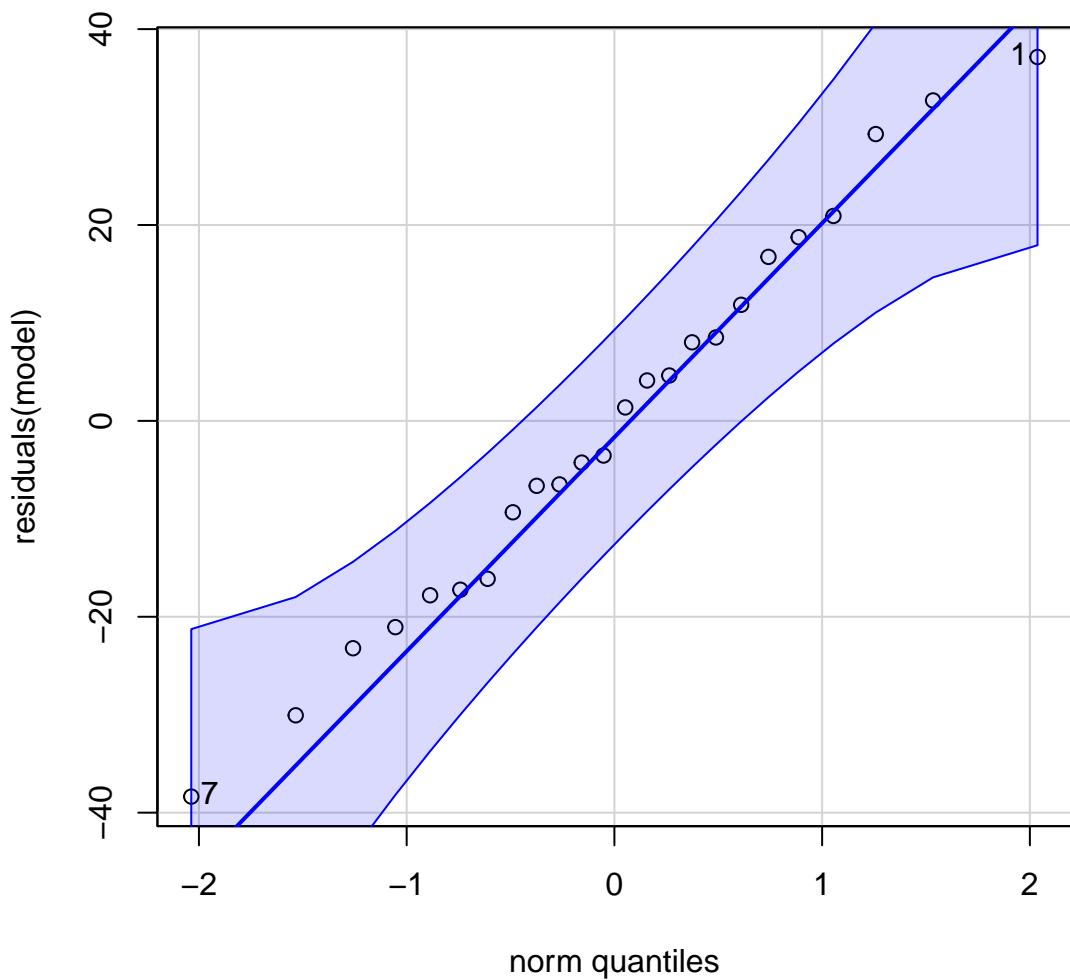

Supplement: Supplementary file 6 — Source Data [file 41467_2026_71014_MOESM6_ESM.zip › Source Data/Statistical Report/Diagnosis/Fig7f_Residuals_QQ_Plot.pdf]

Q-Q Plot

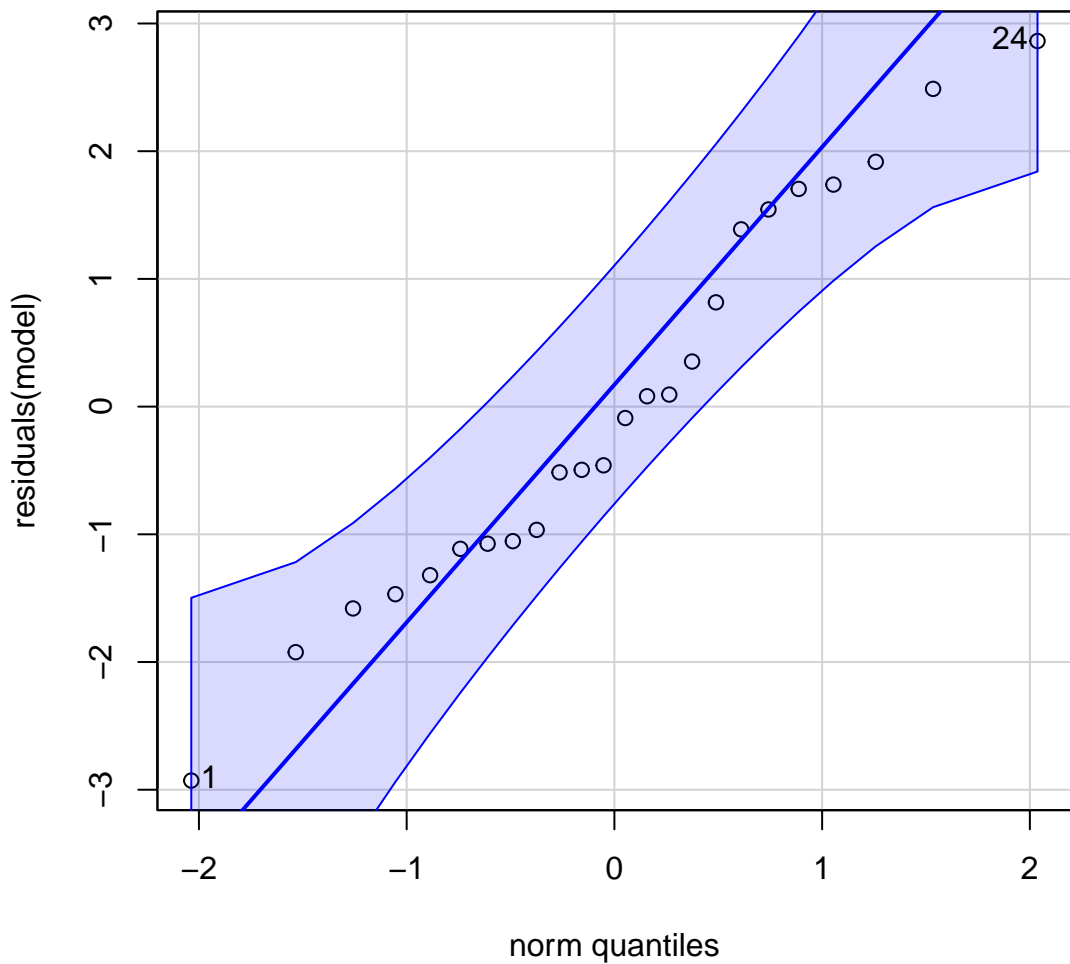

Supplement: Supplementary file 6 — Source Data [file 41467_2026_71014_MOESM6_ESM.zip › Source Data/Statistical Report/Diagnosis/Fig7i_dMbp_Iba1_Residuals_QQ_Plot.pdf]

**Q-Q Plot**

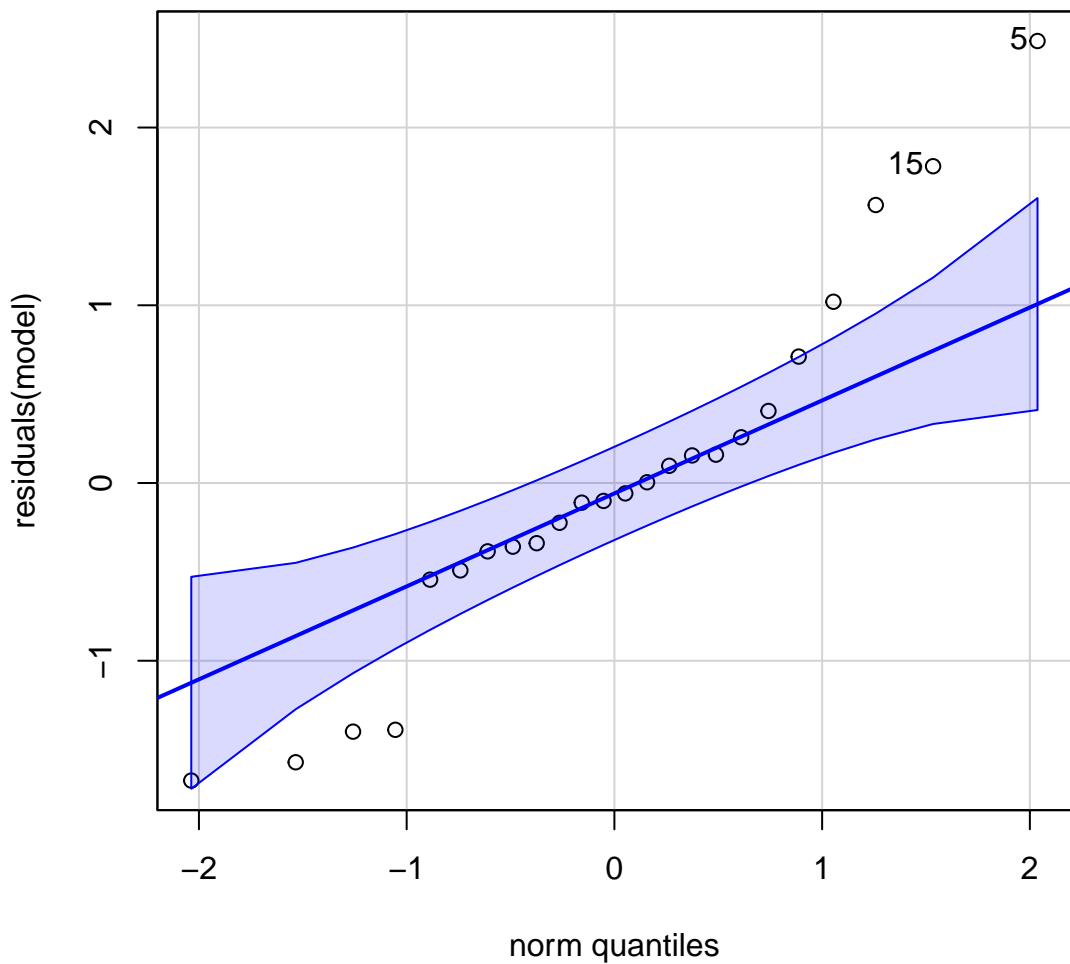

Supplement: Supplementary file 6 — Source Data [file 41467_2026_71014_MOESM6_ESM.zip › Source Data/Statistical Report/Diagnosis/Fig7i_dMbp_Residuals_QQ_Plot.pdf]

Q-Q Plot

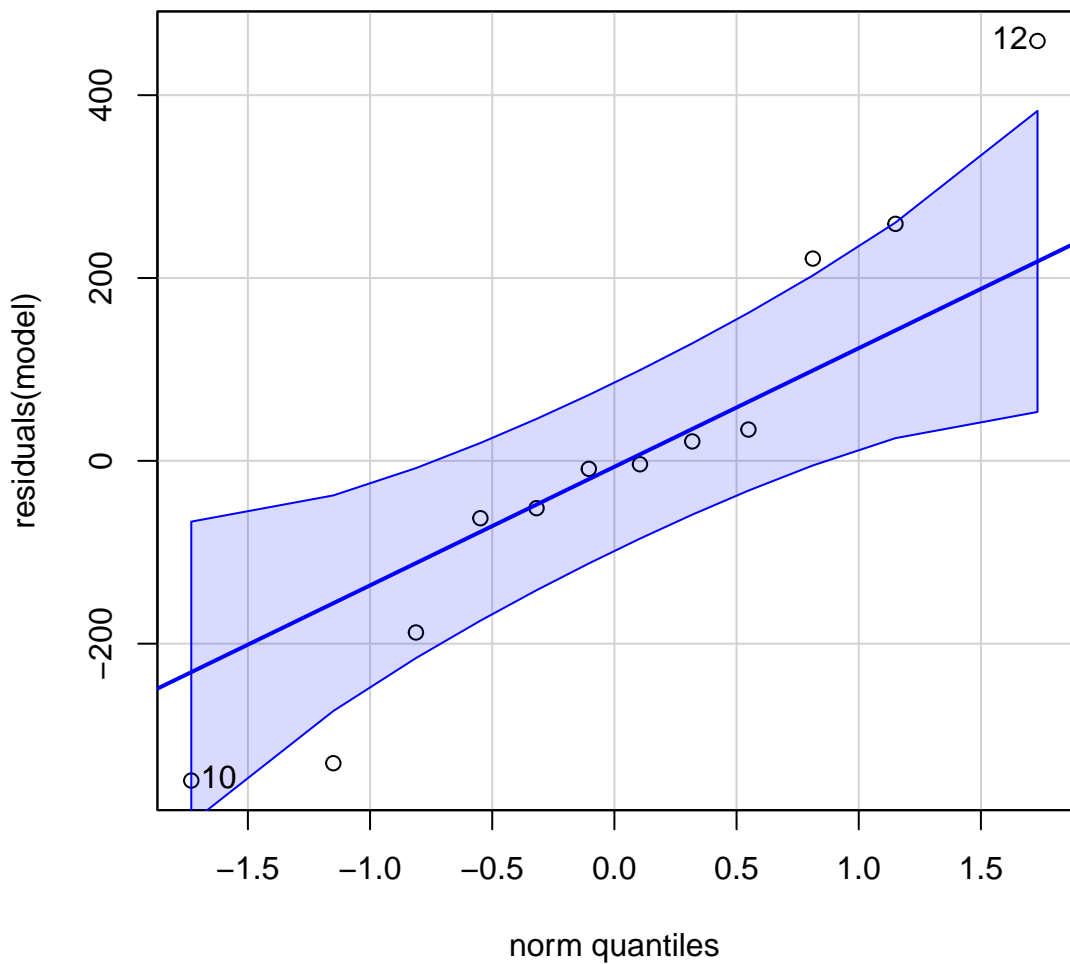

Supplement: Supplementary file 6 — Source Data [file 41467_2026_71014_MOESM6_ESM.zip › Source Data/Statistical Report/Diagnosis/FigS1d_QQ_Plot.pdf]

Q-Q Plot

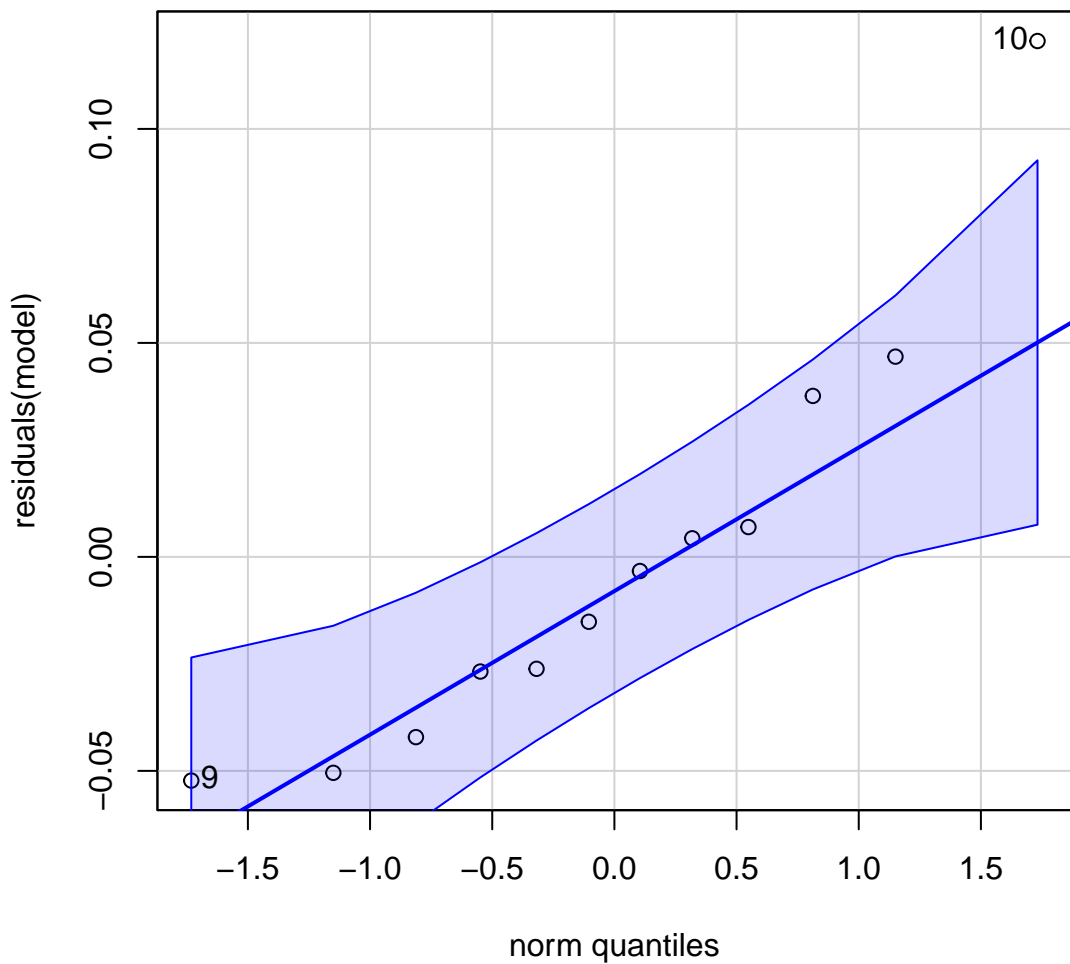

Supplement: Supplementary file 6 — Source Data [file 41467_2026_71014_MOESM6_ESM.zip › Source Data/Statistical Report/Diagnosis/FigS1e_QQ_Plot.pdf]

Q-Q Plot

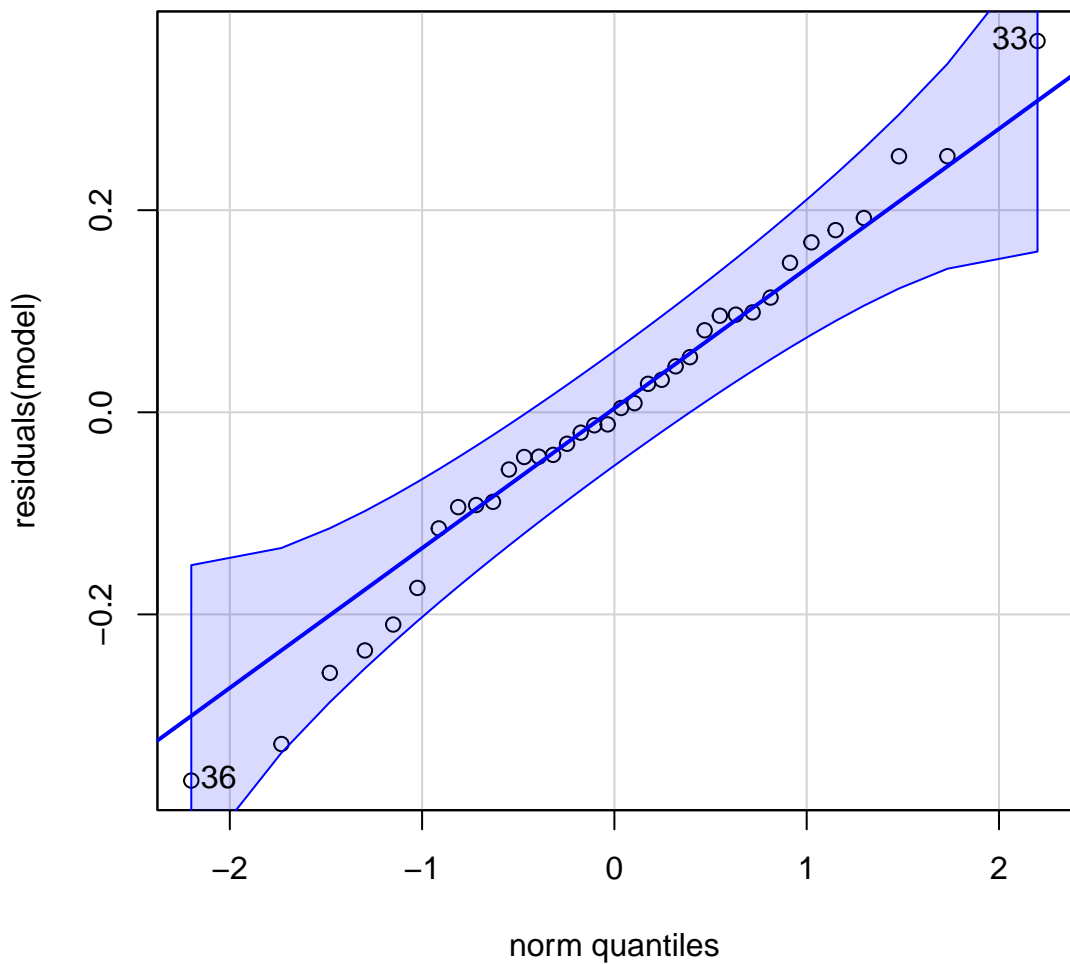

Supplement: Supplementary file 6 — Source Data [file 41467_2026_71014_MOESM6_ESM.zip › Source Data/Statistical Report/Diagnosis/FigS4b_QQ_Plot.pdf]

Q-Q Plot

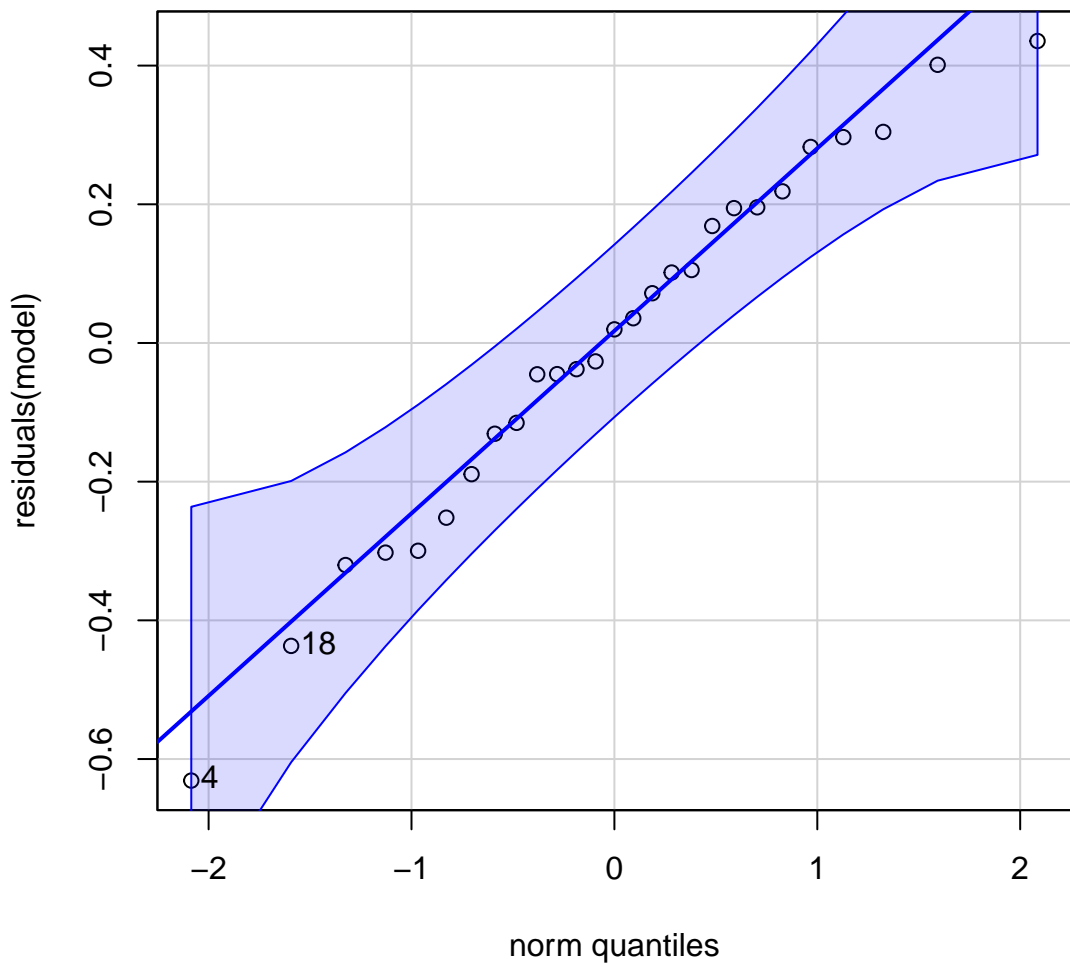

Supplement: Supplementary file 6 — Source Data [file 41467_2026_71014_MOESM6_ESM.zip › Source Data/Statistical Report/Diagnosis/FigS6b_QQ_Plot.pdf]

Q-Q Plot

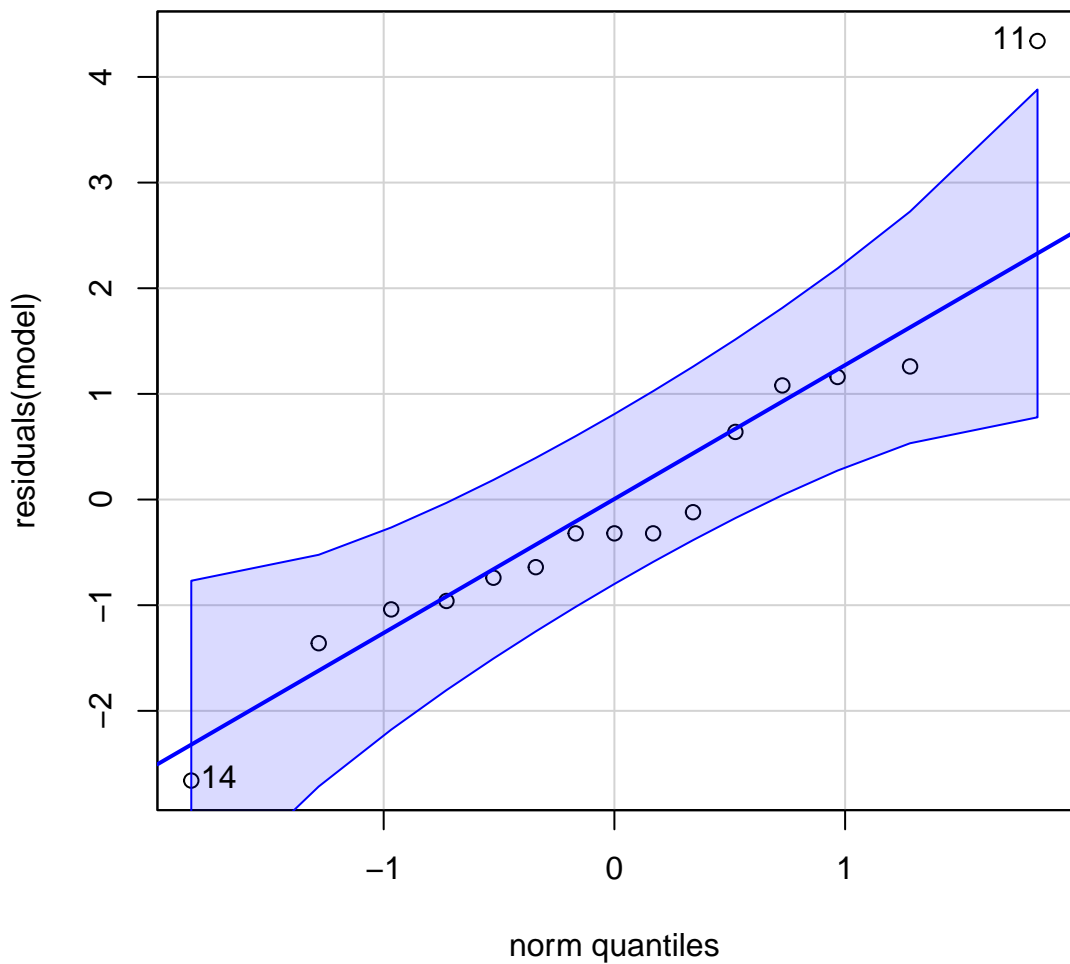

Supplement: Supplementary file 6 — Source Data [file 41467_2026_71014_MOESM6_ESM.zip › Source Data/Statistical Report/Diagnosis/FigS6c_QQ_Plot.pdf]

**Q-Q Plot**

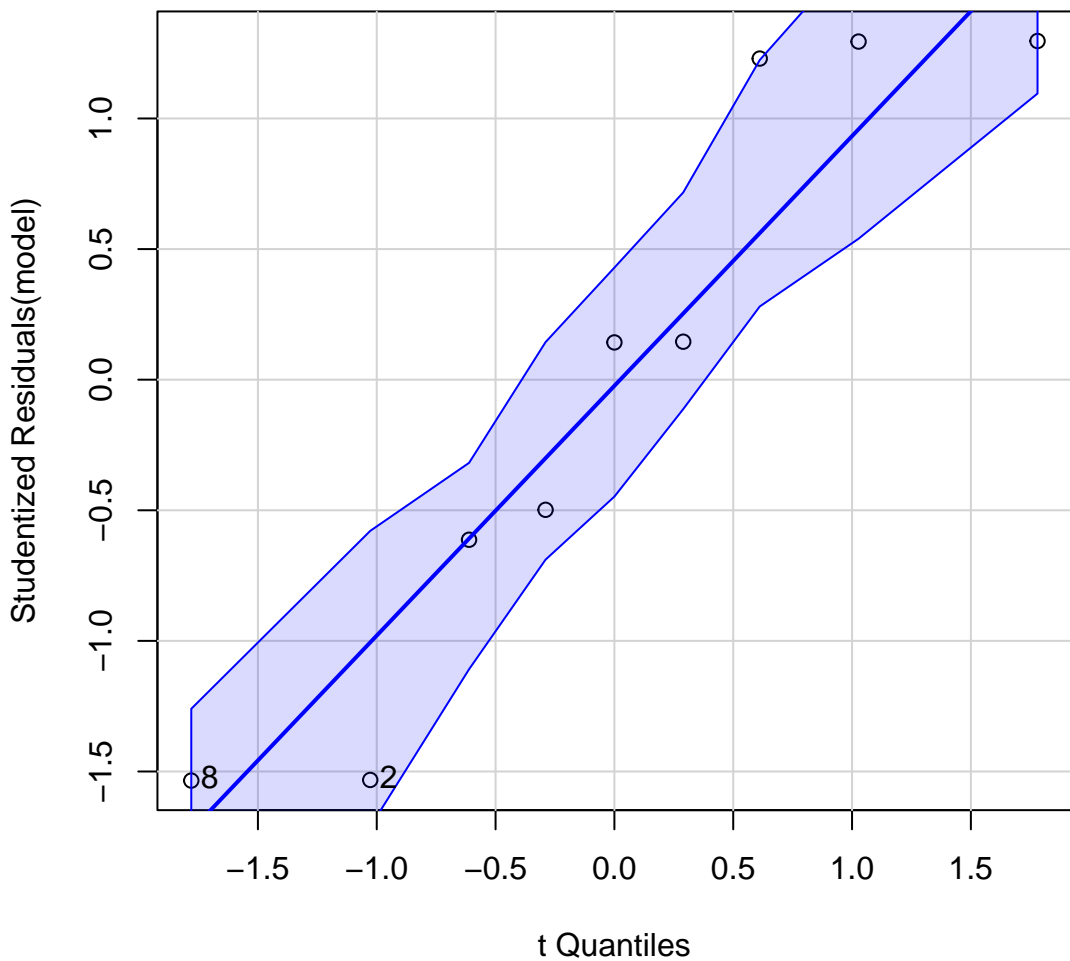

Supplement: Supplementary file 6 — Source Data [file 41467_2026_71014_MOESM6_ESM.zip › Source Data/Statistical Report/Diagnosis/FigS7b_Residuals_QQ_Plot.pdf]

Q-Q Plot

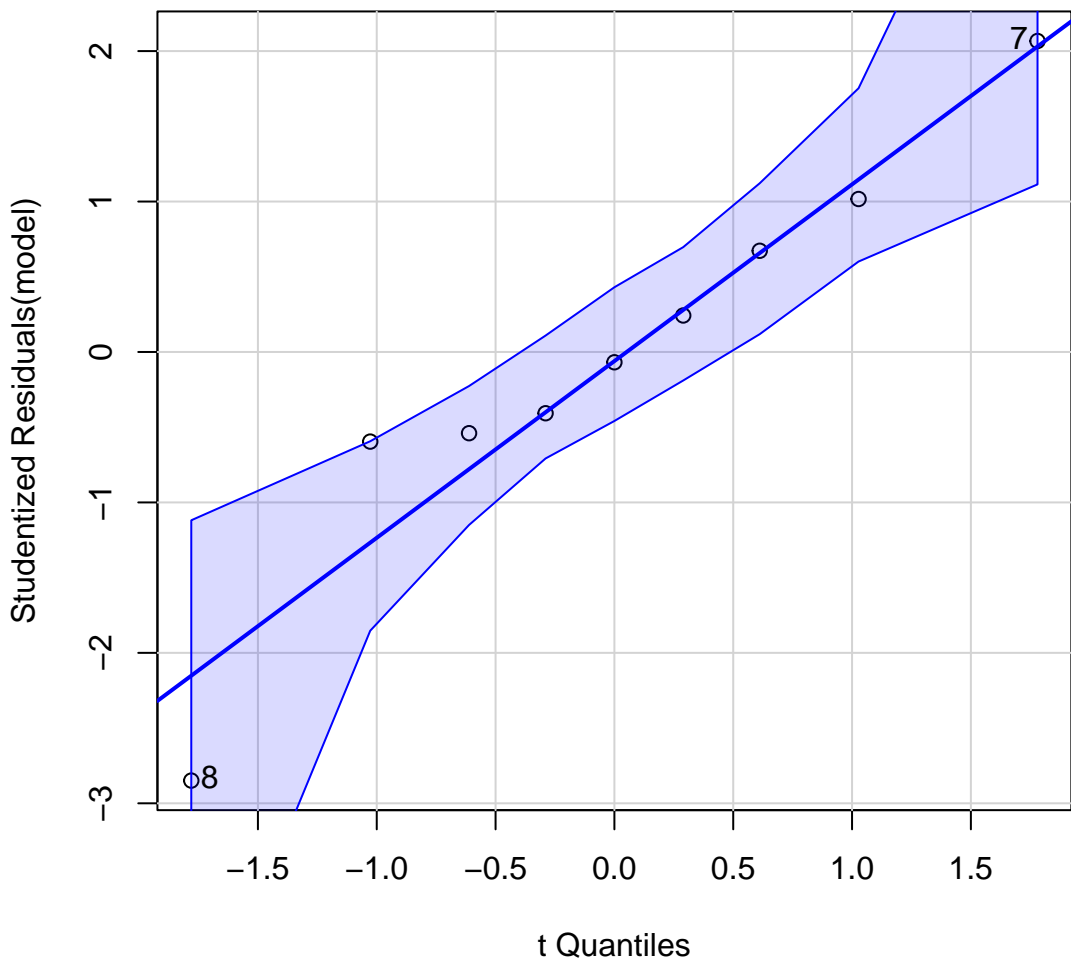

Supplement: Supplementary file 6 — Source Data [file 41467_2026_71014_MOESM6_ESM.zip › Source Data/Statistical Report/Diagnosis/FigS7c_Residuals_QQ_Plot.pdf]

Q-Q Plot

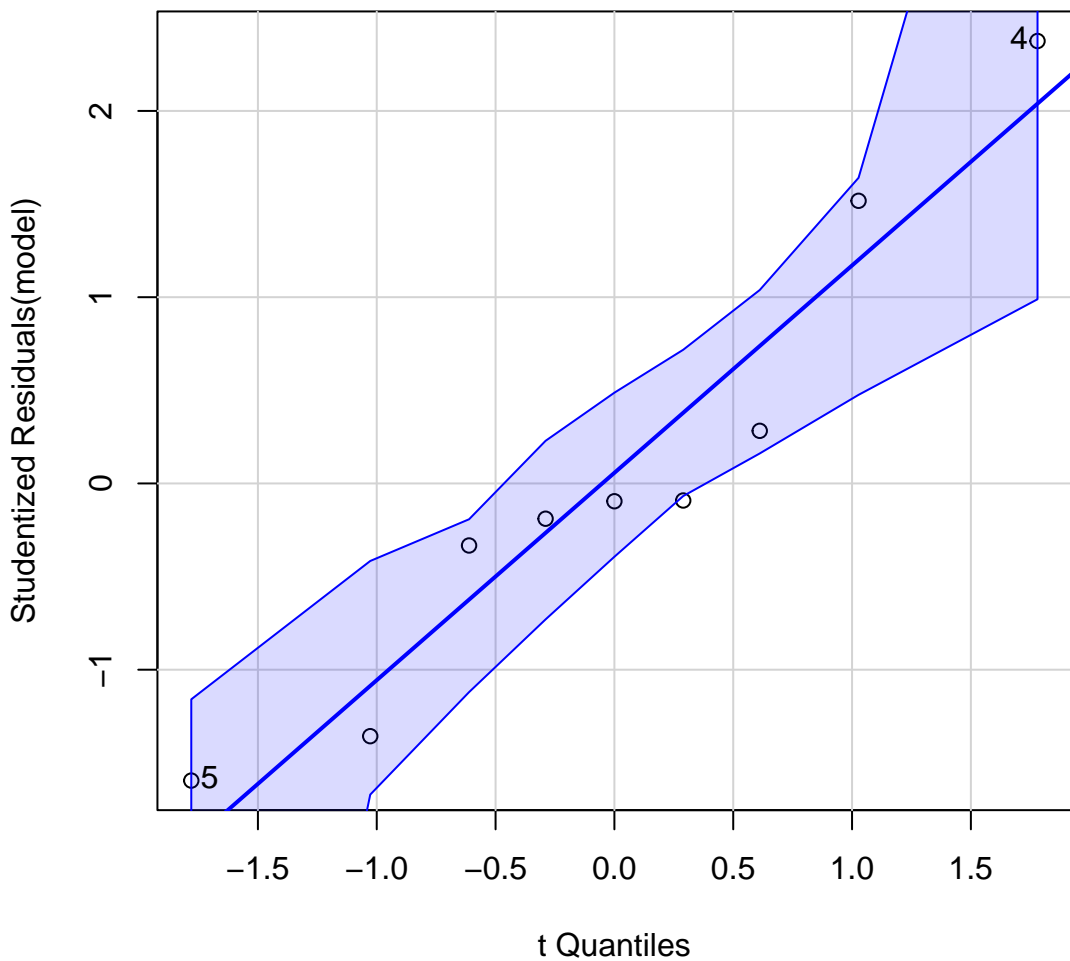

Supplement: Supplementary file 6 — Source Data [file 41467_2026_71014_MOESM6_ESM.zip › Source Data/Statistical Report/Diagnosis/FigS8a_Residuals_QQ_Plot.pdf]

Q-Q Plot

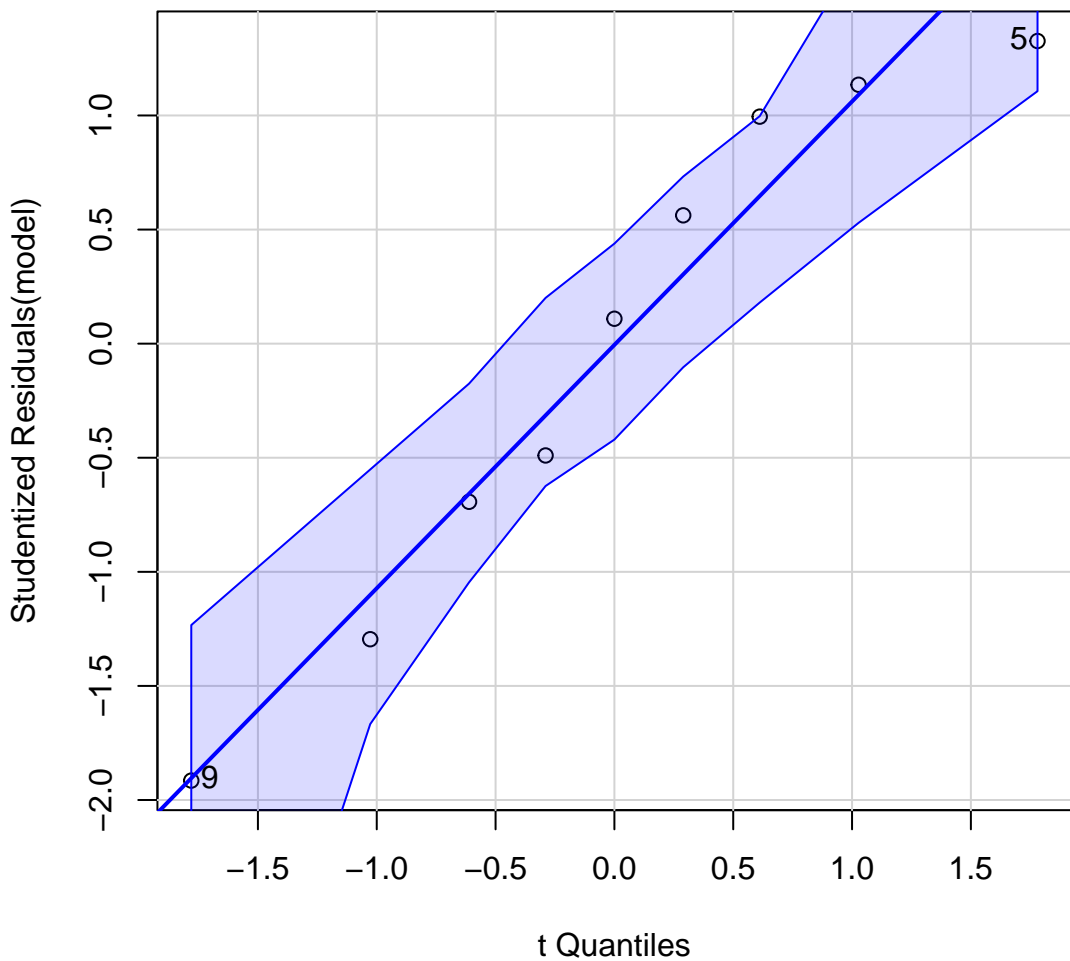

Supplement: Supplementary file 6 — Source Data [file 41467_2026_71014_MOESM6_ESM.zip › Source Data/Statistical Report/Diagnosis/FigS8b_Residuals_QQ_Plot.pdf]

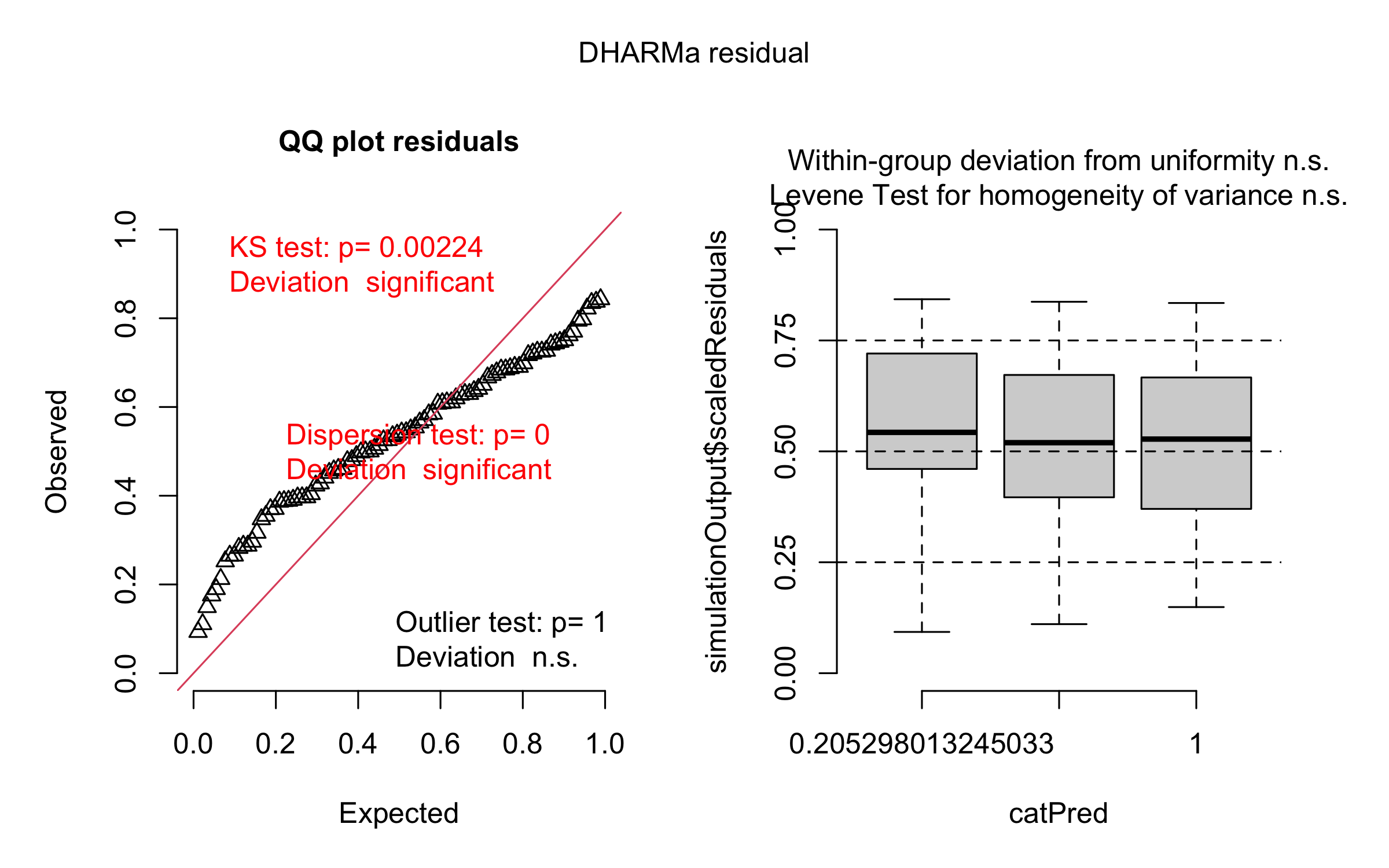

Supplement: Supplementary file 6 — Source Data [file 41467_2026_71014_MOESM6_ESM.zip › Source Data/Statistical Report/Diagnosis/Figure2B_Max_Intersection_DHARMa.png]

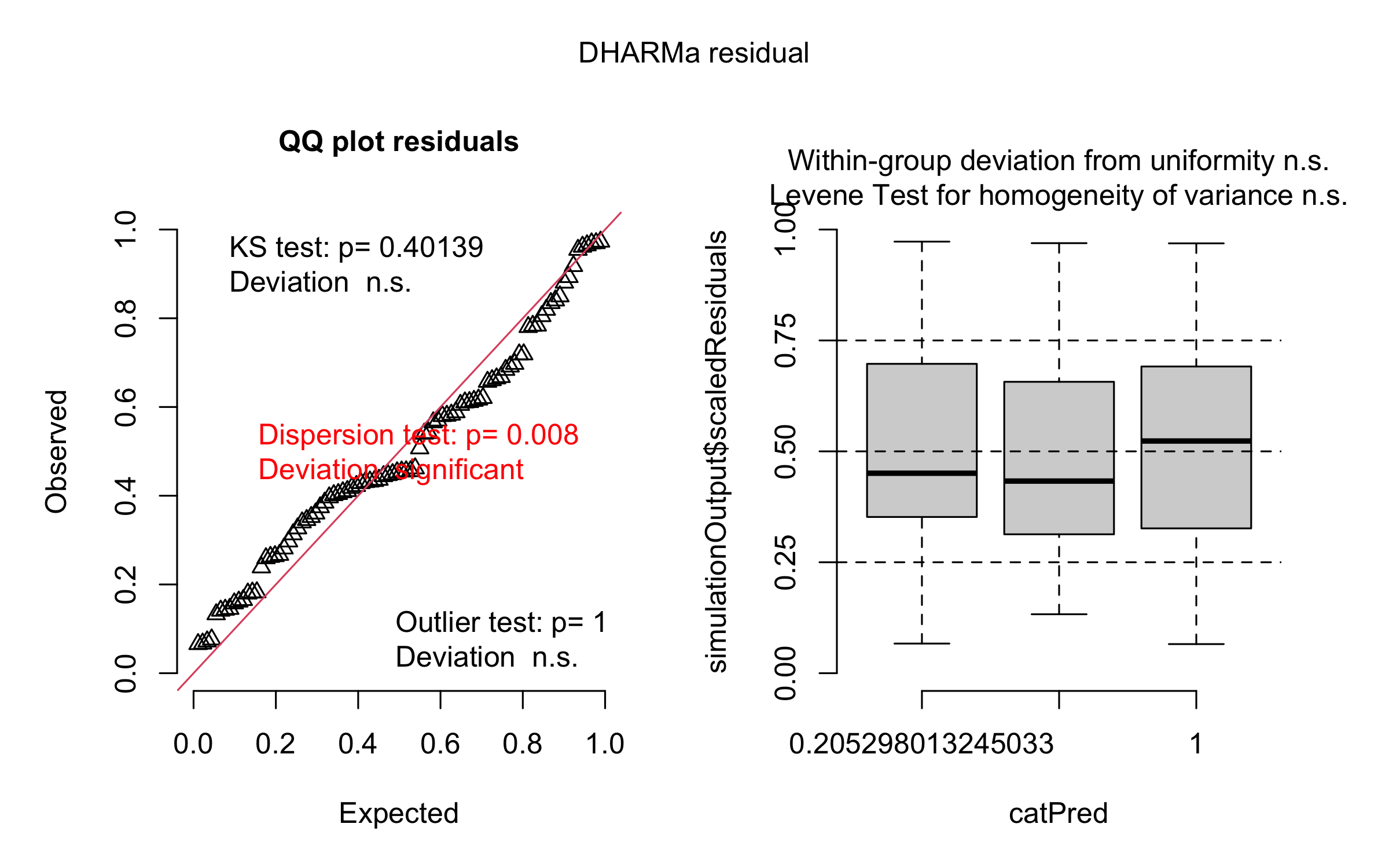

Supplement: Supplementary file 6 — Source Data [file 41467_2026_71014_MOESM6_ESM.zip › Source Data/Statistical Report/Diagnosis/Figure2B_Number_of_Branches_DHARMa.png]

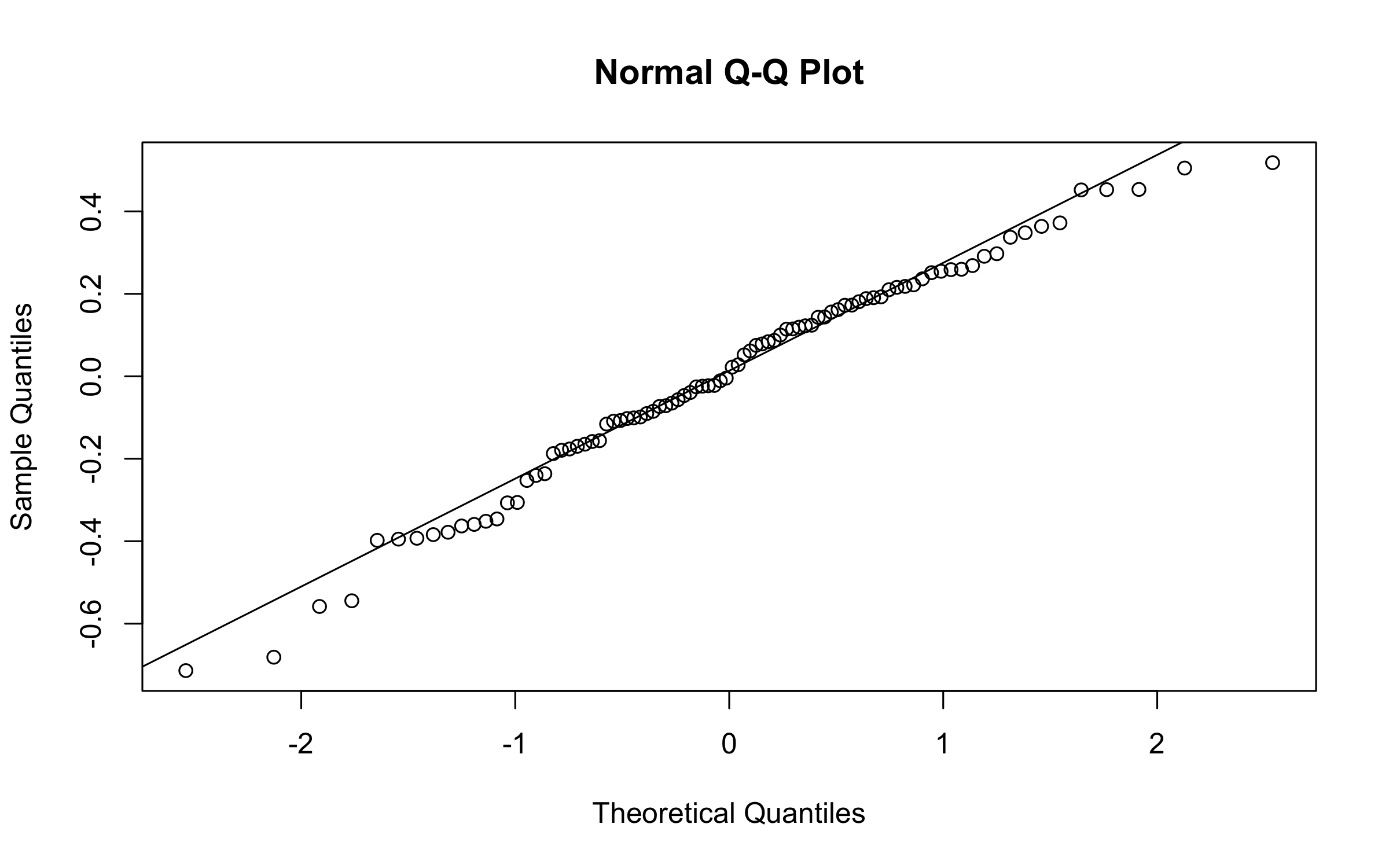

Supplement: Supplementary file 6 — Source Data [file 41467_2026_71014_MOESM6_ESM.zip › Source Data/Statistical Report/Diagnosis/Figure2B_Total_Branches_Log_QQ.png]

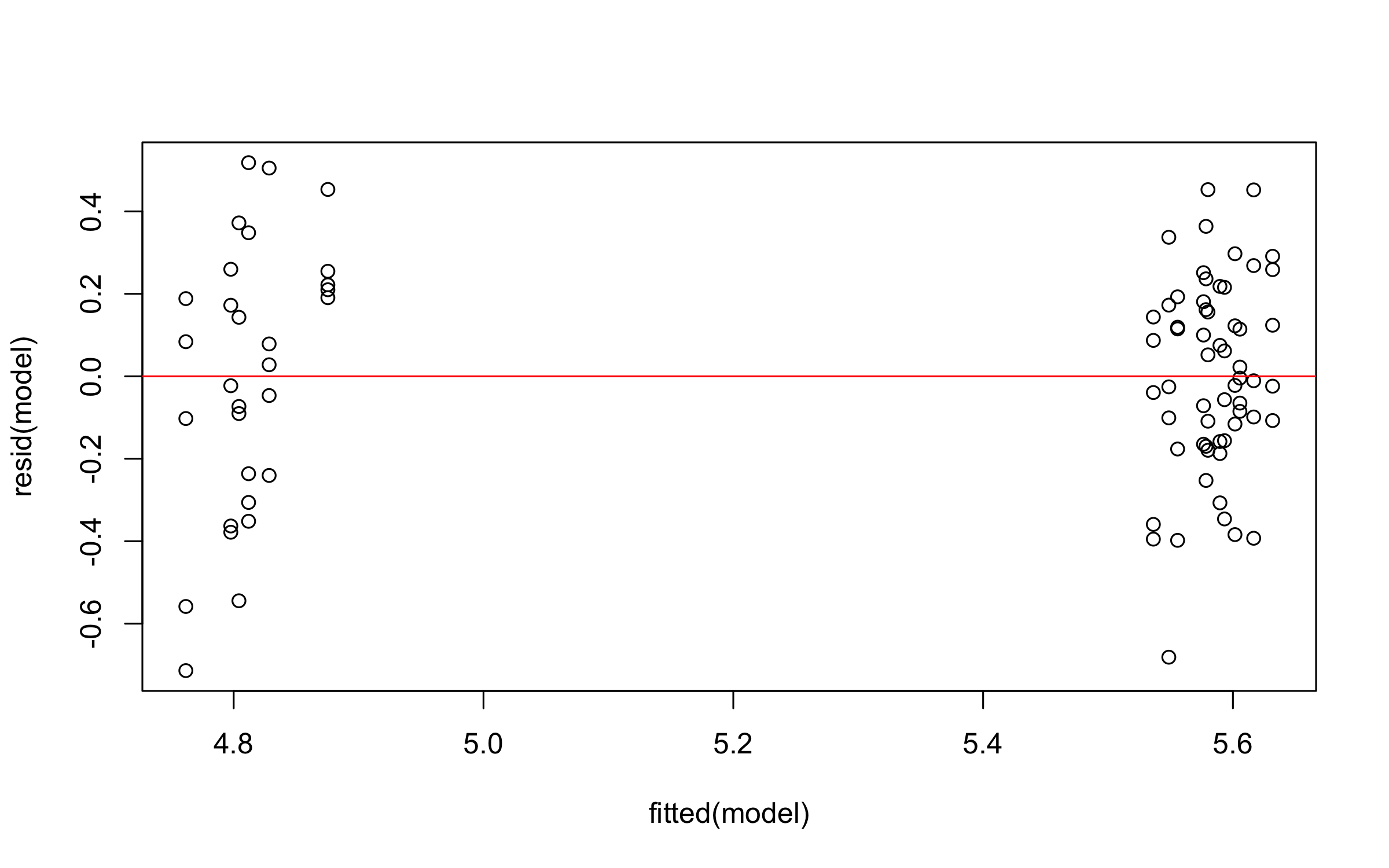

Supplement: Supplementary file 6 — Source Data [file 41467_2026_71014_MOESM6_ESM.zip › Source Data/Statistical Report/Diagnosis/Figure2B_Total_Branches_Log_ResidualFit.png]

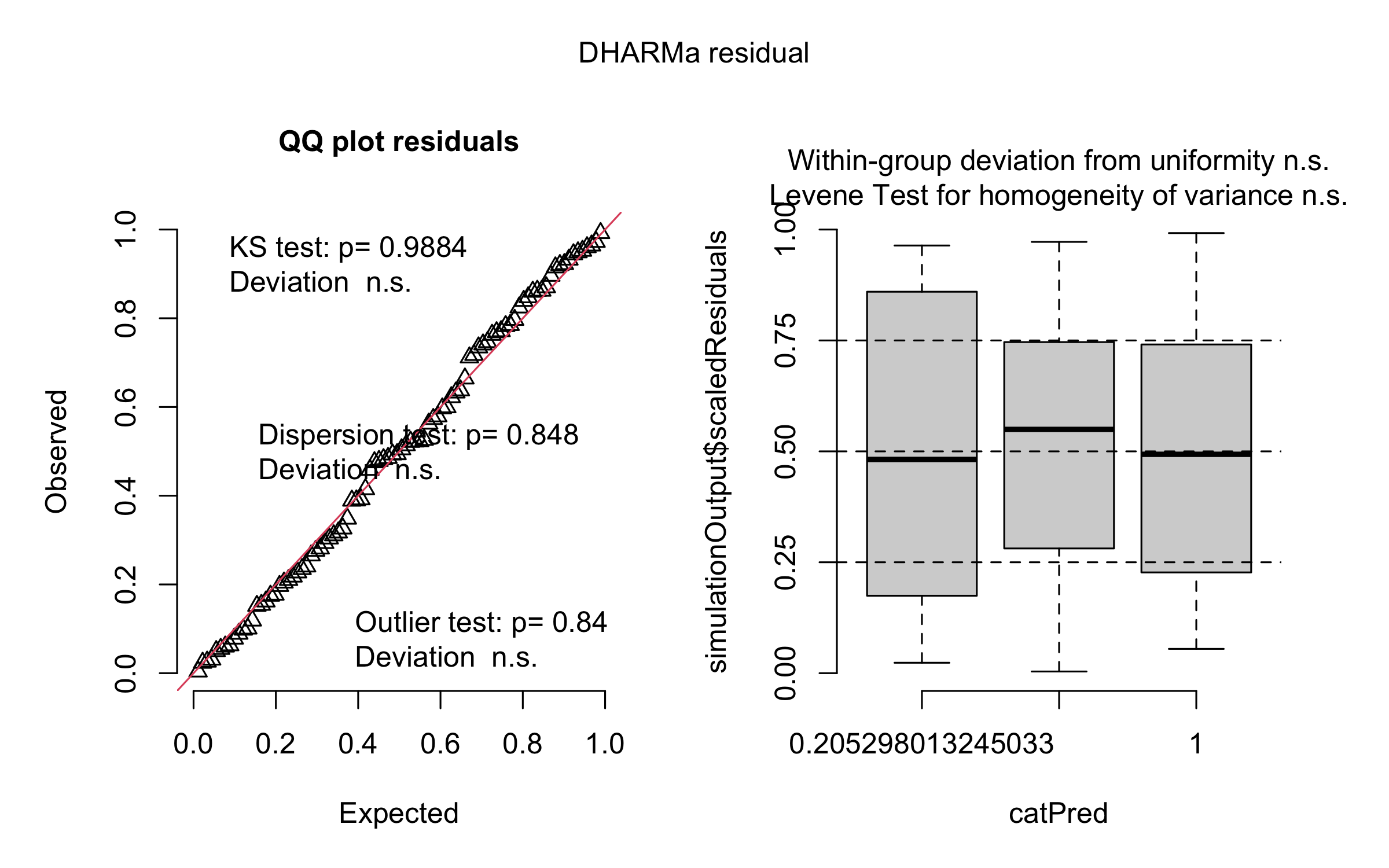

Supplement: Supplementary file 6 — Source Data [file 41467_2026_71014_MOESM6_ESM.zip › Source Data/Statistical Report/Diagnosis/Figure2B_Total_Intersections_DHARMa.png]

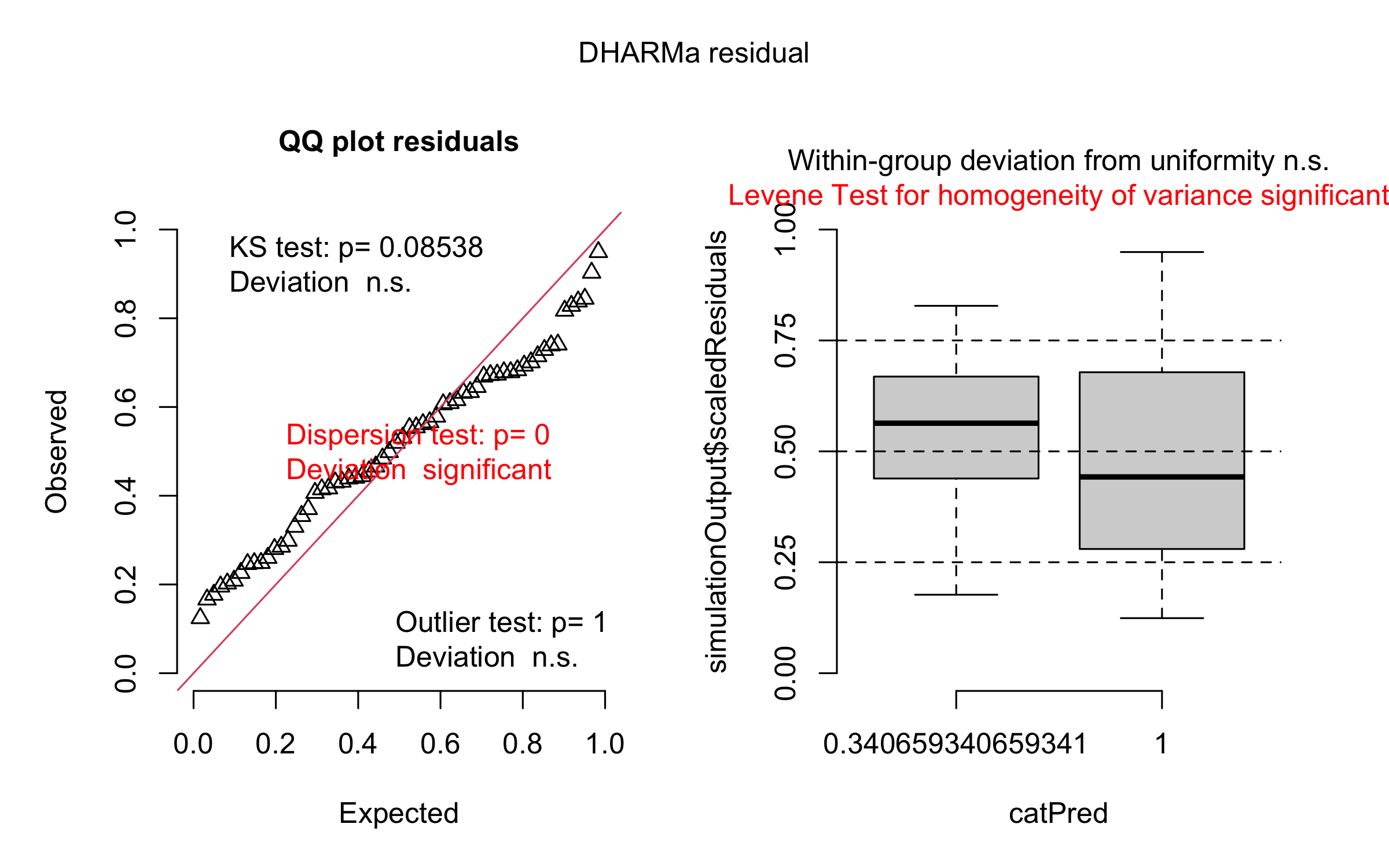

Supplement: Supplementary file 6 — Source Data [file 41467_2026_71014_MOESM6_ESM.zip › Source Data/Statistical Report/Diagnosis/Figure4C_Max_Intersection_DHARMa.png]

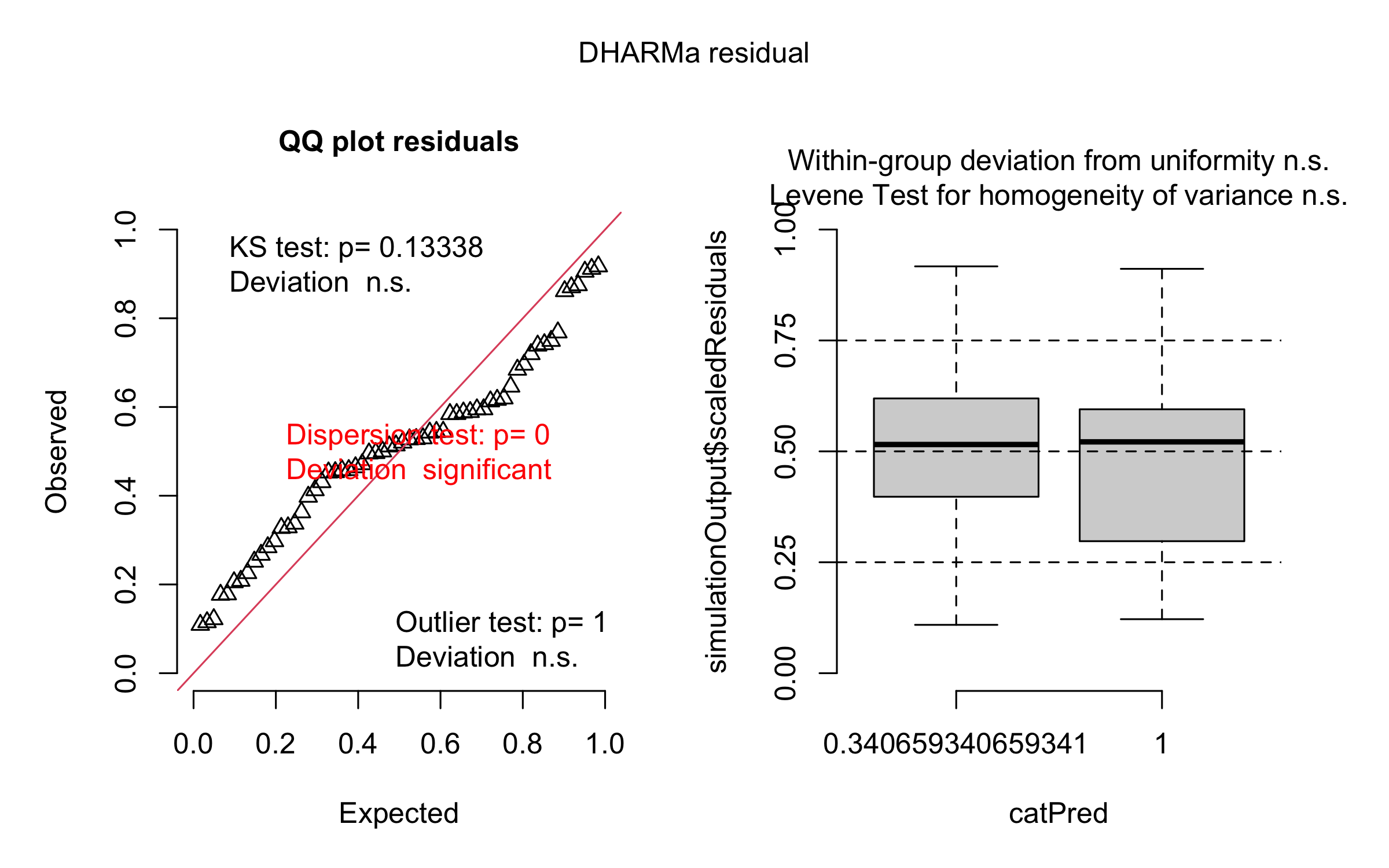

Supplement: Supplementary file 6 — Source Data [file 41467_2026_71014_MOESM6_ESM.zip › Source Data/Statistical Report/Diagnosis/Figure4C_Number_of_Branches_DHARMa.png]

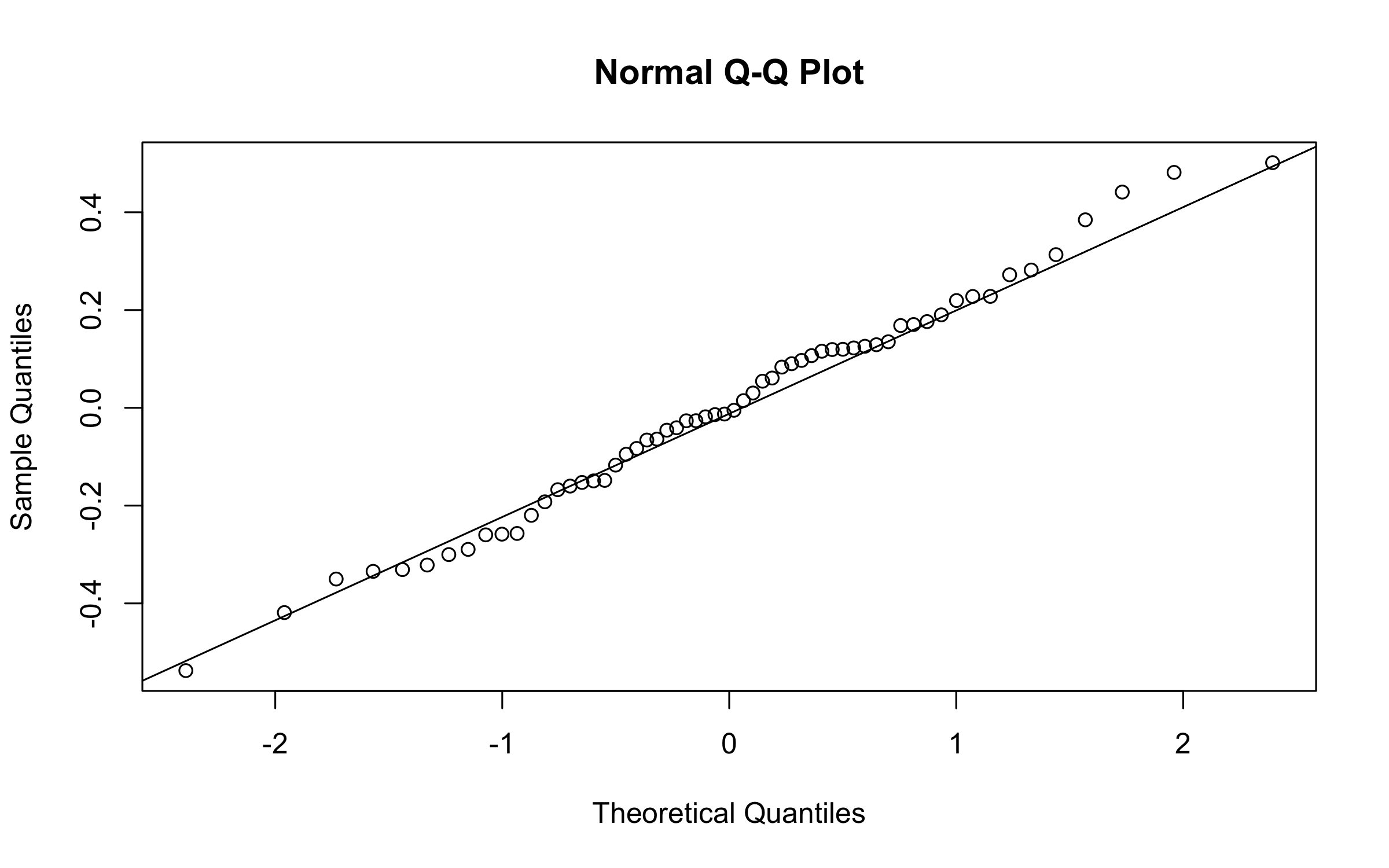

Supplement: Supplementary file 6 — Source Data [file 41467_2026_71014_MOESM6_ESM.zip › Source Data/Statistical Report/Diagnosis/Figure4C_Total_Branches_Log_QQ.png]

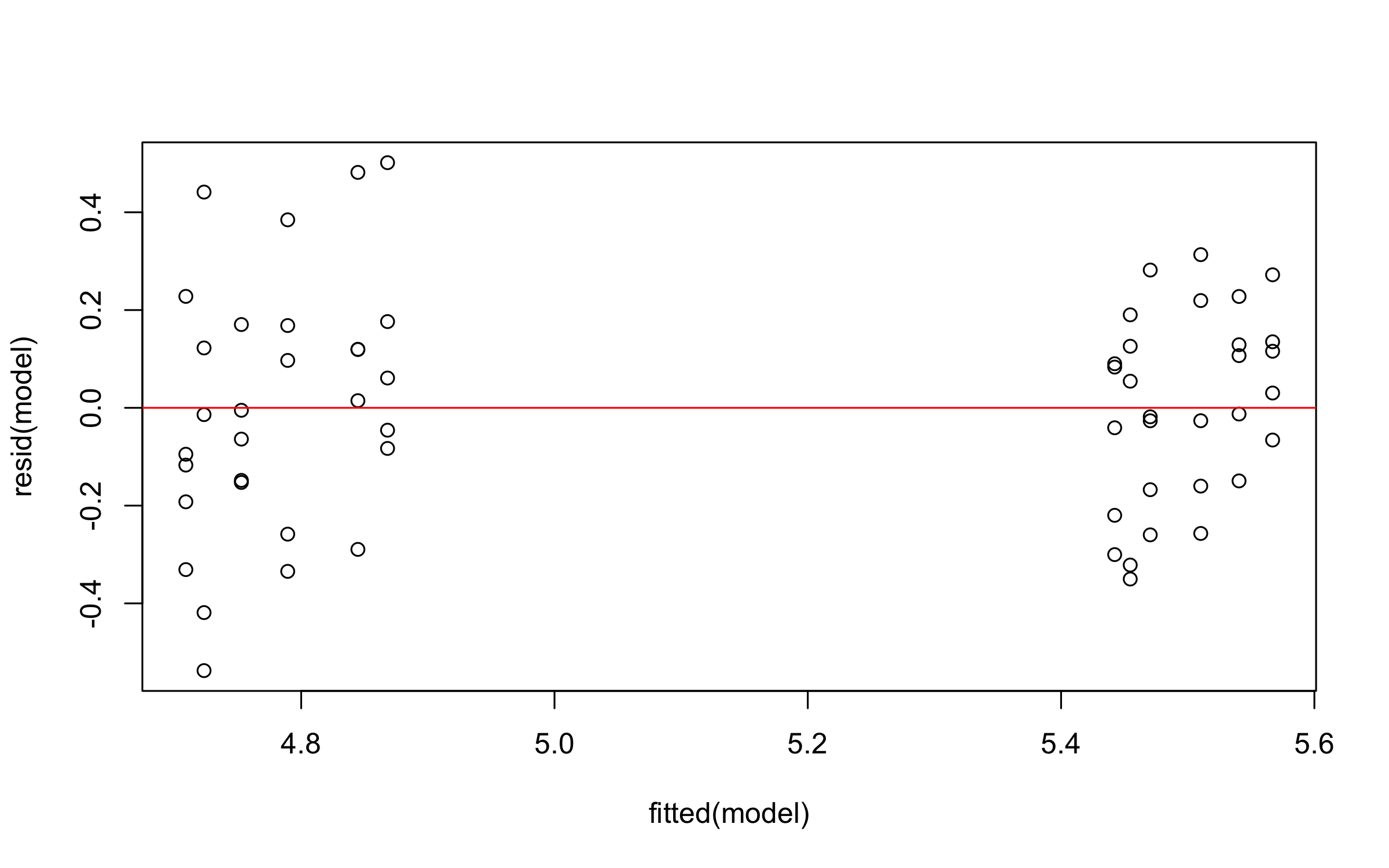

Supplement: Supplementary file 6 — Source Data [file 41467_2026_71014_MOESM6_ESM.zip › Source Data/Statistical Report/Diagnosis/Figure4C_Total_Branches_Log_ResidualFit.png]

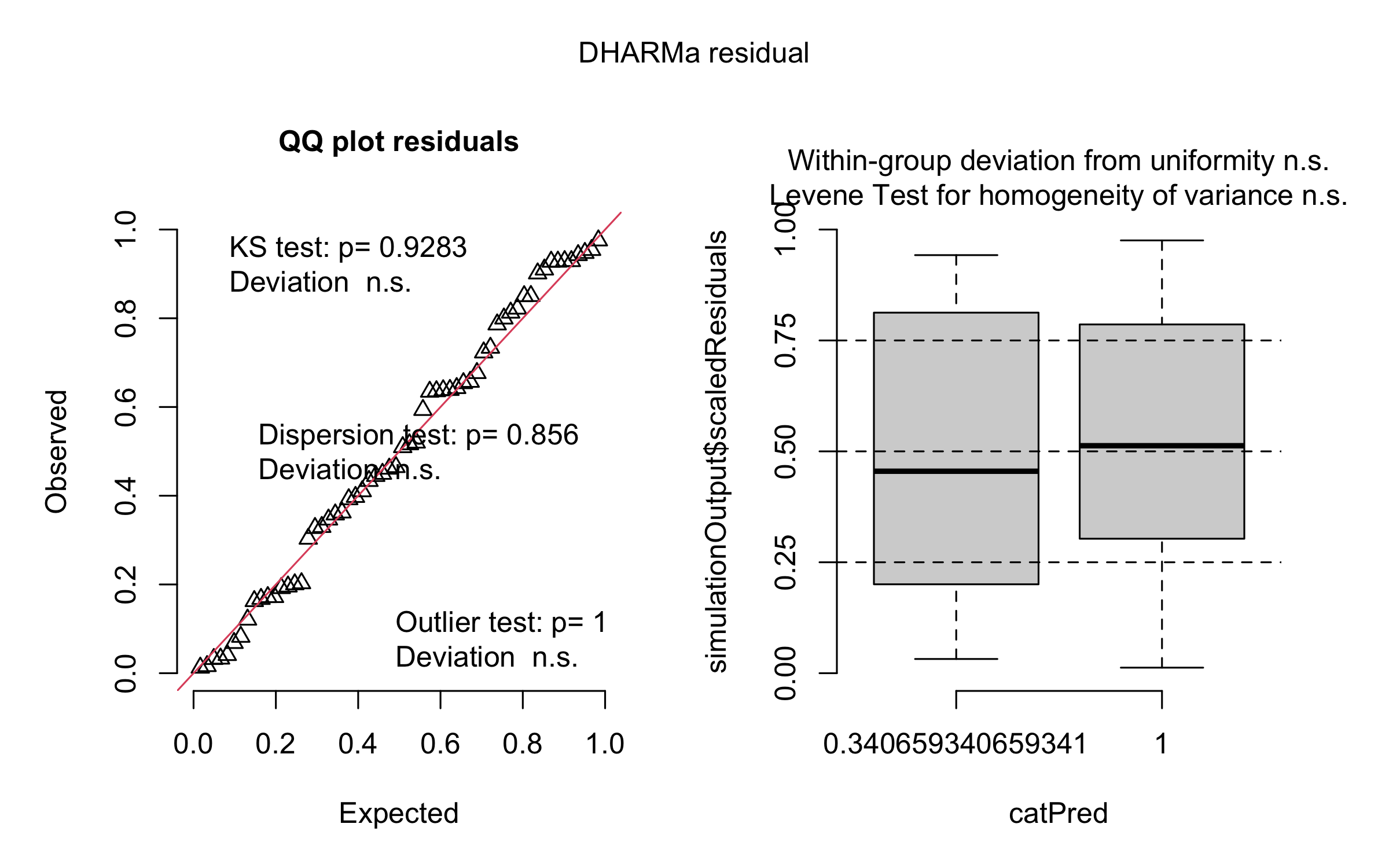

Supplement: Supplementary file 6 — Source Data [file 41467_2026_71014_MOESM6_ESM.zip › Source Data/Statistical Report/Diagnosis/Figure4C_Total_Intersections_DHARMa.png]

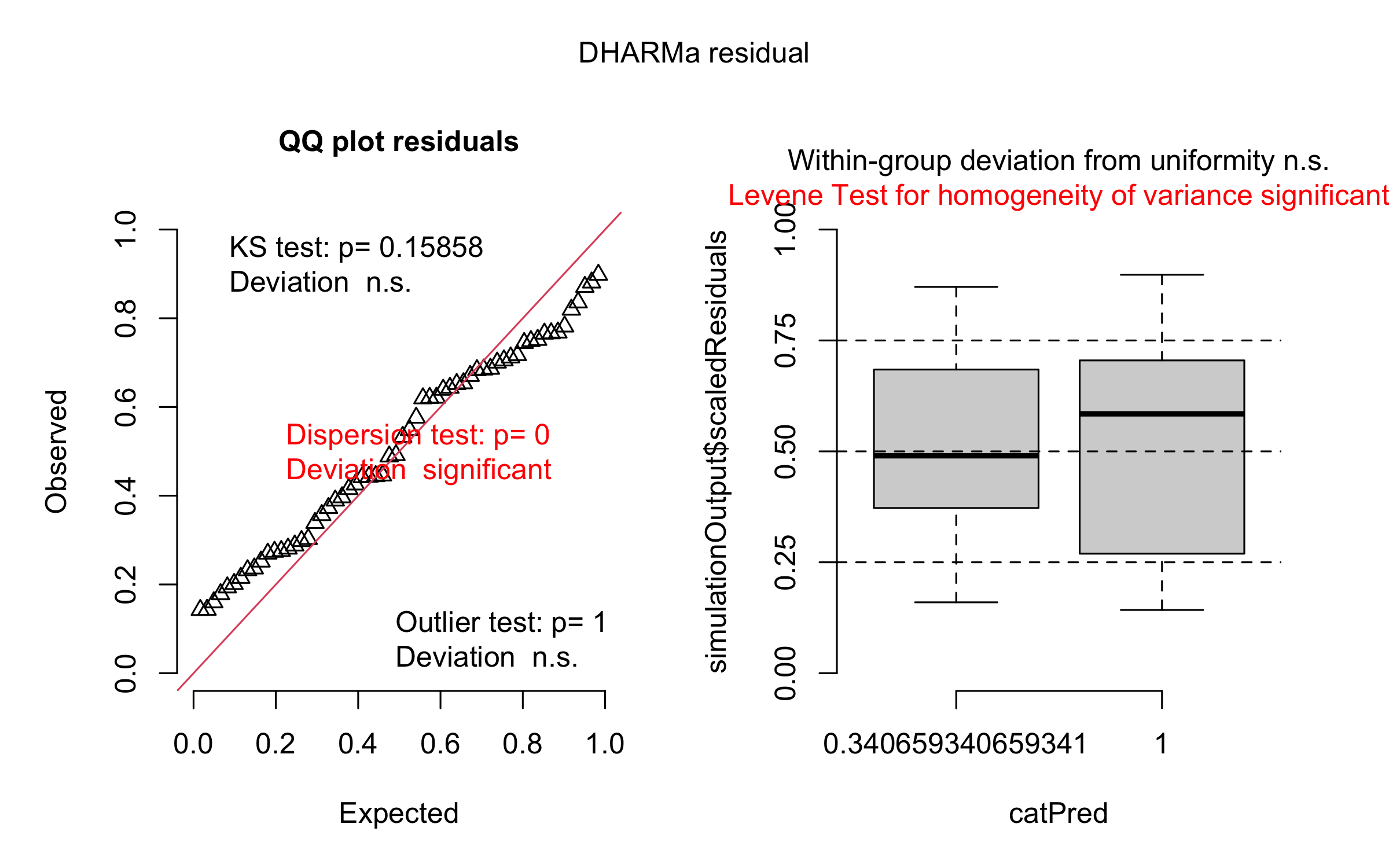

Supplement: Supplementary file 6 — Source Data [file 41467_2026_71014_MOESM6_ESM.zip › Source Data/Statistical Report/Diagnosis/Figure6A_Max_Intersection_DHARMa.png]

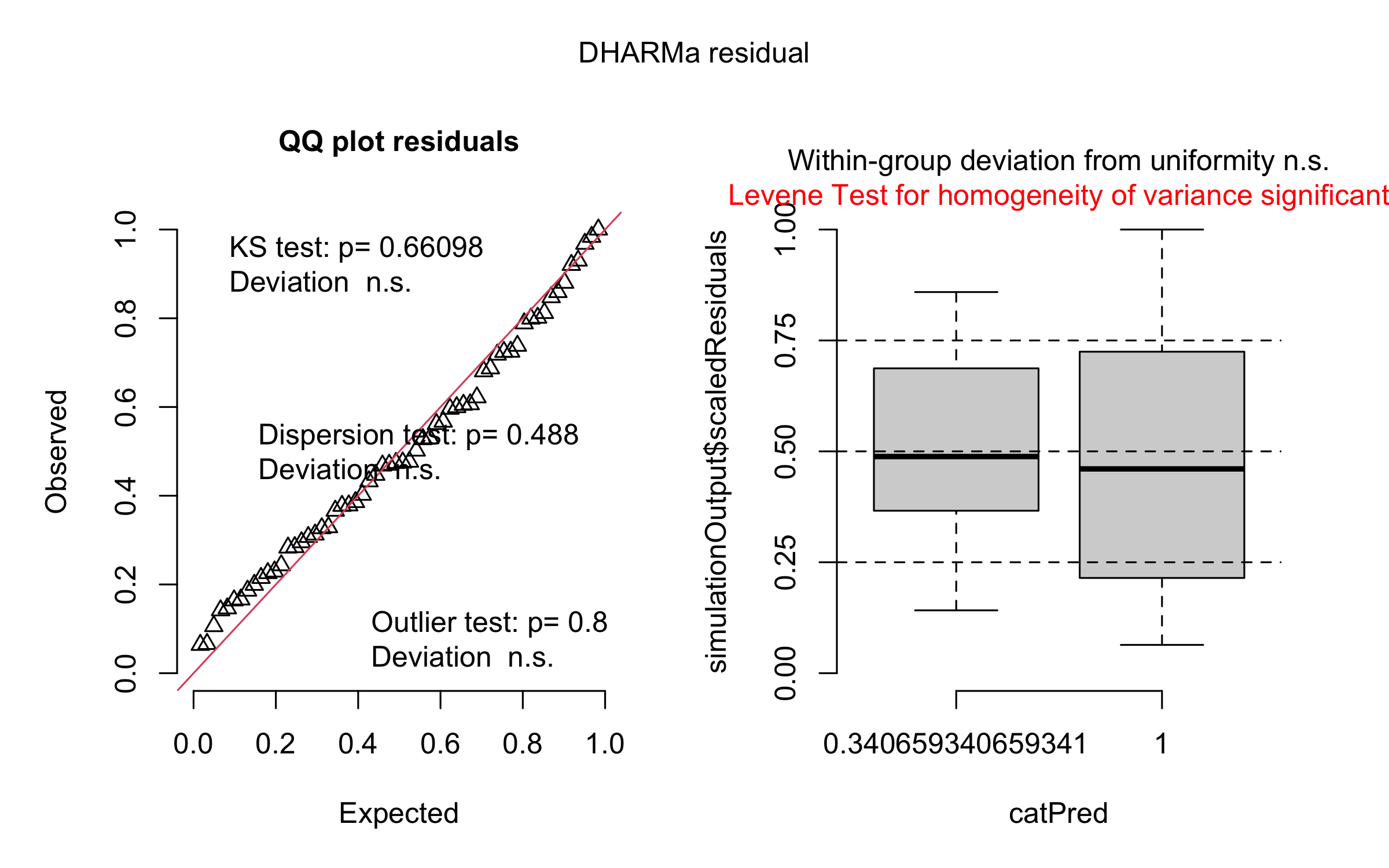

Supplement: Supplementary file 6 — Source Data [file 41467_2026_71014_MOESM6_ESM.zip › Source Data/Statistical Report/Diagnosis/Figure6A_Number_of_Branches_DHARMa.png]

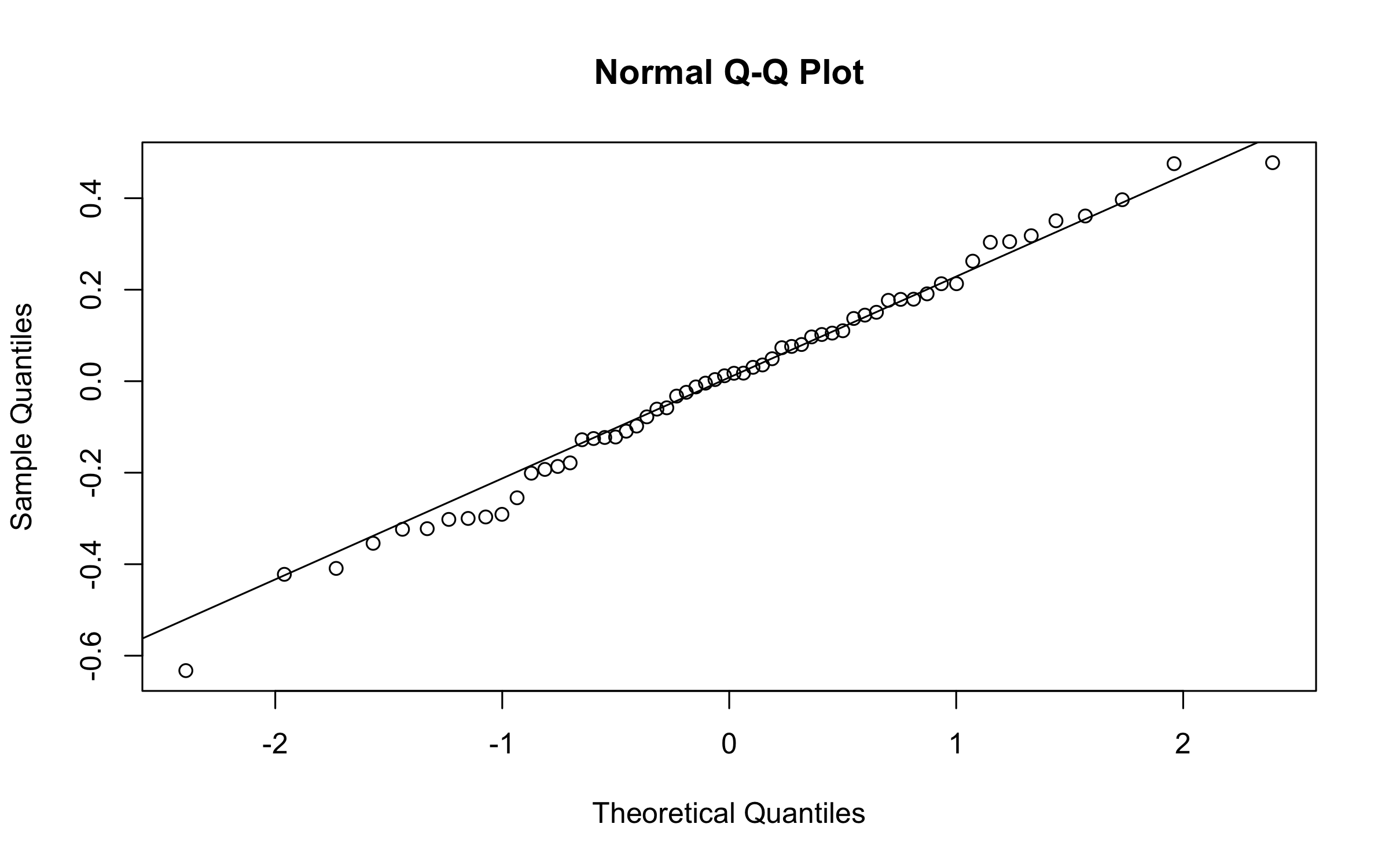

Supplement: Supplementary file 6 — Source Data [file 41467_2026_71014_MOESM6_ESM.zip › Source Data/Statistical Report/Diagnosis/Figure6A_Total_Branches_Log_QQ.png]

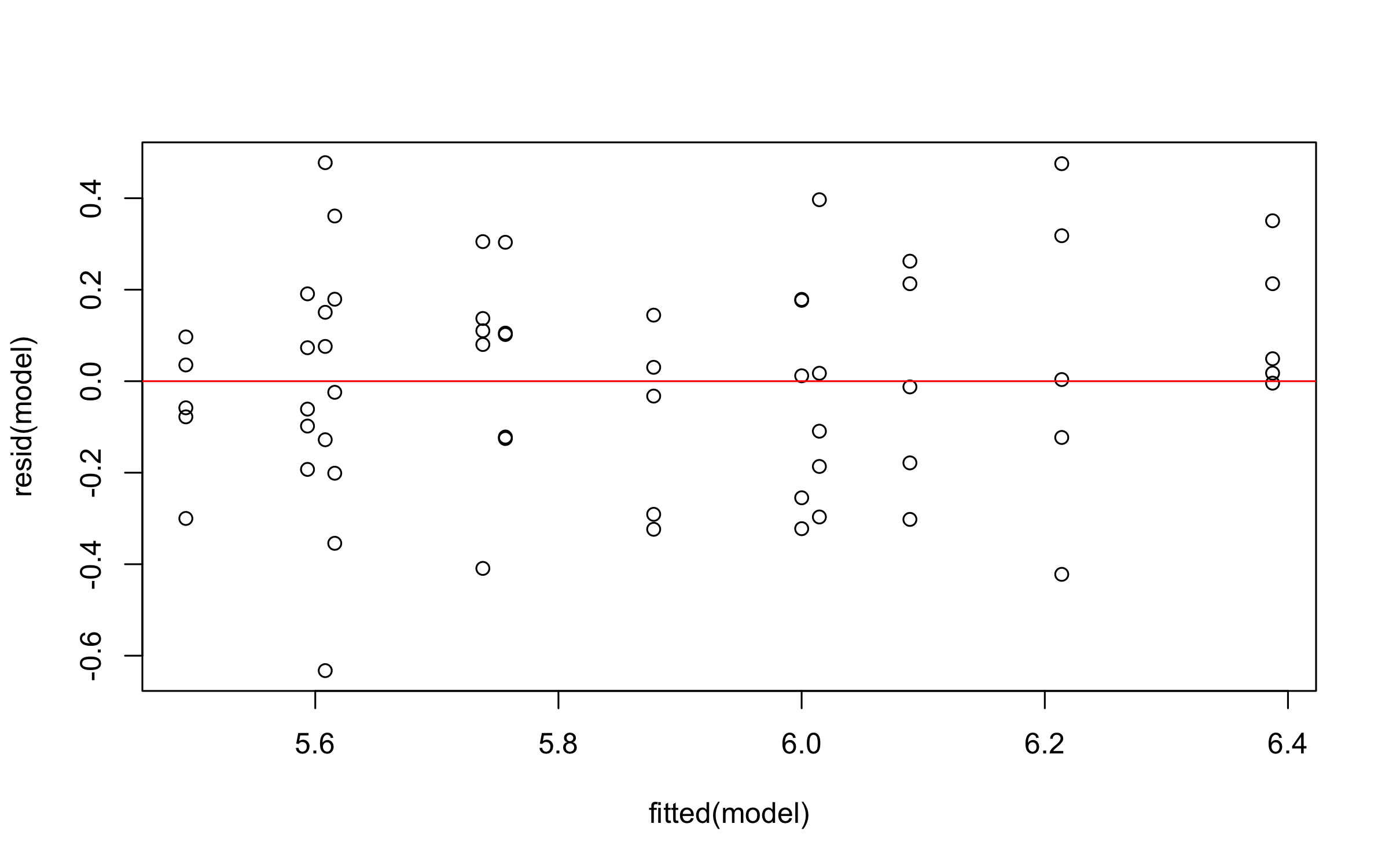

Supplement: Supplementary file 6 — Source Data [file 41467_2026_71014_MOESM6_ESM.zip › Source Data/Statistical Report/Diagnosis/Figure6A_Total_Branches_Log_ResidualFit.png]

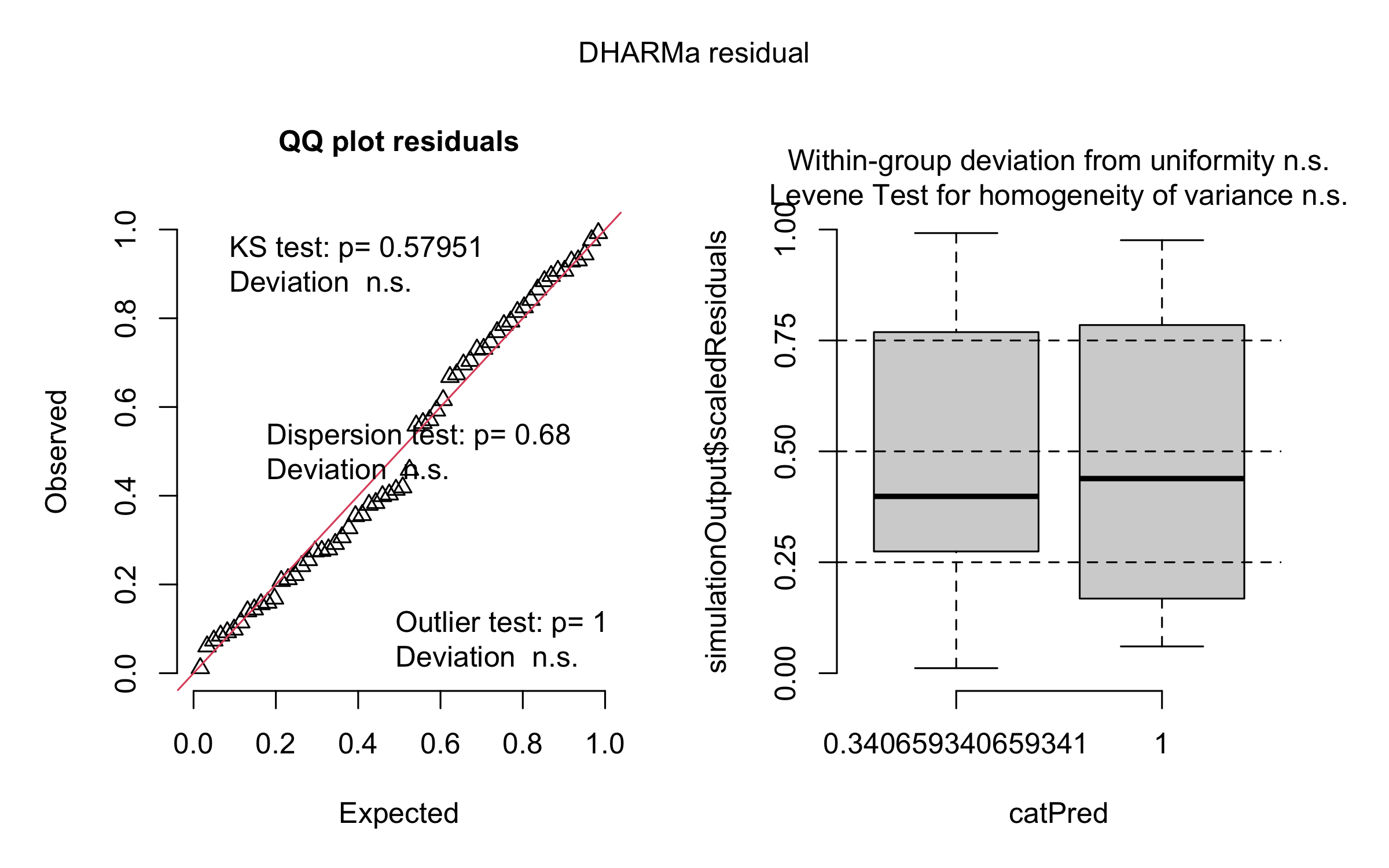

Supplement: Supplementary file 6 — Source Data [file 41467_2026_71014_MOESM6_ESM.zip › Source Data/Statistical Report/Diagnosis/Figure6A_Total_Intersections_DHARMa.png]

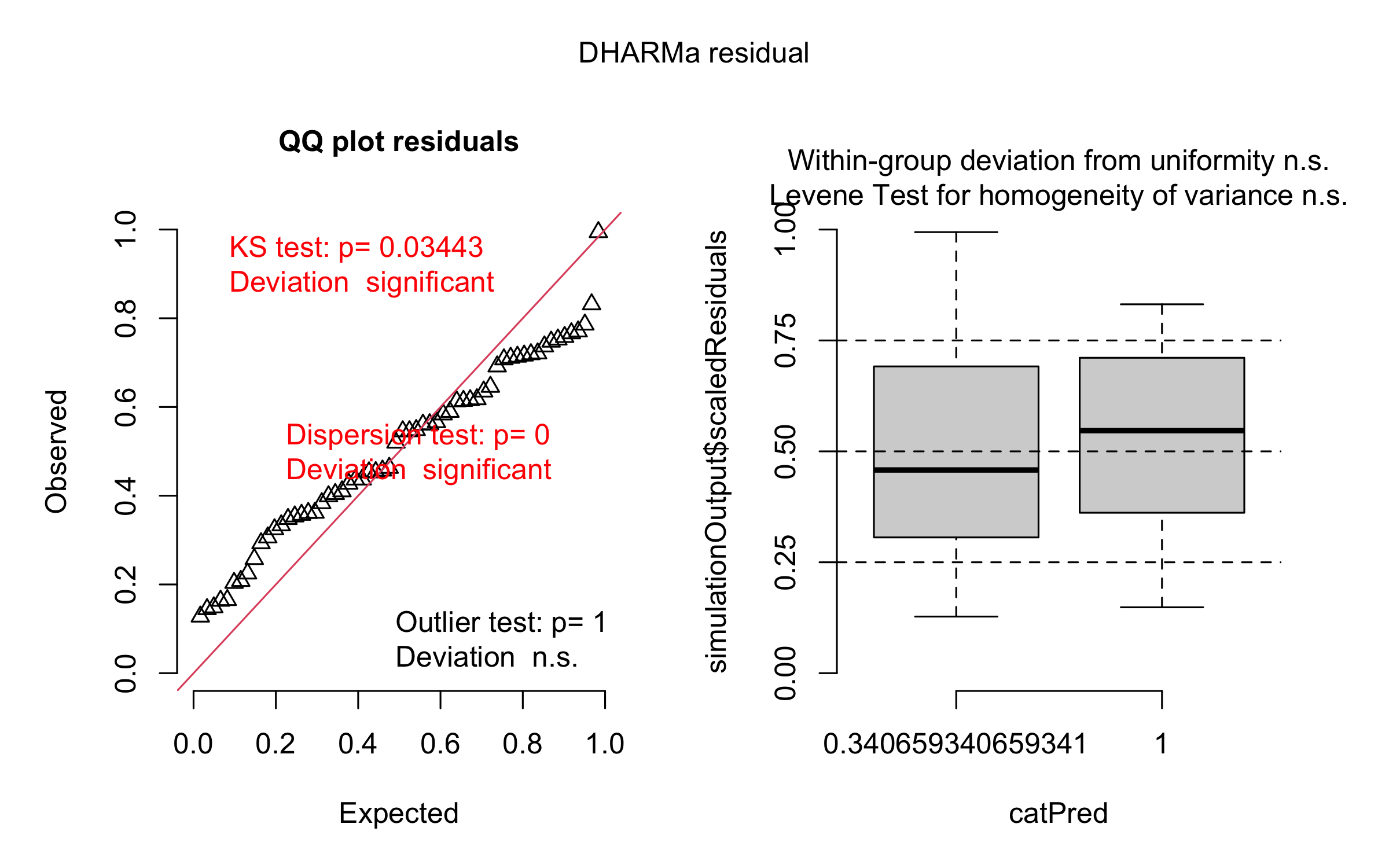

Supplement: Supplementary file 6 — Source Data [file 41467_2026_71014_MOESM6_ESM.zip › Source Data/Statistical Report/Diagnosis/Figure6B_Max_Intersection_DHARMa.png]

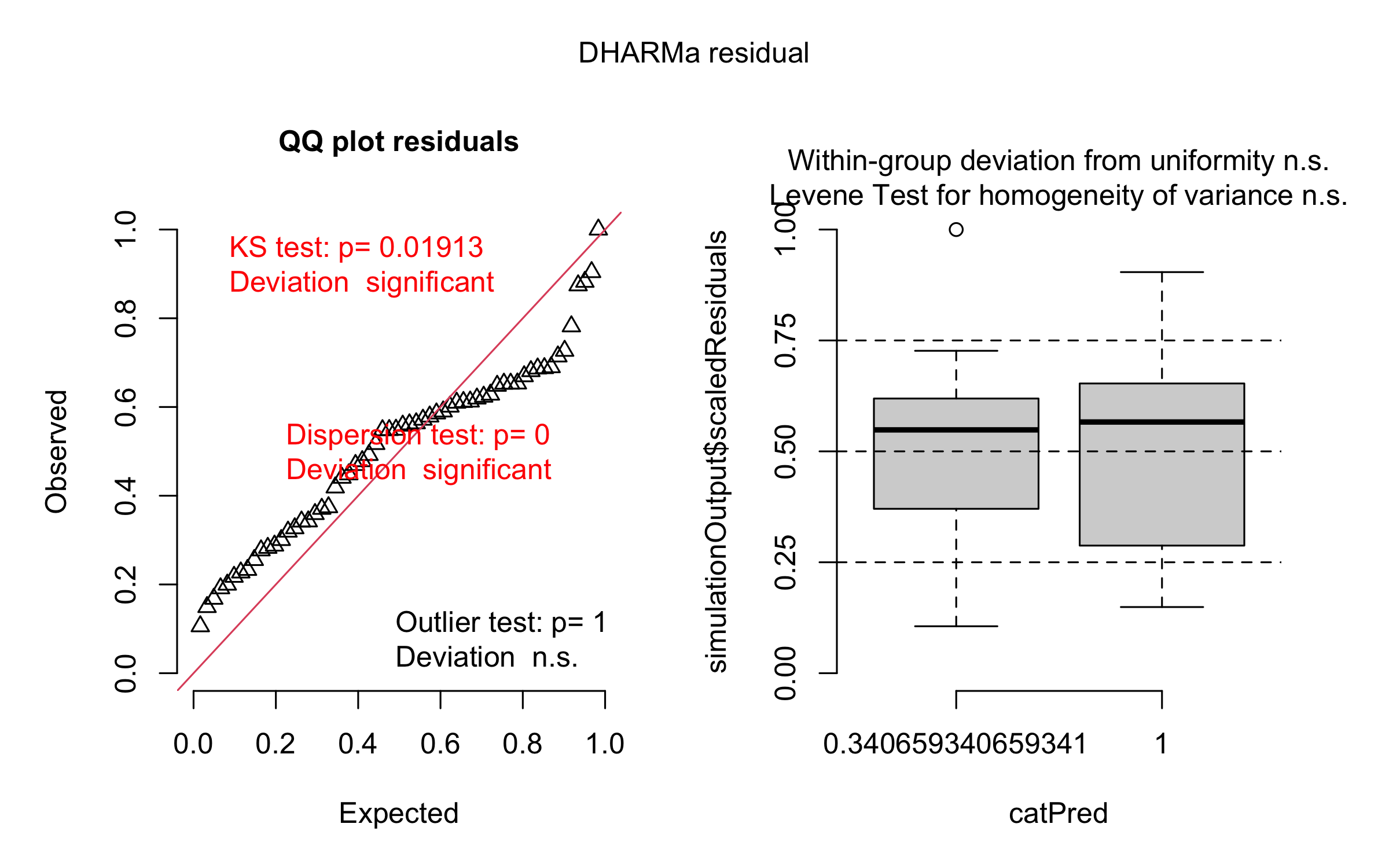

Supplement: Supplementary file 6 — Source Data [file 41467_2026_71014_MOESM6_ESM.zip › Source Data/Statistical Report/Diagnosis/Figure6B_Number_of_Branches_DHARMa.png]

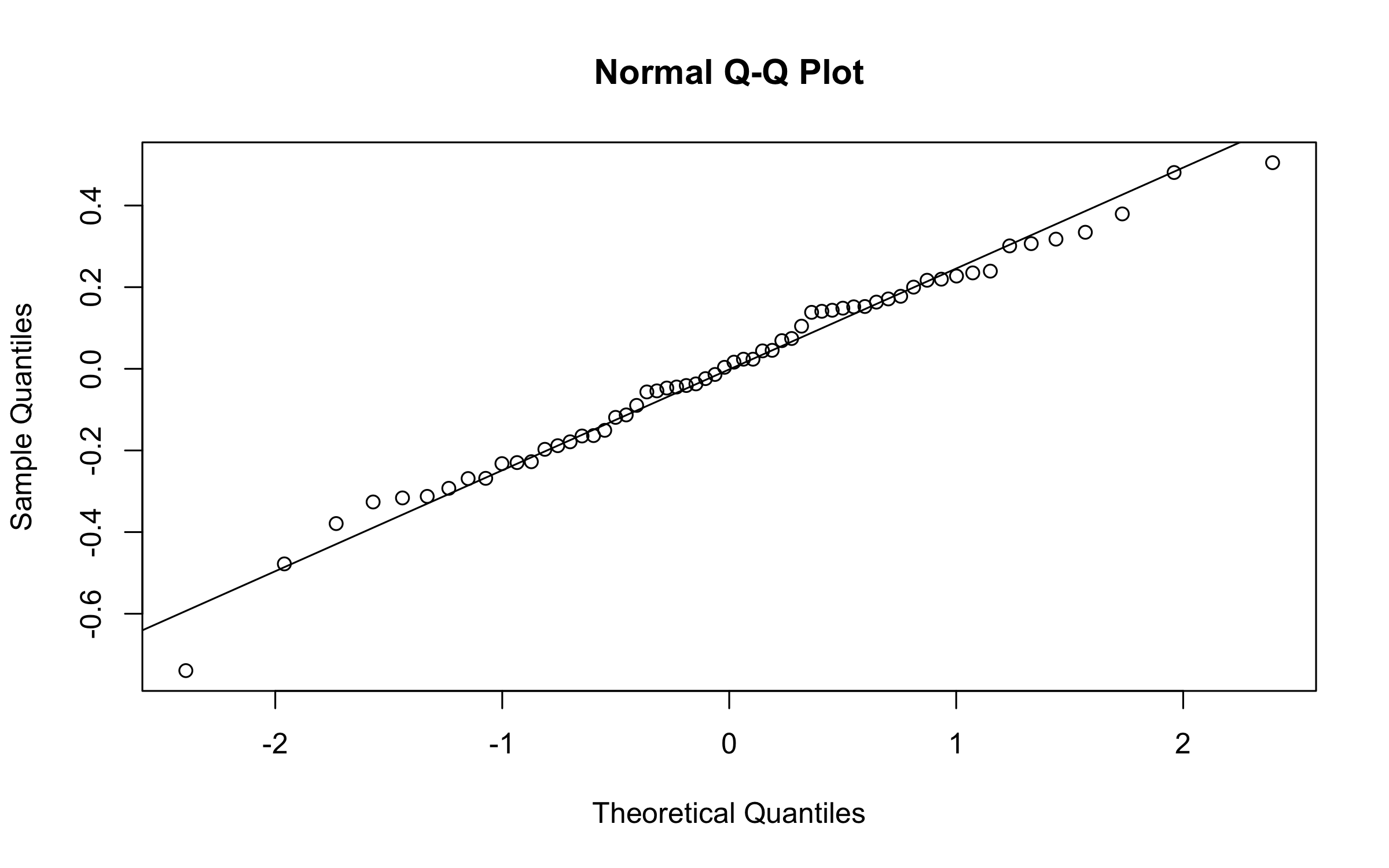

Supplement: Supplementary file 6 — Source Data [file 41467_2026_71014_MOESM6_ESM.zip › Source Data/Statistical Report/Diagnosis/Figure6B_Total_Branches_Log_QQ.png]

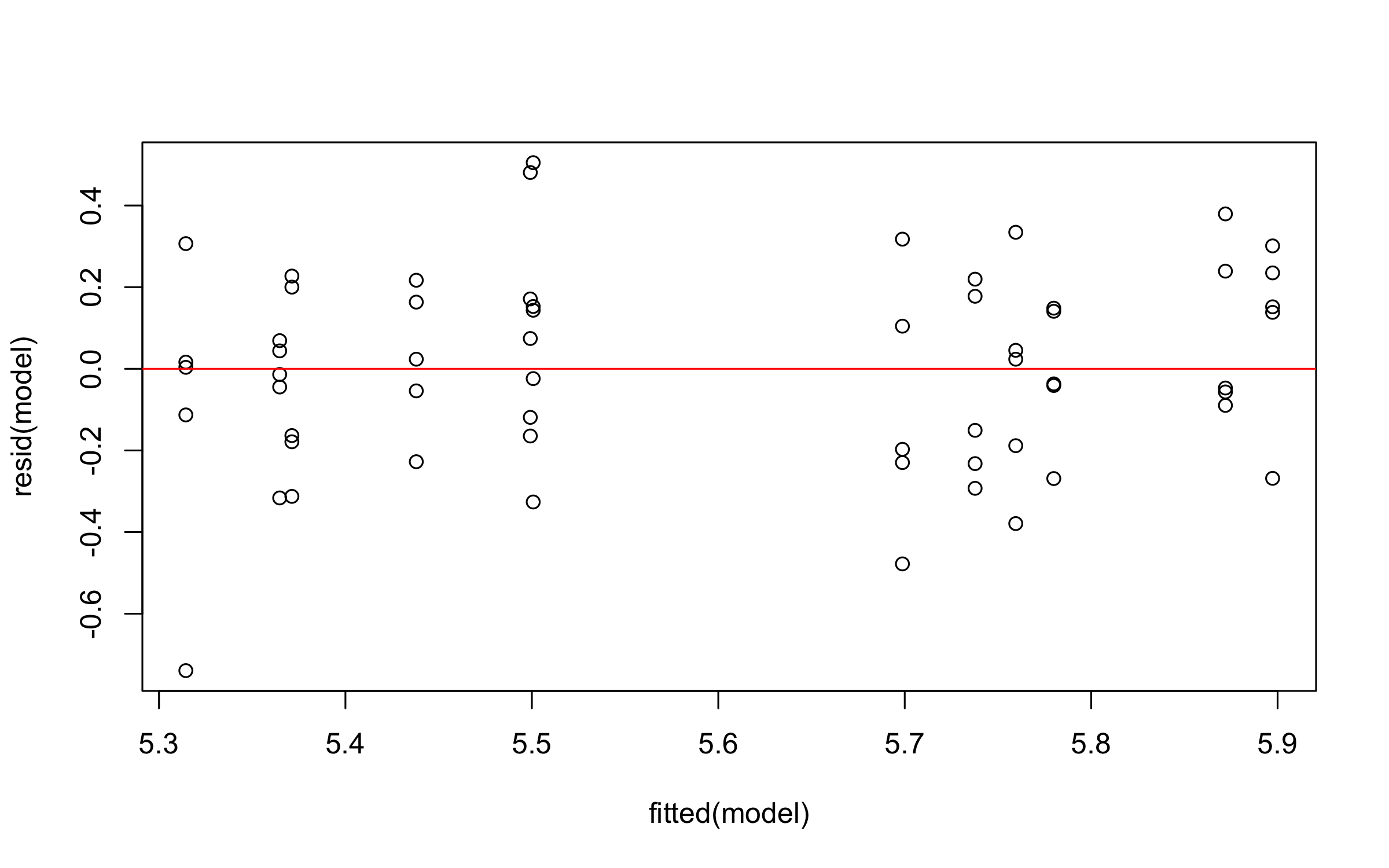

Supplement: Supplementary file 6 — Source Data [file 41467_2026_71014_MOESM6_ESM.zip › Source Data/Statistical Report/Diagnosis/Figure6B_Total_Branches_Log_ResidualFit.png]

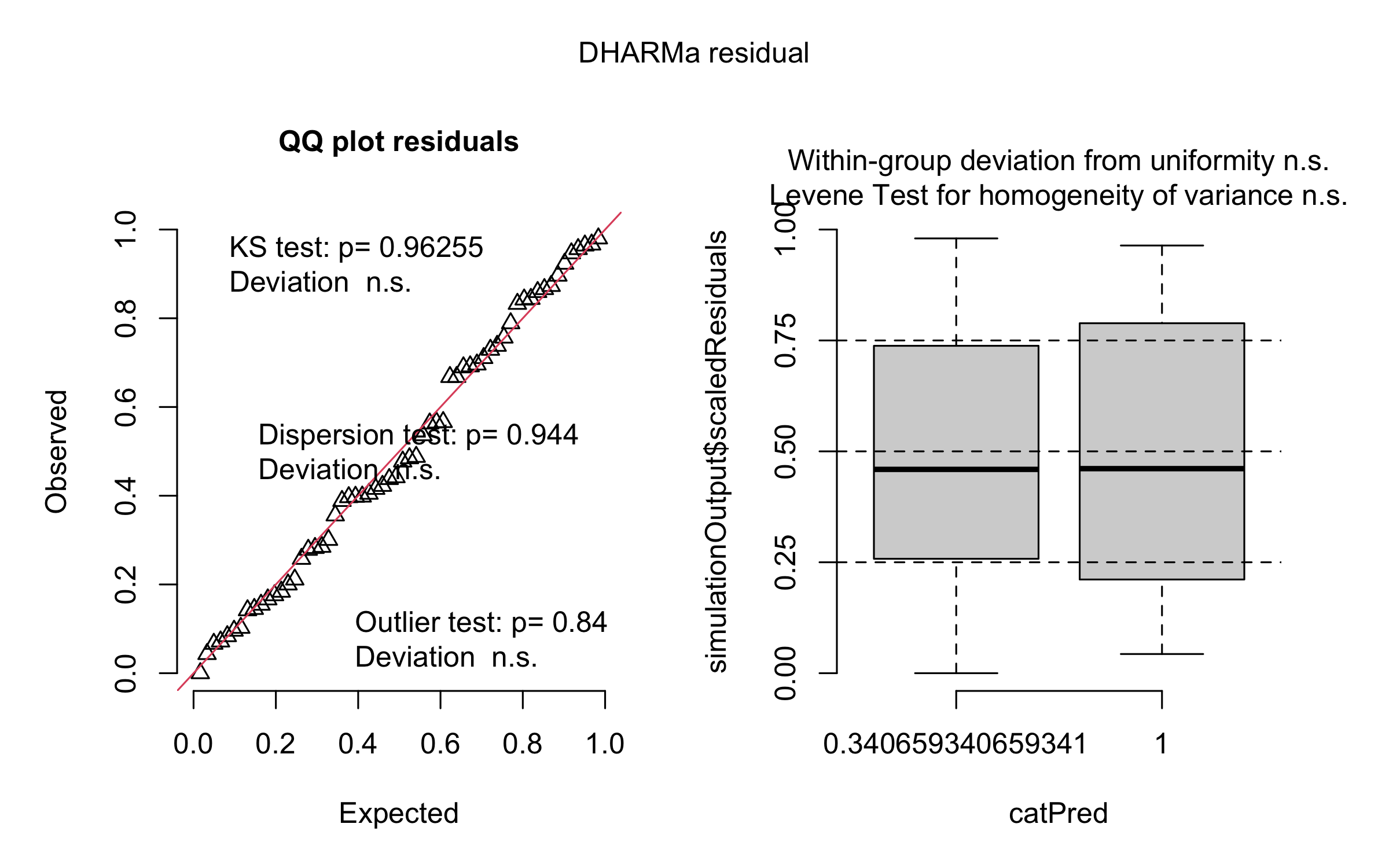

Supplement: Supplementary file 6 — Source Data [file 41467_2026_71014_MOESM6_ESM.zip › Source Data/Statistical Report/Diagnosis/Figure6B_Total_Intersections_DHARMa.png]

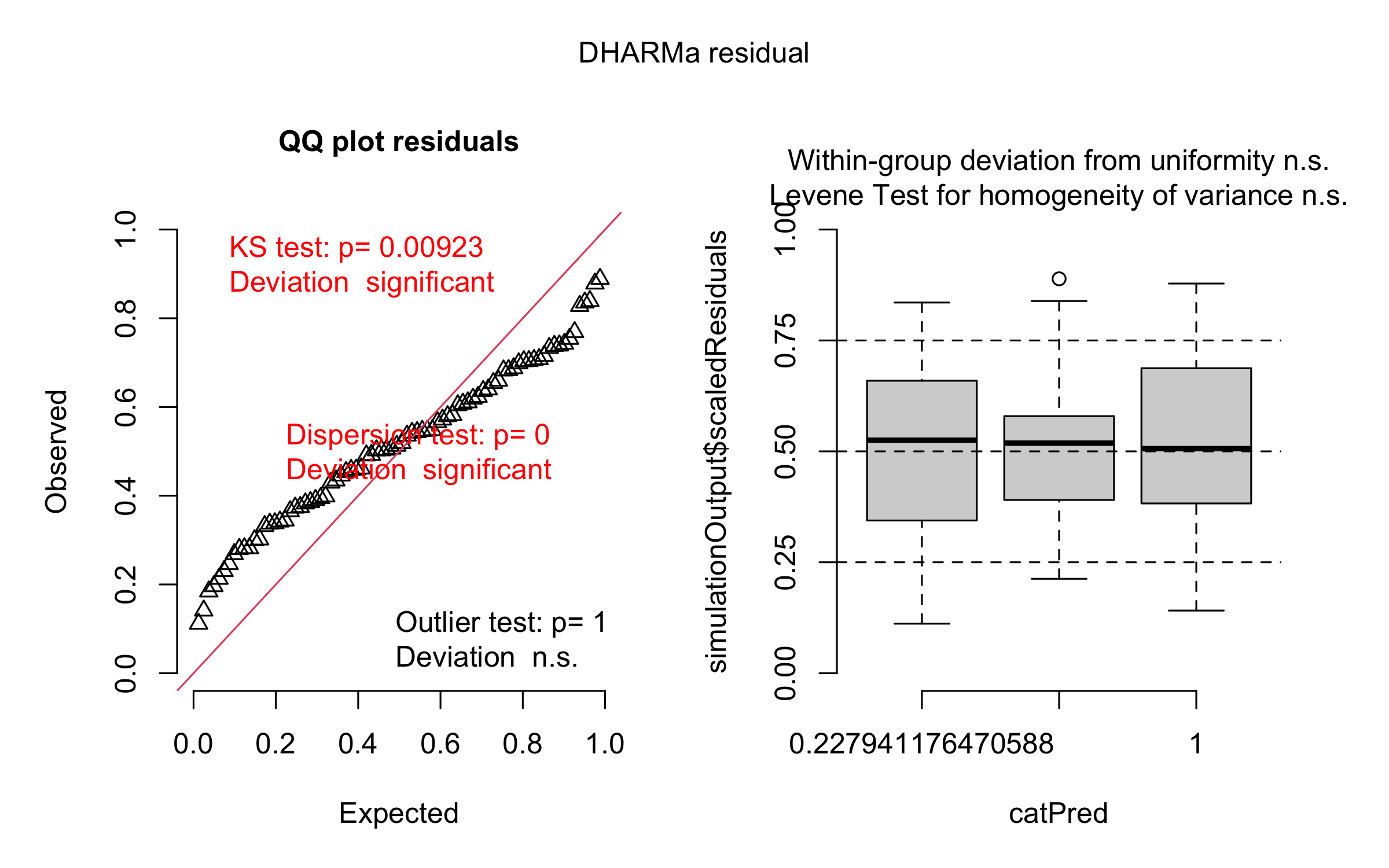

Supplement: Supplementary file 6 — Source Data [file 41467_2026_71014_MOESM6_ESM.zip › Source Data/Statistical Report/Diagnosis/FigureS3B_Max_Intersection_DHARMa.png]

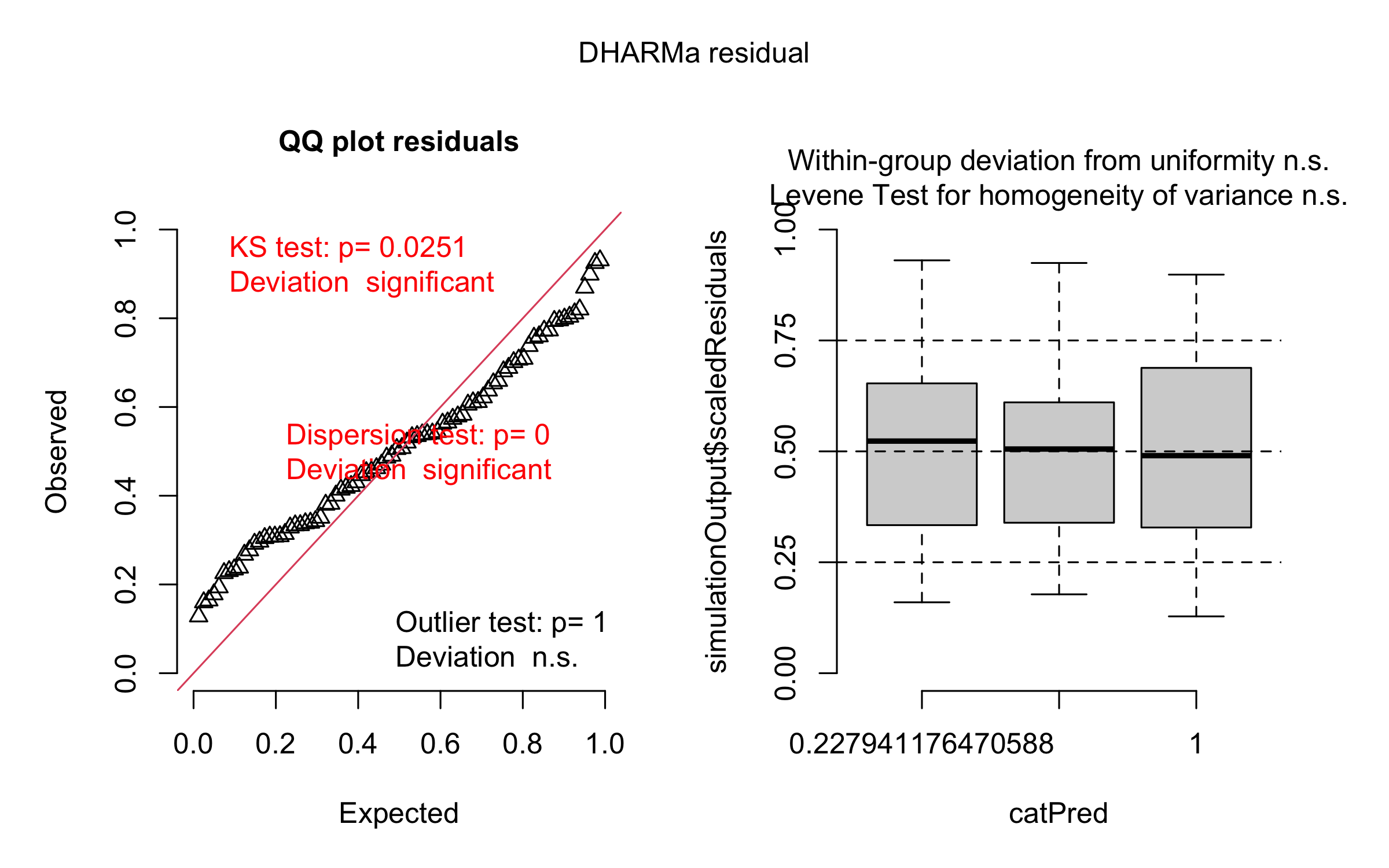

Supplement: Supplementary file 6 — Source Data [file 41467_2026_71014_MOESM6_ESM.zip › Source Data/Statistical Report/Diagnosis/FigureS3B_Number_of_Branches_DHARMa.png]

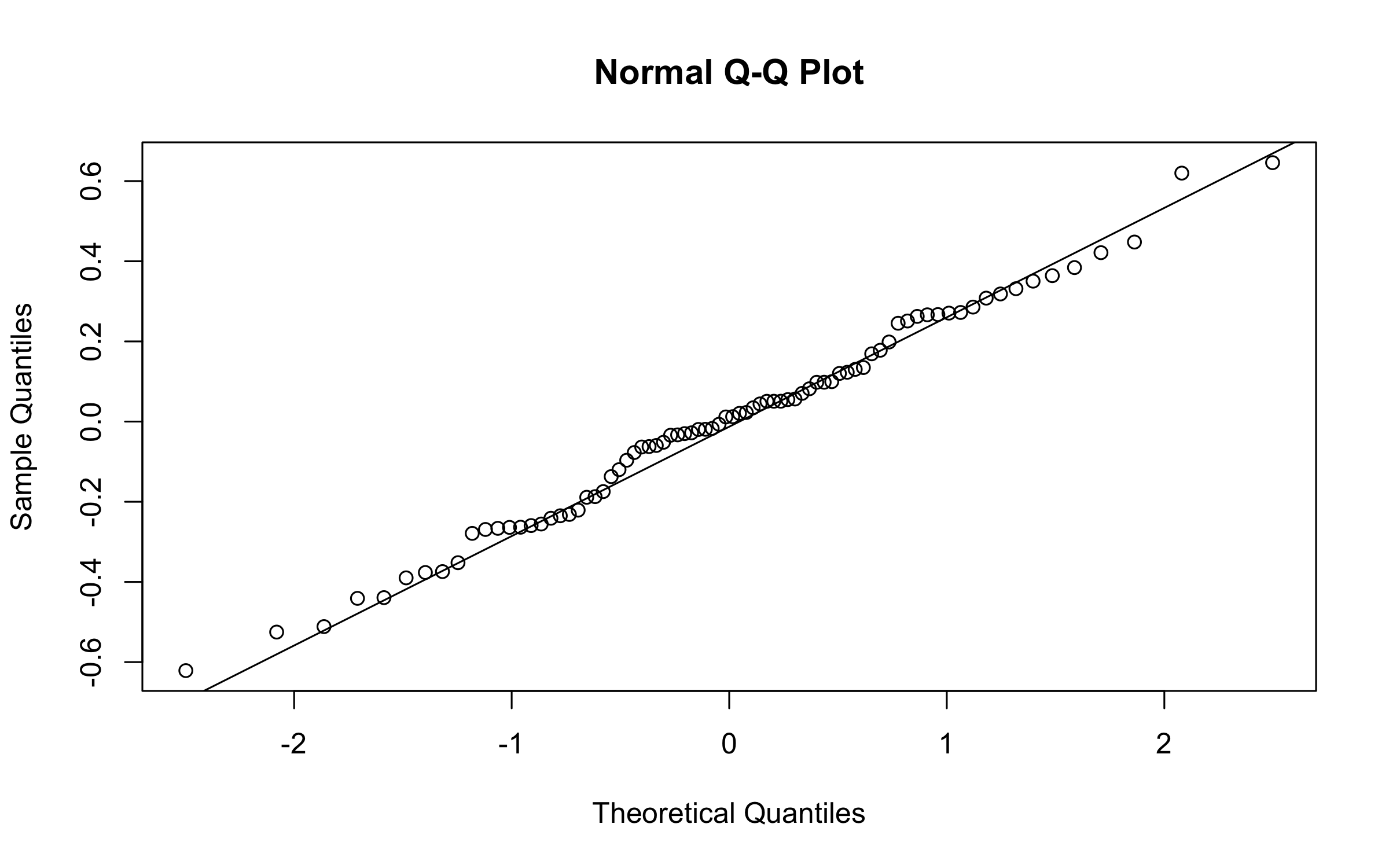

Supplement: Supplementary file 6 — Source Data [file 41467_2026_71014_MOESM6_ESM.zip › Source Data/Statistical Report/Diagnosis/FigureS3B_Total_Branches_Log_QQ.png]

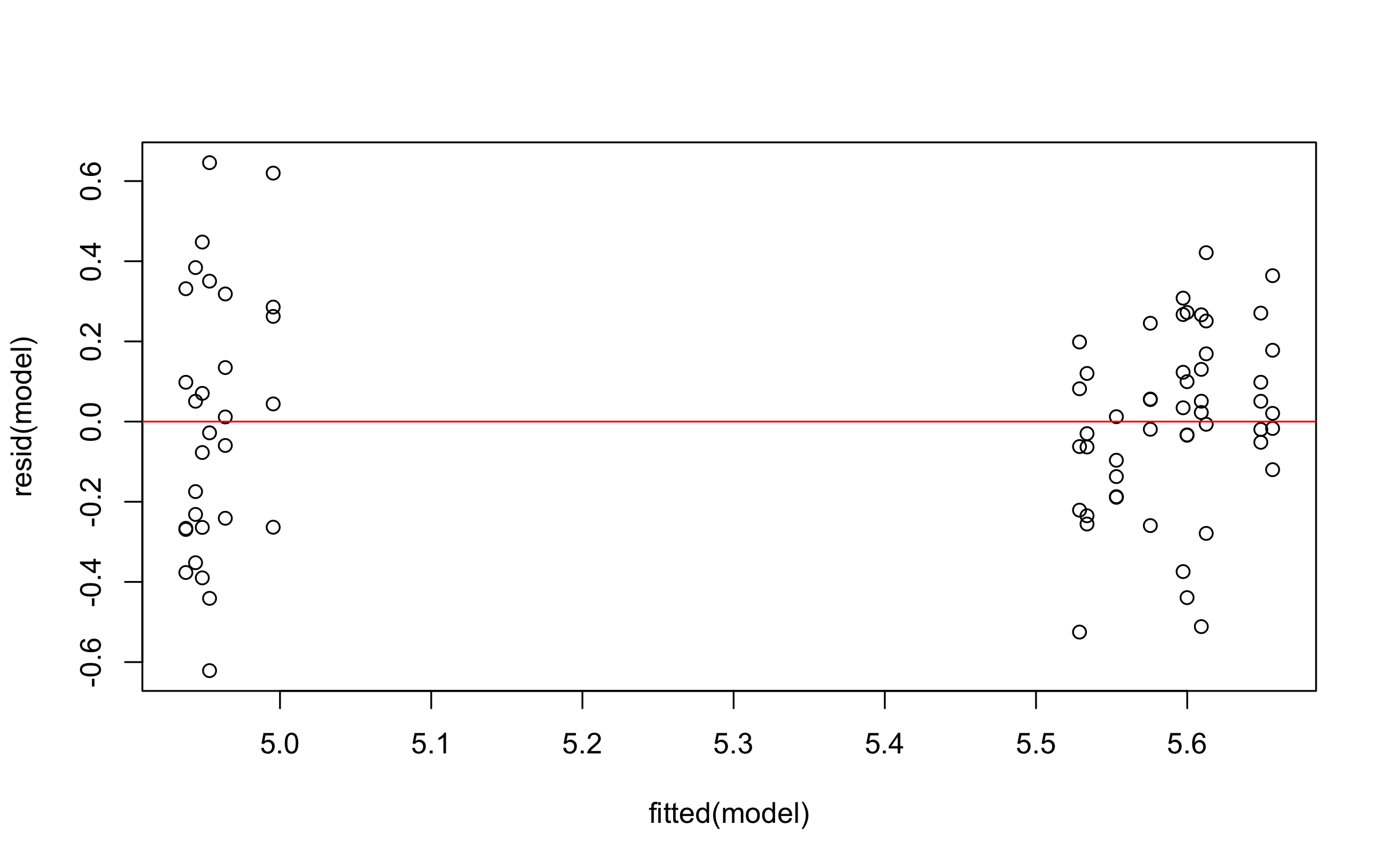

Supplement: Supplementary file 6 — Source Data [file 41467_2026_71014_MOESM6_ESM.zip › Source Data/Statistical Report/Diagnosis/FigureS3B_Total_Branches_Log_ResidualFit.png]

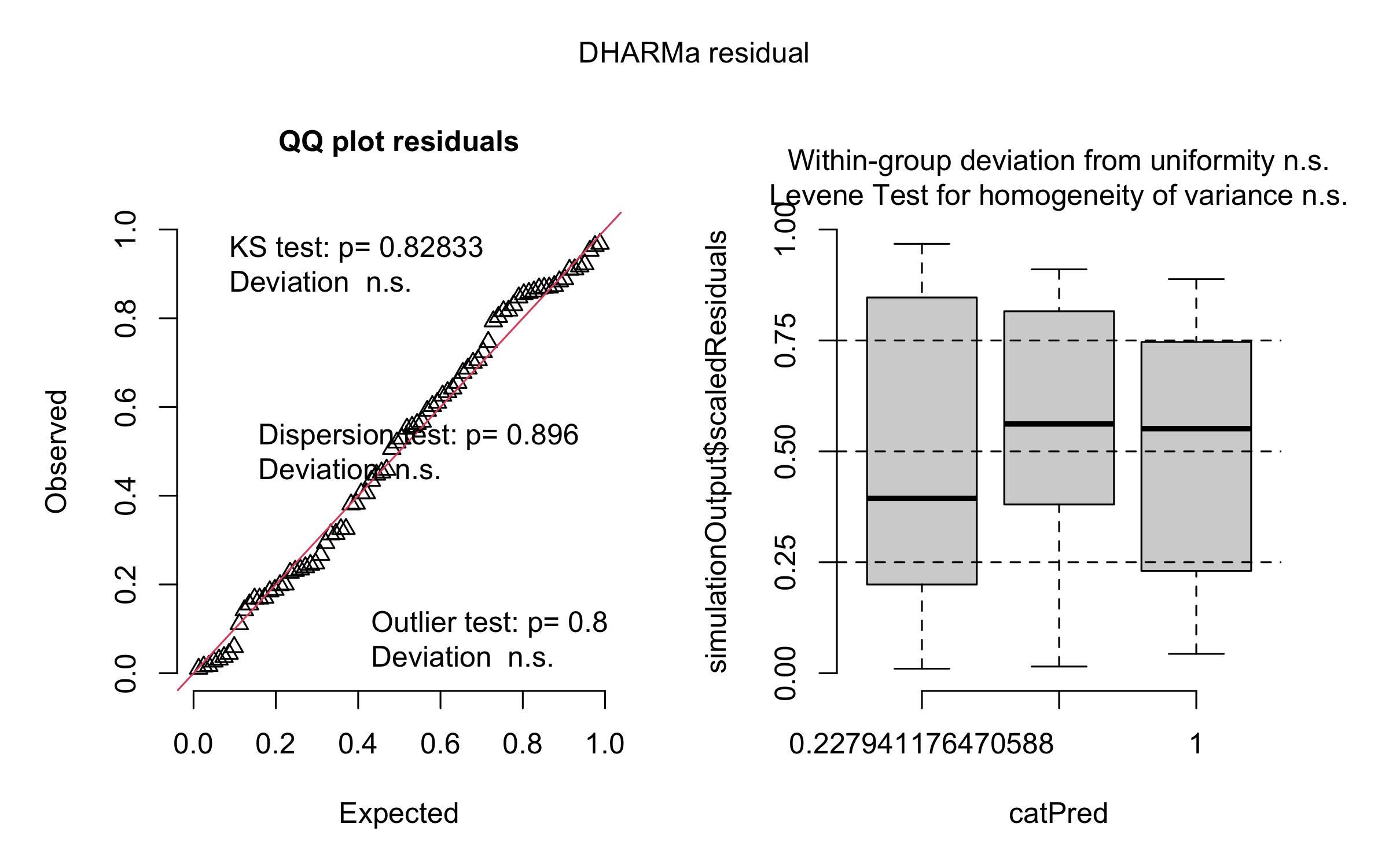

Supplement: Supplementary file 6 — Source Data [file 41467_2026_71014_MOESM6_ESM.zip › Source Data/Statistical Report/Diagnosis/FigureS3B_Total_Intersections_DHARMa.png]

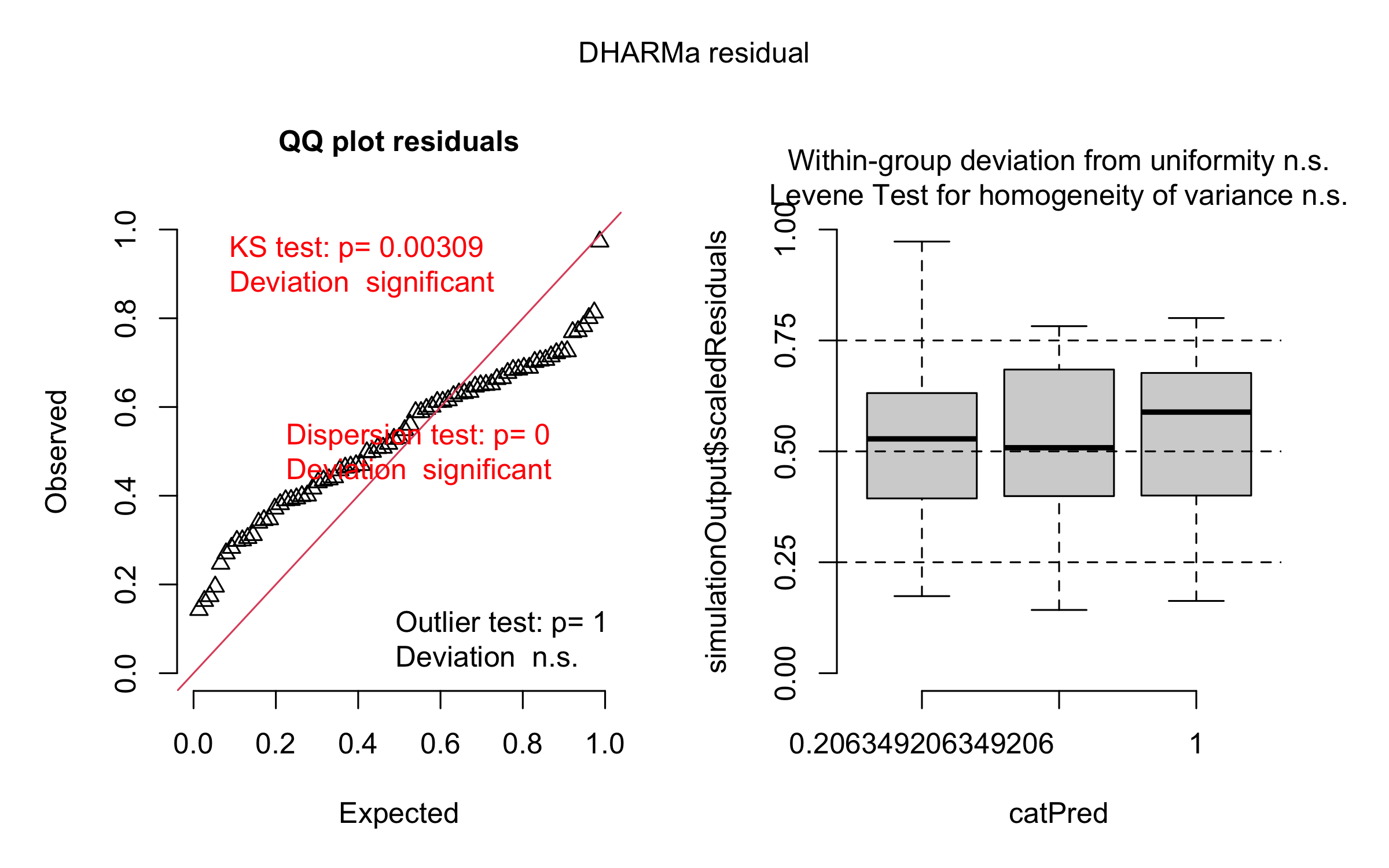

Supplement: Supplementary file 6 — Source Data [file 41467_2026_71014_MOESM6_ESM.zip › Source Data/Statistical Report/Diagnosis/FigureS3D_Max_Intersection_DHARMa.png]

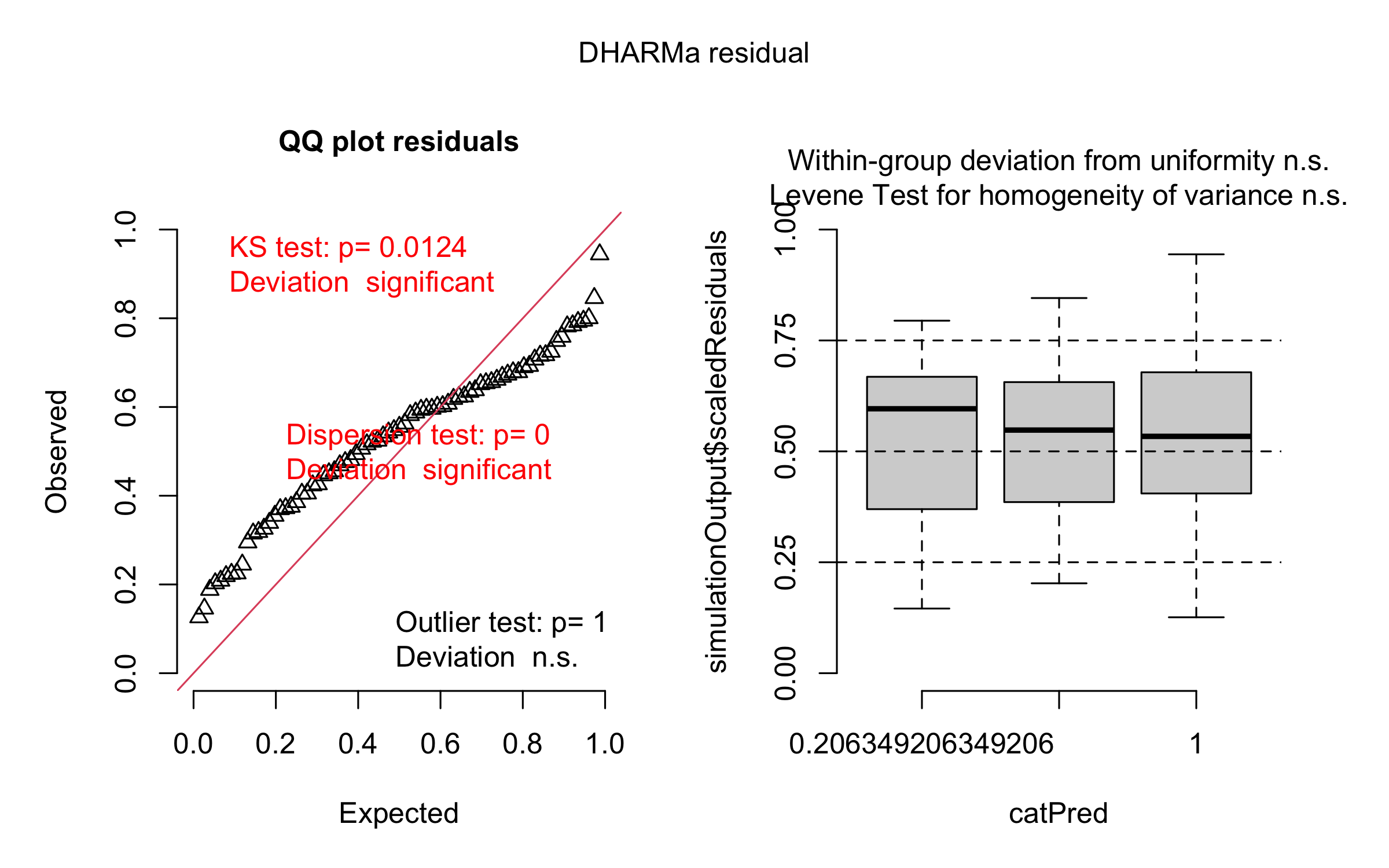

Supplement: Supplementary file 6 — Source Data [file 41467_2026_71014_MOESM6_ESM.zip › Source Data/Statistical Report/Diagnosis/FigureS3D_Number_of_Branches_DHARMa.png]

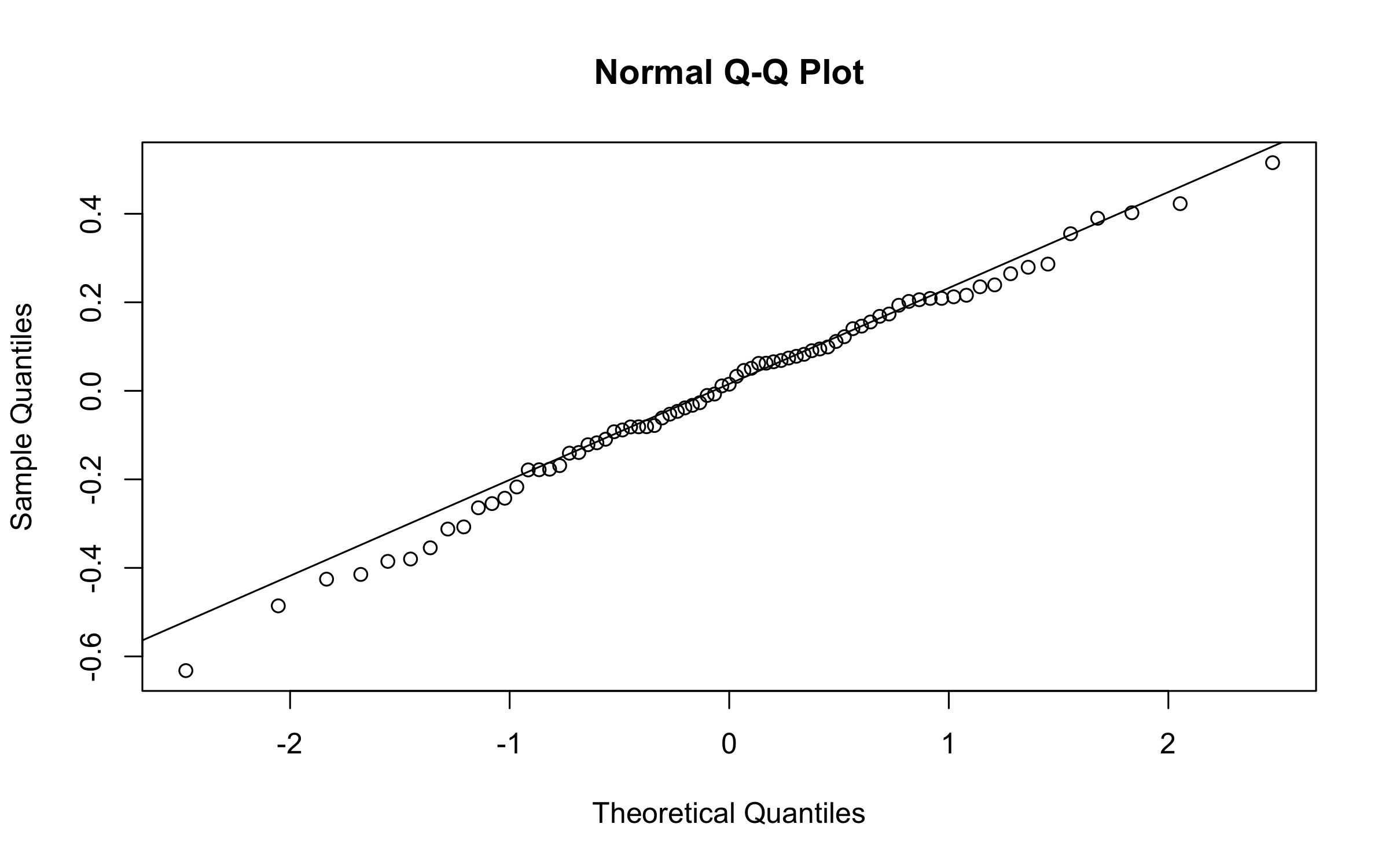

Supplement: Supplementary file 6 — Source Data [file 41467_2026_71014_MOESM6_ESM.zip › Source Data/Statistical Report/Diagnosis/FigureS3D_Total_Branches_Log_QQ.png]

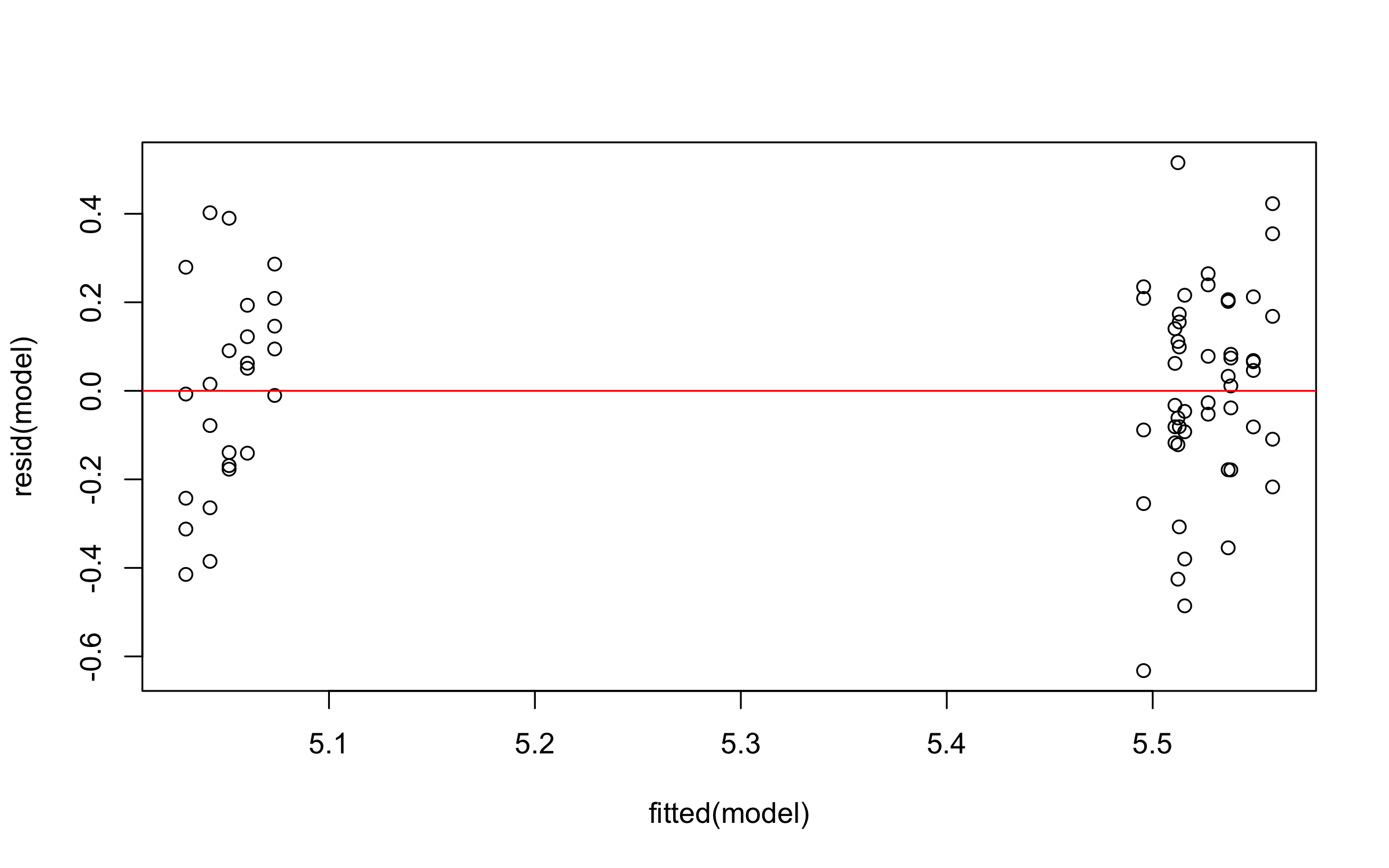

Supplement: Supplementary file 6 — Source Data [file 41467_2026_71014_MOESM6_ESM.zip › Source Data/Statistical Report/Diagnosis/FigureS3D_Total_Branches_Log_ResidualFit.png]

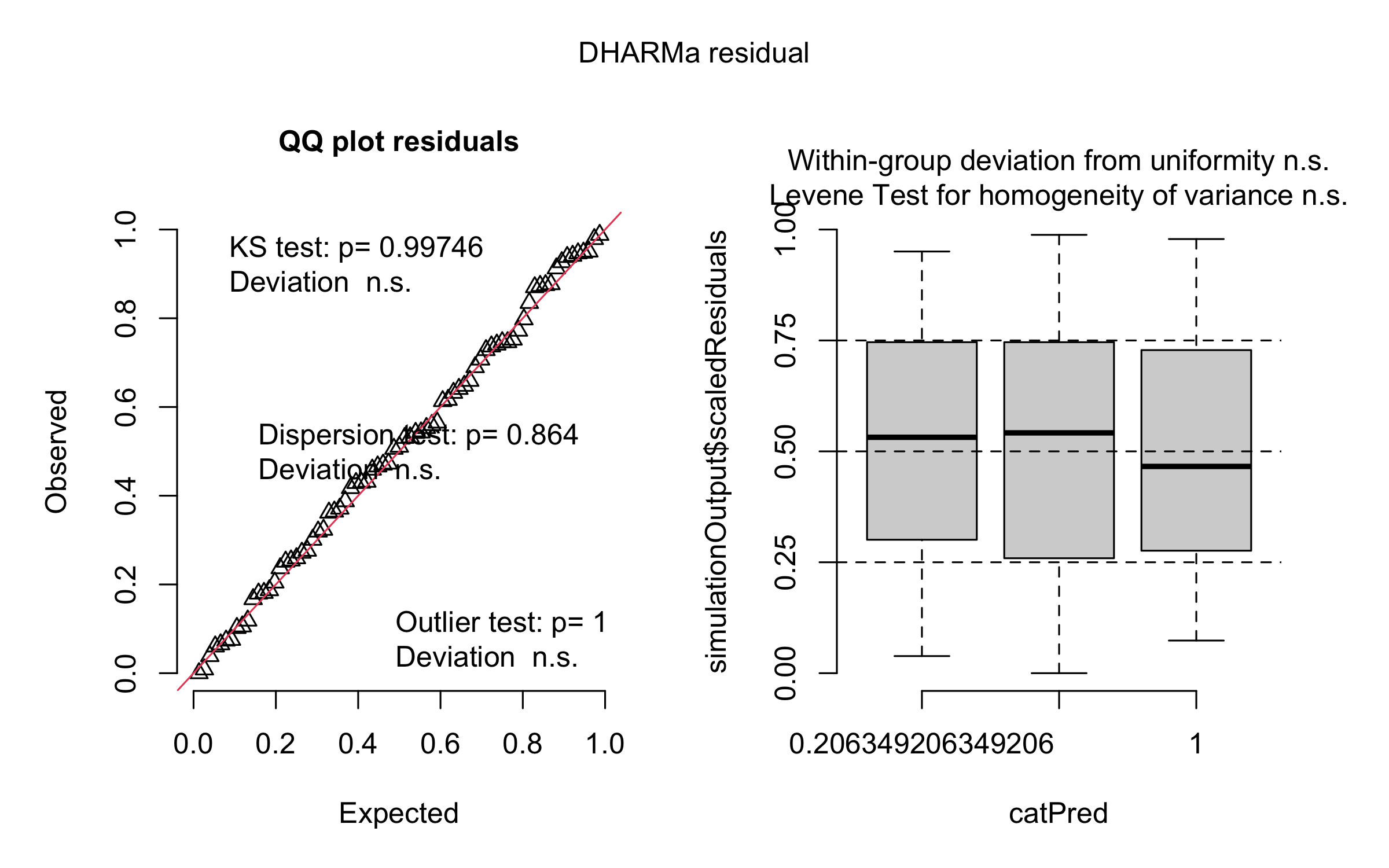

Supplement: Supplementary file 6 — Source Data [file 41467_2026_71014_MOESM6_ESM.zip › Source Data/Statistical Report/Diagnosis/FigureS3D_Total_Intersections_DHARMa.png]

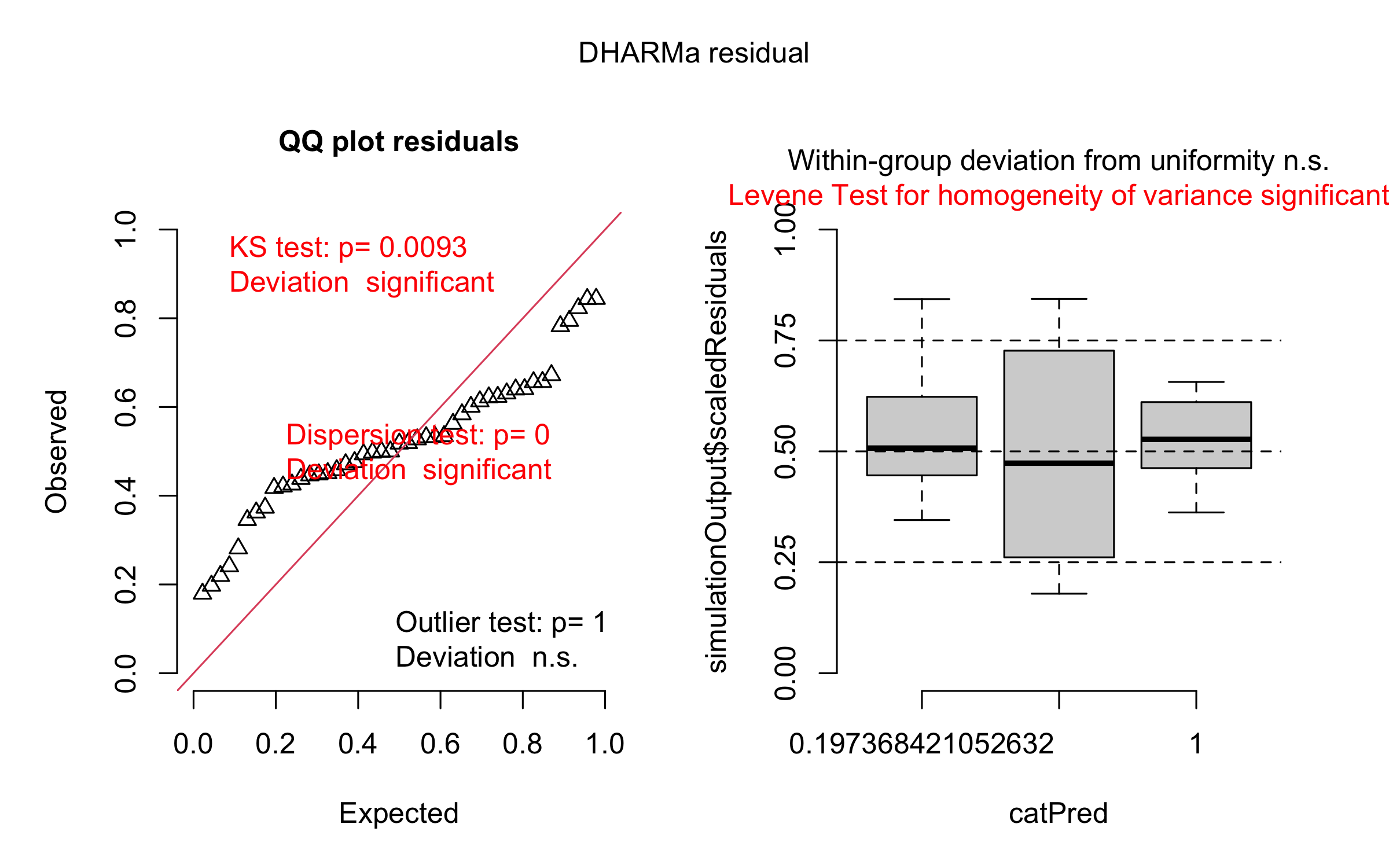

Supplement: Supplementary file 6 — Source Data [file 41467_2026_71014_MOESM6_ESM.zip › Source Data/Statistical Report/Diagnosis/FigureS6D_Max_Intersection_DHARMa.png]

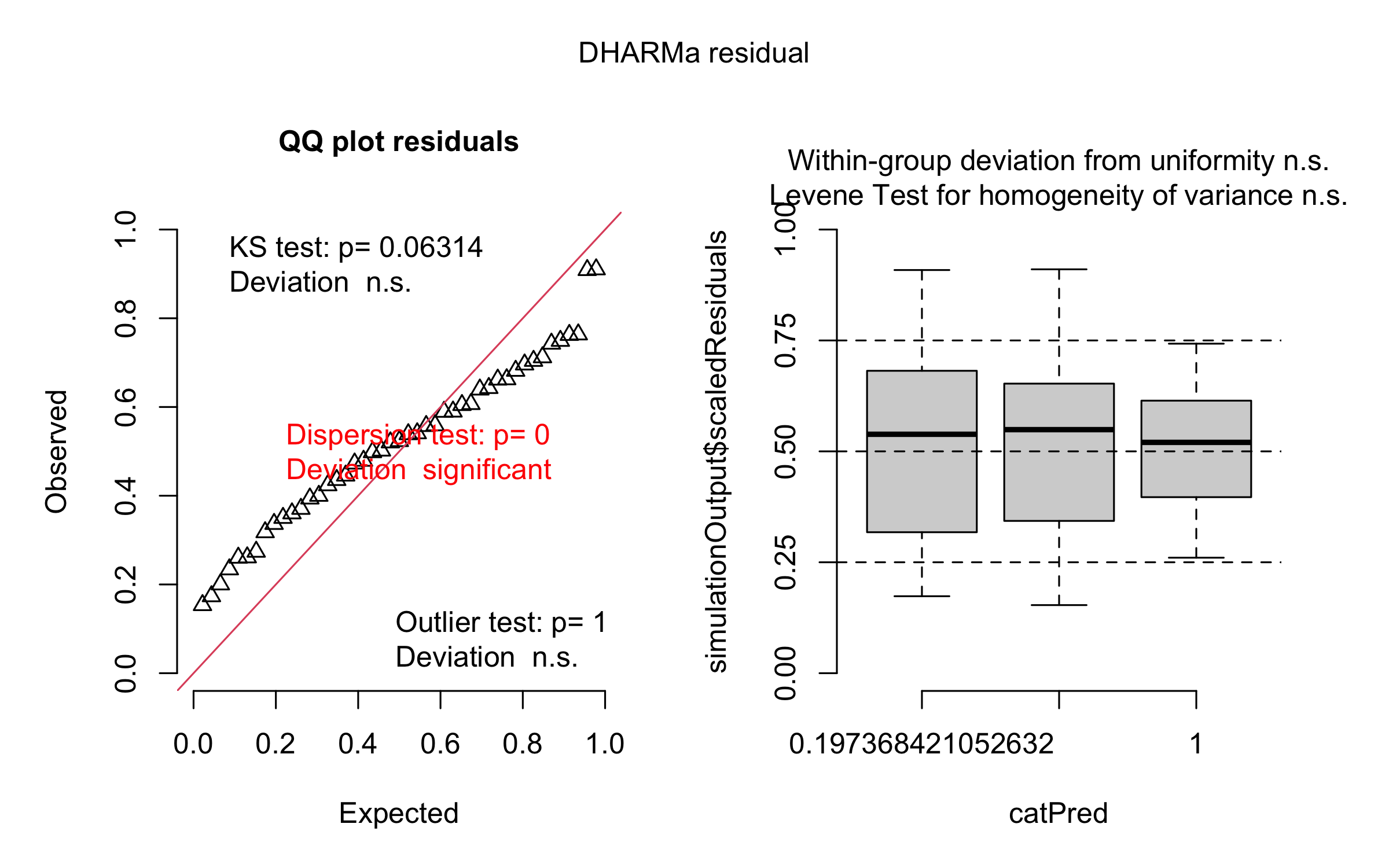

Supplement: Supplementary file 6 — Source Data [file 41467_2026_71014_MOESM6_ESM.zip › Source Data/Statistical Report/Diagnosis/FigureS6D_Number_of_Branches_DHARMa.png]

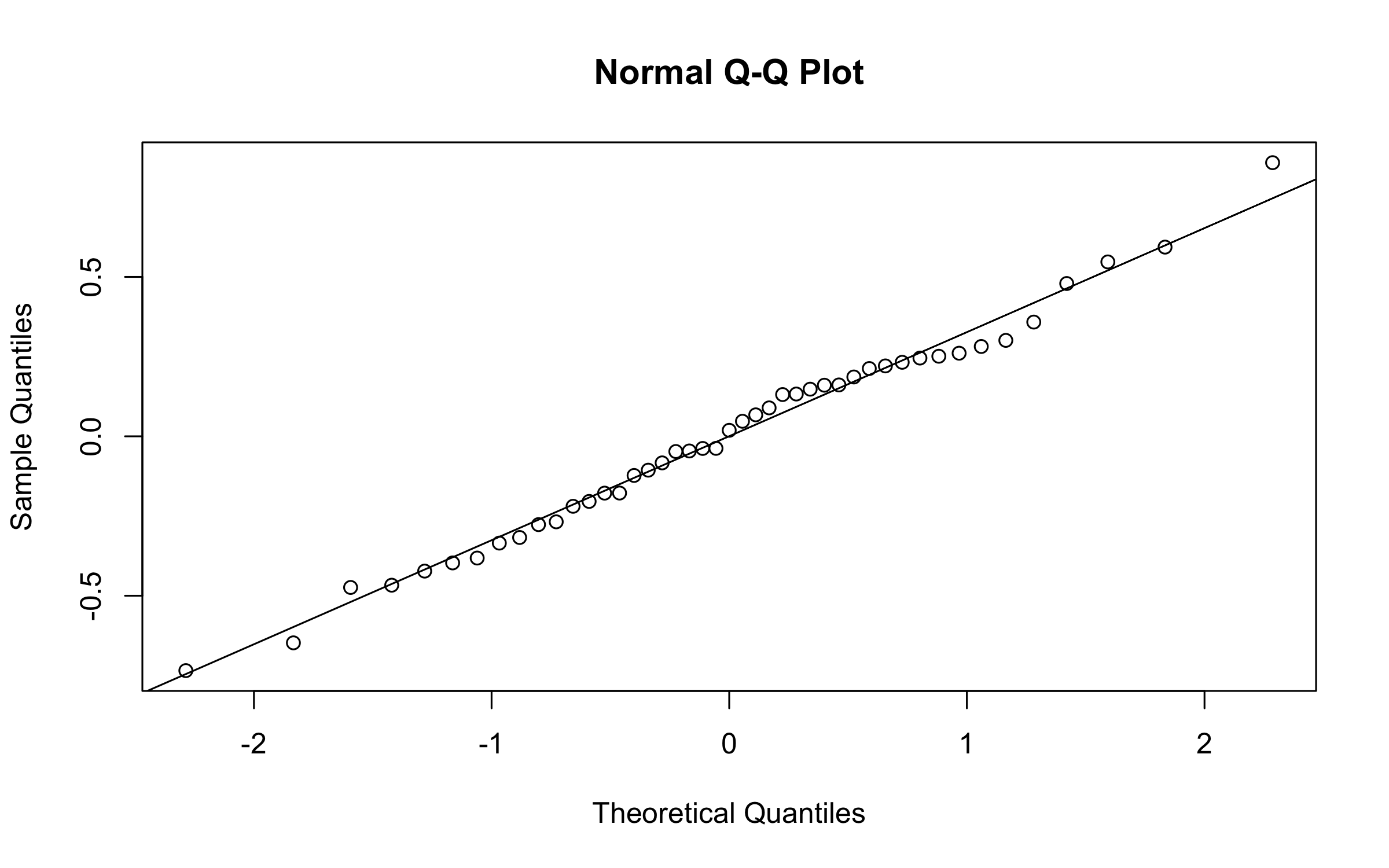

Supplement: Supplementary file 6 — Source Data [file 41467_2026_71014_MOESM6_ESM.zip › Source Data/Statistical Report/Diagnosis/FigureS6D_Total_Branches_Log_QQ.png]

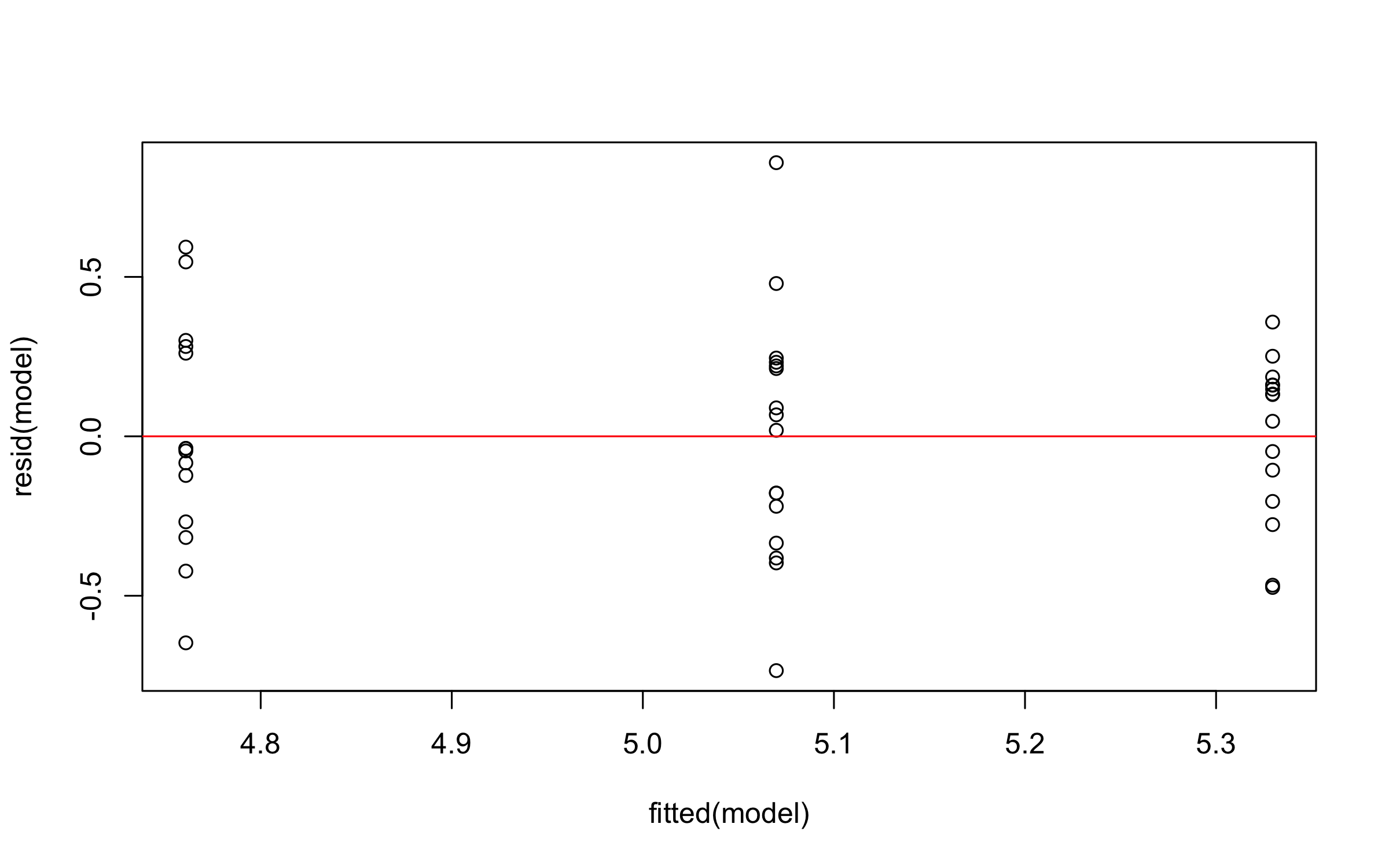

Supplement: Supplementary file 6 — Source Data [file 41467_2026_71014_MOESM6_ESM.zip › Source Data/Statistical Report/Diagnosis/FigureS6D_Total_Branches_Log_ResidualFit.png]

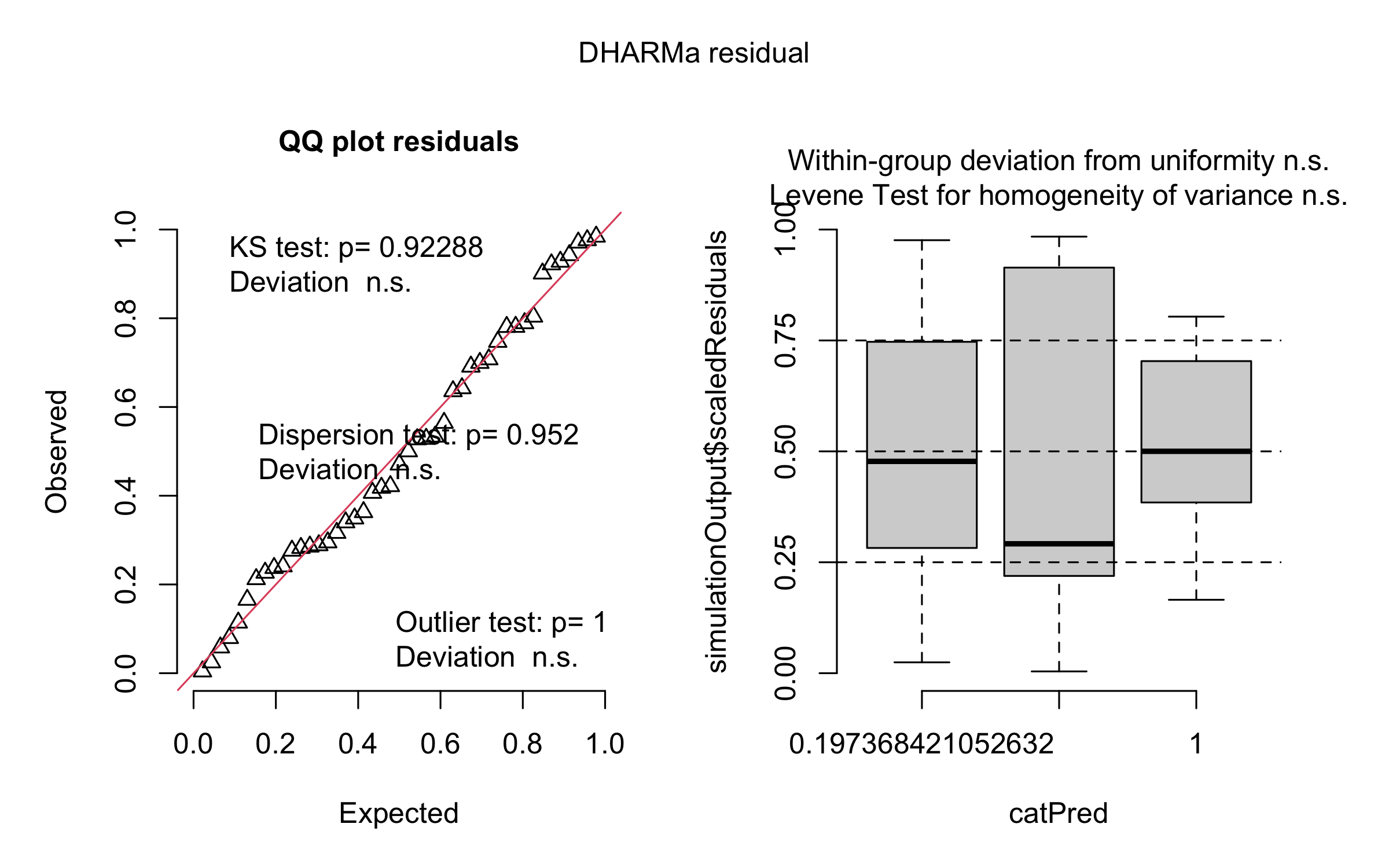

Supplement: Supplementary file 6 — Source Data [file 41467_2026_71014_MOESM6_ESM.zip › Source Data/Statistical Report/Diagnosis/FigureS6D_Total_Intersections_DHARMa.png]
