# Supplementary material for: Aurka-Bhlhe41 axis prevents premature aging-like microglial dysfunction and promotes remyelination
Source: Nat Commun. 2026 Mar 27;17:5238. doi: 10.1038/s41467-026-71014-w (PMC13260908; doi:10.1038/s41467-026-71014-w)
Supplement: Supplementary file 5 — Transparent Peer Review file [file 41467_2026_71014_MOESM5_ESM.pdf]

nature portfolio

Peer Review File

Aurka-Bhlhe41 axis prevents premature aging-like microglial dysfunction and promotes remyelination

Corresponding Author: Professor Hui Wang

This file contains all reviewer reports in order by version, followed by all author rebuttals in order by version.

Attachments originally included by the reviewers as part of their assessment can be found at the end of this file.

Version 0:

Reviewer comments:

Reviewer #1

(Remarks to the Author)
The authors investigated the role of the transcription factor Bhlhe41 and its coregulator Aurora kinase A (Aurka) in regulating microglial morphology, phagocytosis, and both de- and remyelination. They propose that Aurka mitigates the self-repressive activity of Bhlhe41 in microglia, thereby preventing the upregulation of CD22—a phagocytic receptor and marker of aged microglia. Loss of either Bhlhe41 or Aurka results in CD22 upregulation, leading to a premature aging phenotype characterized by dystrophic microglial morphology, phagocytic impairments, and defective remyelination.

The authors employ multiple mouse models, including both global and conditional knockouts of Bhlhe41 and Aurka, and provide mechanistic insights supported by a rescue experiment in which ectopic Bhlhe41 expression restores the phenotype in Aurka-deficient mice.

We appreciate the substantial effort that went into this study, and we recognize its potential relevance to the field of microglial biology and myelin repair. However, in its current form, the manuscript contains major weaknesses in experimental design, model validation, and statistical interpretation. Below, we outline the key concerns.

Summary of Major Issues

Lack of Wild-Type Controls

Virtually all experiments compare only B41HET and B41KO mice. Without inclusion of WT animals, the direction and magnitude of Bhlhe41's effects cannot be established. This omission is particularly problematic given the hypothesized autoregulatory loop of Bhlhe41. WT controls are essential for proper interpretation of gene dosage effects.

Incomplete Knockout and Model Validation

The Bhlhe41^dTomato-Cre line used for microglia-specific ablation retains substantial Bhlhe41 protein in "KO" cells (Fig. S1D), undermining the validity of the conclusions drawn from most of the experiments. The authors should instead use the floxed null allele (KO) and respective WT control mice that they generated in this study, after confirmation of microglia specificity and estimation of recombination efficiency.

Underuse of the Floxed Bhlhe41 Model

The Bhlhe41^fl/fl allele is a powerful tool that could enable definitive comparisons across WT, HET, and KO genotypes. Yet it is used only once, and without WT controls. This limits the ability to assess gene dosage effects and the plausibility of the proposed self-repression mechanism.

Lack of Statistical Details and Pseudoreplication

Statistical analyses appear to treat repeated measurements as independent replicates. For example, n=16 data points in Figures 2B and S3B/D arise from only three mice. This inflates statistical significance and violates assumptions of independence. Reanalysis using the correct unit of replication (e.g., individual animals) or appropriate hierarchical models is required. Another major issue is the lack of statistical details like test statistics with degrees of freedom, confidence intervals, and multiple testing correction used.

Additional Comments and Questions

Use of B41HET Mice as Controls

- What is the rationale for using B41HET as a default comparator in all experiments? If HET mice already exhibit haploinsufficient phenotypes, this invalidates conclusions drawn from HET vs KO comparisons.

- Supplementary Figure 1D suggests persistent Bhlhe41 expression in KO cells. Could this reflect Cre leakiness or incomplete recombination?

- Figure 1A suggests no increase in dTomato in B41HET relative to WT, arguing against self-repression at the heterozygous state. Is autoregulation only observed in complete KO?

- Is B41Het an appropriate tool to study microglia in vivo, as stated in line 187, given that these cells still retain 50% Bhlhe41?

- What does remyelination look like in WT mice? Are B41HET mice already impaired?

Validation of Mouse Models

- Have the authors validated microglia-specific recombination using lineage markers (e.g., P2RY12, CX3CR1) in their conditional KO (B41cKO, and Cx3cr1-Aurka fl/fl))?

- Has Bhlhe41 deletion been confirmed at the mRNA/protein level in sorted microglia?

- Similar validation is needed for Aurka deletion in B41HET;Aurka^fl/fl and Cx3cr1-Aurka^fl/fl backgrounds (microglial identity, Aurka loss via flow or western blot.

Clarification of MBP and Myelin Analyses (Figure 3D)

- Does “MBP reduction” refer to total intensity, area coverage, or puncta count? Consider plotting raw MBP intensities.

- Lines 257–259 mention increased myelin debris and unstructured sheaths; however, Figure 3D does not clearly differentiate between MBP puncta (fragmented myelin) vs intact sheaths.

- Have authors considered temporal delay in remyelination rather than failure? Are later time points (e.g., 2 weeks) available?

Phagocytosis Assays

- Lines 282–283 overstate the conclusions. Phagocytosis of myelin debris has not been directly measured. E. coli and zymosan beads do not capture endogenous phagocytosis of myelin debris.

- Co-staining for CD68 or LAMP1 with MBP could confirm in vivo internalization.

- Morphological assessments alone are insufficient to support claims of defective clearance (Line 349). Use of CD68/LAMP1 co-staining may be a better choice.

Minor Issues and Clarifications

- Supplementary Figure 1: Show gMFI and percent positivity for all populations (microglia, B-1 cells, alveolar macrophages). Quantify blots for all genotypes.

- Figure 1A: CD206 alone is insufficient to distinguish BAM subtypes; consider Lyve1 (PVM) and MHC-II (LMM). Iba1 is not specific for microglia—prefer P2RY12, Sall1, or Tmem119.

- Figures 2C, 2E: Include gMFI for phagocytosis markers to assess per-cell capacity.

- Line 306: Revise “microglial identity”—the authors are measuring function and morphology.

- Supplementary Figure Mismatch: Correct citation errors (e.g., Lines 311–321).
- Line 311: Should refer to Supplementary Figure 6, not 5.
- Line 317: Supplementary Figure 7A, not 6A.
- Line 319: Supplementary Figure 7A-B, not 6A-B.
- Line 321: Supplementary Figure 7C, not 6C.

- Line 894: Clarify method for microglial isolation (mechanical vs enzymatic) as this can influence microglial activation. Indicate whether whole brains were used or regions excluded.

- Supplementary Figure 12: CD68 signal is faint and hard to interpret.

(Remarks on code availability)

Reviewer #2

(Remarks to the Author)
I co-reviewed this manuscript with one of the reviewers who provided the listed reports. This is part of the Nature Communications initiative to facilitate training in peer review and to provide appropriate recognition for Early Career Researchers who co-review manuscripts.

(Remarks on code availability)

Reviewer #3

(Remarks to the Author)
Li and colleagues use several new and interesting mouse lines to investigate the transcription factor Bhlhe41. There are some novel and interesting findings in this manuscript regarding how Bhlhe41 impacts microglia morphology and phagocytosis. They find that Bhlhe41 restrains CD22, a known inhibitor of phagocytosis that increases with age. Overall, there is essential findings in this work. However, I do have some serious concerns about how conclusions are made without appropriate analyses or data to support these conclusions.

Major concerns
-the claim that B41KO recovery in the EAE is not due to hyperactive T-cells is not well backed up by the data. Figure 3B measures the proportion of CD4 T-cells that express IFN-g and IL17. These measurements are too superficial for this conclusion. They should measure the proportion of leukocytes/ CD4 and CD8 T-cells as a whole. Many other immune cells could also contribute to this recovery, so the link with microglia is extremely tenuous.
-For the cuprizone work in Figures 3 and 4, the MBP images are (for me) blurry and unclear). By convention, the dorsal side of the brain should be up, but I suspect these are flipped. It is also unclear where these images are taken in the corpus callosum. MBP will label both myelin and myelin debris, so the claims of more or less demyelination/ remyelination with MBP are problematic. These analyses should be confirmed with compact myelin stains that are more sensitive to intact myelin, such as Sudan black or black gold staining.
-While g-ratios are a good metric for EM analyses, they do not directly inform the degree of demyelination and remyelination in this model. These EM images should be quantified and the proportion of unmyelinated and myelinated axons should be reported in the corpus callosum. The authors should also make it clear how they know they were quantifying axons in the corpus callosum
The methods for the cuprizone section should outline how they analyzed the various data (olig2/ MBP, etc.). For example, how many images were taken, what region was quantified, how were these images blinded, etc.
-The authors talk about myelin debris but offer no data about how much debris is inside of microglia during demyelination and remyelination in this model. The authors need to examine debris uptake directly to conclude in line 263 “that Bhlhe41 is required to promote clearance of myelin debris”. The authors should also not use enlarged cell bodies of microglia as indications of “sustained phagocytic activity.” This should be measured more directly.
-as there may or may not (it is unclear from the data) be differences in baseline demyelination in this model, and differences in demyelination will impact remyelination, the authors are not in a position to make any conclusion on remyelination with the data they provide. They do not differentiate between unmyelinated and myelinated axons, spared myelin and myelin debris, or remyelination and myelin debris.
-The authors use Olig2 as a marker of oligodendrocyte. Olig2 is found in both OPCs and oligodendrocytes. They should redo these quantifications with markers of both OPCs and oligodendrocytes.
I do not see CD22 in all the immunofluorescence images. The authors need to re-examine the CD22 representative images. For the phagocytosis assays, it is important to understand how the anti-CD22 antibody impacts control animals, not just Aukura cKO and B41 KO mice.
-given that they treat cuprizone mice with anti-CD22 throughout the demyelination phase, it is not possible to differentiate

between the possibility that this works to limit demyelination or promote remyelination.

Smaller points

-In figure 1c, I do not limited tdTom in the pvM and mM as the images are too zoomed out and of a low quality
-in line 185, you call Bhlhe41 microglia specific, but it is in a population of BAM and likely other myeloid cells, so this language should be tempered to something like microglia enriched
-In Figures 2A and 2B, you refer to microglia in the Bhlhe41 deficient group as being dystrophic. These do not look dystrophic but more shrunken with shorter, fatter processes. I did not see blebbing or fragmented processes from the images, although my images were of a low quality.
-The rationale behind doing an EAE study was problematic. Line 229:"To examine whether Bhlhe41 deficiency affects CNS myelination, we established an inflammatory demyelinating model…". EAE is an inflammatory model, so it is unlikely to impact myelination, such as developmental myelination. Also, no myelin metric was investigated here. EAE is not a model (with these mice) to distinguish remyelination, so a new rationale should be included.
-Line 319 may refer to Supp Fig. 7, not Supp Fig. 6.

(Remarks on code availability)

Version 1:

Reviewer comments:

Reviewer #1

(Remarks to the Author)
See attached pdf document with my comments for the authors

(Remarks on code availability)

Reviewer #2

(Remarks to the Author)
I co-reviewed this manuscript with one of the reviewers who provided the listed reports. This is part of the Nature Communications initiative to facilitate training in peer review and to provide appropriate recognition for Early Career Researchers who co-review manuscripts.

(Remarks on code availability)

Reviewer #4

(Remarks to the Author)
This is a revised manuscript aimed at studying the function of Aurka/Bhlhe41 in the context of myelin repair. I want to commend the authors for their work on this revised manuscript; overall, I feel that it has been significantly strengthened. The study used an impressive combination of genetic mouse models and pharmaceutical intervention to show that Bhlhe41- or Aurka-deficient mice have microglia that exhibit aging characteristics, consisting of altered morphology, phagocytic deficits, progressive CD22 upregulation, leading to remyelination impairment in cuprizone-induced demyelination. I have a few minor suggestions for the authors.

The diversity of phagocytic cargo used is confusing. Why do the authors utilize E. coli and zymosan, which are not biologically relevant to the study of remyelination? A refocus on myelin debris and apoptotic cells could be more informative.

The authors should be careful about their conclusions regarding phagocytosis; only Phrodo-labeled molecules (Fig. 2d for example) will allow the team to discern between internalization versus binding.

The authors may want to include in the text that OLIG2 stains all cells of the oligodendrocyte lineage (OPCs and oligodendrocytes).

Fig 4a-b should be moved to a supplementary figure.

(Remarks on code availability)

Version 2:

Reviewer comments:

Reviewer #1

(Remarks to the Author)
I thank the authors for their careful and thorough responses to my previous comments. The revised manuscript has satisfactorily addressed all of my major concerns.

I have no further substantive comments and consider the manuscript suitable for publication.

(Remarks on code availability)
I did not independently run the code. However, the repository appears well organized and publicly accessible, with sufficient documentation to allow inspection and reuse by the community. The availability of the code supports transparency and reproducibility.

Reviewer #2

(Remarks to the Author)
I co-reviewed this manuscript with one of the reviewers who provided the listed reports. This is part of the Nature Communications initiative to facilitate training in peer review and to provide appropriate recognition for Early Career Researchers who co-review manuscripts.

(Remarks on code availability)

Open Access This Peer Review File is licensed under a Creative Commons Attribution 4.0 International License, which permits use, sharing, adaptation, distribution and reproduction in any medium or format, as long as you give appropriate credit to the original author(s) and the source, provide a link to the Creative Commons license, and indicate if changes were made.
In cases where reviewers are anonymous, credit should be given to 'Anonymous Referee' and the source.
The images or other third party material in this Peer Review File are included in the article's Creative Commons license, unless indicated otherwise in a credit line to the material. If material is not included in the article's Creative Commons license and your intended use is not permitted by statutory regulation or exceeds the permitted use, you will need to obtain permission directly from the copyright holder.
To view a copy of this license, visit https://creativecommons.org/licenses/by/4.0/

Response to Reviewers

Manuscript ID: NCOMMS-25-19678A-Z
Title: Aurka-Bhlhe41 axis prevents premature aging-like microglial dysfunction and promotes remyelination

We thank the reviewers for their time and insightful comments, which have significantly improved our manuscript. We have addressed all points raised in a point-by-point manner below. All changes in the manuscript have been tracked, and new data have been incorporated into the figures. In addition, we have provided a high-resolution PDF version of all images as a separate file to ensure optimal clarity for visualization and publication.

Reviewers' comments:

Reviewer #1 (Remarks to the Author):
The authors investigated the role of the transcription factor Bhlhe41 and its coregulator Aurora kinase A (Aurka) in regulating microglial morphology, phagocytosis, and both de- and remyelination. They propose that Aurka mitigates the self-repressive activity of Bhlhe41 in microglia, thereby preventing the upregulation of CD22—a phagocytic receptor and marker of aged microglia. Loss of either Bhlhe41 or Aurka results in CD22 upregulation, leading to a premature aging phenotype characterized by dystrophic microglial morphology, phagocytic impairments, and defective remyelination.

The authors employ multiple mouse models, including both global and conditional knockouts of Bhlhe41 and Aurka, and provide mechanistic insights supported by a rescue experiment in which ectopic Bhlhe41 expression restores the phenotype in Aurka-deficient mice.

We appreciate the substantial effort that went into this study, and we recognize its potential relevance to the field of microglial biology and myelin repair. However, in its current form, the manuscript contains major weaknesses in experimental design, model validation, and statistical interpretation. Below, we outline the key concerns.

Summary of Major Issues

Lack of Wild-Type Controls
Virtually all experiments compare only B41HET and B41KO mice. Without inclusion of WT animals, the direction and magnitude of Bhlhe41's effects cannot be established. This omission is particularly problematic given the hypothesized autoregulatory loop of Bhlhe41. WT controls are essential for proper interpretation of gene dosage effects.

- Response: We thank the reviewer for this critical and insightful comment. We fully

agree that the inclusion of WT controls is essential to establish a baseline and properly interpret gene dosage effects, especially for Bhlhe41 that is self-repressed in microglia.

In our initial investigation, we did include WT controls for the foundational characterization of microglia number and morphology under homeostatic conditions. Those data confirmed that *B41*^{HET} and *B41*^{cHET} microglia were indistinguishable from WT. This is supported by comparable CD22 expression between WT and *B41*^{HET} microglia (**Fig 5b**, **Fig 6c** and **Fig S7**). However, we agree that WT controls should be included for all key experiments as a true baseline. We apologize for this in our initial submission.

To address this fundamental concern, we have included the WT data from our initial analysis and re-performed key experiments using WT mice as controls. This includes:

1. Western blot (WB) analysis of Bhlhe41 expression, revealing its partial but not halved reduction in both *B41*^{HET} (**Fig.1b**) and *B41*^{cHET} (**Fig.2c**) microglia compared to WT, supporting its auto-repressive mechanism.

2. Immunofluorescence (IF) analysis of microglia number and morphology in both Bhlhe41 global (WT, *B41*^{HET} and *B41*^{KO} mice, **Fig.2a-b**, **S3a-b**) and conditional (WT, *B41*^{cHET} and *B41*^{cKO} mice, **Fig.S3c-d**) knockout models, demonstrating a dystrophic-like phenotype only in KO mice, with WT and HET mice being indistinguishable.

3. WB analysis of Aurka and Bhlhe41 expression in WT, *B41*^{HET}*Aurka*$^{fl/+}$, *B41*^{HET}*Aurka*$^{fl/fl}$ (**Fig.4b**) and *Cx3cr1*$^{Cre/+}$*Aurka*$^{+/+}$, *Cx3cr1*$^{Cre/+}$*Aurka*$^{fl/+}$ and *Cx3cr1*$^{Cre/+}$*Aurka*$^{fl/fl}$ *microglia* (**Fig. 4c**), *revealing s*uccessful knockout of *Aurka* in a gene-dosage-dependent manner. While Aurka expression was halved in Aurka cHET microglia and ablated in Aurka cKO microglia, Bhlhe41 expression was significantly reduced only in the Aurka cKO group but slightly reduced in Aurka cHET microglia.

4. IF analysis of microglia number and morphology in microglial-specific Aurka knockout mice models (*B41*^{HET}*Aurka*$^{+/+}$, *B41*^{HET}*Aurka*$^{fl/+}$ and *B41*^{HET}*Aurka*$^{fl/fl}$ mice, **Fig.S5a**; *Cx3cr1*$^{Cre/+}$*Aurka*$^{+/+}$, *Cx3cr1*$^{Cre/+}$*Aurka*$^{fl/+}$ and *Cx3cr1*$^{Cre/+}$*Aurka*$^{fl/fl}$. **Fig. S5b**), demonstrating impaired microglia number and morphology in Aurka cKO mice, with WT and cHET mice being indistinguishable.

5. Key functional assays, including *in vitro* and *in vivo* phagocytosis (**Fig.2d-i** and **Fig. 4f**) and the CPZ-induced demyelination model (**Fig.3** and **Fig.4g-j**), were re-performed using *B41*^{cKO} and *Aurka*^{cKO} mice, and *Cx3cr1*$^{Cre/+}$ mice as WT controls.

Incomplete Knockout and Model Validation
The Bhlhe41^dTomato-Cre line used for microglia-specific ablation retains substantial

Bhlhe41 protein in "KO" cells (Fig. S1D), undermining the validity of the conclusions drawn from most of the experiments. The authors should instead use the floxed null allele (KO) and respective WT control mice that they generated in this study, after confirmation of microglia specificity and estimation of recombination efficiency.

- Response**:** We thank the reviewer for raising this important point regarding the validation of our genetic model. We agree that the faint band observed in the WB (previously Fig. S1D, now **Fig.1b** in the revised manuscript) warrants careful interpretation.

We would like to clarify that our Bhlhe41$^{dTomato\text{-}Cre}$ knock-in allele (designated *B41*^{HET} in heterozygotes and *B41*^{KO} in homozygotes) was designed to disrupt native Bhlhe41 protein expression by replacing its initiation codon with a *dTomato-2A-Cre-polyA* cassette. It is well-established that Bhlhe41 is essential for B-1a cell self-renewal[1]. In our previous publication[2], we functionally validated this allele by demonstrating a severe impairment of B-1a cell populations in *B41*^{KO} mice, with no effect observed in WT or *B41*^{HET} mice (as shown in Fig. 1C-D of the referenced work; an observation supported by an impaired B-1 cell ratio in now **Fig.S1d** in the revised manuscript). This result is consistent with the established role of Bhlhe41 and confirms that our allele acts as a functional null.

To further address this concern directly, we performed additional WB analysis of *B41*^{KO} and *B41*^{cKO} microglia alongside WT and HET controls. Although a faint residual band was observed in both knockout models, quantitative densitometric analysis (ImageJ) revealed a significant reduction in Bhlhe41 protein levels in *B41*^{HO} and *B41*^{cKO} microglia compared to WT controls (new data presented in **Fig.1b** and **Fig.2c**).

We acknowledge that the faint residual signal could potentially stem from nonspecific binding of the anti-Bhlhe41 polyclonal antibody used. However, the profound reduction in Bhlhe41 protein levels, coupled with the severe functional deficits across all our assays, provides compelling evidence that our model effectively ablates Bhlhe41 function in microglia.

Underuse of the Floxed Bhlhe41 Model

The Bhlhe41^fl/fl allele is a powerful tool that could enable definitive comparisons across WT, HET, and KO genotypes. Yet it is used only once, and without WT controls. This limits the ability to assess gene dosage effects and the plausibility of the proposed self-repression mechanism.

- Response: We thank the reviewer for this insightful comment, which we fully agree with. We have now performed extensive new experiments using *B41*^{cKO} mice with WT controls to provide definitive comparisons.

Our initial molecular and phenotypic analyses (in both the original submission and the

revised data) consistently demonstrated that microglia from *B41*^{HET} and *B41*^{cHET} mice are functionally and morphologically indistinguishable from *WT* controls (**Fig.1b** and **Fig.2C** for Bhlhe41 expression; **Fig.2a-b** and **Fig.S3a-d** for morphology; **Fig. 5b** and **Fig.6c** for CD22 expression).

Therefore, to most efficiently address the reviewer's concern while maximizing the biological relevance of our comparisons, we focused our efforts on the most informative contrast: direct comparison between *B41*^{cKO} and WT controls in key functional and disease models.

Specifically, we have now performed new experiments for:

1. *In vitro* and *in vivo* phagocytosis assay**s** (revised **Fig.2d**, **2g** and **2I,** and **Fig.4f** for *Aurka*^{cKO}) clearly demonstrate phagocytic deficits in *B41*^{cKO} and *Aurka*^{cKO} group compared to *WT* controls
2. The CPZ-induced demyelination/remyelination model (revised **Fig. 3** and **Fig.4g-j** for *Aurka*^{cKO}), confirming that remyelination impairment is specifically caused by loss of *Bhlhe41* or *Aurka* in microglia.
3. CD22 blockade experiments, including i*n vivo* phagocytosis assays (**Fig.7b**) and the CPZ-induced demyelination model (**Fig.7d-i**) using *B41*^{cKO} mice.

Lack of Statistical Details and Pseudoreplication
Statistical analyses appear to treat repeated measurements as independent replicates. For example, n=16 data points in Figures 2B and S3B/D arise from only three mice. This inflates statistical significance and violates assumptions of independence. Reanalysis using the correct unit of replication (e.g., individual animals) or appropriate hierarchical models is required. Another major issue is the lack of statistical details like test statistics with degrees of freedom, confidence intervals, and multiple testing correction used.

- Response: We thank the reviewer for this important comment. We agree that treating individual cells as independent replicates inflates statistical significance. We have re-analyzed the Sholl data using a linear mixed-effects model, treating individual cells as repeated measurements nested within each mouse. This approach properly accounts for intra-mouse correlation, and the results remain consistent with our original conclusions. We have thoroughly revised the manuscript to include detailed descriptions of all statistical tests in both the figure legends and the Methods section, ensuring full transparency and reproducibility.

Additional Comments and Questions
Use of B41HET Mice as Controls
- What is the rationale for using B41HET as a default comparator in all experiments? If HET mice already exhibit haploinsufficient phenotypes, this invalidates conclusions drawn from HET vs KO comparisons.

- Response: We thank the reviewer for raising this critical point regarding the choice of controls, which we acknowledge was not sufficiently justified in our original submission.

The decision to use $B41^{HET}$ mice as controls in many experiments was based on our initial phenotypic characterization under homeostatic conditions. This analysis, which included WT, $B41^{\mathrm{HET}}$ and $B41^{\mathrm{KO}}$ mice, consistently demonstrated that microglia number and morphology in $B41^{HET}$ mice were phenotypically indistinguishable from WT controls. Furthermore, the use of littermate $B41^{\mathrm{HET}}$ and $B41^{\mathrm{KO}}$ mice was intended to minimize experimental variation in subsequent disease models.

However, we fully agree with the reviewer that establishing a true WT baseline is essential for all key experiments. Therefore, as detailed in our response to the previous comment on "## Lack of Wild-Type Controls", we have now systematically included WT control data into all critical experiments in the revised manuscript.

The new data now provides a clear and consistent narrative: while $B41^{\mathrm{HET}}$ microglia are functionally and morphologically normal, $B41^{\mathrm{KO}}$ microglia exhibit severe deficits across all assays. The inclusion of WT controls throughout the study firmly establishes this baseline and confirms that the conclusions drawn from the HET vs. KO comparisons are indeed valid.

We sincerely thank the reviewer for prompting us to provide this comprehensive analysis, which has significantly strengthened the rigor of our study.

- Supplementary Figure 1D suggests persistent Bhlhe41 expression in KO cells. Could this reflect Cre leakiness or incomplete recombination?

- Response: We thank the reviewer for this question, which is closely related to the previous point regarding model validation (##Incomplete Knockout and Model Validation). We believe there may be a misunderstanding regarding the fundamental design of our $\mathrm{Bhlhe41}^{\mathrm{dtomato\text{-}Cre}}$ genetic model, which is crucial for interpreting the data.

As detailed in our response to the previous comment (##Incomplete Knockout and Model Validation), this allele is not a conditional (floxed) allele, but rather a knock-in null allele. The genetic design involves replacing the endogenous *Bhlhe41* initiation codon with a *dTomato-2A-Cre-polyA* cassette. This manipulation abrogates the production of native Bhlhe41 protein, regardless of Cre activity. Therefore, the concepts of "Cre leakiness" or "incomplete recombination" do not apply to the core mechanism of *Bhlhe41* gene inactivation in this model.

However, the reviewer's question remains highly relevant to the interpretation of the WB data. Given that the allele is a definitive null, the most plausible explanations for the faint residual band in the $B41^{\mathrm{KO}}$ lanes (previous Fig. S1D, now Fig.1b) are non-

specific binding by the anti-Bhlhe41 polyclonal antibody to other proteins of similar molecular weight.

We have comprehensively addressed the concerns about protein expression and model validity in our previous response (##Incomplete Knockout and Model Validation), which we kindly refer the reviewer to.

- Figure 1A suggests no increase in dTomato in B41HET relative to WT, arguing against self-repression at the heterozygous state. Is autoregulation only observed in complete KO?

- Response: We thank the reviewer for this comment, which allows us to clarify a key aspect of our experimental design and the interpretation of the dTomato reporter.

There is no dTomato signal in WT microglia, as they lack the reporter allele entirely. In our *Bhlhe41*$^{dTomato\text{-}Cre}$ knock-in model, the dTomato reporter is expressed only in cells that have one or two copies of the knocked-in allele (*B41*^{HET} or *B41*^{KO}), replacing the native *Bhlhe41* gene. Therefore, the critical comparison for assessing Bhlhe41's autoregulation of its own promoter is not between *WT* (which lacks the dTomato reporter entirely) and *B41*^{HET} (which possesses one copy of the reporter), but rather between *B41*^{HET} and *B41*^{KO} microglia. Both *B41*^{HET} and *B41*^{KO} microglia possess the knocked-in dTomato reporter and therefore exhibit fluorescence driven by the *Bhlhe41* promoter. The key difference is that *B41*^{HET} microglia can produce functional Bhlhe41 protein to repress this promoter, whereas *B41*^{KO} cells cannot.

As we show in **Fig.1a**, deleting both alleles of *Bhlhe41* (*B41*^{KO}) causes a dramatic ~10-fold increase in dTomato signal compared to *B41*^{HET} microglia. This magnitude of change far exceeds the expected 2-fold increase from gene dosage alone and provides strong evidence for a positive feedback loop where Bhlhe41 protein represses its own promoter. Consistently, ectopic expression of Bhlhe41 from the Rosa26 promoter (*B41*^{HET}*R26*$^{mB41\text{-}EYFP/+}$) suppressed dTomato expression, further supporting the model of Bhlhe41-mediated self-repression (**Fig. 6c**).

Furthermore, this finding of Bhlhe41 self-repression is independently corroborated by WB analysis. We found that Bhlhe41 protein levels in *B41*^{HET} and *B41*^{cHET} microglia are not reduced by half, but instead show only a partial reduction compared to WT (**Fig.1b** and **Fig.2c**). This clear deviation from the expected gene dosage effect further supports the conclusion that Bhlhe41 limits its own expression.

- Is B41Het an appropriate tool to study microglia in vivo, as stated in line 187, given that these cells still retain 50% Bhlhe41?

- Response: We thank the reviewer for this important question. *B41*^{HET} microglia retain one functional *Bhlhe41* allele, and as we show in the revised manuscript possess

substantial Bhlhe41 protein activity (**Fig.1b** and **Fig.2c**). We agree that for studying the cell-autonomous functions of Bhlhe41, the homozygous knockout (*B41*^{KO}) would be the more appropriate model. However, our statement that "*B41*^{HET} mice may be a useful tool to study microglia *in vivo*" was made in a specific context: its utility as a highly specific genetic tool for targeting and tracing microglia.

The key evidence supporting this utility is:

1. Exceptional cellular specificity: In *B41*^{HET}*R26*^{EYFP} mice, we observed irreversible EYFP labeling in >99% of microglia, with minimal labeling of other CNS cell types (~10% in CNS-associated macrophages) (**Fig. 1e-f** and **Fig.S2A**).
2. Early and Sustained Expression: This microglia-specific labeling is established as early as E15.5 in the fetus (**Fig.1g** and **Fig.S2b**) and persists into adulthood, enabling fate-mapping and longitudinal studies.

In summary, we propose that the *B41*^{HET} mouse is not a tool for interrogating the loss of Bhlhe41 function, but rather a highly specific and sensitive genetic tool for targeting, tracking, and manipulating microglia *in vivo* across developmental and adult stages. We have revised the wording in the manuscript (now Line 206-207) from "a useful tool" to "a useful genetic tool for targeting and tracing microglia *in vivo*" to eliminate any potential ambiguity regarding its intended application.

- What does remyelination look like in WT mice? Are B41HET mice already impaired?

- Response: We thank the reviewer for this critical question regarding the baseline of remyelination and the potential phenotype of *B41*^{HET} mice.

In the CPZ-induced demyelination model, WT mice exhibit the expected sequence of events: robust demyelination after 5 weeks of CPZ diet, followed by efficient spontaneous remyelination upon return to a normal diet. To assess myelination in the corpus callosum, we analyzed WT and B41^{cKO} mice using complementary approaches: Black Gold II (BGII) staining to visualize intact myelin, immunofluorescence for myelin basic protein (Mbp) to detect total myelin, and transmission electron microscopy (TEM) to quantify myelinated and unmyelinated axons. Our revised data clearly recapitulate this process in WT mice, showing substantial myelin loss during demyelination and significant recovery after a 2-week recovery period (revised **Fig. 3b–d**).

Regarding the reviewer's specific question about *B41*^{HET} mice, our initial and revised analyses consistently demonstrate that *B41*^{HET} and *B41*^{cHET} microglia are molecularly and phenotypically indistinguishable from WT controls. This is evidenced by: 1) only a partial, non-50% reduction in Bhlhe41 protein levels (**Fig. 1b** and **Fig. 2c**), consistent with its self-repressive mechanism. 2) comparable microglial morphology (**Fig. 2a-b** and **Fig.S3**) and 3) no significant difference in the expression of CD22 (**Fig.5b** and

Fig.6c).

Therefore, we focused our analysis on the comparison between WT and *B41*^{cKO} (**Fig. 3**), which revealed a profound and specific requirement for Bhlhe41 in promoting microglial clearance of myelin debris and supporting remyelination.

Validation of Mouse Models
- Have the authors validated microglia-specific recombination using lineage markers (e.g., P2RY12, CX3CR1) in their conditional KO (B41cKO, and Cx3cr1-Aurka fl/fl))?

- Has Bhlhe41 deletion been confirmed at the mRNA/protein level in sorted microglia?
- Similar validation is needed for Aurka deletion in B41HET;Aurka^fl/fl and Cx3cr1-Aurka^fl/fl backgrounds (microglial identity, Aurka loss via flow or western blot.

- Responses: We thank the reviewer for raising this important point regarding the validation of cell type specificity in our conditional knockout models. We fully agree that demonstrating microglia-specific recombination and gene deletion at the tissue level would be ideal. Unfortunately, due to the lack of reliable immunofluorescence-grade antibodies for Bhlhe41 and Aurka, we were unable to directly assess protein loss in situ within brain sections. Regarding specificity, the Cx3cr1-Cre driver line is widely used and well-established for achieving efficient recombination in microglia within the CNS, although partial recombination in peripheral myeloid cells has been reported. Our phenotypic analyses were performed on homeostatic microglia and the CPZ-induced demyelination model without peripheral immune cells infiltration, minimizing confounding effects from peripheral subsets.

To validate our KO mouse models, we validated gene deletion in sorted microglia by WB, which confirmed efficient loss of Bhlhe41 in *B41*^{KO} (**Fig.1b)** and *B41*^{cKO} (**Fig.2c)** mice, and of Aurka in *B41*^{HET}*Aurka*$^{fl/fl}$ (**Fig.4b**), *Cx3cr1*$^{Cre/+}$*Aurka*$^{fl/fl}$ (**Fig.4c)** and *Cx3cr1*$^{CreER/+}$*Aurka*$^{fl/fl}$ (**Fig.S9a**) mice, compared with WT and HET mice. These results verify effective deletion at the protein level in microglia. We have clarified these points in the revised manuscript and included our microglia validation data to strengthen confidence in the knockout specificity.

Clarification of MBP and Myelin Analyses (Figure 3D)
- Does "MBP reduction" refer to total intensity, area coverage, or puncta count?
- Lines 257–259 mention increased myelin debris and unstructured sheaths; however, Figure 3D does not clearly differentiate between MBP puncta (fragmented myelin) vs intact sheaths.

- Responses: We thank the reviewer for this important comment. In the original version of the manuscript, "MBP reduction" referred to the quantification of total Mbp fluorescence intensity, which we acknowledge does not fully distinguish between intact myelin sheaths and fragmented myelin debris.

To address this limitation, in the revised manuscript we have repeated the analysis using WT and *B41*^{cKO} mice. In addition, we introduced staining for degraded Mbp (dMbp) to specifically identify myelin debris, thereby complementing the Mbp analysis. Quantification was still performed on total intensity, but the inclusion of dMbp now allows us to better differentiate between intact myelin and myelin debris.

Additionally, we performed Black-Gold II staining, which selectively labels compact myelin sheaths. This provided an independent validation of myelin loss and confirmed the conclusions drawn from our Mbp/dMbp analyses.

These new data are presented in **Fig 3b-c,g** and the corresponding text has been updated accordingly.

- Have authors considered temporal delay in remyelination rather than failure? Are later time points (e.g., 2 weeks) available?

- Responses: We thank the reviewer for this thoughtful comment. We agree that a temporal delay in remyelination is a possible explanation for the observed phenotype. In the current study, our analyses were limited to 2-week recovery after cuprizone withdrawal. At this stage, myelin restoration was markedly reduced compared with WT controls, but we observed ongoing myelin repair in the *B41*^{cKO} and *Aurka*^{cKO} mice, evidenced by increased colocalization of dMbp with Iba-1 (**Fig 3g** and **Fig 4j**). Thus, the phenotype may reflect a delayed rather than a completely failed remyelination process. We have clarified this interpretation in the revised Results sections.

Phagocytosis Assays
- Lines 282–283 overstate the conclusions. Phagocytosis of myelin debris has not been directly measured. E. coli and zymosan beads do not capture endogenous phagocytosis of myelin debris.
- Co-staining for CD68 or LAMP1 with MBP could confirm in vivo internalization.
- Morphological assessments alone are insufficient to support claims of defective clearance (Line 349). Use of CD68/LAMP1 co-staining may be a better choice.
- Responses: We thank the reviewer for pointing out this overstatement. We agree that our original assays using *E. coli* and zymosan particles did not directly capture endogenous phagocytosis of myelin debris. To address this concern, in the revised manuscript we performed co-staining of Iba-1 with dMbp, which specifically labels myelin debris. In WT mice subjected to cuprizone, we observed robust colocalization of Iba-1^{+} microglia with dMbp, whereas this colocalization was markedly reduced in *B41*^{cKO} mice, indicating impaired uptake of myelin debris.

Although we were unable to perform additional co-staining of CD68 with Mbp due to both antibodies being rat-derived, the Iba-1/dMbp analysis nonetheless provides direct *in vivo* evidence of myelin debris internalization by microglia. These new data are

included in **Fig.3g**, which reinforce our interpretation of defective myelin debris clearance in *B41*^{cKO} mice.

Minor Issues and Clarifications

- Supplementary Figure 1: Show gMFI and percent positivity for all populations (microglia, B-1 cells, alveolar macrophages). Quantify blots for all genotypes.

- Responses: We thank the reviewer for this suggestion. Our goal in **Fig.1** and **Fig. S1** was to quantitatively assess Bhlhe41 expression levels, particularly in populations such as microglia that exhibit continuous rather than binary expression. For this purpose, geometric mean fluorescence intensity (gMFI) provides a more informative measure of expression intensity than percent positivity. Regarding the WB data, all blots included in the manuscript were quantified across the relevant genotypes, and these quantifications are presented in the corresponding figures (**Fig.1b**, **Fig.2c**, **Fig.4b-c**, **Fig.9a** and **Fig.10b**).

- Figure 1A: CD206 alone is insufficient to distinguish BAM subtypes; consider Lyve1 (PVM) and MHC-II (LMM). Iba1 is not specific for microglia—prefer P2RY12, Sall1, or Tmem119.

- Responses: We thank the reviewer for this comment and agree that CD206 alone is insufficient to distinguish BAM subtypes. In **Fig.1d** and **1f**, CD206 was combined with anatomical localization to distinguish perivascular from leptomeningeal macrophages, which is commonly used [3]. We also agree that Iba-1 is not strictly microglia-specific[4]. However, parenchymal Iba-1^{+} cells predominantly correspond to microglia. Importantly, our flow cytometric analysis of homeostatic microglia using CD45^{lo}CD11b^{+}Cx3cr1^{+} gating confirmed microglial identity and allowed subsequent quantification analysis of Bhlhe41/Aurka expression and recombination efficiency, supporting the conclusions drawn from **Fig.1d** and **1f**.

- Figures 2C, 2E: Include gMFI for phagocytosis markers to assess per-cell capacity.

- Responses: We thank the reviewer for this insightful suggestion. For original Fig. 2C (now **Fig. 2d**, *in vitro* phagocytosis of *E coli*-pHrodo Green), we have now included gMFI analysis to assess per-cell phagocytic capacity, as the fluorescence intensity of pHrodo Green reflects both particle uptake and phagosome acidification.

For original Figure 2E (now **Fig. 2f-g**, *in vivo* phagocytosis of Zymosan- AF488), the AF488 signal does not change with phagosome acidification and only reports particle uptake. Therefore, gMFI is not an accurate measure of per-cell phagocytic activity in this context. Instead, we quantified the percentage of phagocytic microglia.

- Line 306: Revise “microglial identity”—the authors are measuring function and morphology.

- Responses: We thank the reviewer for pointing this out. We agree that “microglial identity” was not precise, as the experiments measured microglial number, morphology and phagocytic activity rather than identity per se. Accordingly, we have revised the sentence to:
“This finding led us to examine the role of Aurka in regulating microglial number, morphology and phagocytic function.”, line 358-359.

- Supplementary Figure Mismatch: Correct citation errors (e.g., Lines 311–321).
- Line 311: Should refer to Supplementary Figure 6, not 5.
- Line 317: Supplementary Figure 7A, not 6A.
- Line 319: Supplementary Figure 7A-B, not 6A-B.
- Line 321: Supplementary Figure 7C, not 6C.

- Responses: We thank the reviewer for pointing out the citation errors. These have been corrected in the revised manuscript. We have double-checked all figure citations throughout the manuscript to ensure their accuracy.

- Line 894: Clarify method for microglial isolation (mechanical vs enzymatic) as this can influence microglial activation. Indicate whether whole brains were used or regions excluded.

- Responses: We used mechanical dissociation to isolate microglia from whole brains. We have now clarified this in the Methods section, line 1150-1151.

- Supplementary Figure 12: CD68 signal is faint and hard to interpret.

- Responses: We acknowledge that the CD68 signal appeared relatively faint in the initial submission. In the revised manuscript, we have adjusted brightness and contrast to improve visibility and provided high-resolution PDF figures.

Reviewer #2 (Remarks to the Author):
I co-reviewed this manuscript with one of the reviewers who provided the listed reports. This is part of the Nature Communications initiative to facilitate training in peer review and to provide appropriate recognition for Early Career Researchers who co-review manuscripts.

Reviewer #3 (Remarks to the Author):

Li and colleagues use several new and interesting mouse lines to investigate the transcription factor Bhlhe41. There are some novel and interesting findings in this manuscript regarding how Bhlhe41 impacts microglia morphology and phagocytosis. They find that Bhlhe41 restrains CD22, a known inhibitor of phagocytosis that increases with age. Overall, there is essential findings in this work. However, I do have some serious concerns about how conclusions are made without appropriate analyses or data to support these conclusions.

Major concerns

-the claim that B41KO recovery in the EAE is not due to hyperactive T-cells is not well backed up by the data. Figure 3B measures the proportion of CD4 T-cells that express IFN-g and IL17. These measurements are too superficial for this conclusion. They should measure the proportion of leukocytes/ CD4 and CD8 T-cells as a whole. Many other immune cells could also contribute to this recovery, so the link with microglia is extremely tenuous.

-The rationale behind doing an EAE study was problematic. Line 229:"To examine whether Bhlhe41 deficiency affects CNS myelination, we established an inflammatory demyelinating model…". EAE is an inflammatory model, so it is unlikely to impact myelination, such as developmental myelination. Also, no myelin metric was investigated here. EAE is not a model (with these mice) to distinguish remyelination, so a new rationale should be included.

- Response: We address the above two related comments together. We thank the reviewer for raising concerns regarding our EAE experiments. We agree that the rationale for using EAE to assess the role of Bhlhe41 in CNS myelination was problematic. As the reviewer correctly pointed out, EAE is primarily an inflammatory demyelination model and does not reliably reflect developmental myelination or remyelination. Moreover, our original data on the proportion of IFN-γ^+ and IL-17$^+$ CD4$^+$ T cells were insufficient to conclude that recovery in *B41*^{KO} mice is independent of hyperactive T cells. In fact, we did examine overall leukocyte, CD4$^+$ T, CD8$^+$ T, neutrophil, macrophage and microglia proportions and numbers, and found no significant differences. We agree that EAE is not an ideal model to link microglial function with remyelination.

To better address the core focus of our study—microglial regulation of remyelination—we have removed the EAE data from the manuscript. Instead, we re-performed the CPZ-induced demyelination model using WT controls and *B41*^{cKO} mice. Moreover, we performed Black Gold II staining and co-staining of dMbp with Iba-1 to assess myelination and microglial phagocytic clearance of myelin debris (**Fig.3**). These new analyses provide direct and robust evidence supporting our finding that Bhlhe41 regulates microglial responses during remyelination.

-For the cuprizone work in Figures 3 and 4, the MBP images are (for me) blurry and unclear). By convention, the dorsal side of the brain should be up, but I suspect these

are flipped. It is also unclear where these images are taken in the corpus callosum. MBP will label both myelin and myelin debris, so the claims of more or less demyelination/ remyelination with MBP are problematic. These analyses should be confirmed with compact myelin stains that are more sensitive to intact myelin, such as Sudan black or black gold staining.

- Response: We thank the reviewer for the constructive suggestions regarding our CPZ experiments. To address these concerns and taking into account the reviewer's comment on appropriate controls, we re-performed the CPZ-induced demyelination study using WT mice as controls alongside *B41*^{cKO} and *Aurka*^{cKO} mice. As suggested, we employed Black Gold II staining to more accurately visualize compact myelin. In addition, we performed co-staining of dMbp with Iba-1 to examine microglial internalization with myelin debris. These new analyses provide clearer and more reliable evidence for the impact of *Bhlhe41* and *Aurka* deficiency in microglia on demyelination and remyelination.

-While g-ratios are a good metric for EM analyses, they do not directly inform the degree of demyelination and remyelination in this model. These EM images should be quantified and the proportion of unmyelinated and myelinated axons should be reported in the corpus callosum. The authors should also make it clear how they know they were quantifying axons in the corpus callosum

- Response: We thank the reviewer for this excellent suggestion regarding the EM analysis. We agree that quantifying the proportion of myelinated axons provides a more direct measure of remyelination efficiency. In the updated **Fig.3d** and **Fig.4h**, we analyzed the EM images by quantifying the proportion of myelinated vs. unmyelinated axons in the corpus callosum. This new analysis confirms that *B41*^{cKO} and *Aurka*^{cKO} mice exhibit a significantly higher percentage of unmyelinated axons compared to WT controls during the recovery phase.

Furthermore, as the reviewer rightly points out, we have now made it explicit in the Methods section how the corpus callosum was identified for EM sampling.

"The corpus callosum (CC), identified as the white matter tract between the lateral ventricles in coronal brain sections," line 997-999.

The methods for the cuprizone section should outline how they analyzed the various data (olig2/ MBP, etc.). For example, how many images were taken, what region was quantified, how were these images blinded, etc.

- Response: We thank the reviewer for this important comment. We agree that a detailed description of the quantitative image analysis is essential for rigor and reproducibility. We have now thoroughly revised the Methods section for the cuprizone model analyses. Please see line 1006-1010; line 1142-1145 in the revised manuscript.

-The authors talk about myelin debris but offer no data about how much debris is inside of microglia during demyelination and remyelination in this model. The authors need to examine debris uptake directly to conclude in line 263 “that Bhlhe41 is required to promote clearance of myelin debris”. The authors should also not use enlarged cell bodies of microglia as indications of “sustained phagocytic activity.” This should be measured more directly.

- Response: We thank the reviewer for this insightful comment, which is in line with a similar concern raised by Reviewer #1. We fully agree that direct evidence of myelin debris uptake is required to substantiate our conclusion.

To address this point, we performed new experiments in the revised manuscript to quantify myelin debris internalization by microglia *in vivo*. Specifically, we conducted co-staining of the microglial marker Iba-1 with dMbp in brain sections from cuprizone-treated mice.

This new analysis, now presented in **Fig.3g**, **Fig.4j** and **Fig.7i,** demonstrates that:
WT Iba1^{+} microglia exhibit robust colocalization with dMbp^{+} myelin debris during the demyelination phase. In contrast, this colocalization is markedly reduced in *B41*^{cKO}/*Aurka*^{cKO} mice during demyelination, indicating impaired uptake of myelin debris (**Fig.3g**, **Fig.4j**). In anti-CD22-treated *B41*^{cKO} mice, this colocalization is increased, indicating improved uptake of myelin debris (**Fig.7i**).

Regarding the point on microglial morphology, we thank the reviewer for the helpful feedback. We have removed the text in the revised manuscript and rely on the Iba-1/dMbp colocalization data as the primary evidence for the uptake of myelin debris.

-as there may or may not (it is unclear from the data) be differences in baseline demyelination in this model, and differences in demyelination will impact remyelination, the authors are not in a position to make any conclusion on remyelination with the data they provide. They do not differentiate between unmyelinated and myelinated axons, spared myelin and myelin debris, or remyelination and myelin debris.

- Response: We thank the reviewer for this important comment. We agree that potential differences in baseline demyelination must be carefully evaluated, as they can strongly influence the interpretation of remyelination.

To address this, in the revised manuscript we performed new experiments using *B41*^{cKO}, *Aurka*^{cKO} and WT mice subjected to the cuprizone-induced demyelination model. To examine myelinated axon, unmyelinated axon, intact myelin and myelin debris, Black-Gold II staining, Mbp/dMbp/Iba-1 staining and TEM were employed. Under homeostatic conditions, Black-Gold II staining, Mbp/dMbp staining and TEM analyses revealed no differences in myelination between cKO and WT mice. After 5

weeks of cuprizone treatment, Black-Gold II and TEM analyses showed comparable levels of demyelination between cKO and WT groups, despite slight increased Mbp staining observed. However, dMbp staining, which selectively labels myelin debris, revealed increased debris accumulation in cKO mice compared to WT mice. This suggests that while the extent of demyelination is similar, impaired debris clearance may already be evident during the demyelination phase. We fully acknowledge that microglial regulation of remyelination is a dynamic process that begins with efficient debris removal and proceeds to subsequent remyelination.

Our revised text now carefully reflects this distinction by interpreting our findings primarily in terms of defective debris clearance, while noting that this impairment is likely to impact the efficiency of the remyelination phase.

-The authors use Olig2 as a marker of oligodendrocyte. Olig2 is found in both OPCs and oligodendrocytes. They should redo these quantifications with markers of both OPCs and oligodendrocytes.

- Response: We thank the reviewer for this helpful comment. We agree that Olig2 alone does not distinguish between oligodendrocyte precursor cells (OPCs) and mature oligodendrocytes. In the revised manuscript, we therefore performed additional staining and quantification using CC-1 in addition to Olig2, which allows specific identification of mature oligodendrocytes. These new data are now included in **Fig. 3f, 4i** and **7h,** which confirm our original conclusion.

I do not see CD22 in all the immunofluorescence images. The authors need to re-examine the CD22 representative images.

- Response: We thank the reviewer for this careful observation. We apologize for the difficulty in visualizing CD22 in the immunofluorescence images. CD22 was detected in the green channel, which may have been less apparent in the original figure. Colocalization of CD22 (Green) and Iba-1(red) can be observed as yellow spots in the merged image (**Fig.5-6**). To address this, we have reprocessed the images by adjusting the contrast and brightness of the CD22 (green) channel, and white arrows were used to indicate areas of colocalization appearing as yellow. A high-resolution PDF version of the images has also been included to ensure that CD22 staining can be clearly visualized.

For the phagocytosis assays, it is important to understand how the anti-CD22 antibody impacts control animals, not just Aukura cKO and B41 KO mice.

- Response: We thank the reviewer for this important comment. Indeed, it is critical to understand the effect of anti-CD22 treatment in control animals. As demonstrated in the Nature 2019 study [5](Fig.3g of referenced paper), anti-CD22 treatment did not exert significant effects on young control mice, consistent with the very low baseline

expression of CD22 in young microglia. Therefore, our interpretation focuses on the differential impact observed in the *B41*^{cKO} and *Aurka*^{cKO} models, which show upregulated CD22 expression on young microglia.

-given that they treat cuprizone mice with anti-CD22 throughout the demyelination phase, it is not possible to differentiate between the possibility that this works to limit demyelination or promote remyelination.

- Response: We thank the reviewer for raising this important point. To address this, we performed additional experiments including both the cuprizone demyelination phase and the recovery phase. In the cuprizone-treated groups, despite a slight increase of Mbp staining, Black-Gold II staining revealed no overt differences between control and anti-CD22–treated mice, suggesting that global demyelination was comparable (**Fig. e-f**). However, staining for dMbp showed a reduction in the anti-CD22–treated group, indicating that CD22 blockade may already facilitate microglial clearance of myelin debris during the demyelination phase.

We agree with the reviewer that this raises the possibility that anti-CD22 acts not only during remyelination but also earlier during demyelination by enhancing debris removal. Given that microglial regulation of remyelination is a dynamic process that begins with efficient clearance of myelin debris, our data suggest that anti-CD22 treatment may contribute to both phases. We have revised the text to carefully reflect this nuance without overstating the conclusions.

Smaller points

-In figure 1c, I do not limited tdTom in the pvM and mM as the images are too zoomed out and of a low quality

- Response: We thank the reviewer for the comment and apologize for the low quality of the original JPG images. As the average proportion of tdTomato^{+} cells is only ~10%, we have now provided enlarged views highlighting the positive cells, together with high-resolution PDF versions of the figures to allow clearer visualization.

-in line 185, you call Bhlhe41 microglia specific, but it is in a population of BAM and likely other myeloid cells, so this language should be tempered to something like microglia enriched

- Response: We thank the reviewer for this insightful comment. We agree that "microglia-specific" was an overstatement and have revised the text throughout the manuscript to describe Bhlhe41 as "microglia-enriched". This change ensures our terminology precisely aligns with the expression data.

-In Figures 2A and 2B, you refer to microglia in the Bhlhe41 deficient group as being

dystrophic. These do not look dystrophic but more shrunken with shorter, fatter processes. I did not see blebbing or fragmented processes from the images, although my images were of a low quality.

- Response: We thank the reviewer for this careful observation and apologize for the low clarity of the original images. To better illustrate microglial morphology, we have now provided single-cell 3D reconstructions of young microglia in **Fig.2b** and middle-aged microglia in **Fig.S3**, from WT, HET, *B41*^{KO}, and *B41*^{cKO} mice. Dystrophic microglia, as described in the literature[6], are characterized by beaded and discontinuous processes. Our analyses show that *Bhlhe41*-deficient microglia exhibit similar features, including shorter and discontinuous processes, and fewer branch intersections. To ensure rigor and avoid overinterpretation, we have revised the text throughout the manuscript to describe these cells as displaying dystrophic-like morphology.

-Line 319 may refer to Supp Fig. 7, not Supp Fig. 6.

- Response: We thank the reviewer for pointing this out. Following the removal of one supplementary figure during revision, the numbering of subsequent figures has shifted. The correct citation has now been updated to Supplementary Fig.6. We have double-checked all figure citations throughout the manuscript to ensure their accuracy.

References

1. Kreslavsky, T., Vilagos, B., Tagoh, H., Poliakova, D.K., Schwickert, T.A., Wohner, M., Jaritz, M., Weiss, S., Taneja, R., Rossner, M.J., and Busslinger, M. (2017). Essential role for the transcription factor Bhlhe41 in regulating the development, self-renewal and BCR repertoire of B-1a cells. Nat. Immunol. *18*, 442-455. 10.1038/ni.3694.
2. Li, H., Tang, Y., Ren, J., Bai, R., Hu, L., Jia, W., Cao, Y., Hong, L., Xu, M., Gao, S., et al. (2022). Identification of novel B-1 transitional progenitors by B-1 lymphocyte fate-mapping transgenic mouse model Bhlhe41dTomato-Cre. Front. Immunol. *13*. 10.3389/fimmu.2022.946202.
3. Brioschi, S., Belk, J.A., Peng, V., Molgora, M., Rodrigues, P.F., Nguyen, K.M., Wang, S., Du, S., Wang, W.L., Grajales-Reyes, G.E., et al. (2023). A Cre-deleter specific for embryo-derived brain macrophages reveals distinct features of microglia and border macrophages. Immunity *56*, 1027-1045 e1028. 10.1016/j.immuni.2023.01.028.
4. Eme-Scolan, E., and Dando, S.J. (2020). Tools and Approaches for Studying Microglia In vivo. Frontiers in immunology *11*, 583647. 10.3389/fimmu.2020.583647.
5. Pluvinage, J.V., Haney, M.S., Smith, B.A.H., Sun, J., Iram, T., Bonanno, L., Li, L., Lee, D.P., Morgens, D.W., Yang, A.C., et al. (2019). CD22 blockade restores homeostatic microglial phagocytosis in ageing brains. Nature *568*, 187-192. 10.1038/s41586-019-1088-4.

6. Shahidehpour, R.K., Higdon, R.E., Crawford, N.G., Neltner, J.H., Ighodaro, E.T., Patel, E., Price, D., Nelson, P.T., and Bachstetter, A.D. (2021). Dystrophic microglia are associated with neurodegenerative disease and not healthy aging in the human brain. Neurobiol Aging *99*, 19-27. 10.1016/j.neurobiolaging.2020.12.003.

Response to Reviewers

Manuscript ID: NCOMMS-25-19678A-Z

Title: Aurka-Bhlhe41 axis prevents premature aging-like microglial dysfunction and promotes remyelination

We thank the reviewers for their time and insightful comments, which have significantly improved our manuscript. We have addressed all points raised in a point-by-point manner below. All changes in the manuscript have been tracked, and new data have been incorporated into the figures.

Reviewers' comments:

Reviewer #1:
The authors did a good job revising the manuscript and addressed most of my concerns regarding WT controls, statistical analysis, and demyelination assays. There are a few minor issues that should be addressed prior to publication:

1. Flow cytometry histograms: The Y-axes on the gMFI histograms are labeled inconsistently as "Normalized to Mode" or "Normalized to Model." This typo should be corrected, and the meaning of "Mode" or "Model" should be explained in the figure legend.
- Response: We thank the reviewer for pointing out the typo. We have corrected "Normalized to Model" to "Normalized to Mode" throughout the figures. In the revised manuscript, the figure legend now contains a brief definition, and a detailed description of this normalization method has been added to the revised Methods section.

In each figure legend with flow histograms:
"Fluorescence intensity histograms are normalized to their respective modes."

In the method sections:
"For visualization in histogram overlays, fluorescence intensity data were normalized to the mode of each distribution to standardize peak heights, thereby enabling direct visual comparison of expression distribution shapes across samples independent of cell count."

2. Figures 1a and 4a: Two-way ANOVA was used to analyze the data. Were the three groups compared at each time point, or were AUC values compared between genotypes?
- Response: We thank the reviewer for this comment and apologize for the confusion caused by the significance markings in the original figures. For both Fig. 1a and Fig. 4a (now Fig. S4a), we performed two-way ANOVA (Genotype × Time) followed by Bonferroni-adjusted pairwise tests to assess differences in Bhlhe41 expression at each developmental time point. To clarify, no AUC or cumulative measures were calculated.

We have revised the figures and updated the figure legends to indicate the P values for all time points.

3. Figure 3: Two-way ANOVA was employed, most likely to test interactions between phase and genotype, but the interaction results were not reported.

- Response: We thank the reviewer for this important comment and agree that the interaction result should be reported. We have added interaction P value to Fig 3, Fig 4 and Fig 7. All raw data and complete statistical details are provided in Source Data.

4. Supplementary Figure 1c: This figure does not seem to show an age-dependent increase in Bhlhe41 expression as stated in line 180. Instead it shows a gating strategy to sort or analyze microglia.

- Response: We thank the reviewer for pointing out the potential for confusion. We have revised line 180 to clarify that Supplementary Fig. 1c shows the gating strategy for microglia, while Fig. 1a contains the age-dependent expression data. The revised text now reads: "However, dTomato in microglia (Supplementary Fig.1c) from Bhlhe41$^{dTomato\text{-}Cre/+}$ mice (hereafter referred to as B41^{HET} mice) increased in an age-dependent manner (Fig.1a)."

5. Sholl measurements: It is appreciated that the authors used mixed models, which allow them to fully leverage their dataset. However, the statistical details provided are too minimal. The authors report using the lme4 package, but the package version (v.X.X.X) is missing. What were considered fixed and random effects? Ideally, the authors should share the model formula and raw data for reproducibility.

Response: We thank the reviewer for this comment. The Sholl statistical analyses were performed in R (v4.2.2) using the lme4 (v1.1-33), lmerTest (v3.1-3), emmeans (v1.11.1), DHARMa (v0.4.7), MASS (v7.3-60) and nlme (v3.1-162) packages.

All Sholl-related measurements were analyzed using linear mixed-effects models (LMMs) for continuous outcomes (Total_Branches) and generalized linear mixed-effects models (GLMMs) for count outcomes (Number_of_Branches, Total_Intersections and Max_Intersections). Genotype was treated as a fixed effect, and mouse identity was included as a random effect to account for repeated measurements from individual animals. In R, the model was specified as:

Outcome ~ Genotype + (1 | Mouse_ID)

For count data, model adequacy was evaluated via DHARMa residual simulations; when significant overdispersion or distributional deviations were detected (test$Dispersion P < 0.05 or Kolmogorov–Smirnov test P < 0.05), models were refitted with negative-binomial GLMMs (NB-GLMMs) with the same random-effects structure to accommodate the excess variance and provide a more accurate fit to the true underlying data distribution. If a Poisson GLMM yielded a singular random-effect variance (indicating negligible between-mouse variation), a Poisson generalized linear model (GLM) was used instead, with negative-binomial GLMs (NB-GLM) employed when overdispersion was present (dispersion > 1.2).

Continuous variables were assessed for heteroscedasticity; if variance heterogeneity across genotypes was observed (Breusch-Pagan test P < 0.05), weighted LMMs using the nlme (v3.1-162) package were applied. All post hoc pairwise comparisons were adjusted using the Bonferroni correction. Significance was set at adjusted P < 0.05.

We have now revised the Method section and Figure legends and provided all raw data and full statistical outputs in Source Data. Code is available at https://github.com/YelinZhao-A/Microglia_BHLHE41 .

6. Lines 184–185: Increased TdTomato intensity in B41KO is not sufficient to claim self-repression of Bhlhe41. The authors should consider adding a citation that experimentally validates the self-repression mechanism (e.g., using dual-luciferase assays or H3K27ac data). Even if such validation is performed in other immune cell types, this would provide stronger support for a self-repressing mechanism in microglia. While self-repression likely explains the observed pattern of Bhlhe41 expression across genotypes and models, it has not been directly tested in this study. This limitation should be clearly stated.
- Response: We thank the reviewer for the important point regarding mechanistic evidence. We have provided key supportive data: Bhlhe41 KO increases, while its overexpression decreases, its own promoter activity, and ChIP-qPCR analysis confirms its binding to self-promoter. These findings are consistent with a model of negative autoregulation. However, we do agree that definitive proof of direct transcriptional repression (e.g., via luciferase assays) is an essential next step. As suggested, we have revised the manuscript to use more cautious language in the Results section and discuss this limitation in the Discussion section. This adjustment ensures our claims are precise and aligned with the evidence presented.

7. Figure 3b: The scale bar is missing.
- Response: We thank the reviewer for pointing this out. The scale bar was present but rendered in white, making it difficult to read against the light background. We have revised Fig. 3b by moving the scale bar to a darker background to ensure clear visibility.

8. Figure 3d: Individual data points are obscured within bar graphs, and the data were analyzed using two-way ANOVA. Linear mixed-effects models could also be applied here.
- Response: We thank the reviewer for these statistical and presentation suggestions. We have changed the bar chart with individual data points shown in Figure 3d and 4f. Each data point in Fig. 3d and 4f represents the average number of myelinated and demyelinated axons. We have also reperformed the statistical analysis using LMM. We have revised the figures and figure legends accordingly.

9. Line 334: Microglial morphology cannot be “impaired”, altered or similar adjectives

would be more appropriate.
- Response: We thank the reviewer for pointing out this issue and agree that microglial morphology cannot be impaired. Considering the limited characters for the subtitle, we have now changed to text in line 334 to"Aurka loss impairs Bhlhe41, microglial function and remyelination". Accordingly, we have changed the title of Fig. 4 to "Loss of Aurka inhibits Bhlhe41 expression, and disrupts microglial morphology, phagocytic function and responses to remyelination."

10. Figure 4a: Quantification shows a 50% reduction of Aurka in Cx3cr1Cre/+ Aurka fl/+ microglia, but the representative Western blot image does not support this. Is Aurka also self-repressing?
- Response: We thank the reviewer for this critical observation. Aurka is not self-repressed. As the original representative image in Fig. 4c (now Fig 4a) did not optimally illustrate the quantification, we have now replaced it with a new, clearer representative blot that better aligns with the statistical data. Furthermore, to ensure full transparency, we have provided all uncropped, raw Western blot images in Source Data.

11. Supplementary Figure 6: WT controls are missing. Aurka fl/+ shows 50% Aurka expression and reduced Bhlhe41 expression and therefore cannot serve as proper controls. WT samples should be included.
- Response: We fully agree with the reviewer that WT mice should be used as controls. We have now included WT control data to Fig. S6. The revised Fig. S6 now includes WT, cHET and cKO genotypes, with statistical analysis focusing on comparison between WT and cKO genotypes.

12. Line 516: "Aurka-medicated" should be corrected to "Aurka-mediated."
- Response: We thank the reviewer for pointing out this typo. We have corrected it.

13. Supplementary Figure 10b: Validation of ectopic Bhlhe41 expression should be performed in microglial cells rather than B cells.
- Response: We thank the reviewer for this critical suggestion. We have now validated the ectopic Bhlhe41 expression by Western blot analysis of microglia from $B41^{HET}R26^{B41\text{-}EYFP/+}$ *mice. The new data has been incorporated into Fig. S10b, and the corresponding Figure legend, Methods and Results section have been updated accordingly.*

14. Figure 6d: How does the negative control (secondary antibody only) look? Including this would help distinguish specific signal from background.
- Response: We thank the reviewer for this important suggestion. We have now performed the mouse IgG1 primary antibody/secondary antibody control staining on brain sections from both young and aged mice (20 months). As suggested, these control images (now provided in new Fig. S7a) show no detectable signal, confirming the specificity of the CD22 immunostaining. Consistent with the previous report[1], the

specific CD22 antibody staining clearly reveals an aging-related upregulation. We have added this to the Result section.

15. CD22 blockade: Is the restoration of microglial phagocytosis and improved recovery during remyelination driven specifically by CD22 blockade in microglia? Given that CD22 is highly expressed in oligodendrocytes and minimally in microglia (as shown in this and cited human scRNA-seq studies), a multicellular mechanism may be involved. This should be discussed.

- Response: We thank the reviewer for raising this critical point regarding the cellular mechanism of CD22 blockade. A previous study and our analysis (Fig. 8a) show that CD22 is mainly expressed in oligodendrocytes in human CNS[2]. However, CD22 is restricted to microglia within the mouse CNS[1]. Our new staining data also confirmed that CD22 was localized to microglia in mouse brain, with no detectable signal in oligodendrocytes (Fig. S7a). However, we fully agree with the reviewer and the editor that a multicellular mechanism could be operative given that peripheral immune cells such as B cells also express CD22. We have discussed this as a limitation in the Discussion section.

One major issue remains related to data sharing and reproducibility, and in particular re: the use and reporting of statistical results and procedures that, while greatly improved compared to the original submission, is still lacking critical details, and revealed mis-application of statistical tests in several of the reported analyses.

Critical statistical details are still missing: for each contrast presented in the main text and figures not only the level of significance but also the exact p-value, effect size with unit (unless standardized) and 95% confidence interval, test statistic with df, etc. depending on test used (see for example statistical reports generated with the report R package that follows APA standards).

Also, when reporting the use of a linear model it is not sufficient to just say “a linear model was used”. Instead, the exact model specification should be reported, e.g., using the R formula syntax or conventional statistical model notation (the aforementioned R package can also help with the reporting of linear model fits and contrasts). If the authors prefer to avoid crowding figure legends with statistical details, one option is to have “lean” figure legends reporting only the most important contrasts with full details, and the same figure legends or a spreadsheet with all statistical details in Suppl. Information.

- Response: We sincerely thank the reviewer for these critical comments regarding statistical reporting. We have undertaken comprehensive statistical revisions, ensuring full transparency and reproducibility.

To ensure statistical transparency and reproducibility while keeping concise figure legends, we have provided all raw data together with compressed statistical outputs as Source Data. The statistical outputs include the tests used, exact P values or adjusted

P value, enabling the exact reproduction of every analysis. In revised figures, we have now provided exact P value if larger than 0.0001.

Regarding Sholl statistical analysis, we have now provided a detailed description in the revised Method section and a brief text in the revised Figure legends (see our responses to Comment "5. Sholl measurements:"). The corresponding codes has now been updated in https://github.com/YelinZhao-A/Microglia_BHLHE41.

Please also note that sample size should report what "n" refers to (e.g., n = 4 mice per genotype, or n = 4 fields of view per mouse, 3 mice per genotype, when repeated measures are involved) and that figure 1 is missing the statistical procedures used for panels c-g.
- Response: We thank the reviewer for this comment. All figure legends have been revised to explicitly define the sample size 'n'.

Regarding Fig. 1c, we have added descriptive proportions of dTomato^{+} cells from four major CNS populations. Since this figure aims to show microglia-enriched Bhlhe41 expression rather than formally compare Bhlhe41^{+} differences between cell types, we have not performed inferential statistical tests, and the legend now clarifies this descriptive nature.

Regarding Fig 1e–g (now revised Fig 1d-f), as well as Fig S2 and Fig 6c-e, we presented EYFP^{+} patterns in CNS cells from B41^{HET}Rosa26$^{EYFP/+}$ or B41^{HET}R26$^{B41\text{-}EYFP/+}$ mice while WT or *B41^{HET} are shown to establish baseline EYFP negativity. As the primary aim is to visualize Bhlhe41 expression patterns (EYFP^{+}) rather than quantify EYFP^{+} differences between genotypes, we have now removed B41^{HET} data in Fig1e-g (now Fig 1d-f) and presented these as descriptive data without inferential statistics. We have clarified in Figure legends that these data are presented descriptively without inferential statistics.*

Mis-application of statistical tests: Most if not all the statistical tests used, given the small sample sizes, assume normally distributed, independent, unbiased, and homoskedastic residuals and an unbounded, continuous outcome variable. Great care needs to be taken when using these test with bounded and/or discrete outcome variables, and/or the model assumptions are clearly violated (e.g., when using counts and proportions as outcome variables and data points are near the lower and/or upper bounds, e.g., figures 1f-g, 3g, 4b-c, or when pseudoreplicates are fed to a test like the t-test, which assumes independence of the data points, e.g., figure 8e and g). Because of these concerns, I would like to request a statistical review of this manuscript.

- Response: We sincerely thank the reviewer for raising these critical points regarding the proper application of statistical tests. We agree with the reviewer and have comprehensively revised our statistical approach in response to these concerns, applying statistical frameworks appropriate for each specific data type and

experimental design.

Statistical analyses were performed using R (v4.2.2) with the following packages: lme4 (v1.1-33), lmerTest (v3.1-3), emmeans (v1.11.1), DHARMa (v0.4.7), MASS (v7.3-60), nlme (v3.1-162), rstatix (v0.7.2) and ARTool (v0.11.2).Genotype, treatment/model phase, CNS region and axon status were treated as categorical variables. Prior to analysis, data distributions were assessed using Q-Q plots and/or Shapiro-Wilk test, and homogeneity of variance was evaluated using Levene's test. When appropriate, outcome variables were log2-transformed to meet normality assumptions.

For bounded or non-normal continuous outcomes, non-parametric methods were applied. For two-group comparisons of continuous outcomes meeting parametric assumptions, Student's t-test or paired t-test was used when variances were equal; Welch's t-test was applied if variances were unequal. When normality assumptions were violated or sample sizes were very small, Wilcoxon rank-sum tests were used. For comparisons involving more than two groups, one-way or two-way ANOVA was applied, including interaction terms when multiple factors were modeled. When assumptions of normality or homogeneity were violated, or for bounded continuous data, Kruskal-Wallis tests (non-factorial designs) or Aligned Rank Transform (ART) ANOVA (factorial designs) were applied.

Group differences in count outcomes were analyzed using generalized linear models (GLMs) with either a Poisson or negative binomial distribution, selected based on the presence of overdispersion. Model fit was assessed using Pearson residuals, and overdispersion was evaluated. For experiments with repeated measurements from individual animals, linear mixed-effects models (LMMs) were applied. Mouse identity was included as a random intercept to account for within-animal correlation, while genotype, treatment/model phase, axon status, and their interactions were treated as fixed effects. Model assumptions were evaluated using DHARMa residual diagnostics, including tests for goodness-of-fit (testUniformity) and dispersion (testDispersion). In the presence of heteroscedasticity, weighted mixed-effects models were fitted using the nlme package. All post hoc pairwise comparisons were performed using estimated marginal means (EMMeans) with Bonferroni correction to adjust for multiple testing.

Our specific revisions in response to each point are as follows:

1. Addressing Pseudo-replication and Non-independence (Figures 8e and 8g):
We agree that applying t-tests to single-cell data can result in pseudoreplication and violate assumptions of independence and normality. In response, we have re-analyzed these data using a pseudobulk approach. Single-cell data are now aggregated to the donor level by calculating the mean BHLHE41-CD22 Tendency Score per donor per cluster. All statistical comparisons now use independent biological replicates (n ≥ 20 donors). Statistical comparisons between clusters are performed using a two-sided Wilcoxon rank-sum test, which does not assume normality and correctly operates on

independent donor-level data points.

2. Analyzing Bounded/Proportion, Discrete Data or small-size data (Figures 1b, 2c,3g, new 4a, S1d-e, S4b-c, S8, S9a, S10b):
We have reanalyzed these data using the nonparametric tests such as Kruskal-Wallis test with Dunn's post-hoc test or ART-ANOVA. This approach makes no assumptions of normality and is more appropriate for bounded data or non-normally distributed data.

We acknowledge that some adjusted P values for WB comparisons are > 0.05 despite visible clear and consistent biological trends. This is likely due to the reduced statistical power of non-parametric tests with multiple comparison correction when applied to small number, variable datasets like WB quantification (n=3-5). Critically, the consistent directional change in WB across replicates and models (knockdown, knockout and transgenic) fully aligns with the highly significant phenotypic results. Our conclusion is based on this convergent evidence, not on a single pairwise P value.

3. Correcting the Sholl Analyses
All Sholl-related metrics, which involve repeated measurements within each mouse, were re-analyzed using linear mixed-effects models (LMMs) for continuous outcomes and generalized linear mixed-effects models (GLMMs) for count outcomes. Mouse identity was included as a random effect to account for repeated measures and avoid pseudoreplication. For count outcomes, if a Poisson GLMM yielded a singular random-effect variance (indicating negligible between-mouse variation), a Poisson GLM was used instead; negative-binomial GLMs were applied when overdispersion was detected (dispersion > 1.2). For continuous outcomes, residual normality and homoskedasticity were assessed (QQ-plots, Breusch–Pagan test), and weighted LMMs were used if heteroscedasticity was detected.

4. Clarifying descriptive data without inferential statistical tests (e.g., revised Fig. 1c-f, 6c-e for EYFP and S2):
We wish to clarify that for Fig 1c aims to show microglia-enriched Bhlhe41 expression rather than formally compare Bhlhe41^{+} differences between cell types, we have not performed inferential statistical tests.

For Fig 1e–g (now revised as Fig 1d-f), as well as Fig S2 and Fig 6c-e, we presented EYFP^{+} patterns in CNS cells from B41^{HET}Rosa26$^{EYFP/+}$ or B41^{HET}R26$^{B41\text{-}EYFP/+}$ mice while WT or *B41^{HET} are shown to establish baseline EYFP negativity. As the primary aim is to visualize Bhlhe41 expression patterns (EYFP^{+}) rather than quantify EYFP^{+} differences between genotypes, we have now removed B41^{HET} data in Fig1e-g (now Fig 1d-f) and presented these as descriptive data without inferential statistics. We have clarified in Figure legends that these data are presented descriptively without inferential statistics.*

In summary, we have re-run all statistical tests throughout the manuscript and have

updated the "Statistical Analysis" section in the Methods and the relevant figure legends to detail these new approaches. In addition, we have provided the complete raw datasets and detailed statistical outputs in Source Data.

We are grateful to the reviewer for their insightful critique, which has significantly strengthened the statistical rigor of our manuscript.

Data sharing and reproducibility: All raw data, metadata, and computational notebooks/scripts that were used to generate all statistical results and plots shown in the article (not just those related to transcriptomic data), should be made available as Supplementary Info. and/or shared in publicly accessible repositories at the time of publication. Data available upon request is no longer an acceptable stance. The code that is currently available on the github repository is not sufficient.
- Response: We have provided all raw data and complete statistical outputs in Source Data and all scripts for scRNA-seq analysis and statistical analyses are updated on GitHub (https://github.com/YelinZhao-A/Microglia_BHLHE41).

Reviewer #2 (Remarks to the Author):

I co-reviewed this manuscript with one of the reviewers who provided the listed reports. This is part of the Nature Communications initiative to facilitate training in peer review and to provide appropriate recognition for Early Career Researchers who co-review manuscripts.
- Response: We thank Reviewer #2 for their time and contribution to the peer review of our manuscript.

Reviewer #4 (Remarks to the Author):
This is a revised manuscript aimed at studying the function of Aurka/Bhlhe41 in the context of myelin repair. I want to commend the authors for their work on this revised manuscript; overall, I feel that it has been significantly strengthened. The study used an impressive combination of genetic mouse models and pharmaceutical intervention to show that Bhlhe41- or Aurka-deficient mice have microglia that exhibit aging characteristics, consisting of altered morphology, phagocytic deficits, progressive CD22 upregulation, leading to remyelination impairment in cuprizone-induced demyelination. I have a few minor suggestions for the authors.

The diversity of phagocytic cargo used is confusing. Why do the authors utilize E. coli and zymosan, which are not biologically relevant to the study of remyelination? A refocus on myelin debris and apoptotic cells could be more informative.
- Response: We thank the reviewer for this important question regarding the phagocytic assays.

Our initial observation that Bhlhe41-deficient microglia exhibited dystrophic morphology led us to hypothesize a broad functional deficit. To test this, we first

employed commercialized phagocytic targets (fluorescent labeled E. coli and zymosan) as a general and sensitive screening tool to examine phagocytic function.

Subsequently, and central to the focus of our study on remyelination, we assessed the phagocytosis of biologically relevant cargo using in-house-prepared, Dil-labeled myelin debris. We agree that the myelin debris assay is the most biologically relevant, and we have featured it in our results to address the core question of remyelination.

The authors should be careful about their conclusions regarding phagocytosis; only Phrodo-labeled molecules (Fig. 2d for example) will allow the team to discern between internalization versus binding.
- Response: We thank the reviewer for raising this essential technical point regarding the specificity of phagocytosis measurement. We fully agree that distinguishing internalization from surface binding is critical.

Our 4°C control samples, in which phagocytosis is arrested, showed no detectable pHrodo signal (Fig. 2c-d). This result, combined with the robust signal observed at 37°C, definitively confirms that the measured fluorescence originates from internalized cargo within acidic phagolysosomes, not from particles bound to the cell surface (Fig. 2c-d). Therefore, the phagocytic deficit we report in Fig. 2c-d is defined as a failure in cargo internalization.

For experiments using non-pH-sensitive fluorophores (AF488-zymosan), we employed a standardized protocol including extensive washing during flow cytometry sample preparation to minimize surface-bound signal. While this method does not discriminate internalization with the same molecular specificity as pHrodo, it provides a functional readout of overall cargo clearance. Importantly, the increased residual fluorescence detected by immunofluorescence analysis in the KO mice further supports the conclusion of reduced phagocytic clearance, which is fully consistent with the specific internalization defect revealed by our pHrodo-based assays.

The authors may want to include in the text that OLIG2 stains all cells of the oligodendrocyte lineage (OPCs and oligodendrocytes).
- Response: We thank the reviewer for this helpful clarification. We have updated the Results section and the Fig legends to explicitly state that OLIG2 is a pan-oligodendrocyte lineage cell marker, staining both oligodendrocyte progenitor cells (OPCs) and mature oligodendrocytes.

Fig 4a-b should be moved to a supplementary figure.
- Response: We thank the reviewer for this constructive suggestion. We have moved Fig. 4a-b to Supplementary Figure 4 (now Fig. S4a-b).

References

1. Pluvinage, J.V., et al., *CD22 blockade restores homeostatic microglial*

phagocytosis in ageing brains. Nature, 2019. **568**(7751): p. 187-192.
2. Pluvinage, J.V., et al., *The CD22-IGF2R interaction is a therapeutic target for microglial lysosome dysfunction in Niemann-Pick type C.* Sci Transl Med, 2021. **13**(622): p. eabg2919.

Response to referees

REVIEWERS' COMMENTS

Reviewer #1 (Remarks to the Author):

I thank the authors for their careful and thorough responses to my previous comments. The revised manuscript has satisfactorily addressed all of my major concerns.

I have no further substantive comments and consider the manuscript suitable for publication.

Reviewer #1 (Remarks on code availability):

I did not independently run the code. However, the repository appears well organized and publicly accessible, with sufficient documentation to allow inspection and reuse by the community. The availability of the code supports transparency and reproducibility.

Responses: We thank the reviewer again for their constructive comments, which have greatly improved the rigor of our work.

Reviewer #2 (Remarks to the Author):

I co-reviewed this manuscript with one of the reviewers who provided the listed reports. This is part of the Nature Communications initiative to facilitate training in peer review and to provide appropriate recognition for Early Career Researchers who co-review manuscripts.

Responses: We thank the reviewer again for their constructive comments, which have greatly improved the rigor of our work.

The authors did a good job revising the manuscript and addressed most of my concerns regarding WT controls, statistical analysis, and demyelination assays. There are a few **minor issues** that should be addressed prior to publication:

1. **Flow cytometry histograms:** The Y-axes on the gMFI histograms are labeled inconsistently as "Normalized to Mode" or "Normalized to Model." This typo should be corrected, and the meaning of "Mode" or "Model" should be explained in the figure legend.
2. **Figures 1a and 4a:** Two-way ANOVA was used to analyze the data. Were the three groups compared at each time point, or were AUC values compared between genotypes?
3. **Figure 3:** Two-way ANOVA was employed, most likely to test interactions between phase and genotype, but the interaction results were not reported.
4. **Supplementary Figure 1c:** This figure does not seem to show an age-dependent increase in Bhlhe41 expression as stated in line 180. Instead it shows a gating strategy to sort or analyze microglia.
5. **Sholl measurements:** It is appreciated that the authors used mixed models, which allow them to fully leverage their dataset. However, the statistical details provided are too minimal. The authors report using the `lme4` package, but the package version (v.X.X.X) is missing. What were considered fixed and random effects? Ideally, the authors should share the model formula and raw data for reproducibility.
6. **Lines 184–185:** Increased TdTomato intensity in B41KO is not sufficient to claim self-repression of Bhlhe41. The authors should consider adding a citation that experimentally validates the self-repression mechanism (e.g., using dual-luciferase assays or H3K27ac data). Even if such validation is performed in other immune cell types, this would provide stronger support for a self-repressing mechanism in microglia. While self-repression likely explains the observed pattern of Bhlhe41 expression across genotypes and models, it has not been directly tested in this study. This limitation should be clearly stated.
7. **Figure 3b:** The scale bar is missing.
8. **Figure 3d:** Individual data points are obscured within bar graphs, and the data were analyzed using two-way ANOVA. Linear mixed-effects models could also be applied here.
9. **Line 334**: Microglial morphology cannot be "impaired", altered or similar adjectives would be more appropriate.
10. **Figure 4a:** Quantification shows a 50% reduction of Aurka in Cx3cr1Cre/+ Aurka fl/+ microglia, but the representative Western blot image does not support this. Is Aurka also self-repressing?
11. **Supplementary Figure 6:** WT controls are missing. Aurka fl/+ shows 50% Aurka expression and reduced Bhlhe41 expression and therefore cannot serve as proper controls. WT samples should be included.

12. **Line 516:** "Aurka-medicated" should be corrected to "Aurka-mediated."
13. **Supplementary Figure 10b:** Validation of ectopic Bhlhe41 expression should be performed in microglial cells rather than B cells.
14. **Figure 6d:** How does the negative control (secondary antibody only) look? Including this would help distinguish specific signal from background.
15. **CD22 blockade:** Is the restoration of microglial phagocytosis and improved recovery during remyelination driven specifically by CD22 blockade in microglia? Given that CD22 is highly expressed in oligodendrocytes and minimally in microglia (as shown in this and cited human scRNA-seq studies), a multicellular mechanism may be involved. This should be discussed.

One major issue remains related to data sharing and reproducibility, and in particular re: the use and reporting of statistical results and procedures that, while greatly improved compared to the original submission, is still lacking critical details, and revealed mis-application of statistical tests in several of the reported analyses.

Critical statistical details are still missing: for each contrast presented in the main text and figures not only the level of significance but also the exact p-value, effect size with unit (unless standardized) and 95% confidence interval, test statistic with df, etc. depending on test used (see for example statistical reports generated with the report R package that follows APA standards). Also, when reporting the use of a linear model it is not sufficient to just say "a linear model was used". Instead, the exact model specification should be reported, e.g., using the R formula syntax or conventional statistical model notation (the aforementioned R package can also help with the reporting of linear model fits and contrasts).

If the authors prefer to avoid crowding figure legends with statistical details, one option is to have "lean" figure legends reporting only the most important contrasts with full details, and the same figure legends or a spreadsheet with all statistical details in Suppl. Information.

Please also note that sample size should report what "n" refers to (e.g., n = 4 mice per genotype, or n = 4 fields of view per mouse, 3 mice per genotype, when repeated measures are involved) and that figure 1 is missing the statistical procedures used for panels c-g.

Mis-application of statistical tests: Most if not all the statistical tests used, given the small sample sizes, assume normally distributed, independent, unbiased, and homoskedastic residuals and an unbounded, continuous outcome variable. Great care needs to be taken when using these test with bounded and/or discrete outcome variables, and/or the model assumptions are clearly violated (e.g., when using counts and proportions as outcome variables and data points are near the lower and/or upper bounds, e.g., figures 1f-g, 3g, 4b-c, or when pseudoreplicates are fed to a

test like the t-test, which assumes independence of the data points, e.g., figure 8e and g). Because of these concerns, I would like to request a statistical review of this manuscript.

Data sharing and reproducibility: All raw data, metadata, and computational notebooks/scripts that were used to generate all statistical results and plots shown in the article (not just those related to transcriptomic data), should be made available as Supplementary Info. and/or shared in publicly accessible repositories at the time of publication. Data available upon request is no longer an acceptable stance. The code that is currently available on the github repository is not sufficient.
